# Studies on Isoniazid Derivatives through a Medicinal Chemistry Approach for the Identification of New Inhibitors of Urease and Inflammatory Markers

**DOI:** 10.1038/s41598-019-43082-0

**Published:** 2019-05-01

**Authors:** Fazila Rizvi, Majid Khan, Almas Jabeen, Hina Siddiqui, M. Iqbal Choudhary

**Affiliations:** 1 H.E.J. Research Institute of Chemistry, International Center for Chemical and Biological Sciences, University of Karachi, Karachi, 75270 Pakistan; 2 Dr. Panjwani Center for Molecular Medicine and Drug Research, International Center for Chemical and Biological Sciences, University of Karachi, Karachi, 75270 Pakistan; 3 Department of Biochemistry, Faculty of Science, King Abdulaziz University, Jeddah, 21452 Saudi Arabia

**Keywords:** Drug discovery and development, Drug discovery and development

## Abstract

A library of thiosemicarbazide derivatives of isoniazid **3–27**, was synthesized and evaluated for their anti-inflammatory and urease inhibition activities, by using *in vitro* bioassays. Among these compounds **9**, **10**, **12**, **21**, and **26** were identified as new derivatives. Prolonged use of non-steroidal anti-inflammatory drugs (NSAIDs) and infections caused by *Helicobacter pylori* (ureolytic bacteria), are the two most significant causes of gastric and peptic ulcers. We focused on the identification of the dual inhibitors of inflammation and urease enzyme. Compound **23** was identified as the best dual inhibitor of inflammation (ROS; IC_50_ = 12.3 µg/mL), and urease enzyme inhibition activity (IC_50_ = 22.4 µM). Many of these compounds showed comparable activities to the standard anti-inflammatory drug (ibuprofen, IC_50_ = 11.2 µg/mL) and urease inhibitor (thiourea/acetohydraoxamic acid, IC_50_ = 21.1/20.3 µM). Compound **12** was found to be the most potent urease inhibitor (IC_50_ = 12.3 µM) and good inhibitor of inflammation (IC_50_ = 27.7 µg/mL). Compounds **19**, **11**, **13**, **9**, **17**, **10**, and **16**, were also found to be potent inhibitors of urease. Cytotoxicity was also evaluated and all the compounds were found to be non-cytotoxic, except compound **18** and the parent drug isoniazid (IC_50_ = 29.5 and 28.5 µM, respectively).

## Introduction

Urease (urea amidohydrolase; E.C. 3.5.1.5) is a nickel-containing enzyme produced by plants, bacteria, fungi and parasites. Urease catalyzes the hydrolysis of urea into ammonia and carbon dioxide which increases the pH of stomach of the host^1,2^. The produced ammonia may cause several diseases, such as hepatic encephalopathy, gastric and peptic ulcers, atherosclerosis or rheumatoid arthritis. Urease produced by the *Helicobacter pylori* serve as a virulence factor through increasing the pH of the stomach, which helps the bacteria to colonize in the acidic environment of stomach and causes gastritis and peptic ulcers. Therefore, urease inhibitors serves as the anti-ulcer drugs^3^.

Inflammation is the host defense mechanism which protect the body from harmful stimuli and speeds up the restoration process^4^. The stimulus can be any microbial infection or chemicals. The inflammation is characterized with redness, pain, warmth, swelling and lack of function in the injured region^5^. The inadequate healing process of the wounds or any other dysfunction will result in a chronic inflammation which need to be treated^6^.

### Currently Available Marketed Drugs and Their Side Effects

**Figure 1:**
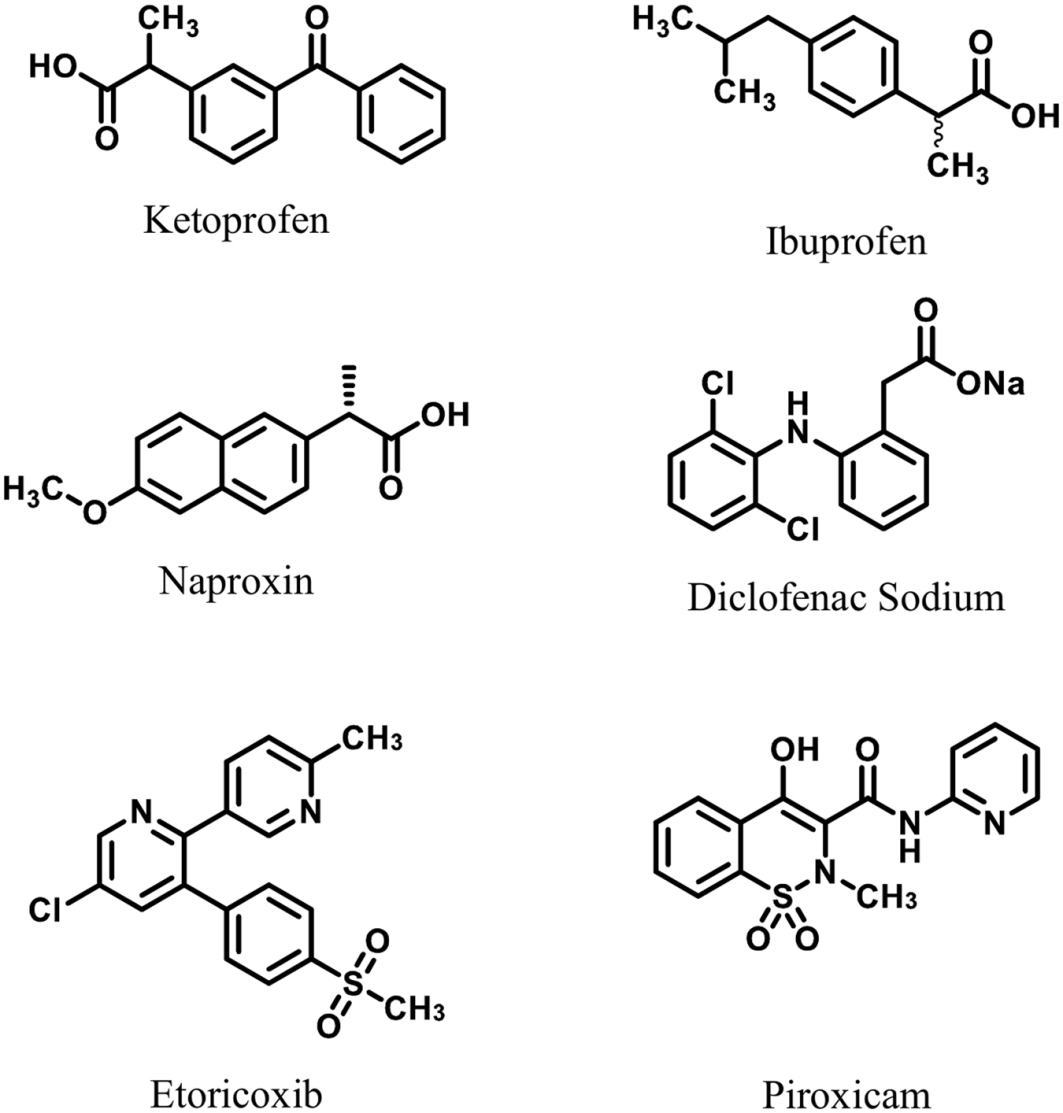
Examples of Non-Steroidal Anti-inflammatory Drugs.

Globally used drugs for the treatment of inflammation and associated conditions, such as traumatic injuries, arthritis, fever, and pain, are non-steroidal anti-inflammatory drugs (NSAIDs), such as ketoprofen, ibuprofen, naproxin, diclofenac sodium, piroxicam, and etoricoxib^7^ (Fig. 1). These drugs are the selective inhibitors of cyclooxygenase-2 (COX-2) enzyme^6^. The major side effects caused by the NSAIDS are ulceration and gastrointestinal (GI) hemorrhage^8^. This has attracted the attention of the scientists towards the development of the new anti-inflammatory agents with no or less side effects^9^.

**Figure 2:**
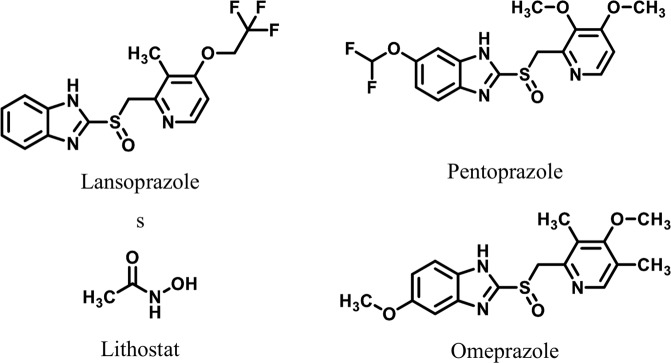
Examples of Urease Inhibitors Used as Anti-Ulcer Drugs.

The drugs currently available for the treatment of ulceration and gsastrointestinal (GI) hemorrhage include pantoprazole, lansoprazole, lithostat, and omeprazole^10^ (Fig. 2). A study by Saniee.*et al*. (2015) showed that the proton pump inhibitors (PPI), such as omeprazole and lansoprazole, are also the urease inhibitors^11^. Due to the high prevalence of gastroesophageal reflux disease the use of PPIs has increased remarkably and now they are among the most frequently prescribed medicines globally^12^. Adverse effects of PPIs has also been observed such as nutritional deficiencies, visual impairment, chronic kidney disease^13^, dementia, infections, nervous system related abnormalities. The most adverse effect is the decreased in the bacterial richness and alteration in the gut microbiome. Approximately 65% increase in the risk of enteric infections development especially *Clostridium difficile* infection has been reported^14^, which limit clinical applications of PPIs^15^.

### A Medicinal Chemistry Approach of Drug Discovery

Drug development is a time-intensive, costly, and high-risk process. One approach that has attracted a lot of attention in modern drug discovery is drug repositioning or repurposing^16^. Drug repurposing include cases in which a current drug, endorsed by an administrative organization for a particular disease, is found to have effect against another illness. Conversely, drug repositioning also depicts a condition where a drug that is in use for a disease is utilized as a template for the synthesis of new analogs possessing activity against another disease^17^. Drug repositioning thus essentially shorten the drug development process and thus decrease the discovery cost^18^.

**Figure 3:**
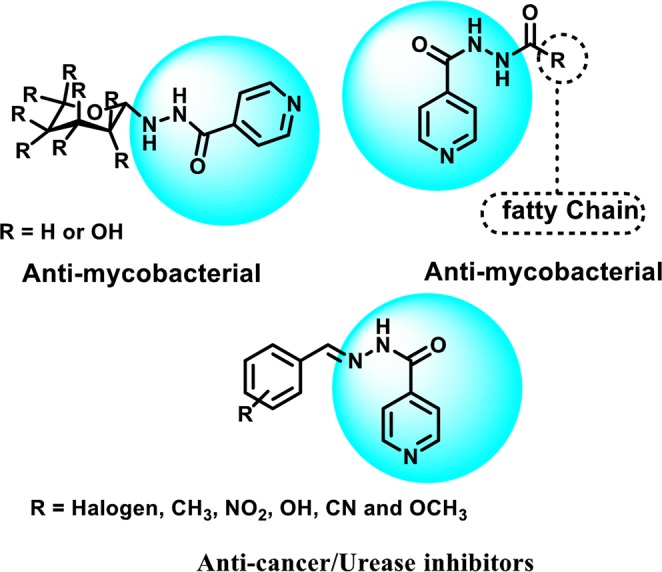
Some previously reported derivatives of isoniazid.

**Figure 4:**
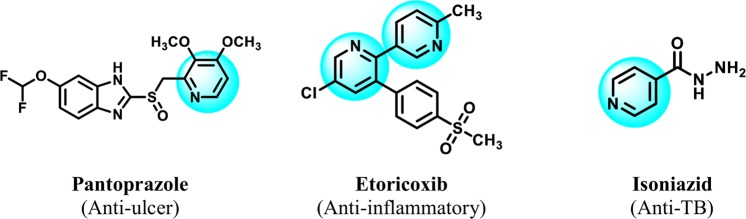
Component structural similarities between isoniazid, etoricoxib, and pantoprazole.

Current study describes the repositioning of isoniazid, an anti-bacterial agent. Isoniazid, was synthesized in 1952 for the treatment of tuberculosis^19^. The recommended daily dose of isoniazid is from 5–300 mg/day, which rarely causes side effects in individuals^20^. The use of isoniazid as the main scaffold for the synthesis of medicinally important compounds is well known as reported in the literature^21–24^ (Fig. 3). Therefore, we have randomly synthesized the library of compounds (**3–27**) followed by random screening against various biological targets. It was observed that some of these compounds are the significant dual inhibitors of inflammation, and urease. The structural similarity of synthesized compounds with the pyridine based anti-ulcer drug pantoprazole^23^, and anti-inflammatory drug etoricoxib^25^ may be the reason for the activities of these compounds (Fig. 4).

**Figure 5:**
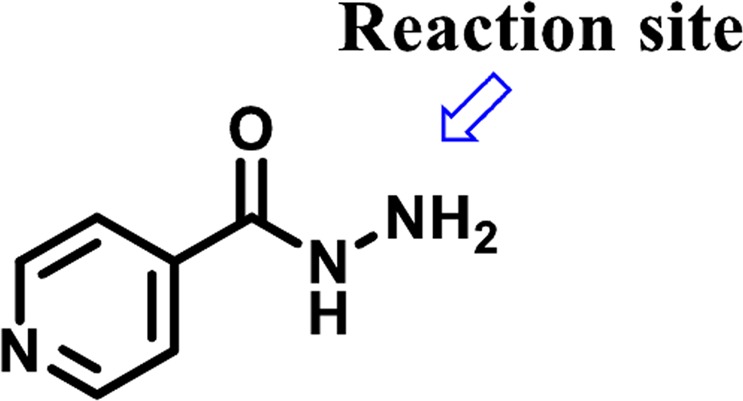
Isoniazid (**1**).

During the current study, we have synthesized thiosemicarbazide derivatives of isoniazid (**3–27**) through modification at terminal NH_2_ (Fig. 5) by reacting with different isothiocyanates. Thiosemicarbazide class of compounds possess the diverse biological activities, such as anti-cancer^26^, anti-fungal^27^, anti-helminthic^28^, anti-bacterial^29^ and anti-HIV^30^. Among synthesized compounds, all were identified as previously known^31–38^, except **9**, **10**, **12**, **21**, and **26**. However, these compounds have not been reported as the dual inhibitor of inflammation and urease. Cytotoxicity of these compounds were also evaluated against 3T3 mouse fibroblast cell line.

## Results

### Chemistry

**Figure 6:**
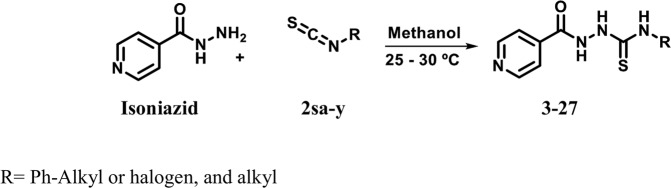
Synthesis of thiosemicarbazide derivatives of isoniazid (**3–27**).

Thiosemicarbazide derivatives of isoniazid (**3–27**) were synthesized by its reaction with various isothiocyanates using method reported by Yahyazadeh. *et al*. (2013) with slight modification^39^ (Fig. 6). The resulting product was purified through solvent-solvent extraction with hexane and ethyl acetate, followed by recrystallization with methanol. Structures of synthesized compounds were elucidated by using different spectroscopic techniques, such as mass spectrometry, infrared spectrophotometry, and ^1^H- and ^13^C-NMR spectroscopy. Among synthesized compounds, **9**, **10**, **12**, **21**, and **26** were found to be new.

#### Structure Elucidation of Representative Compound **4**

**Figure 7:**
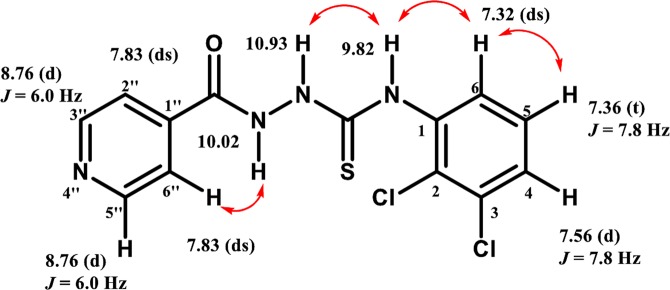
NMR and Key 2D NOSEY correlations of *N*-(2, 3-Dichlorophenyl)−2-isonicotinoylhydrazinecarbothioamide (**4**).

The structure elucidation of the synthesized thiosemicarbazide derivatives (**3–27**) was performed through various spectroscopic techniques (MS, NMR, and IR). Structure elucidation of compound **4**, a representative member of the library, is presented in Fig. 7.

Singlets of protons attached to nitrogen were at *δ*10.93, 10.02, and 9.82. A doublet at *δ* 8.76 showed two protons of pyridine ring *i*.*e*. H-3″, H-5″ which are coupled with H-2″, H-6″ (*J*_3″,2″_ = *J*_5″,6″_ = 6 Hz). A distorted singlet at *δ* 7.83 represented another two protons of pyridine ring *i*.*e*. H-2″, and H-6″. Doublet appeared at *δ* 7.56 showed H-4 of phenyl ring, coupled with the H-5, with *ortho* coupling (*J*_*4,5*_ = 7.8 Hz), while triplet at *δ* 7.36 showed a proton of phenyl ring *i*.*e*.H-5 which is coupled with H-4 and H-6 with coupling constant (*J*_*5(*4–6)_ = 7.8 Hz). H-6 proton of phenyl ring showed a distorted singlet at *δ* 7.32.

The conformation of the synthesized compound was deduced based on key 2D NOESY correlations (Fig. 7). NH (*δ*9.82, singlet) showed correlations with the H-6 phenyl ring proton (*δ*7.32, distorted singlet), and NH (*δ*10.93, singlet). On the other hand, proton attached to nitrogen NH (*δ*10.02, singlet) showed correlation with pyridine ring H-6″ (*δ*7.83, distorted singlet).

^13^C NMR spectrum of compound **4** showed five characteristic peaks of quaternary carbons at *δ*182.0 (*C*=S), 165.0 (C=O), 140.0 (C-1″), 139.3 (C-1) and 132.1, 131.0 (C-2, C-3). C-3″, C-5″ and C-2″, C-6″ of pyridine ring were resonated at *δ*150.6 and 121.7, while C-4 and C-5 of phenyl ring were resonated at *δ* 128.6 and 127.5, respectively. C-6 of phenyl ring appeared at *δ* 129.9. The HRFAB-MS (+ve mode) was observed at *m/z *341.0017 correlating with the molecular formula [(C_13_H_10_N_4_O_1_Cl_2_S_1_) + H]^+^ (341.0031).

### Bioactivities

#### *In Vitro* Urease Inhibition Activity

Thiosemicarbazide derivatives, synthesized in the current study, were evaluated for their *in vitro* urease inhibitory activity. The standard inhibitor used in the assay was thiourea (IC_50_ = 21.1 ± 0.2 µM) and acetohydroxamic acid (IC_50_ = 20.3 ± 0.4 µM). Most of the synthesized thiosemicarbazides showed urease inhibition activity between the range of 63.2 ± 2.0 µM to 12.3 ± 1.04 µM (Table 1).

**Table 1:** *In vitro* urease Inhibition, anti-inflammatory, and cytotoxicity of synthetic compounds **3–27**.

Compounds	R	Urease Inhibition Activity IC_50_ ± SEM^a^ µM	Anti-Inflammatory Activity IC_50_ ± SD^b^ µg/mL	Cytotoxicity (3T3 Cell line) IC_50_ ± SD^b^ µM
3		Inactive^c^	Inactive	>30
4		38.8 ± 0.35	Inactive	>30
5		36.5 ± 0.14	18.5 ± 1.0	>30
6		Insoluble	Inactive	>30
7		46.3 ± 0.43	Inactive	>30
8		44.4 ± 0.43	Inactive	>30
9		14.2 ± 1.38	26.7 ± 2.5	>30
10		15.7 ± 1.32	Inactive	>30
11		13.2 ± 1.64	Inactive	>30
12		12.3 ± 1.04	27.7 ± 2.4	>30
13		13.6 ± 1.61	Inactive	>30
14		35.8 ± 1.89	Inactive	>30
15		34.1 ± 0.45	Inactive	>30
16		21.5 ± 1.3	29.7 ± 1.7	>30
17		14.7 ± 1.01	36.9 ± 3.0	>30
18		26.3 ± 1.59	Inactive	29.5 ± 1.9
19		12.7 ± 0.8	Inactive	>30
20		22.0 ± 1.15	Inactive	>30
21		28.2 ± 1.48	Inactive	>30
22		22.1 ± 1.91	25.1 ± 0.4	>30
23		22.4 ± 1.83	12.3 ± 1.2	>30
24		63.2 ± 2.0	Inactive	>30
25		Insoluble	Inactive	>30
26		46.8 ± 1.8	25.4 ± 1.3	>30
27		Insoluble^d^	Inactive	>30
28	Isoniazid(1)	Inactive	Inactive	28.5 ± 1.2
29	Thiourea (Standard)	21.1 ± 0.2 (Observed)	—	—
30	Aetohydaoxamic acid (standard)	20.3 ± 0.4 (Observed) 17.2 ± 0.9 (Reported)		
31	Ibuprofen (Standard)	—	11.2 ± 1.9 (observed) 12.97 ± 0.23 (reported)	—
32	Cyclohexanamide (Standard)	—	—	0.8 ± 0.2 (observed) 0.26 ± 0.04 (reported)

**SEM**^a^ is the standard error of the mean, **SD**^**b**^ is the standard deviation, and **Insoluble**^**d**^ refers to those analogue which are partially soluble in HPLC methanol used in the protocol of *in vitro* urease inhibition activity.

Compounds showed <50% inhibition were considered as inactive.

For Urease enzyme inhibition activity, screening concentration was 0.5 mM.

For ROS inhibition assay screening concentration was 25 µg/mL.

Note: All data were presented as mean ± standard deviation/standard error of the mean of three independent experiments where each sample was run in triplicate. The IC_50_ values were obtained using three concentrations of test compound, and were calculated using Excel Based Program.

Compound **12** (IC_50_ = 12.3 ± 1.04 µM), **19** (IC_50_ = 12.7 ± 0.8 µM), **11** (IC_50_ = 13.2 ± 1.64 µM), **13** (IC_50_ 13.6 ± 1.61 µM), **9** (IC_50_ = 14.2 ± 1.38 µM), **17** (IC_50_ = 14.7 ± 1.01 µM), **10** (IC_50_ = 15.7 ± 1.32 µM), and **16** (IC_50_ = 21.5 ± 1.3 µM) were found more potent than the thiourea (Standard: IC_50_ = 21.1 ± 0.2 µM)/acetohydroxamic acid (Standard: IC_50_ = 20.3 ± 0.4 µM). While compounds **20** (IC_50_ = 22.0 ± 1.1 µM), **22** (IC_50_ = 22.1 ± 1.91 µM), and **23** (IC_50_ = 22.4 ± 1.83 µM) showed comparable activity with standard thiourea (IC_50_ = 21.1 ± 0.2 µM)/acetohydroxamic acid (Standard: IC_50_ = 20.3 ± 0.4 µM). Compounds **18** (IC_50_ = 26.3 ± 1.59 µM), **21** (IC_50_ = 28.2 ± 1.48 µM), **15** (IC_50_ = 34.1 ± 0.45 µM), **14** (IC_50_ = 35.8 ± 1.8 µM), **4** (IC_50_ = 38.8 ± 0.35 µM) showed significant activity, while **24** (IC_50_ = 63.2 ± 2.0 µM) showed moderate activity. Whereas, compound **3** and the parent drug isoniazid (**1**) was found to be inactive.

**Figure 8:**
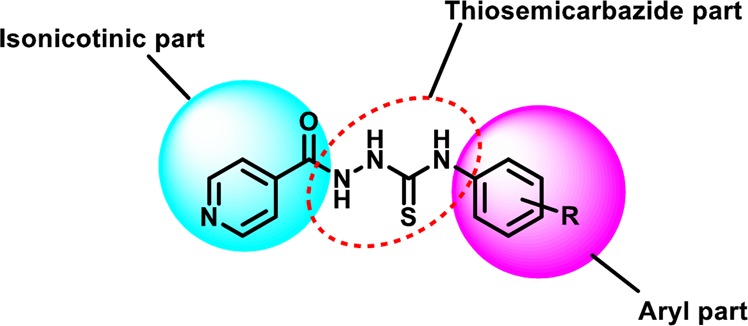
Rationale of the biological activity.

Indeed, the inhibition showed by all the synthesized analogues is due to the mutual participation of all parts of the molecule. However, it is also true that there are some characteristic features, which play an important role in the biological activity. As depicted in the Fig. 8 the current library has only aryl part (R) which is varying. Therefore, only the limited structure activity relationship can be drawn by comparing the position and nature of the substituents present in the aryl part of thiosemicarbazide derivatives (**3–27**).

The compound **24** with no substituents on aryl part showed the lowest activity (IC_50_ = 63.2 ± 2.0 µM) as compared to other members of the library. Substitution of bromo group at the C-3 position as in compound **22** enhances the activity with IC_50_ = 22.1 ± 1.91 µM. While changing the position of bromo group to the C-2 position as in compound **14**, slightly reduces the activity (IC_50_ = 35.8 ± 1.8 µM).

Substitution of chloro group at the C-4 position as in compound **18** showed almost similar activity with the (IC_50_ = 26.3 ± 1.59 µM). Compound **4** possess the two chloro groups at C-2 and C-3 positions and compound **5** with the chloro groups at C-2 and C-5 positions showed reduced activity with the IC_50_ values 38.8 ± 0.35 µM and 36.5 ± 0.14 µM, respectively. The compound with bromo substitution at C-3 position was found to be more efficient in the inhibitory potential as compared to the chloro substituted analogues, similar effect has also been reported by ali. *et al*.^40^.

Compound **12** (IC_50_ = 12.3 ± 1.04 µM), **19** (IC_50_ = 12.7 ± 0.8 µM), **16** (IC_50_ = 21.5 ± 1.3 µM), **15** (IC_50_ = 34.1 ± 0.45 µM), and **7** (IC_50_ = 46.3 ± 0.43 µM), possess fluoro group but they differ in the numbers and positions, compound **19** posses the fluoro group at C-4 positionis found to be the more active (IC_50_ = 12.7 ± 0.8 µM) than the compound **16** (IC_50_ = 21.5 ± 1.3 µM) having the fluoro group at C-3 position among, the disubstituted analogue **15** posses fluoro group at C-2 and C-4 showed inhibition with the IC_50_ value 34.1 ± 0.45 µM. Compound **7** posses the fluoro group at C-2, and C-6 exhibited decrease in the inhibitory potential with the IC_50_ value 46.3 ± 0.43 µM. Penta fluoro substituted compound **12** (IC_50_ = 12.3 ± 1.04 µM) showed the comparable activity as of mono substituted analogue **19**. Similar SAR have been reported by taha. *et al*.^41^.

Incorporation of the chloro and trifluoromethyl group in compound **9** have further enhanced the activity with IC_50_ = 14.2 ± 1.38 µM.

Compound **11** is a *para* substituted derivatives (IC_50_ = 13.2 ± 1.64 µM), which posses an electron withdrawing trifluoromethoxy group, showed comparable activity with compound **10** (IC_50_ = 15.7 ± 1.32 µM) having the cyano group, while compound **20** (IC_50_ = 22.0 ± 1.1 µM) having the electron donating methoxy group showed less activity than the compounds **11** and **10**. It demonstrates that the electron withdrawing groups have the profound effect on inhibitory potential.

From the limited structure activity relationship, it can be summarized that the nature and position of the substituent both are equally responsible for demonstrating the inhibitory activity.

#### *In Vitro* Anti-inflammatory Activity

All synthesized derivatives of isoniazid (**1**) were also evaluated for their anti-inflammatory activity using oxidative burst assay. The standard inhibitor used was ibuprofen (IC_50_ = 11.2 ± 1.9 µg/mL). Among all the compounds, compounds **23**, **5**, **22**, **26**, **9**, **12**, **16**, and **17** showed promising anti-inflammatory activities with IC_50_ value 12.3 ± 1.2 to 36.9 ± 3.0 µg/mL, while other compounds were found inactive (Table 1).

Among these compounds, compound **23** containing a cyclohexyl ring was found to be the most active inhibitor of ROS (IC_50_ = 12.3 ± 1.2 µg/mL). Compound **5** bearing chloro groups at C-2 and C-5 positions showed a good activity with an IC_50_ value 18.5 ± 1.0 µg/mL, while with a bromo group at C-3, compound **22** was also found to be a good inhibitor but less active than compound **5** (IC_50_ = 25.1 ± 0.4 µg/mL). On the other hand, compound **26** with a isothiocyanate group at C-3 showed the similar activity (IC_50_ = 25.4 ± 1.3 µg/mL) as that of compound **5**. Compound **9** with a chloro group at C-4 and trifluoromethyl group at C-3 also showed a good activity (IC_50_ = 26.7 ± 2.5 µg/mL), along with this pentaflouro containing phenyl ring analogue **12** which resulted in decrease of activity from IC_50_ values 26.7 ± 2.5 to 27.7 ± 2.4 µg/mL. Interestingly the mono substituted analogue of fluorine at C-3 (compound **16**) showed a decreased activity with IC_50_ value 29.7 ± 1.7 µg/mL, while the iodine containing analogue at C-3 (compound **17**) showed further decreased activity (IC_50_ = 36.9 ± 3.0 µg/mL).

#### Evaluation of cytotoxicity on 3T3 normal cell line

Derivatives **3–27** and isoniazid (**1**) were evaluated for their cytotoxicity against 3T3 normal (mouse fibroblast) cell lines in which all derivatives were found to be inactive, except compound **18** and isoniazid (**1**) which showed IC_50_ values of 29.5 ± 1.9, and 28.5 ± 1.2 µM, respectively, in comparison to the standard cyclohexamide (IC_50_ = 0.8 ± 1.9 µM) (Table 1).

## Discussion

The present research study indicates that thiosemicarbazides **3–27 **possess promising urease inhibition activity, as well as significant anti-inflammatory activities. Compounds **5**, **9**, **12**, **16**, **17**, **22**, **23**, and **26 **were found to be the dual inhibitors of inflammation (ROS) and urease, few members of the current library have been reported as urease inhibitors by Ali. *et al*.^34^ but here we report the dual inhibitors of urease and inflammation along with its cytoxicity profile against 3T3 mouse fibroblast cell line. The limited SAR concludes that the compound **23 **with the cyclohexyl ring was found to be the most significant dual inhibitor of urease and inflammation (IC_50_ = 22.4 ± 1.83 µM and 12.3 ± 1.2 µg/mL, respectively). This showed comparable activity to the clinically used anti-inflammatory drug ibuprofen (IC_50_ = 11.2 ± 1.9 µg/mL), and urease inhibitor acetohyroxamic acid (IC_50_ = 20.3 ± 1.9 µg/mL). Compound **12**, is the pentafluoro substituted analogue. Compound **12 **was identified as the most potent inhibitor of urease (IC_50_ = 12.3 ± 1.04 µM) and significant inhibitor of inflammation (IC_50_ = 27.7 ± 2.4 µg/mL).

Whereas compounds **10**, **11**, and **13** were identified as potent inhibitors of urease enzyme, however, these compounds were found to be inactive in anti-inflammatory assay. The SAR clearly shows that change in the substituents and its positions or changing the aryl ring by the alkyl ring greatly affects the biological activity of the synthesized compounds.

In conclusion, the compounds reported here may serve as the starting points for the designing and development of new and powerful dual inhibitors of inflammation and infections caused by ureolytic bacteria.

### Experimental

The experimental section describes various methods and technical aspects of the current study including synthesis, purification, characterization of the synthesized analogues through different spectroscopic techniques and evaluation of the biological activities, such as inhibition of urease enzyme and inflammation.

### Chemicals

Isoniazid (**1**) was purchased from Sigma Aldrich, India; 2-bromophenyl isothiocyanate, 3-bromophenyl isothiocyanate, 4-fluorophenyl isothiocyanate, and 3-nitrophenyl isothiocyanate from Aldrich, Poland. Cyclopentylisothiocyanate, cyclohexylisothiocyanate, 2,5-dichlorophenyl isothiocyanate, 3-fluorophenyl isothiocyanate, hexyl isothiocyanate, 3-iodophenyl isothiocyanate, and 4-methoxy phenyl isothiocyanate from Sigma Aldrich, USA; 2,4,5-trichloro phenyl isothiocyanate from Aldrich, USA. 4-Chlorophenylisothiocyanate, 2,4-difluorophenyl isothiocyanate, and 2,6-dichlorophenyl isothiocyanate, 3,4-dichlorophenyl isothiocyanate were obtained from Alfa Aesar, USA. 2-Fluorophenyl isothiocyanate from Sigma Aldrich Chemie, Germany; 2-chloro-5-trifluoromethyl)phenyl isothiocyanate, 4-chloro-3-(trifluoromethyl)phenyl isothiocyanate, 4-cyanophenyl isothiocyanate, and 4-(trifluoromethoxy)phenyl isothiocyanate, 1,3-phenylene diisothiocyanate were purchased from Oakwood Chemicals, USA, 4-Dimethylaminoazobenzene 4′-isothiocyanate, and pentafluorophenylisothiocyanate were bought from Fluka, Switzerland, phenyl isothiocyanate from Schuchardt, Hohenbrunn, Germany.

Precoated silica gel plates (ALUGRAM, SIL G/UV254) were used for thin layer chromatography (TLC). TLC chromatograms were viewed under the ultraviolet light of 254  and 365 nm. Electron impact mass spectroscopy (EI-MS) and Fast atom bombardment direct probe mass spectra (FAB-MS) were obtained through JEOLJMS-600H mass spectrometer (Japan). ^1^H and ^13^C-NMR spectra were performed on a 300, 400, and 100 MHz Bruker Avance spectrometers (Switzerland). Buchi M-560 apparatus was used for recording the melting point (Japan). I. R. Spectrophotometry of the compounds was performed on FTIR-8900 (Shimadzu, Japan) through KBr disc.

### General Procedure of the Synthesis of Compounds 3–27

Isoniazid (**1**) (2 mmol) was refluxed with methanol (10 mL) for 15 minutes. Corresponding isothiocyanate (2 mmol) was added, and kept on stirring for 8–10 hours at room temperature (25 °C). Reaction progress was examined by TLC (7.9: 2: 0.1) (ethyl acetate, methanol and acetic acid) analysis. Disappearance of starting material from reaction mixture indicated the completion of reaction. The reaction mixture was concentrated under reduced pressure, solid product thus obtained was purified through solvent-solvent extraction with the help of hexane and ethyl acetate, and then recrystallized from methanol. Among these compounds, **9**, **10**, **12**, **21**, and **26** were identified as new derivatives. The known compounds were found to be spectroscopically similar to that reported in the literature.

#### *N*-(2-Fluorophenyl)-2-isonicotinoylhydrazinecarbothioamide (**3**)

**R**_**f**_ = 0.7, **Mp:** 241–242 °C; **IR** (**KBr**, **cm**^**–1**^): 3285, 3128 (N–H), 1679 (C=O), 1596, 1545, 1509 (C=C), 1226 (C=S), 1149 (C– F); ^**1**^**H-NMR**(**300** **MHz**, **DMSO-*d***_***6***_): *δ*_H_ 10.91 (s, 1 H, NH), 9.96 (s, 1 H, NH), 9.67 (s, 1 H, NH), 8.75 (d, *J*_3″,2″_ = *J*_5″,6″_ = 6.0 Hz, 2 H, H-3″, H-5″), 7.83 (d, *J*_2″,3″_ = *J*_6″,5″_ = 5.1 Hz, 2 H, H-2″, H-6″), 7.32–7.14 (m, 4 H, H-3, H-4, H-5, H-6); ^**13**^**C-NMR** (**100** **MHz**, **DMSO-*d***_***6***_): δ182.6 (C=S), 165.0 (C=O), 159.0 (d, *J*_*CF*_ = 248.8 Hz, C-2), 140.0 (C-1″), 150.1 (C-3″,C-5″), 130.6 (C-5), 128.0 (C-4), 124.0 (C-6), 121.6 (C2″,C-6″), 116.1 (d, *J* _CF_= 19.9 Hz, C-1), 115.6 (d, *J*_*CF*_ = 19.8 Hz,C-3); **Positive FAB-MS** ***m/z*** (**rel**. **int**. **%**): 291.1 [M + H]^+^ (56), 182.1 (100), 185.1 (27), 171.0 (16); **HRFAB-MS** (+**ve mode**) Calcd. for [(C_13_H_11_N_4_O_1_F_1_S_1_) + H]^+^: (*m/z* = 291.0716) Found 291.0712.

#### *N*-(2, 3-Dichlorophenyl)-2-isonicotinoylhydrazinecarbothioamide (**4**)

**R**_**f**_ = 0.76, **Mp**: 198–199 °C; **IR** (**KBr**, **cm**^**–1**^): 3289, 3137 (N–H), 1679 (C=O), 1546, 1512 (C=C), 1249 (C=S), 1058 (C–Cl). ^**1**^**H-NMR** (**600** **MHz**, **DMSO-*d***_***6***_): *δ*_H_ 10.93 (s, 1 H, NH), 10.02 (s, 1 H, NH), 9.82 (s, 1 H, NH), 8.76 (d, *J*_3″,2″_ = *J*_5″,6″_ = 6.0 Hz, 2 H, H-3″, H-5″), 7.83 (distorted singlet, 2 H, H-2″, H-6″), 7.56 (d, *J*_4,5_ = 7.8 Hz, 1 H, H-4), 7.36 (t, *J*_*5(4*,*6)*_ = 7.8 Hz, 1 H, H-5), 7.32 (distorted singlet, 1 H, H-6); ^**13**^**C-NMR** (**100** **MHz**, **DMSO-*d***_***6***_): δ182.4 (C=S), 165.0(C=O), 150.6(C-3″,C-5″),139.9 (C-1″), 139.3(C-1), 132.1 (C-2), 131.0 (C-3), 129.9 (C-6), 128.6 (C-4), 127.5 (C-5), 121.7 (C-2″,C-6″); **Positive FAB-MS** ***m/z*** (**rel**. **int**. **%**): 342.0 [M + 2]^+^ (3), 341.0 [M + H]^+^ (6), 185.1 (100), 277.1 (16), 219.1 (11); **HRFAB-MS** (+**ve mode**) Calcd. For [(C_13_H_10_N_4_O_1_Cl_2_S_1_) + H]^+^: (*m/z* = 341.0031) Found 341.0017.

#### *N*-(2, 5-Dichlorophenyl)-2-isonicotinoylhydrazinecarbothioamide (**5**)

**R**_**f**_ = 0.73, **Mp:** 172–173 °C; **IR** (**KBr**, **cm**^**–1**^): 3285, 3130 (N–H), 1679 (C=O), 1546, 1512 (C=C), 1252 (C=S), 1092 (C–Cl), ^**1**^**H-NMR** (**400** **MHz**, **DMSO-*d***_***6***_): *δ*_H_ 10.93 (s, 1 H, NH), 10.07 (s, 1 H, NH), 9.76 (s,1 H, NH), 8.76 (d, *J*_3″,2″_ = *J*_5″,6″_ = 6.0 Hz, 2 H, H-3″, H-5″), 7.84(d, *J*_2″,3″_ = *J*_6″,5″_ = 5.0 Hz, 2 H, H-2″, H-6″), 7.54 (d, *J*_5,4_ = 8.4 Hz, 1 H, H-6), 7.38 (distorted t, 2 H, H-3, H-4); **NegativeFAB-MS** ***m/z*** (**rel**. **int**. **%**): 341.1[M + 2]^+^ (3), 339.1[M-H]^**+**^ (6), 275.2 (22) 183.0 (100), 164.0 (10), 150.9 (11); **HRFAB-MS** (+**ve mode**) Calcd. for [(C_13_H_10_N_4_O_1_Cl_2_S_1_) + H]^+^: (*m/z* = 341.0031) Found 341.0010.

#### *N*-(2, 6-Dichlorophenyl)-2-isonicotinoylhydrazinecarbothioamide (**6**)

**R**_**f**_ = 0.73, **Mp:** 127–128 °C; **IR** (**KBr**, **cm**^**–1**^)**:** 3287, 3164 (N–H), 1681 (C=O), 1513 (C=C), 1244 (C=S), 1064 (C–Cl). ^**1**^**H-NMR** (**300** **MHz**, **DMSO-*d***_***6***_)**:** *δ*_H_ 10.91 (s, 1 H, NH), 10.0 (s, 1 H, NH), 9.75 (s, 1 H, NH), 8.76 (d, *J*_3″,2″_ = *J*_5″,6″_ = 5.1 Hz, 2 H, H-3″, H-5″), 7.83 (d, *J*_2″,3″_ = *J*_6″,5″_ = 4.5 Hz, 2 H, H-2″, H-6″), 7.51 (d, *J*_3,4_ = *J*_5,4_ = 8.1 Hz, 2 H, H-3, H-5), 7.39 (distorted t, 1 H, H-4); **Negative FAB-MS** ***m/z*** (**rel**. **int**. **%**): 341.0 [M + 2]^+^ (3), 339.2 [M-H]^**+**^ (8), 275.2 (18), 268.2 (14), 183.1(100), 176.1(44); **HRFAB-MS** (+**ve mode**) Calcd. for [(C_13_H_10_N_4_O_1_Cl_2_S_1_) + H]^+^: (*m/z* = 341.0031) Found 341.0010.

#### *N*-(2, 6-Difluorophenyl)-2-isonicotinoylhydrazinecarbothioamide (**7**)

**R**_**f**_ = 0.7, **Mp:** 248–249 °C; **IR** (**KBr**, **cm**^**–1**^): 3292, 3131 (N–H), 1681 (C=O), 1595, 1546 (C = C), 1246 (C=), 1151 (C–F), ^**1**^**H-NMR** (**400** **MHz**, **DMSO-*d***_***6***_): *δ*_H_ 10.95 (s, 1 H, NH), 10.11 (s, 1 H, NH), 9.47 (s, 1 H, NH), 8.75 (d, *J*_3″,2″_ = *J*_5″,6″_ = 4.8 Hz, 2 H, H-3″, H-5″), 7.84 (distorted singlet, 2 H, H-2″, H-6″), 7.34 (m, 1 H, H-4), 7.12 (t, *J*_3,2/3,4_ = *J*_5,4/5,6_ 8.0 Hz, 2 H, H-3, H-5); ^**13**^**C-NMR** (**125** **MHz**, **DMSO-d**_**6**_)**:** δ182.6 (C=S), 164.4 (C=O), 159.8 (d, *J*_*CF*_ = 247.8 Hz, C-2), 159.7 (d, *J*_*CF*_ = 247 Hz, C-6), 150.1 (C-3″,C-5″), 139.3 (C-1″), 128.8 (t, *J*_*CF*_ = 10 Hz, C-4), 121.7 (C2″,C-6″), 116.6 (t, *J*_*CF*_ = 15.8 Hz, C-1), 111.7 (d, *J*_*CF*_ = 21.8 Hz, C-3, C-5); **Positive FAB-MS** ***m/z*** (**rel**. **int**. **%**): 309.0 [M + H]^+^ (17), 277.0 (27), 219 (19), 185.0 (100), 171.1 (96), 157.0 (45); **HRFAB-MS** (+**ve mode**) Calcd. for [(C_13_H_10_F_2_N_4_OS) + H]^+^: (*m/z* = 309.0622) Found 309.0610.

#### *N*-(2-Chloro-5-(trifluoromethyl)phenyl)-2-isonicotinoylhydrazinecarbothioamide (**8**)

**R**_**f**_ = 0.8, **Mp:** 183–184 °C; **IR** (**KBr**, **cm**^**–1**^): 3304, 3134 (N–H), 1679 (C=O), 1548, 1513 (C=C), 1253 (C=S), 1080 (C–Cl). ^**1**^**H-NMR **(**400** **MHz**, **DMSO-*d***_***6***_): *δ*_H_ 10.96 (s, 1 H, NH), 10.14 (s, 1 H, NH), 9.83 (s, 1 H, NH), 8.76 (d, *J*_3″,2″_ = *J*_5″,6″_ = 5.6 Hz, 2 H, H-3″, H-5″), 7.84 (br s, 2 H, H-2″, H-6″), 7.76 (d, *J*_6,4_ = 8.8 Hz, 1 H, H-6), 7.67 (br s, 2 H, H-3, H-4); **Positive FAB-MS** ***m/z*** (**rel**. **int**. **%**): 376.1[M + 2]^+^ (13),375.1 [M + H]^+^ (19), 277.1 (8), 255.0 (16), 185.2 (83), 157.1 (100); **HRFAB-MS** (+**ve mode**) Calcd. for [(C_14_H_10_N_4_O_1_Cl_1_F_3_S_1_) + H]^+^: (*m/z* = 375.0294) Found 375.0307.

#### *N*-(4-Chloro-3-(trifluoromethyl)phenyl)-2 isonicotinoylhydrazinecarbothioamide (**9**)

**R**_**f**_ = 0.76, **Mp:** 231–232 °C;**IR** (**KBr**, **cm**^**–1**^)**:** 3279, 3125 (N–H), 1675 (C=O), 1551, 1516 (C=C), 1256 (C=S), 1065 (C–Cl). ^**1**^**H-NMR **(**400** **MHz**, **DMSO-d**_**6**_): δ_H_ 10.92 (s, 1 H, NH), 10.14 (s, 1 H, NH), 10.02 (s, 1 H, NH), 8.77 (d, *J*_3″,2″_ = *J*_5″,6″_ = 5.6 Hz, 2 H, H-3″, H-5″), 7.99 (br s, 1 H, H-2), 7.85 (br.s, 3 H, H-6″, H-2″, H-5), 7.67 (d, *J*_3,2_ = 8.4 Hz, 1 H, H-6), ^**13**^**C-NMR** (**125** **MHz**, **DMSO-d**_**6**_): δ180.8 (C=S), 164.4 (C=O), 150.2 (C-3″,C-5″), 139.2 (C-1″), 138.6 (C-1), 131.2 (C-6), 130.5 (C-5), 125.9 (m, CF_3_), 124.2 (C-2), 123.7 (C-3), 121.6 (C2″, C-6″), 119.4 (C-4); **Positive FAB-MS m/z** (**rel**. **int**. **%**): 377.2 [M + 2]^+^ (22), 375.1 [M + H]^+^ (32), 277.0 (27), 219 (19), 185.0 (100), 171.1 (96), 157.0 (45); **HRFAB-MS** (+**ve mode**) Calcd. for [(C_14_H_10_N_4_O_1_Cl_1_F_3_S_1_) + H]: (m/z = 375.0294) Found 375.0295.

#### *N*-(4-Cyanophenyl)-2-isonicotinoylhydrazinecarbothioamide (**10**)

**R**_**f**_ = 0.66, **Mp**: 162–163 °C; **IR** (**KBr**, **cm**^**–1**^): 3314, 3216 (N–H), 2224 (C≡N), 1675 (C=O), 1543, 1510 (C=C), 1217 (C=S). ^**1**^**H-NMR **(**400** **MHz**, **CD**_**3**_**OD**): *δ*_H_ 8.72 (d, *J*_3″,2″_ = *J*_5″,6″_ = 6.0 Hz, 2 H, H-3″, H-5″), 7.88 (d, *J*_2″,3″_ = *J*_6″,5″_ = 6.0 Hz, 2 H, H-2″, H-6″), 7.82 (d, *J*_2,3_ = *J*_6,5_ = 8.4 Hz, 2 H, H-2, H-6), 7.67 (d, *J*_3,2_ = *J*_5,6_ = 8.4 Hz, 2 H, H-3, H-5), ^**13**^**C-NMR** (**150** **MHz**, **DMSO-d**_**6**_): δ180.6 (C=S), 164.4 (C=O), 150.2 (C-3″,C-5″), 143.6 (C-1″), 139.3 (C-1), 132.5 (C-3), 132.1 (C-5), 125.4 (C-2), 122.6 (C-6), 118.9 (C-4), 106.8 (CN); **Positive FAB-MS** ***m/z*** (**rel**. **int**. **%**): 297.1 [M + H]^+^ (4), 277.1 (15), 219.2 (8), 185.0 (100), 171.0 (23), 157.0 (8); **HRFAB-MS** (+**ve mode**) Calcd. for [(C_14_H_11_N_5_O_1_S_1_) + H]^+^: (*m/z* = 298.0763) Found 298.0788.

#### 2-Isonicotinoyl-*N*-(4-(trifluoromethoxy) phenyl) hydrazine carbothioamide (**11**)

**R**_**f**_ = 0.76, **Mp**: 192–193 °C; **IR** (**KBr**, **cm**^**–1**^): 3271, 3125 (N–H), 1678 (C=O), 1513 (C=C), 1267 (C=S). ^**1**^**H-NMR **(**400** **MHz**, **DMSO-*d***_***6***_): *δ*_H_ 10.86 (s, 1 H, NH), 9.92 (s, 2 H, NH), 8.76 (d, *J*_3″,2″_ = *J*_5″,6″_ = 5.6 Hz, 2 H, H-3″, H-5″), 7.83 (d, *J*_2″,3″_ = *J*_6″,5″_ = 5.6 Hz, 2 H, H-2″, H-6″), 7.54 (br s, 2 H, H-2, H-6), 7.32 (d, *J*_3,2_ = *J*_5,6_ = 8.4 Hz, 2 H, H-3, H-5), **Positive FAB-MS** ***m/z*** (**rel**. **int**. **%**): 357.1 [M + H]^+^ (38), 329 (8), 277.1 (15), 236.9 (58), 185.0 (100), 171.0 (19), 157.0 (8); **HRFAB-MS** (+**ve mode**) Calcd. for [(C_14_H_11_N_4_O_2_F_3_S_1_) + H]^+^: (*m/z* = 357.0633) Found 357.0619.

#### 2-Isonicotinoyl-*N*-(perfluorophenyl) hydrazinecarbothioamide (**12**)

**R**_**f**_ = 0.7, **Mp:** 255–256 °C; **IR** (**KBr**, **cm**^**–1**^)**:** 3303, 3150 (N–H), 1682 (C=O), 1528, 1505 (C=C), 1249 (C=S), 1153 (C–F). ^**1**^**H-NMR **(**300** **MHz**, **DMSO-d**_**6**_)**:** δ_H_ 11.06 (s, 1 H, NH), 10.44 (s, 1 H, NH), 9.72 (s, 1 H, NH), 8.77 (d, J_3″,2″_ = J_5″,6″_ = 5.1 Hz, 2 H, H-3″, H-5″), 7.84 (distorted singlet, 2 H, H-2″, H-6″), ^**13**^**C-NMR** (**125** **MHz**, **DMSO-d**_**6**_)**:** δ182.6 (C=S), 164.4 (C=O), 150.2 (C-3″,C-5″), 144.7 (d, J = 206 Hz, C-2, C-6), 140.5 (t, J = 11.4 Hz, C-1), 139.5 (C-1″), 138.9 (m, C-4), 137.8 (t, J = 11.9 Hz, C-3), 136.2 (t, J = 14.3 Hz, C-3), **Positive FAB-MS m/z** (**rel**. **int**. **%**)**:** 363.0 [M + H]^+^ (15), 335.0 (4), 277.0 (17), 243.0 (7), 185.2 (100); **HRFAB-MS** (+**ve mode**) Calcd. for [(C_13_H_7_N_4_O_1_F_5_S_1_) + H]^+^ (*m/z* = 363.0339) Found 363.0323.

#### *N*-Cyclopentyl-2-isonicotinoylhydrazinecarbothioamide (**13**)

**R**_**f**_ = 0.7, **Mp**: 223–224 °C; **IR** (**KBr**, **cm**^**–1**^): 3268, 3142 (N–H), 1679 (C=O), 1552, 1527 (C=C), 1451 (CH_2_ bending), 1263 (C=S stretching). ^**1**^**H-NMR **(**300** **MHz**, **DMSO-*d***_***6***_): *δ*_H_ 10.54 (s, 1 H, NH), 9.26 (s, 1 H, NH), 8.74 (d, *J*_3″,2″_ = *J*_5″,6″_ = 5.7 Hz, 2 H, H-3″, H-5″), 7.80 (d, *J*_2″,3″_ = *J*_6″,5″_ = *J*_3′,4′_ = 5.7 Hz, 3 H, H-2″, H-6″, NH), 4.11 (br s, 1 H, H-1), 1.77–1.55 (m, 4 H, H-2a, H-2b, H-5a, H-5b), 1.27–1.03 (m, 4 H, H-3a, H-3b, H-4a, H-4b), **Negative FAB-MS** ***m/z*** (**rel**. **int**. **%**): 265.2 [M-H]^+^ (4), 219.9 (17), 207.0 (18), 183.0 (100), 164.0 (4); **HRFAB-MS** (+**ve mode**) Calcd. for. [C_12_H_16_N_4_OS + H]^+^: (*m/z* = 265.1045) Found 265.1043.

#### *N*-(2-Bromophenyl)-2-isonicotinoylhydrazinecarbothioamide (**14**)

**R**_**f**_ = 0.73, **Mp:** 156–157 °C;**IR** (**KBr**, **cm**^**–1**^)**:** 3275, 3131 (N–H), 1679 (C=O), 1543, 1474 (C=C), 1251 (C=S), 1059 (C–Br). ^**1**^**H-NMR **(**300** **MHz**, **DMSO-d**_**6**_)**:** δ_H_ 10.89 (s, 1 H, NH), 9.90 (s, 1 H, NH), 9.70 (s, 1 H, NH), 8.75 (d,J_3″,2″_ = J_5″,6″_ = 6.3 Hz, 2 H, H-3″, H-5″), 7.84 (d,J_2″,3″_ = J_6″,5″_ = 5.1 Hz, 2 H, H-2″, H-6″), 7.65 (d, J_3,4_ = 7.8 Hz, 1 H, H-3), 7.38 (br s, 2 H, H-4, H-6), 7.19 (m, 1 H, H-5), **Positive FAB-MS m/z** (**rel**. **int**. **%**)**:** 351.9 [(M + 2)]^+^ (7), 350.9 [M + H]^+^ (8), 277.1 (15), 185.2 (100), 171.2 (17), **HRFAB-MS** (+**ve mode**) Calcd. for [(C_13_H_11_N_4_O_1_Br_1_S_1)_ + H]^+^ (*m/z *= 351.9993) Found 351.9999.

#### *N*-(2, 4-Difluorophenyl)-2-isonicotinoylhydrazinecarbothioamide (**15**)

**R**_**f**_ = 0.43, **Mp** 191–192 °C; **IR** (**KBr**, **cm**^**–1**^): 3300, 3141 (N–H), 1682 (C=O), 1550, 1514 (C=C), 1251 (C=S stretching), 1142 (C–F), ^**1**^**H-NMR **(**400** **MHz**, **DMSO-*d***_***6***_): *δ*_H_ 10.91 (s, 1 H, NH), 10.0 (s, 1 H, NH), 9.61 (s,1 H, NH), 8.75 (d, *J*_3″,2″_ = *J*_5″,6″_ = 4.8 Hz, 2 H, H-3″, H-5″), 7.83 (s, 2 H, H-2″, H-6″), 7.26 (t, *J*_5,6_ = *J*_6,5_ = 8.4 Hz, 2 H, H-6, H-5), 7.00 (t, *J*_*3(*4,2)_ = 8.2 Hz, 1 H, H-3); **Electron Ionization Mass spectrometry** (**direct probe**) ***m/z*** 308.1 [M]^+^ (2.2), 274.1 (11.5), 171.0 (100), 106.0 (45.6), 78.0 (43.1); **HREI-MS** Calcd. For [C_13_H_9_N_4_O_1_F_2_S_1_]: (*m/z* = 308.0543) Found 308.0548.

#### *N*-(3-Fluorophenyl)-2-isonicotinoylhydrazinecarbothioamide (**16**)

**R**_**f**_ = 0.73, **Mp** 174–175 °C; **IR** (**KBr**, **cm**^**–1**^): 3266, 3112 (N–H), 1674 (C=O), 1601, 1515 (C=C), 1238 (C=S), 1142 (C–F); ^**1**^**H-NMR **(**400** **MHz**, **DMSO-*d***_***6***_): *δ*_H_ 10.86 (s, 1 H, NH), 9.93 (s, 2 H, NH), 8.76 (d, *J*_3″,2″_ = *J*_5″,6″_ = 6 Hz, 2 H, H-3″, H-5″), 7.83 (d, *J*_2″,3″_ = *J*_6″,5″_ = 5.6 Hz, 2 H, H-2″, H-6″), 7.45 (br.s, 1 H, H-2), 7.35 (q, *J*_5(4,6)_ = 8.0 Hz, 1 H, H-5), 7.27 (d, *J*_6,5_ = 8.0 Hz, 1 H, H-6), 6.98 (t, *J*_4(5,6)_ = 7.6 Hz, 1 H, H-4); **Positive FAB-MS** ***m/z*** (**rel**. **int**. **%**): 291.0 [M + H]^+^ (5), 185.1 (100), 277.1 (15), 219.1 (12); **HRFAB-MS** (+**ve mode**) Calcd. for [(C_13_H_11_FN_4_OS) + H]^+^ (*m/z* = 291.0716) Found 291.0721.

#### *N*-(3-Iodophenyl)-2-isonicotinoylhydrazinecarbothioamide (**17**)

**R**_**f**_ = 0.73, **Mp** 192–193 °C; **IR** (**KBr**, **cm**^**–1**^): 3266, 3112.3 (N–H), 1674.5 (C=O), 1601, 1515 (C=C), 1237.9 (C=S), 851.8 (C–I). ^**1**^**H-NMR**(**400** **MHz**, **DMSO-*d***_***6***_): *δ*_H_ 10.85 (s, 1 H, NH), 9.93 (s, 1 H, NH), 9.84 (s, 1 H, NH), 8.76 (d, *J*_3″,2″_ = *J*_5″,6″_ = 5.6 Hz, 2 H, H-3″, H-5″), 7.83 (d, *J*_2″,3″_ = *J*_6″,5″_ = *J*_4,5_ = 5.2 Hz, 3 H, H-2″, H-6″, H-4), 7.51 (t, *J*_5,4/5,6)_ = *J*_2,4/2,6)_ = 6.8 Hz, 2 H, H-2, H-5), 7.12 (t, *J*_6,5_ = 8.0 Hz, 1 H, H-6); **Positive FAB-MS** ***m/z*** (**rel**. **int**. **%**): 399.0 [M + H]^+^ (16), 183.2 (100), 275.2 (19), 243.0 (7); **HRFAB-MS** (+**ve mode**) Calcd. for [(C_13_H_12_N_4_O_1_I_1_S_1)_ + H]^+^ (*m/z* = 398.9776) Found 398.9759.

#### *N*-(4-Chlorophenyl)-2-isonicotinoylhydrazine carbothioamide (**18**)

**R**_**f**_ = 0.73, **Mp** 150–151 °C; **IR** (**KBr**, **cm**^**–1**^): 3251.8, 3130.7 (N–H), 1676 (C=O), 1596, 1545 (C=C), 1256 (C=S), 1093.7 (C–Cl). ^**1**^**H-NMR **(**300** **MHz**, **DMSO-*d***_***6***_): *δ*_H_ 10.86 (s, 1 H, NH), 9.90 (s, 2 H, NH), 8.76 (dd, *J*_3″,2″_ = *J*_5″,6″_ = *J*_1_ = 1.8, *J*_2_ = 1.5 Hz, 2 H, H-3″,H-5″), 7.83 (dd, *J*_2″,3″_ = *J*_6″,5″_ = *J*_1_ = 1.5 Hz, *J*_2_ = 1.2 Hz, 2 H, H-2″, H-6″), 7.70 (s, 1 H, H-2), 7.47 (appear d, *J*_3,2_ = *J*_5,6_ = 7.3 Hz, 2 H, H-3, H-5), 7.37 (m, 2 H, H-2, H-6); **Positive FAB-MS** ***m/z*** (**rel**. **int**. **%**): 307.0 [M + 2]^+^ (5), 305 [M^+^] (15), 185.1(100), 277.1 (14), 219.2 (10); **HRFAB-MS** (+**ve mode**) Calcd. for [(C_13_H_11_N_4_O_1_Cl_1_S_1_) + H]^+^ (*m/z* = 307.0420) Found 307.0450.

#### *N*-(4-Fluorophenyl)-2-isonicotinoylhydrazinecarbothioamide (**19**)

**R**_**f**_ = 0.73, **Mp** 191–192 °C; **IR** (**KBr**, **cm**^**–1**^): 3201, 3155 (N–H), 1684 (C=O), 1544, 1512 (C=C), 1263 (C=S), 1223 (C–F). ^**1**^**H-NMR **(**400** **MHz**, **DMSO-*d***_***6***_): *δ*_H_ 10.84 (s, 1 H, NH), 9.82 (s, 2 H, NH), 8.76 (d, *J*_3″,2″_ = *J*_5″,6″_ = 6 Hz, 2 H, H-3″, H5″),7.83 (d, *J*_2″,3″_ = *J*_6″,5″_ = 5.6 Hz, 2 H, H-2″, H-6″), 7.39 (br.s, 2 H, H-2, H-6), 7.16 (t, *J*_3,2/3,4_ = *J*_5,6/5,4_ = 8.8 Hz, 2 H, H-3, H-5), **Positive FAB-MS** ***m/z*** (**rel**. **int**. **%**): 291.0 [M + H]^+^ (6), 185.1 (100), 277.1 (14), 219.2 (10); **HRFAB-MS** (+**ve mode**) Calcd. for [(C_13_H_11_N_4_O_1_F_1_S_1_) + H]^+^ (*m/z* = 291.0716) Found 291.0730.

#### 2-Isonicotinoyl-*N*-(4-methoxyphenyl) hydrazinecarbothioamide (**20**)

**R**_**f**_ = 0.66, **Mp**: 171–172 °C; **IR** (**KBr**, **cm**^**–1**^): 3255, 3124 (N–H), 1675 (C=O), 1545, 1514 (C=C), 1253 (C=S). ^**1**^**H-NMR** (**400** **MHz**, **DMSO-*d***_***6***_)**: ***δ*_H_10.79 (s,1 H, NH), 9.72 (s,1 H, NH), 9.67 (s,1 H, NH), 8.75 (d, *J*_3″,2″_ = *J*_5″,6″_ = 5.6 Hz, 2 H, H-3″, H-5″), 7.83 (d, *J*_2″,3″_ = *J*_6″,5″_ = 5.6 Hz, 2 H, H-2″, H-6″), 7.25 (d, *J*_3,2_ = *J*_5,6_ = 7.6 Hz, 2 H, H-2, H-6), 6.88 (d, *J*_2,3_ = *J*_6,5_ = 9.2 Hz, 2 H, H-3, H-5), 3.11 (s, 3 H, H-7); **Positive FAB-MS** ***m/z*** (**rel**. **int**. **%**): 303.0 [M + H]^+^ (4), 185.1(100), 277.1(12), 219.1(8); **HRFAB-MS** (+**ve mode**) Calcd. for [(C_14_H_14_N_4_O_2_S_1_) + H]^+^ (*m/z* = 303.0916) Found 303.0924.

#### Isonicotinoyl-*N*-(2, 4, 5-trichlorophenyl) hydrazinecarbothioamide (**21**)

**R**_**f**_ = 0.73, **Mp**: 241–242 °C; **IR** (**KBr**, **cm**^**–1**^): 3282, 3127 (N–H), 1678 (C=O), 1561, 1510, (C=C), 1259 (C=S), 1079 (C–Cl). ^**1**^**H-NMR **(**300** **MHz**, **DMSO-*d***_***6***_): 10.94 (s, 1 H, NH), 10.14 (s, 1 H, NH), 9.79 (s,1 H, NH), 8.76 (d, *J*_*3*″,*2*″_ = *J*_*5*″,*6*″_ = 6 Hz, 2 H, H-3″, H-5″), 7.92 (s, 1 H, H-2), 7.83 (br s, 2 H, H-2″, H-6″), 7.61 (br s, 1 H, H-5); ^**13**^**C-NMR** (**125** **MHz**, **DMSO-d**_**6**_): δ181.9 (C=S), 164.5 (C=O), 150.2 (C-3″,C-5″), 139.3 (C-1″), 137.0(C-1), 132.0 (C-2), 131.4 (C-4),130.4 (C-5), 129.9 (C-6), 129.2 (C-3)121.7 (C2″,C-6″); **Positive FAB-MS** ***m/z*** (**rel**. **int**. **%**): 377.0 [M + 2]^+^ (17),375.2 [M + H]^+^, (19), 275.3 (25), 183.1 (100), 136.1 (14), 127.1 (10). **HRFAB-MS** (+**ve mode**) Calcd. for [(C_13_H_9_N_4_O_1_Cl_3_S_1_) + H]^+^ (*m/z* = 374.9641) Found 374.9633.

#### *N*-(3-Bromophenyl)-2-isonicotinoylhydrazinecarbothioamide (**22**)

**R**_**f**_ = 0.73, **Mp**: 192–193 °C; **IR** (**KBr**, **cm**^**–1**^): 3301, 3143 (N–H), 1683 (C=O), 1589, 1548 (C=C), 1252 (C=S), 1066 (C–Br). ^**1**^**H-NMR **(**400** **MHz**, **DMSO-d**_**6**_): δ_H_ 10.86 (s, 1 H, NH), 9.95 (s, 1 H, NH), 9.88 (s, 1 H, NH), 8.76 (d, J_3″,2″_ = J_5″,6″_ = 5.6 Hz, 2 H, H-3″, H-5″), 7.83 (d,J_2″,3″_ = J_6″,5″_ = 4.8 Hz, 2 H, H-2″, H-6″), 7.70 (s, 1 H, H-2), 7.49 (d, J_4,5_ = 7.6 Hz, 1 H, H-4),7.32(d,J_6,5_ = 7.6 Hz, 1 H, H-6),7.28 (t, J_5,4/5,6_ = 8.0 Hz, 1 H, H5); **Positive FAB-MS m/z** (**rel**. **int**. **%**): 353.0[(M + 2]^+^ (14), 351.0 [M + H]^+^ (15), 185.0 (100), 277.2 (14), 219.1 (4); **HRFAB-MS** (+**ve mode**) Calcd. for [(C_13_H_11_N_4_O_1_S_1_Br_1_) + H]^+^ (*m/z* = 350.9915) Found 350.9895.

#### *N*-Cyclohexyl-2-isonicotinoylhydrazinecarbothioamide (**23**)

**R**_**f**_ = 0.71, **Mp:** 212–213 °C; **IR** (**KBr**, **cm**^**–1**^): 3268, 3141 (N–H), 1680 (C=O), 1551, 1530 (C=C), 1452 (CH_2_ bending), 1266 (C=S). ^**1**^**H-NMR **(**300** **MHz**, **DMSO-*d***_***6***_): *δ*_H_ 10.56 (s, 1 H, NH), 9.28 (s, 1 H,NH), 8.76 (dd, *J*_3″, 2″_ = *J*_5″, 6′ ‘_ = 1.5 Hz, 2 H, H-3_″_, H-5″), 7.81 (d, *J*_2″−3″_ = *J*_6″−5″_ = 6.0 Hz, 3 H, H-2″, H-6″, NH), 4.11 (m, 1 H, H-1), 1.78 (m, 4 H, H-2a, H-2b, H-6a, H-6b), 1.60 (d, *J*_5a,6_ = 12.3 Hz, 1 H, H-5a), 1.28 (m, 4 H, H-3a, H-3b, H-4a, H-4b),1.07 (m, 1 H, H-5b); **Positive FAB-MS** ***m/z*** (**rel**. **int**. **%**)**:** 279.1 [M + H]^+^ (25), 185.1 (100), 219.2 (6), 251.2 (4); **HRFAB-MS** (+**ve mode**) Calcd. for [(C_13_H_18_N_4_O_1_S_1_) + H]^+^ (*m/z* = 279.1280) Found 279.1273.

#### 2-Isonicotinoyl-*N*-phenylhydrazinecarbothioamide (**24**)

**R**_**f**_ = 0.7, **Mp**: 188–189 °C; **IR** (**KBr**, **cm**^**–1**^): 3262, 3120 (N–H), 1675 (C=O), 1547, 1512 (C=C), 1254 (C=S), ^**1**^**H-NMR **(**300** **MHz**, **DMSO-*d***_***6***_)**:** *δ*_H_ 10.84 (s, 1 H, NH), 9.84 (s, 1 H, NH), 9.78 (s,1 H, NH), 8.76 (m, 2 H, H-3″, H-5″), 7.83 (d, *J*_2″,3″_ = *J*_6″,5″_ = 6.0 Hz, 2 H, H-2″, H-6″), 7.41 (d, *J*_2,3_ = *J*_6,5_ = 7.2 Hz, 2 H, H-2, H-6), 7.32 (t, *J*_3,2_ = *J*_4,5_ = *J*_4,6_ = 7.8 Hz, 2 H, H-3, H-5), 7.15 (t, *J*_4,3_ = *J*_4,6_ = 7.2 Hz, 1 H, H-4); **Positive FAB-MS** ***m/z*** (**rel**. **int**. **%**): 273.1 [M + H]^+^ (15), 185.1 (100), 219.1 (8), 263.1 (5), 191.1 (4); **HRFAB-MS** (+**ve mode**) Calcd. for [(C_13_H_12_N_4_O_1_S_1_) + H]^+^ (*m/z* = 273.0810) Found 273.0799.

#### *N*-Hexyl-2-isonicotinoylhydrazinecarbothioamide (**25**)

**R**_**f**_ = 0.46, **Mp** 215–216 °C; **IR** (**KBr**, **cm**^**–1**^): 3300.5, 3170.6 (N–H), 1677.4 (C=O), 1555, 1527 (C=C), 1245 (C=S), 753 (long chain band). ^**1**^**H-NMR **(**400** **MHz**, **DMSO-*d***_***6***_): *δ*_H_ 10.57 (s, 1 H, NH), 9.29 (s, 1 H, NH), 8.74 (d, *J*_3″,2″_ = *J*_5″,6″_ = 6.0 Hz 2 H, H-3″, H-5″), 8.12 (s,1 H, NH), 7.79 (d, *J*_2″,3″_ = *J*_6″,5″_ = 5.6 Hz, 2 H, H-2″, H-6″), 3.42 (m, 2 H, H-1a, H-1b), 1.46 (m, 2 H, H-2a, H-2b), 1.23 (m, 6 H, H-3a, H-3b, H-4a, H-4b, H5a, H-5b), 0.84 (m, 3 H, H-6a, H-6b, H-6c), **Negative FAB-MS** ***m/z*** (**rel**. **int**. **%**): 279 0 [M-H]^+^ (23), 275 (19), 255.1 (8), 183 (100), **HRFAB-MS** (**−ve mode**) Calcd. for [(C_13_H_20_N_4_O_1_S_1_) – H]: (*m/z* = 279.0811) Found 279.0810.

#### *N*-(3-Thiocyanophenyl)-2-isonicotinoylhydrazinecarbothioamide (**26**)

**R**_**f**_ = 0.39, **Mp**: 195–196 °C; **IR** (**KBr**, **cm**^**–1**^): 3283, 3139 (N–H), 2118 (N=C=S), 1678 (C=O), 1595, 1548 (C=C), 1241 (C=S). ^**1**^**H-NMR **(**300 MHz**, **DMSO-*d***_***6***_): *δ*_H_ 10.89 (s, 1 H, NH), 9.91 (s, 2 H, NH), 8.76 (m, 2 H, H-3″, H-5″), 7.83 (d, *J*_2″,3″_ = *J*_6″,5″_ = 5.7 Hz, 2 H, H-2″, H-6″), 7.56 (s, 1 H, H-2), 7.47 (d, *J*_6,5_ = 8.4 Hz, 1 H, H-6),7.39 (m, 1 H, H-5), 7.21 (d, *J*_4,5_ = 7.8 Hz, 1 H, H-4), ^**13**^**C-NMR** (**125 MHz**, **DMSO-*d***_***6***_): δ180.8 (C=S), 164.4 (C=O), 150.2 (C-3″,C-5″), 140.4 (C-1″), 139.5 (C-1), 139.4 (C-3), 134.1 (NCS), 129.4 (C-5), 125.2 (C-6), 122.8 (C-4), 122.4 (C-2) 121.6 (C2″, C-6″); **Negative FAB-MS** ***m/z*** (**rel**. **int**. **%**): 328.1 [M-H]^+^ (15), 275.1 (44), 183 (100), **HRFAB-MS** (**−ve mode**) Calcd. for [(C_14_H_11_N_5_OS_2_) – H]: (*m/z* = 328.0320) Found 328.0315.

#### 2-Isonicotinoyl-*N*-(3-nitrophenyl) hydrazinecarbothioamide (**27**)

**R**_**f**_ = 0.7, **Mp:** 159–160 °C; **IR** (**KBr**, **cm**^**–1**^): 3284, 3124 (N–H), 1676 (C=O), 1597, 1531 (C=C), 1477 (N=O), 1247 (C=S). ^**1**^**H-NMR **(**300 MHz**, **DMSO-*d***_***6***_): *δ*_H_ 10.94 (s, 1 H, NH), 10.15 (s, 2 H, NH), 8.78 (d, *J*_3″,2″_ = *J*_5″,6″_ = 6.0 Hz, 2 H, H-3″, H5″), 8.42 (bs, 1 H, H-2), 7.99 (t, *J*_4,5_ = *J*_6,5_ = 6.6 Hz, 2 H, H-4, H-6), 7.85 (d, *J*_2″,3″_ = *J*_6″,5″_ = 5.7 Hz, 2 H, H-2″, H-6″), 7.61 (t, *J*_5,4/5,6)_ = 8.1 Hz, 1 H, H-5), **Negative FAB-MS** ***m/z*** (**rel**. **int**. **%**): 316 [M-H]^+^ (13), 275 (23), 183 (100), **HRFAB-MS** (**−ve mode**) Calcd. for [(C_13_H_11_N_5_O_3_S) – H]: (*m/z* = 316.1281) Found 316.1280.

### Bioassays

#### Protocol for *In Vitro* Urease Inhibition Assay

The urease inhibition activity of compounds **3–27** was evaluated by using the method reported by Weatherburn. *et al*. (1967). Thiourea and acetohydraoxamic acid were used as standard compounds^42–44^. During the experiments all the compounds were evaluated at 0.5 mM each in triplicate. The compounds with >50% (greater than 50%) inhibition were further studied to determine their IC_50_ value in a separate experiment where, different concentration of the compound from 0.5–0.0078125 mM were tested in triplicates. On the other hand if compound showed <50% (less than 50%) inhibition at 0.5 mM then the compound was considered inactive.

#### Protocol for *In Vitro* Anti-Inflammatory Assay

Oxidative Burst Assay: Anti-inflammatory activity of the thiosemicarbazide derivatives **3–27**, and isoniazid (**1**) was evaluated by following the method reported in Helfand. *et al*. (1982)^45,46^. In this experiment all the compounds were evaluated at 25 µg/mL, each in triplicate. To determine the IC_50_ values, the compounds with >50% inhibition were further evaluated on three different concentrations (1, 10 and 100 µg/mL). While, the compound failed to inhibit the production of ROS from zymosan activated whole blood cells at highest used dose (100 µg/mL) was considered as inactive.

All studies on human blood cells was carried out after an approval from independent ethics committee (Prof. Dr. Ghazala H. Rizwani Chair, IEC, Prof. Qamar Amin, Prof, Dr. Muddasir Uddin, Dr. Shahnaz Ghazi, Dr. SamiuzZaman, Prof. Dr. Ahsana Dar Farooq, Dr. M. Raza Shah member IEC), International Center for Chemical and Biological Sciences, University of Karachi, No: ICCBS/IEC-008-BC-2015/Protocol/1.0. Informed consents were obtained from the volunteers before drawing the blood. All the experiments were performed in accordance with relevant guidelines and regulations.

#### Protocol for Cytotoxicity (MTT assay) Assay

3T3 Cytotoxicity Assay: Cytotoxicity of the thiosemicarbazide derivatives **3–27**, and isoniazid (**1**) was evaluated by the method reported by Pauwels. *et al*. (1988)^47,48^. In this experiment all the compounds were evaluated at 30 µM each in triplicate. If the compound showed >50% inhibition then for the determination of IC_50_ value different concentration of the compound from 30–0.9375 µM were tested. While if the compound showed <50% inhibition at 30 µM then the compound was considered to be inactive.

## Supplementary information

Supplementary Material

## Data Availability

All the supplementary mateial is available.
