# Supplementary material for: Studies on Isoniazid Derivatives through a Medicinal Chemistry Approach for the Identification of New Inhibitors of Urease and Inflammatory Markers
Source: Sci Rep. 2019 May 1;9:6738. doi: 10.1038/s41598-019-43082-0 (PMC6494997; doi:10.1038/s41598-019-43082-0)
Supplement: Supplementary file 1 — Supplementary Material [file 41598_2019_43082_MOESM1_ESM.pdf]

Studies on Isoniazid Derivatives through a Medicinal Chemistry Approach for the Identification of New Inhibitors of Urease and Inflammatory Markers

Fazila Rizvi[1], Majid Khan[1], Almas Jabeen[2], Hina Siddiqui[1*], and M. Iqbal Choudhary[1, 2,3*]

[1]H.E.J. Research Institute of Chemistry, International Center for Chemical and Biological Sciences, University of Karachi, Karachi-75270, Pakistan

[2]Dr. Panjwani Center for Molecular Medicine and Drug Research, International Center for Chemical and Biological Sciences, University of Karachi, Karachi-75270, Pakistan

[3]Department of Biochemistry, Faculty of Science, King Abdulaziz University, Jeddah-21452, Saudi Arabia

Key Words:

Isoniazid, Urease Inhibitors, Anti-Inflammatory agents

*** Correspondence:**
M. Iqbal Choudhary
hej@cyber.net.pk

Hina Siddiqui
hinahej@gmail.com

Bushra / Dr. Hina / Fz-I-ISO-20
1H

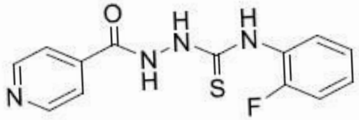

Compound 3

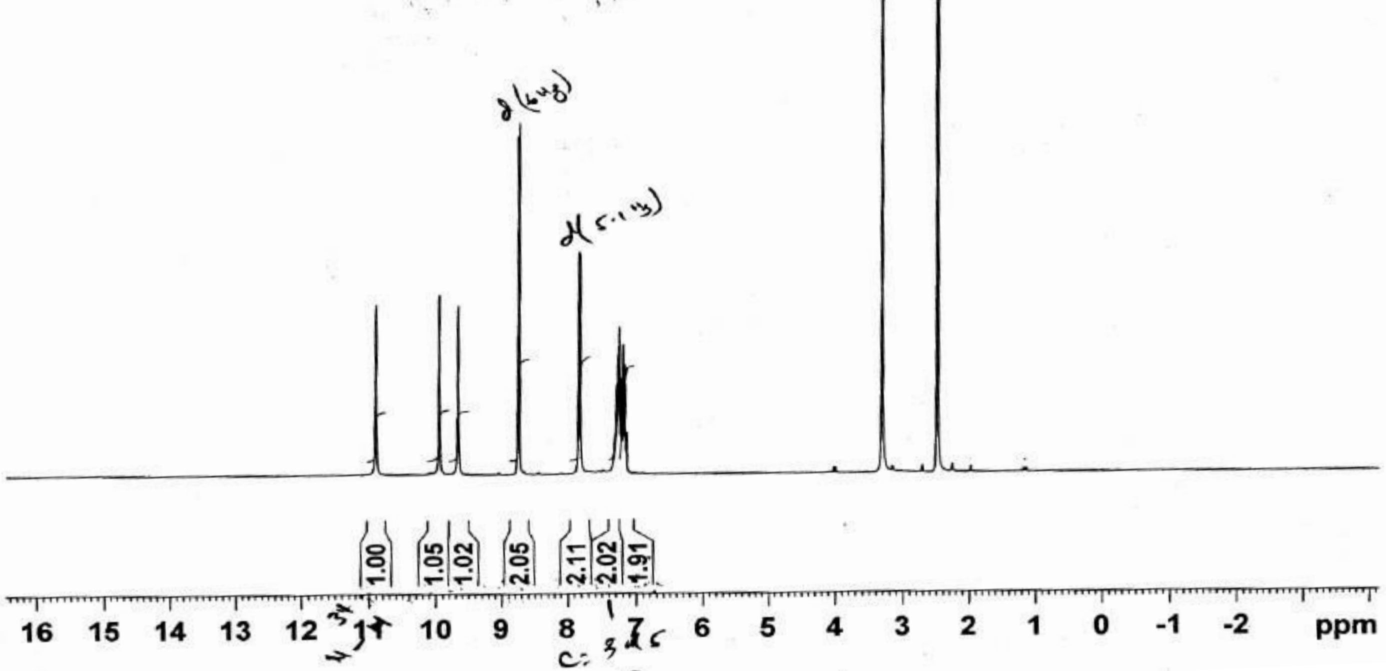

AVANCE AV - III
300 MHz, LAB # 116

NAME	Oct04-16
EXPNO	3
PROCNO	1
Date_	20161004
Time	10.36
INSTRUM	Spect
PROBHD	5 mm BBO BB-1H
PULPROG	zg30
TD	32768
SOLVENT	DMSO
NS	32
DS	0
SWH	6188.119 Hz
FIDRES	0.188846 Hz
AQ	2.6477044 sec
RG	203
DW	80.800 usec
DE	6.50 usec
TE	300.0 K
D1	2.00000000 sec
TD0	1

======== CHANNEL f1 ========

NUC1	1H
P1	12.50 usec
PL1	0.00 dB
PL1W	13.16228485 W
SFO1	300.1318534 MHz
SI	32768
SF	300.1300040 MHz
WDW	EM
SSB	0
LB	0.30 Hz
GB	0
PC	1.00

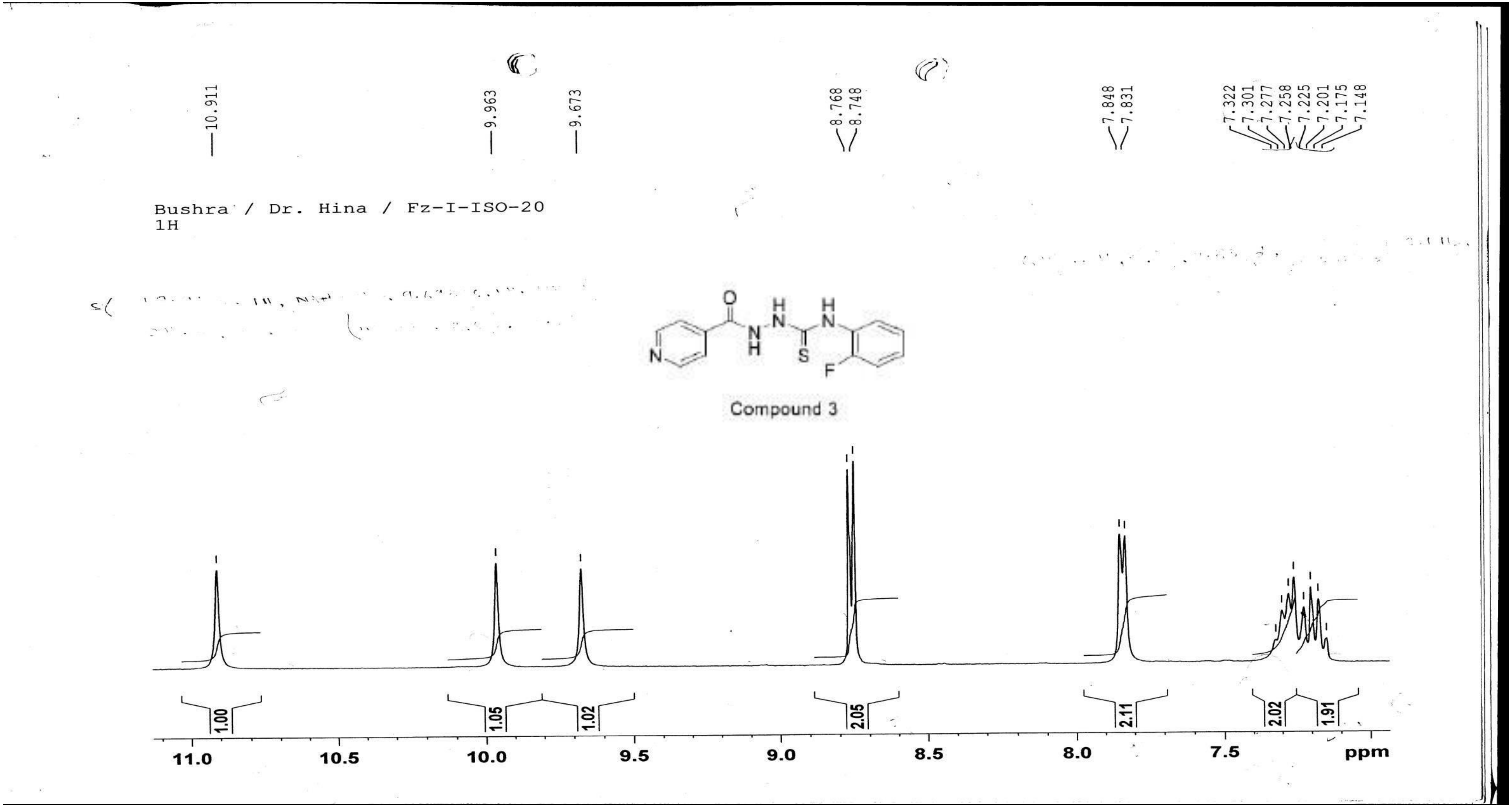

10.911
9.963
9.673
8.768
8.748
7.848
7.831
7.322
7.301
7.277
7.258
7.225
7.201
7.175
7.148
Bushra / Dr. Hina / Fz-I-ISO-20
1H
Compound 3
1.00
1.05
1.02
2.05
2.11
2.02
1.91
11.0
10.5
10.0
9.5
9.0
8.5
8.0
7.5
ppm

HEJ-ICCBS
10/5/2016 10:01:40 AM

File: FZ-I-ISO20
Sample: BUSHRA QAMAR /DR. HINA
Instrument: JEOL-600H-2
Inlet: Direct Probe

Date Run: 10-05-2016 (Time Run: 09:59:13)

Ionization mode: FAB+

Scan: 3
Base: m/z 182; 20.7%FS TIC: 986634

R.T.: .18

#Ions: 99

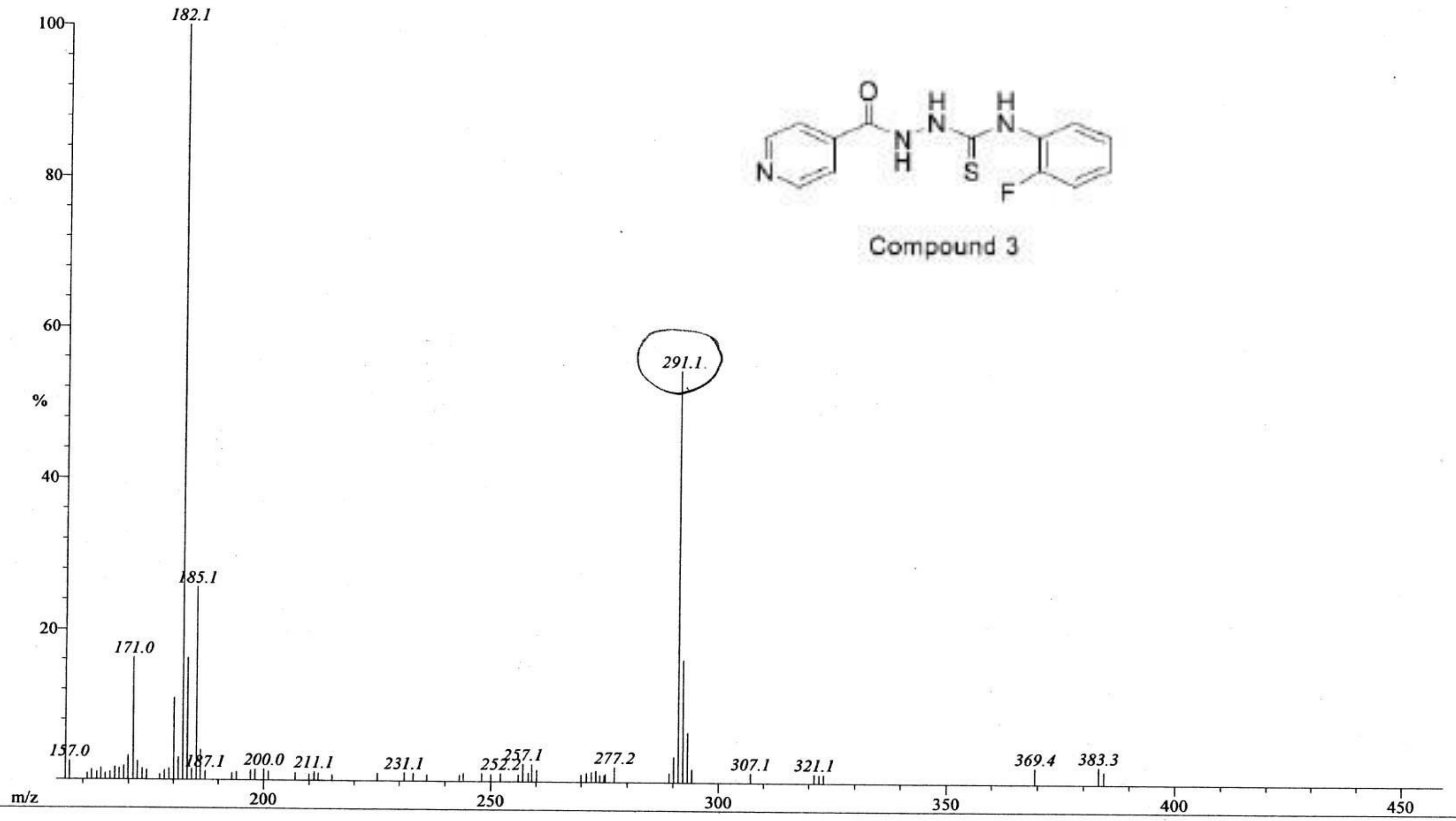

I. C.C.B.S., University of Ka[illegible]chi Analytical Laboratory - Pakistan

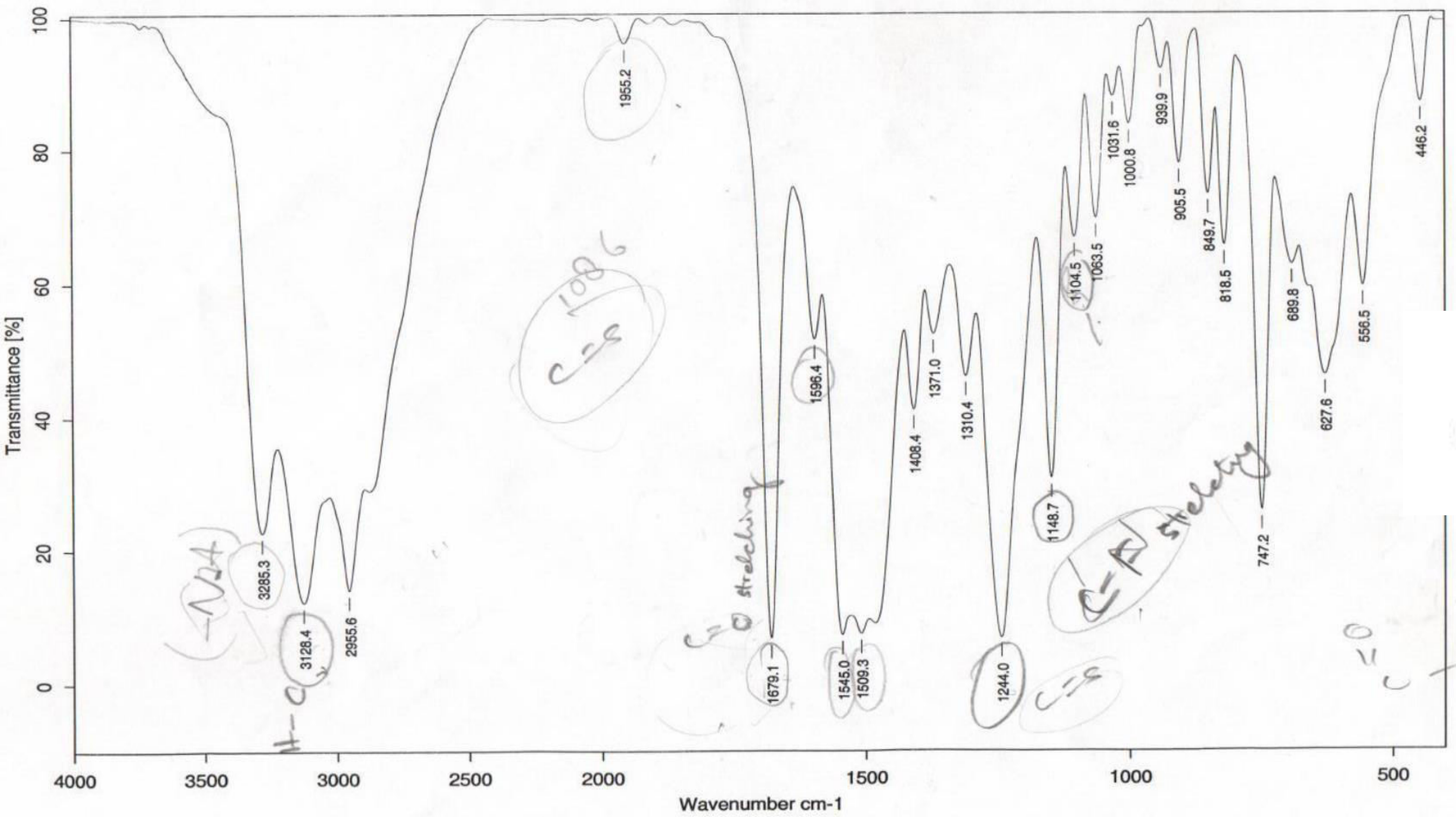

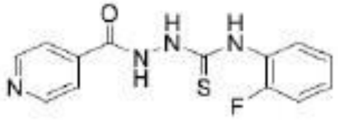

Compound 3

Sample : Fz-I-Iso20/Fazila Rizvi/Dr. Hina

Measured : 29/12/2016 on VECTOR22

Resolution : 4 cm-1 (10 scans)

Spectrum : Fz-I-Iso20.0 (in D:\IRSTUDENT)

Technic : Solid

Analyst : M. Asif

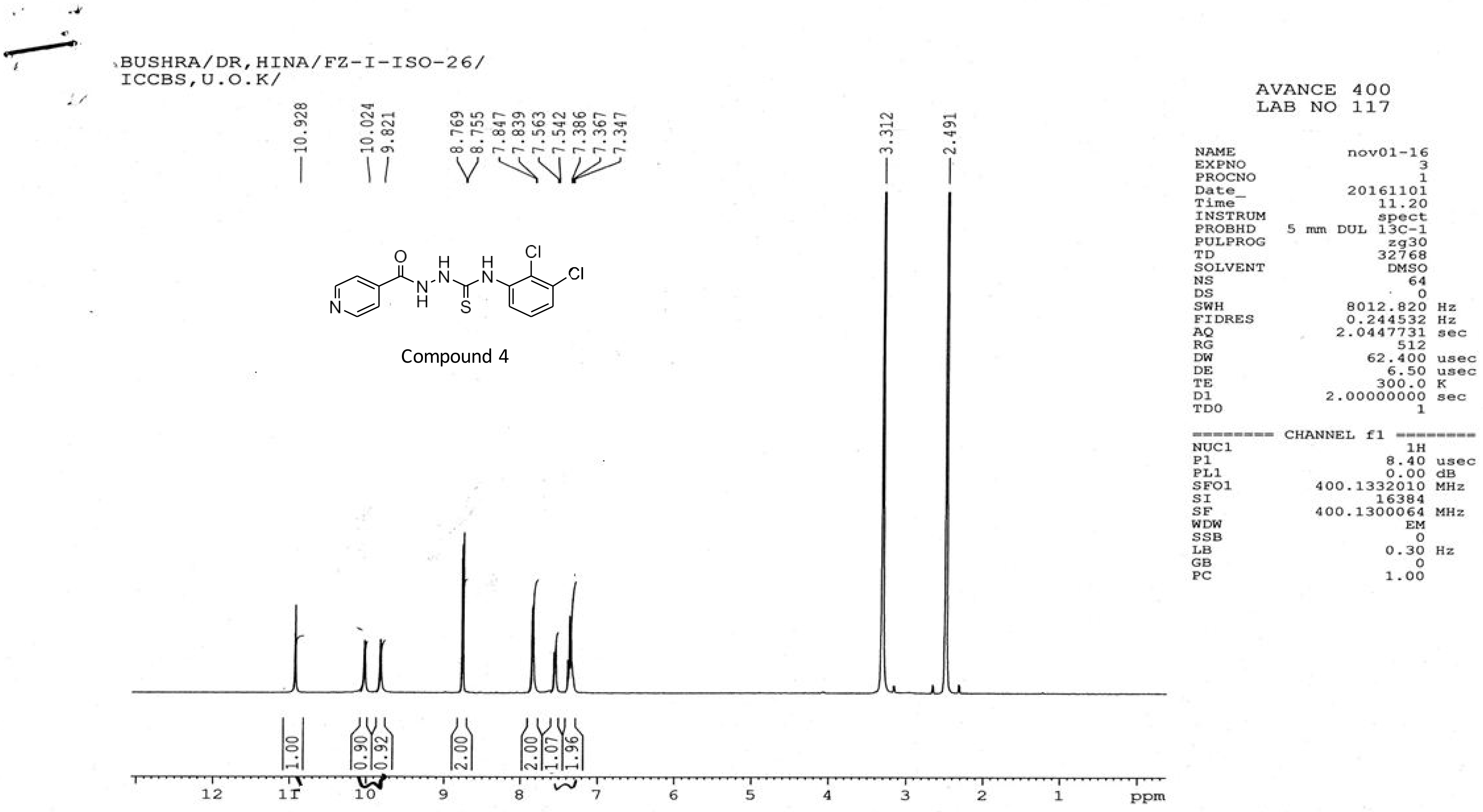

BUSHRA/DR,HINA/FZ-I-ISO-26/
ICCBS,U.O.K/
AVANCE 400
LAB NO 117
10.928
10.024
9.821
8.769
8.755
7.847
7.839
7.563
7.542
7.386
7.367
7.347
3.312
2.491
O
H
H
N
N
N
H
S
Cl
Cl
N
Compound 4
NAME nov01-16
EXPNO 3
PROCNO 1
Date_ 20161101
Time 11.20
INSTRUM spect
PROBHD 5 mm DUL 13C-1
PULPROG zg30
TD 32768
SOLVENT DMSO
NS 64
DS 0
SWH 8012.820 Hz
FIDRES 0.244532 Hz
AQ 2.0447731 sec
RG 512
DW 62.400 usec
DE 6.50 usec
TE 300.0 K
D1 2.00000000 sec
TD0 1
======== CHANNEL f1 ========
NUC1 1H
P1 8.40 usec
PL1 0.00 dB
SFO1 400.1332010 MHz
SI 16384
SF 400.1300064 MHz
WDW EM
SSB 0
LB 0.30 Hz
GB 0
PC 1.00
1.00
0.90
0.92
2.00
2.00
1.07
1.96
12
11
10
9
8
7
6
5
4
3
2
1
ppm

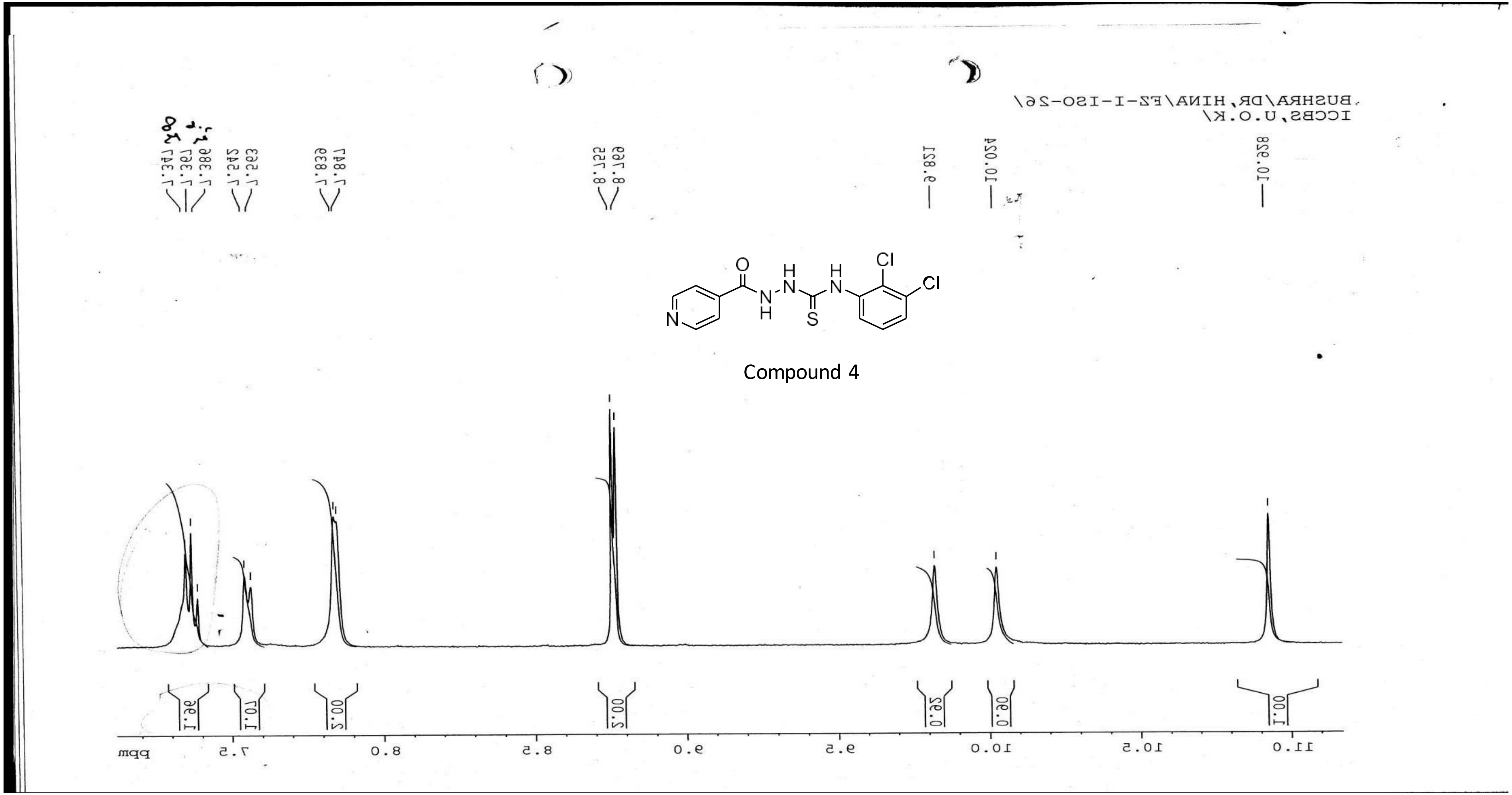

Compound 4

File: FZ-I-ISO26-FABP
Sample: BUSHRA QAMAR /DR. HINA
Instrument: JEOL-600H-2
Inlet: Direct Probe

Date Run: 10-18-2016 (Time Run: 15:43:04)

Ionization mode: FAB+

Scan: 11
Base: m/z 185; 84.7%FS TIC: 2250680

R.T.: .88

#Ions: 124

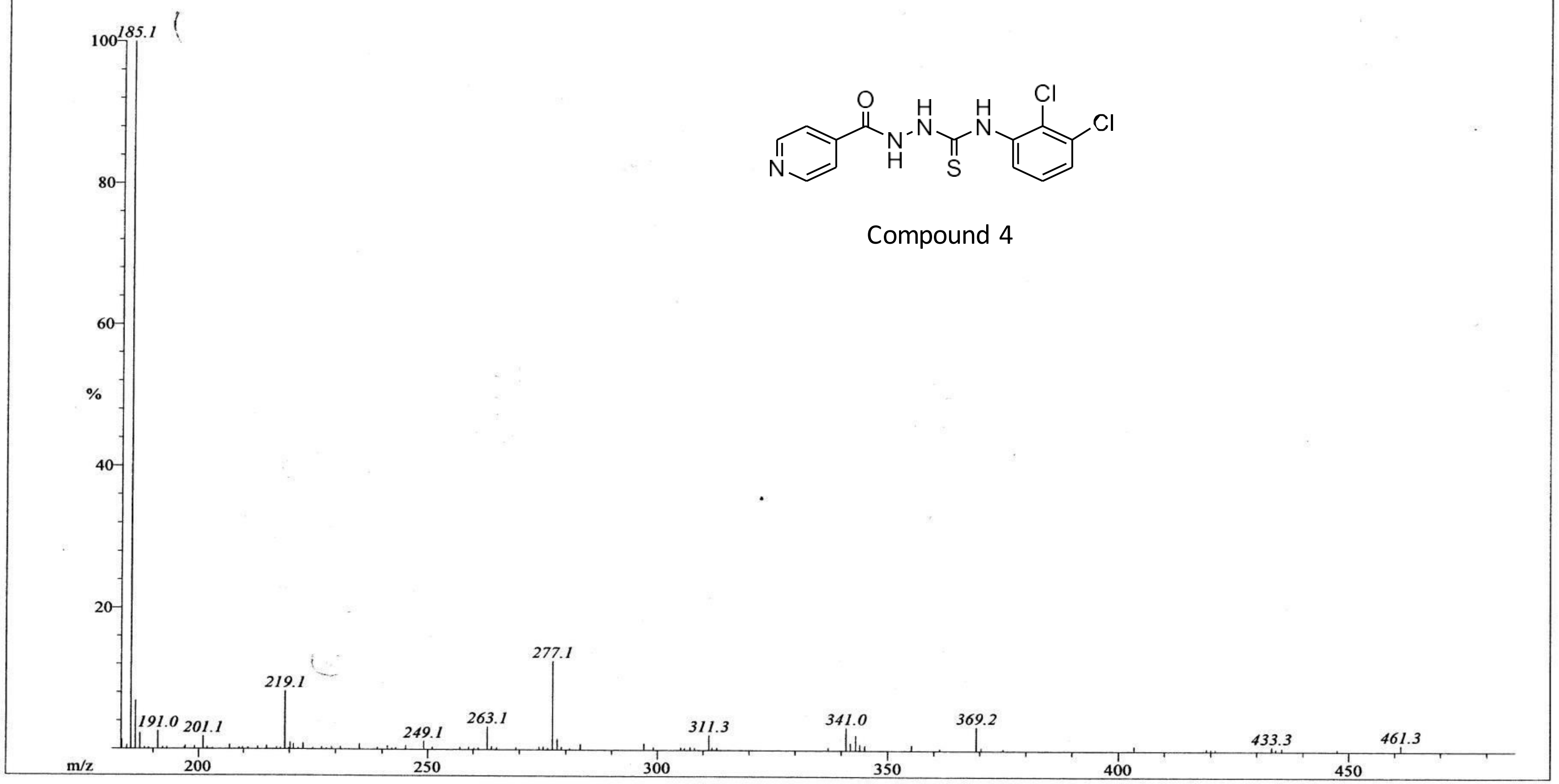

Compound 4

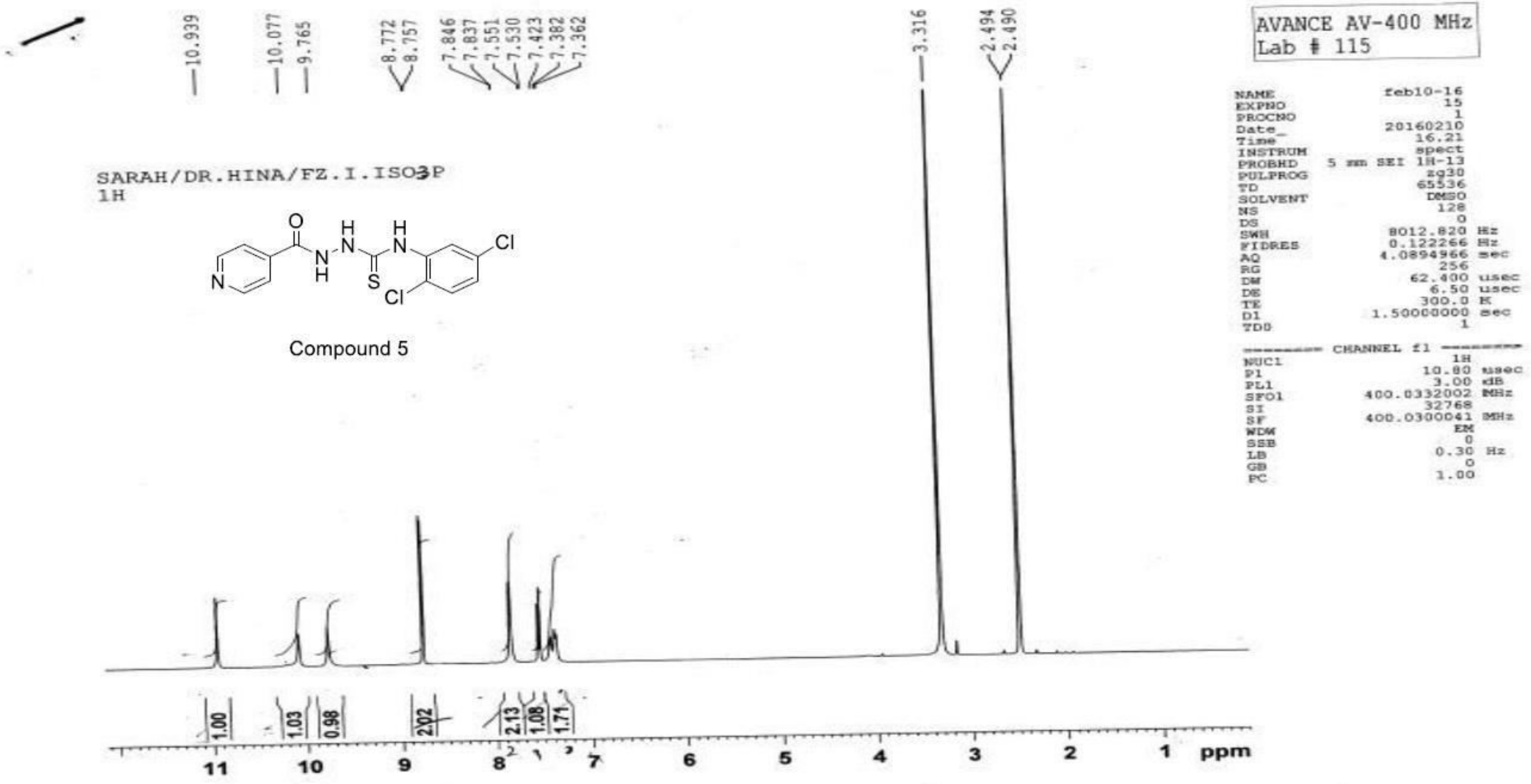

10.939
10.077
9.765
8.772
8.757
7.846
7.837
7.551
7.530
7.423
7.382
7.362
3.316
2.494
2.490
AVANCE AV-400 MHz
Lab # 115
SARAH/DR.HINA/FZ.I.ISO3P
1H
NAME feb10-16
EXPNO 15
PROCNO 1
Date_ 20160210
Time 16.21
INSTRUM spect
PROBHD 5 mm SEI 1H-13
PULPROG zg30
TD 65536
SOLVENT DMSO
NS 128
DS 0
SWH 8012.820 Hz
FIDRES 0.122266 Hz
AQ 4.0894966 sec
RG 256
DW 62.400 usec
DE 6.50 usec
TE 300.0 K
D1 1.50000000 sec
TD0 1
CHANNEL f1
NUC1 1H
P1 10.80 usec
PL1 3.00 dB
SFO1 400.0332002 MHz
SI 32768
SF 400.0300041 MHz
WDW EM
SSB 0
LB 0.30 Hz
GB 0
PC 1.00
1.00
1.03
0.98
2.02
2.13
1.08
1.71
11
10
9
8
7
6
5
4
3
2
1
ppm

Compound 5

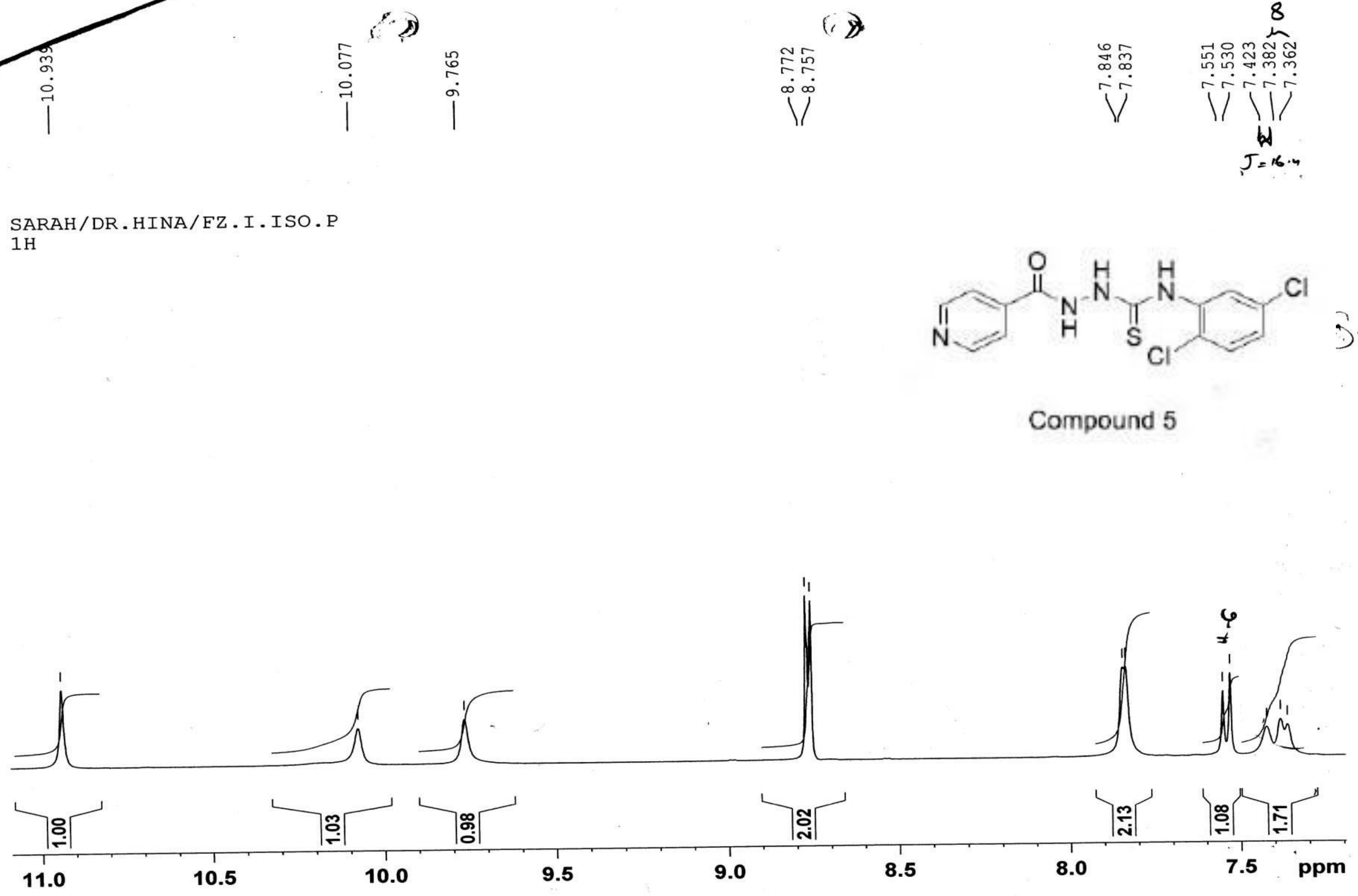
10.939
10.077
9.765
8.772
8.757
7.846
7.837
7.551
7.530
7.423
7.382
7.362
SARAH/DR.HINA/FZ.I.ISO.P
1H
1.00
1.03
0.98
2.02
2.13
1.08
1.71
11.0
10.5
10.0
9.5
9.0
8.5
8.0
7.5
ppm

Compound 5

4/6/2016 10:06:05 AM

File: FZ-I-ISO3(P)-FABN-1
Sample: FAZILA /DR. HINA
Instrument: JEOL-600H-2
Inlet: Direct Probe

Date Run: 04-06-2016 (Time Run: 10:03:56)

Ionization mode: FAB-

Scan: 7
Base: m/z 183; 39.2%FS TIC: 1179980

R.T.: .53

#Ions: 354

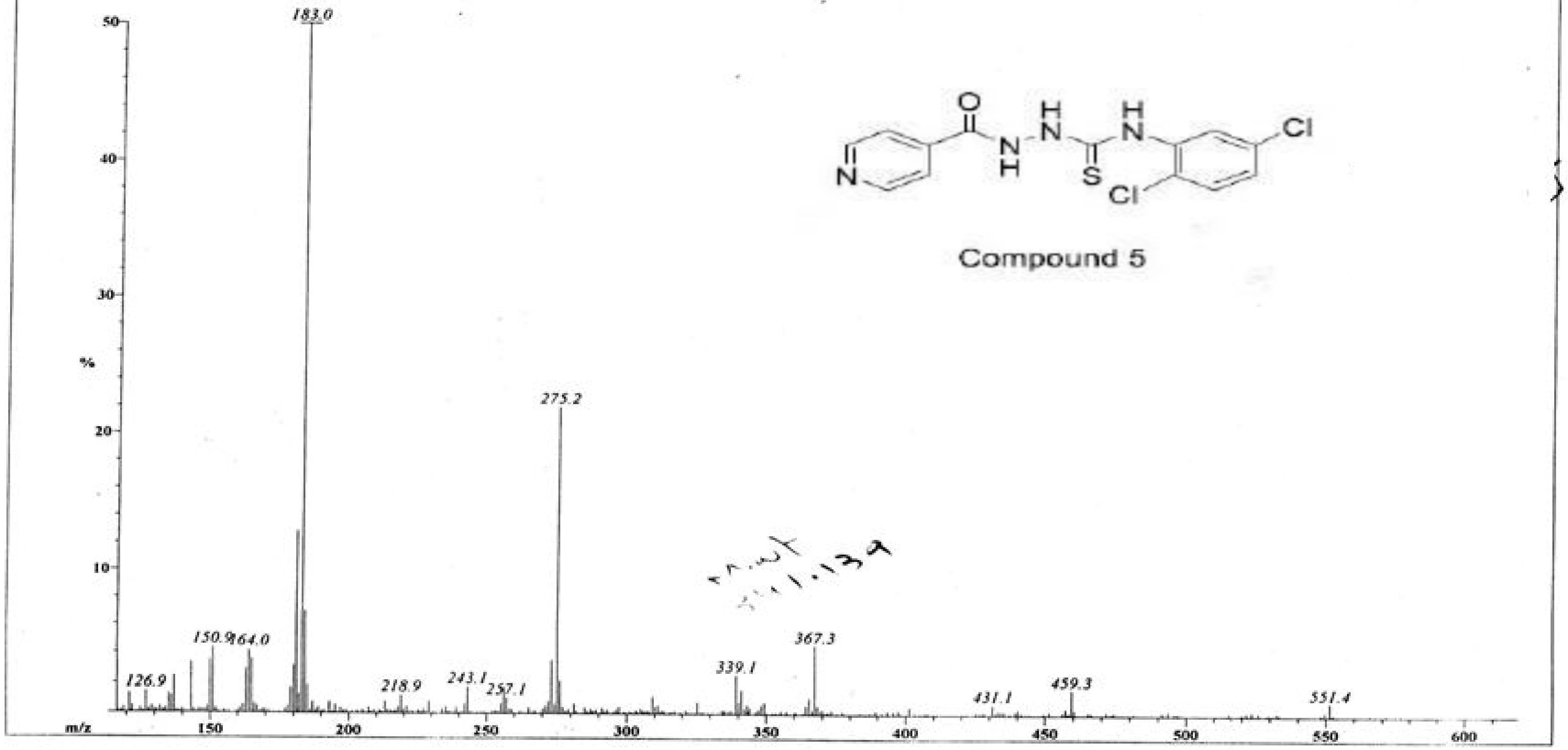

I. C.C.B.S., University of Ka[illegible]hi Analytical Laboratory - Pakistan

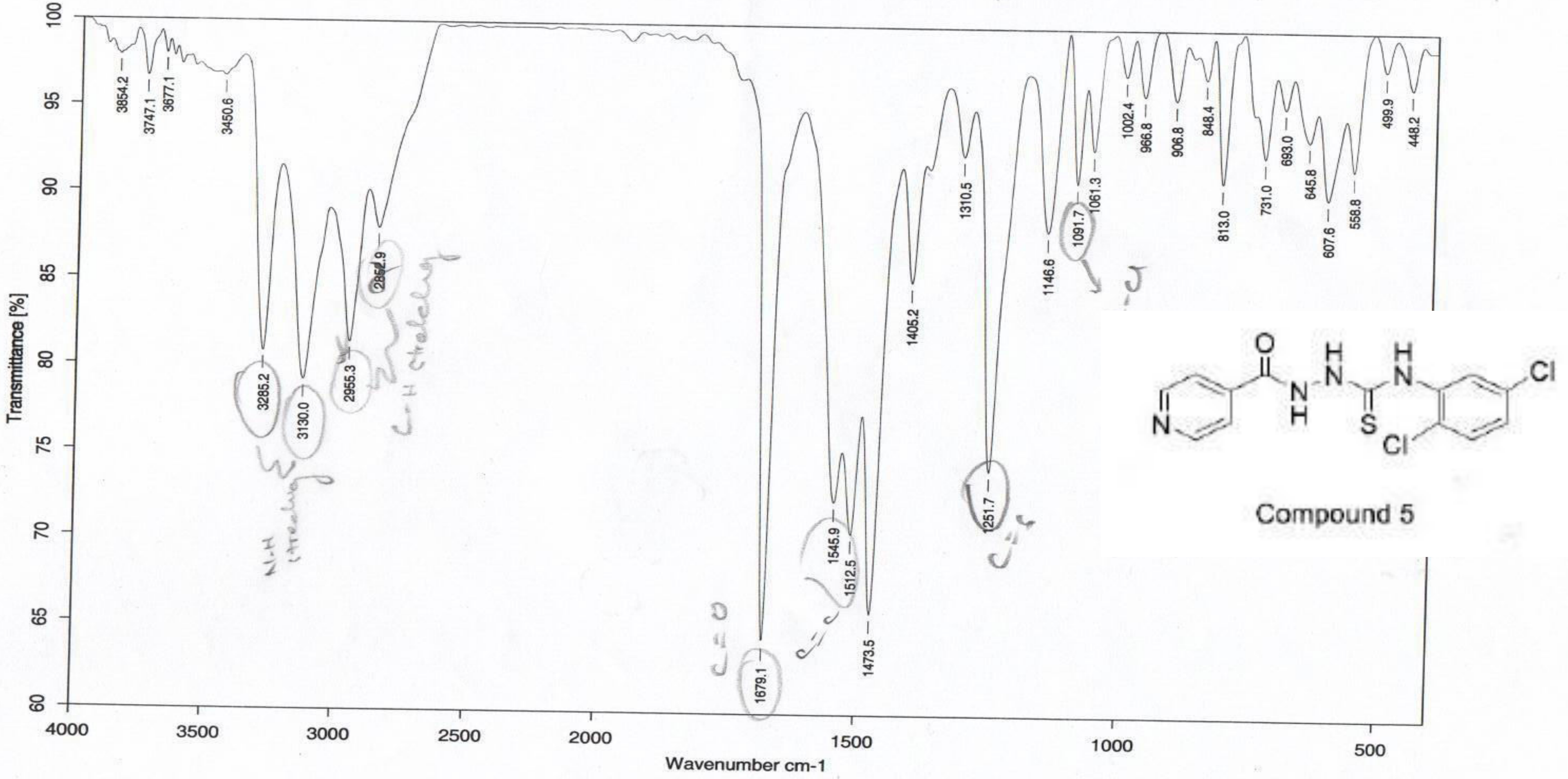

Sample : FZI-ISO3/Fazila Rizvi

Measured : 19/04/2017 on VECTOR22

Resolution : 4 cm-1 (10 scans)

Spectrum : FZ-I-ISO3.0 (in D:\IRSTUDENT)

Technic : Solid

Analyst : MA/ZA/JS

Fazila / Dr. Hina / Fz-I-Iso-4
1H

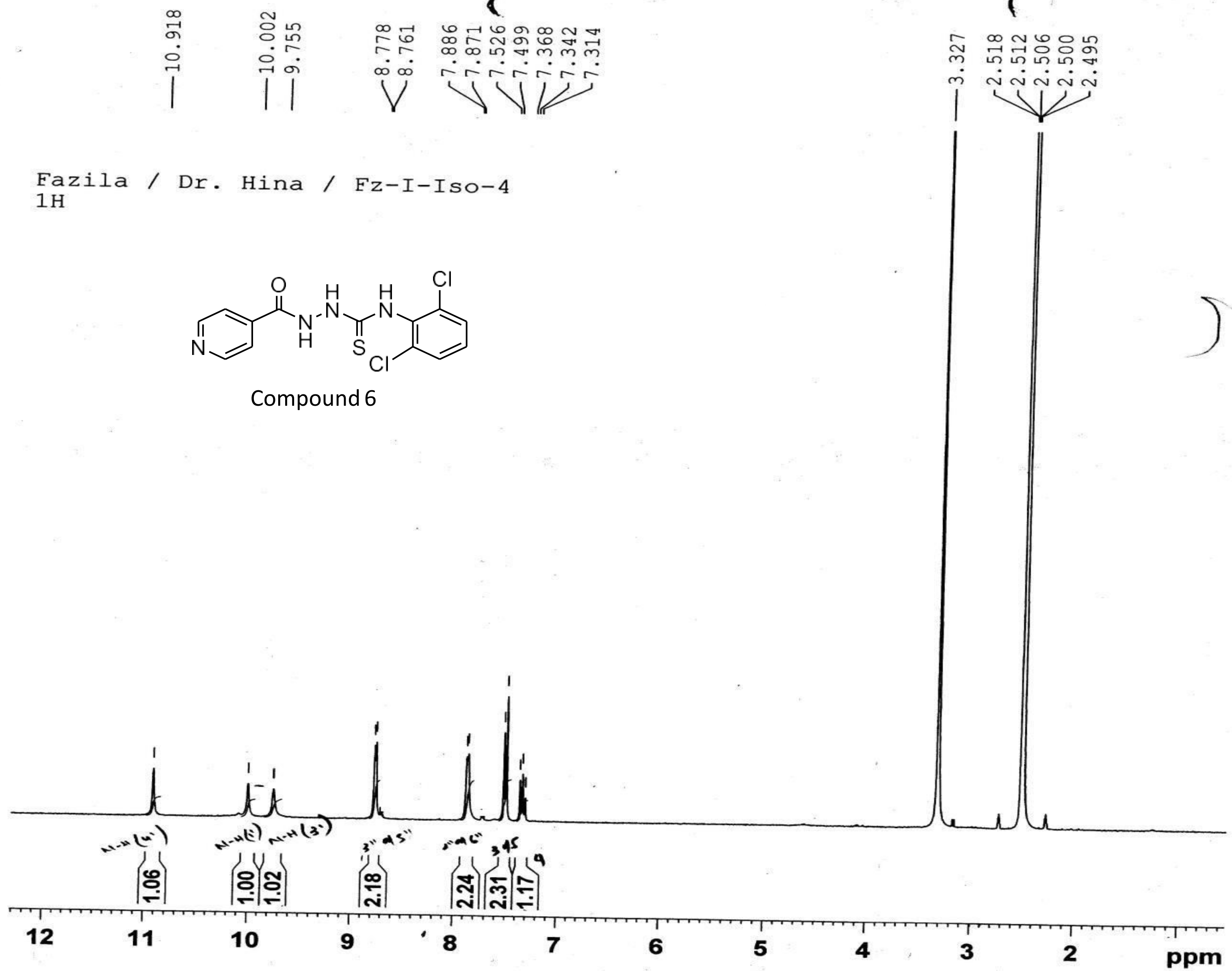

Compound 6

AVANCE AV - III
300 MHz, LAB # 116

NAME	mar22-16
EXPNO	1
PROCNO	1
Date_	20160322
Time	13.13
INSTRUM	Spect
PROBHD	5 mm BBO BB-1H
PULPROG	zg30
TD	32768
SOLVENT	DMSO
NS	128
DS	0
SWH	6188.119 Hz
FIDRES	0.188846 Hz
AQ	2.6477044 sec
RG	203
DW	80.800 usec
DE	6.50 usec
TE	300.0 K
D1	2.00000000 sec
TD0	1

======== CHANNEL f1 ========

NUC1	1H
P1	12.50 usec
PL1	0.00 dB
PL1W	13.16228485 W
SFO1	300.1318534 MHz
SI	32768
SF	300.1299992 MHz
WDW	EM
SSB	0
LB	0.30 Hz
GB	0
PC	1.00

Fazila / Dr. Hina / Fz-I-Iso-4
1H

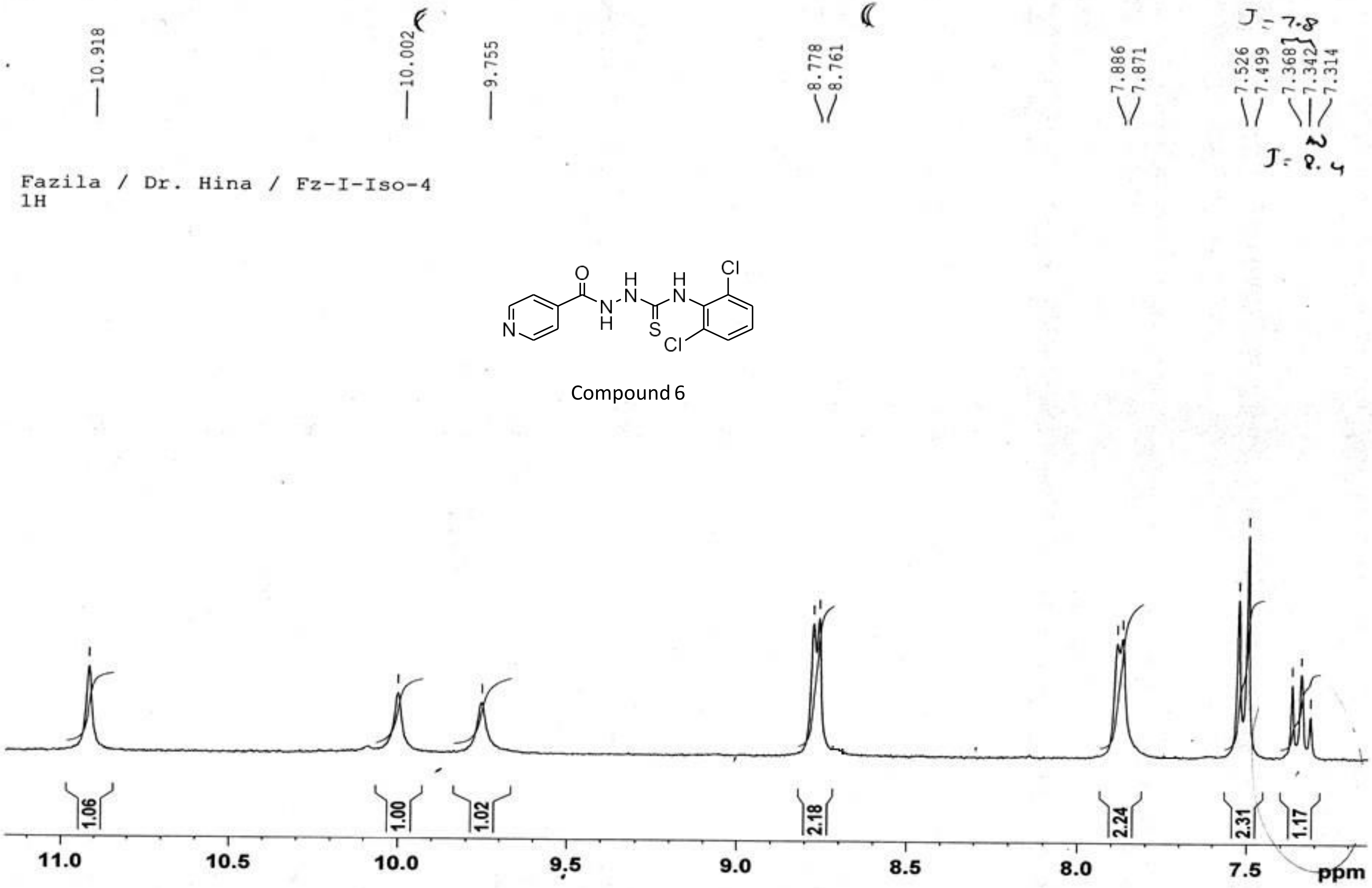

Compound 6

HEJ-ICCBS
3/3/2016 1:25:02 PM

File: FZ-I-ISO4(P)-FABN
Sample: FAZILA /DR. HINA
Instrument: JEOL-600H-2
Inlet: Direct Probe

Date Run: 03-03-2016 (Time Run: 13:21:15)

Ionization mode: FAB-

Scan: 12
Base: m/z 183; 44.1%FS TIC: 2962072

R.T.: .97

#Ions: 676

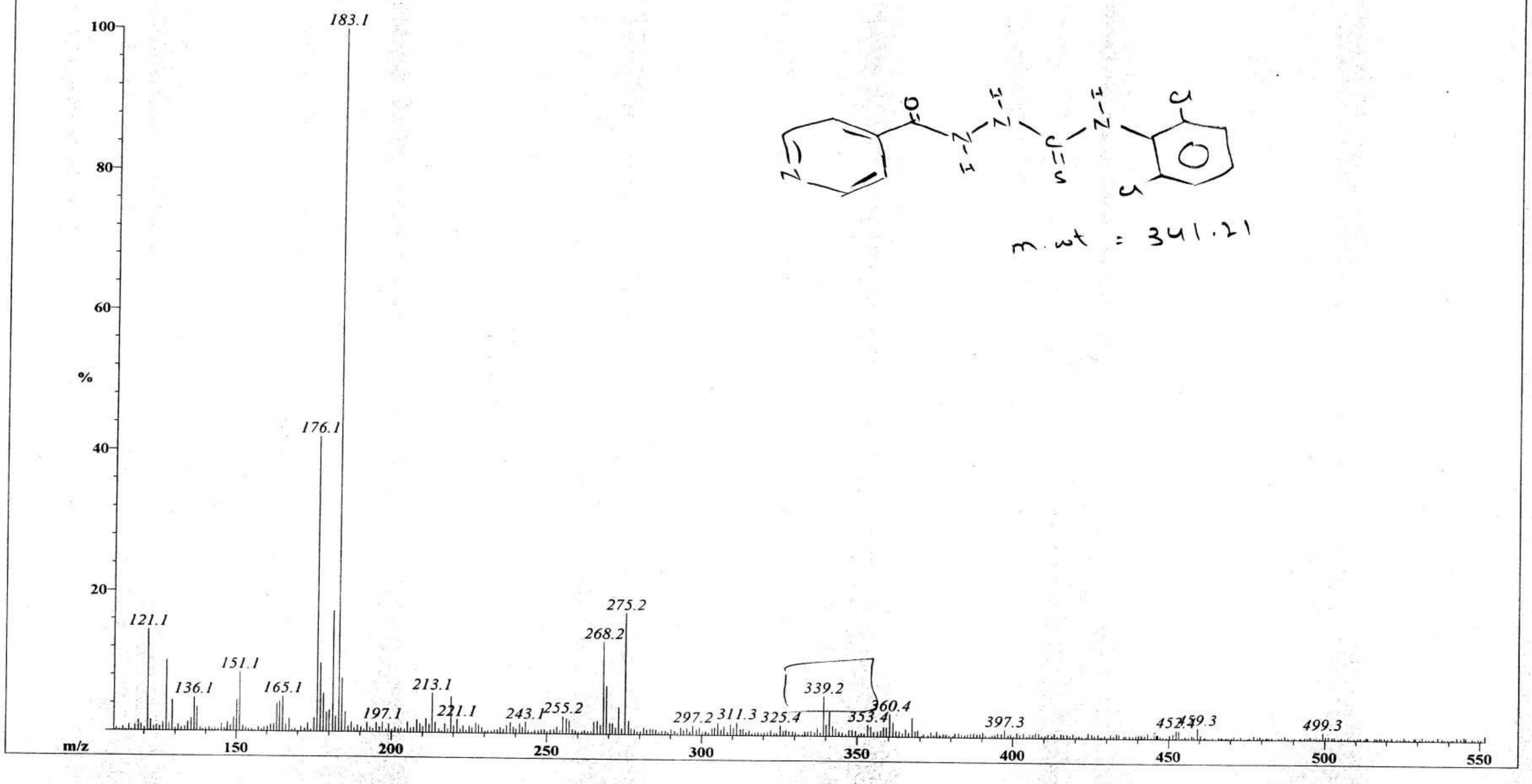

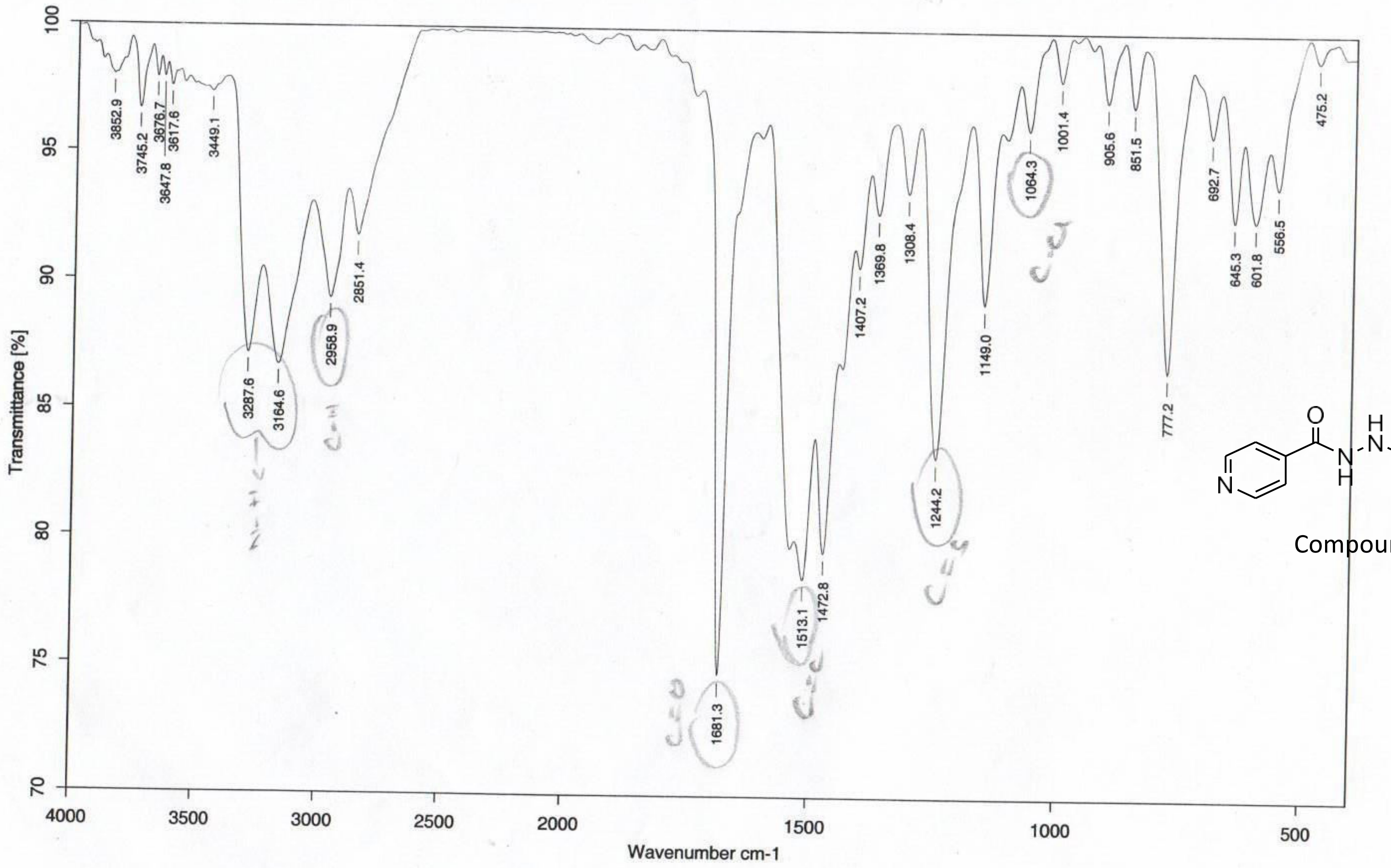

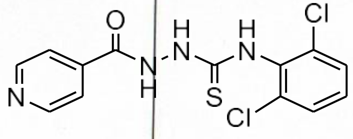

Compound 6

Sample : FZI-ISO4/Fazila Rizvi	Spectrum : FZ-I-ISO4.0 (in D:\IRSTUDENT)
Measured : 19/04/2017 on VECTOR22	Technic : Solid
Resolution : 4 cm-1 (10 scans)	Analyst : MA/ZA/JS

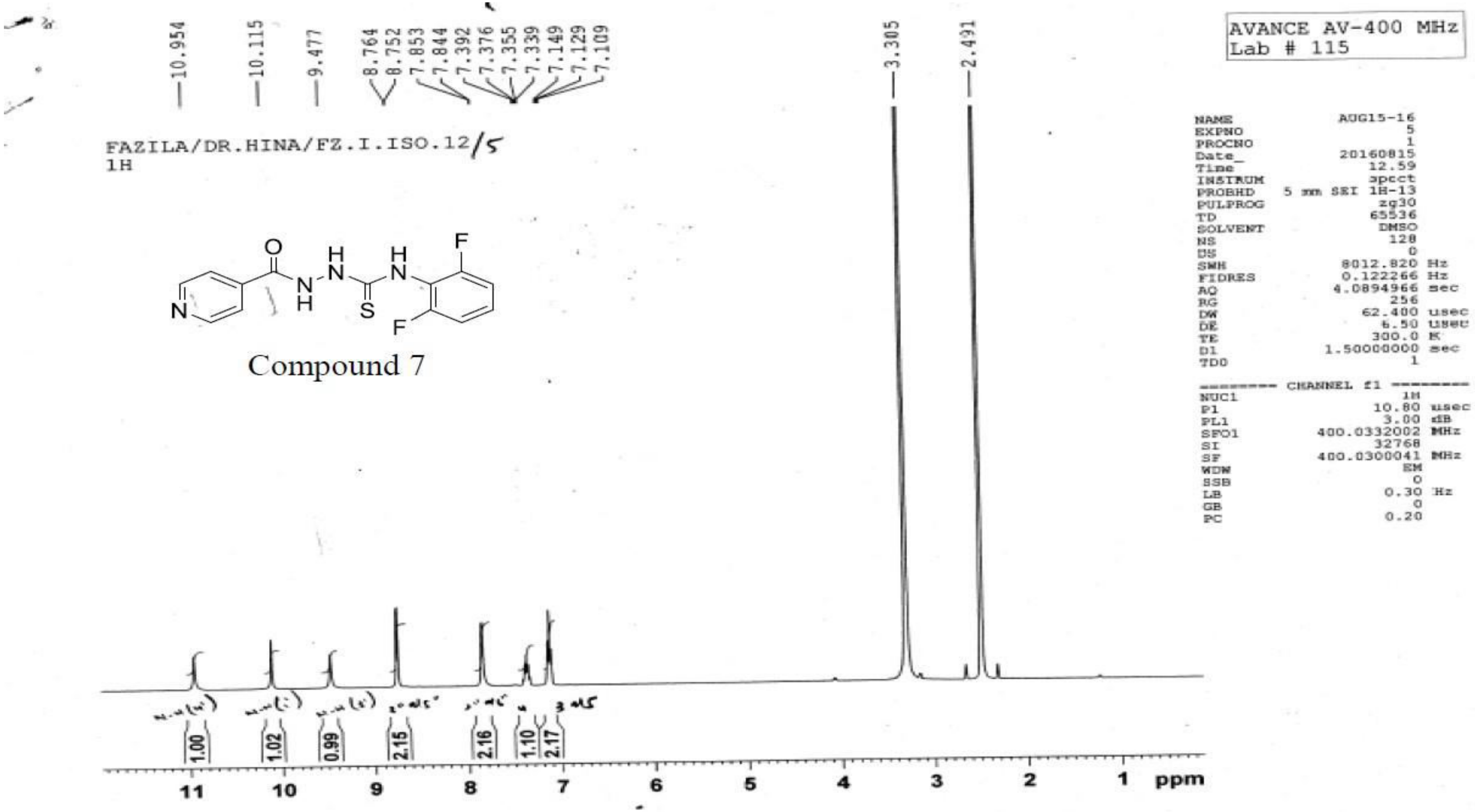
10.954
10.115
9.477
8.764
8.752
7.853
7.844
7.392
7.376
7.355
7.339
7.149
7.129
7.109
3.305
2.491
AVANCE AV-400 MHz
Lab # 115
FAZILA/DR.HINA/FZ.I.ISO.12/5
1H
Compound 7
NAME AUG15-16
EXPNO 5
PROCNO 1
Date_ 20160815
Time 12.59
INSTRUM spect
PROBHD 5 mm SEI 1H-13
PULPROG zg30
TD 65536
SOLVENT DMSO
NS 128
DS 0
SWH 8012.820 Hz
FIDRES 0.122266 Hz
AQ 4.0894966 sec
RG 256
DW 62.400 usec
DE 6.50 usec
TE 300.0 K
D1 1.50000000 sec
TD0 1
CHANNEL f1
NUC1 1H
P1 10.80 usec
PL1 3.00 dB
SFO1 400.0332002 MHz
SI 32768
SF 400.0300041 MHz
WDW EM
SSB 0
LB 0.30 Hz
GB 0
PC 0.20
1.00
1.02
0.99
2.15
2.16
1.10
2.17
11
10
9
8
7
6
5
4
3
2
1
ppm

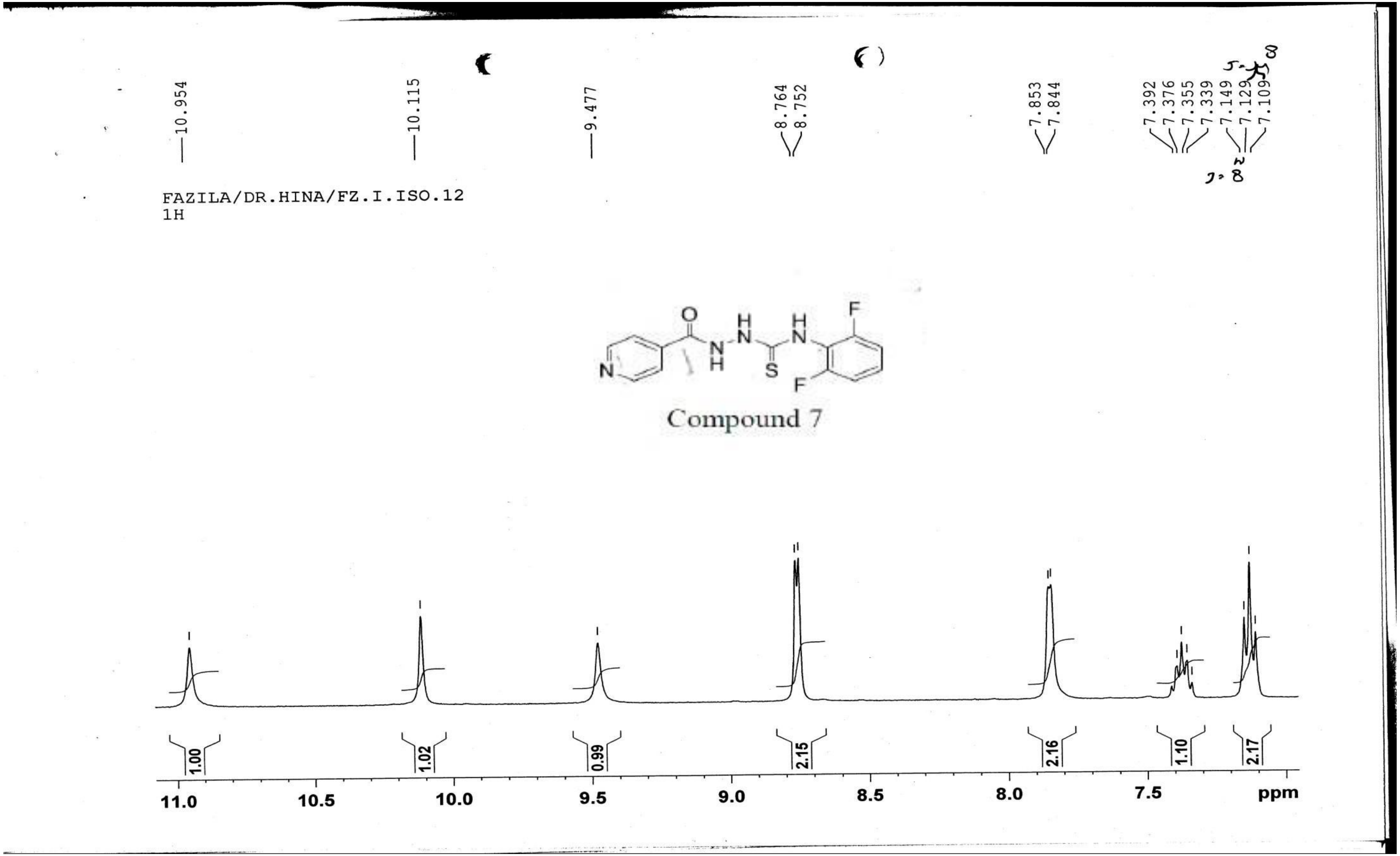

10.954
10.115
9.477
8.764
8.752
7.853
7.844
7.392
7.376
7.355
7.339
7.149
7.129
7.109
FAZILA/DR.HINA/FZ.I.ISO.12
1H
Compound 7
1.00
1.02
0.99
2.15
2.16
1.10
2.17
11.0
10.5
10.0
9.5
9.0
8.5
8.0
7.5
ppm

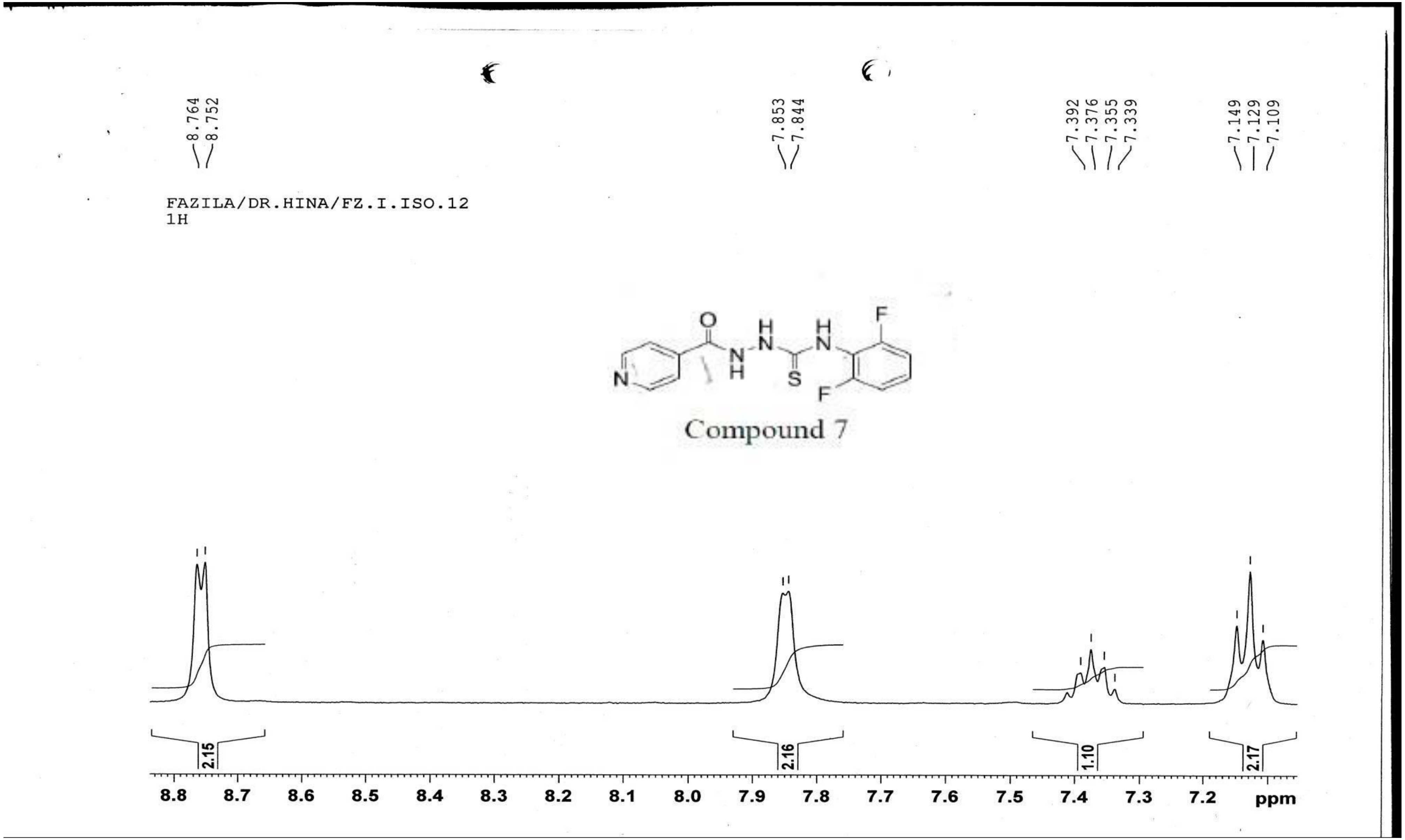
8.764
8.752
7.853
7.844
7.392
7.376
7.355
7.339
7.149
7.129
7.109
FAZILA/DR.HINA/FZ.I.ISO.12
1H
O
H
N
N
H
H
N
S
F
F
N
2.15
2.16
1.10
2.17
8.8
8.7
8.6
8.5
8.4
8.3
8.2
8.1
8.0
7.9
7.8
7.7
7.6
7.5
7.4
7.3
7.2
ppm
Compound 7

HEJ-ICCBS
8/15/2016 4:08:13 PM

File: FZ-I-ISO-12
Sample: FAZILA RIZVI /DR. HINA
Instrument: JEOL-600H-2
Inlet: Direct Probe

Date Run: 08-15-2016 (Time Run: 16:05:45)

Ionization mode: FAB+

Scan: 4
Base: m/z 185; 100%FS TIC: 6513332

R.T.: .27

#Ions: 302

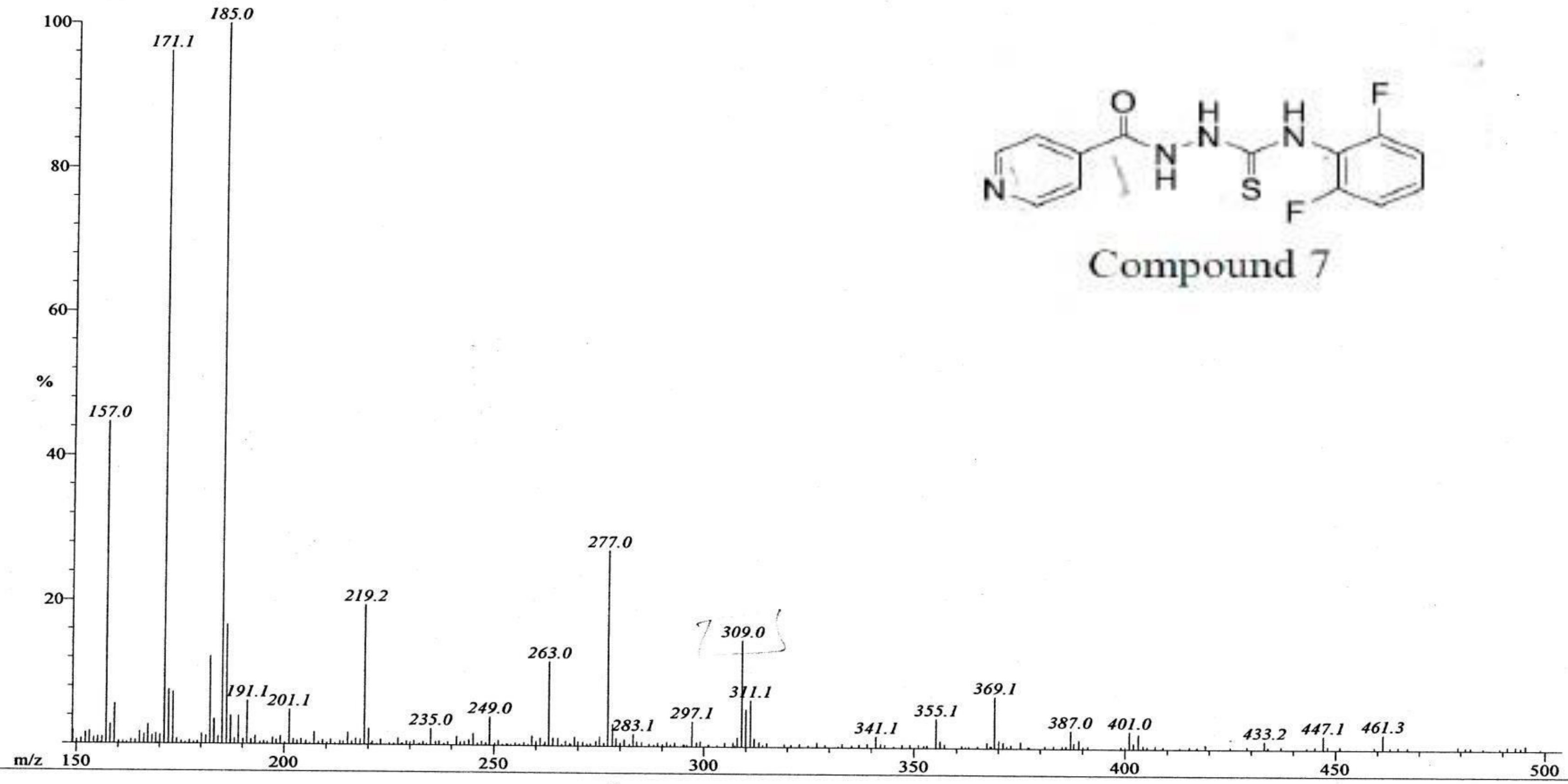

Compound 7

I. C.C.B.S., University of Ka[illegible]hi Analytical Laboratory - Pakistan

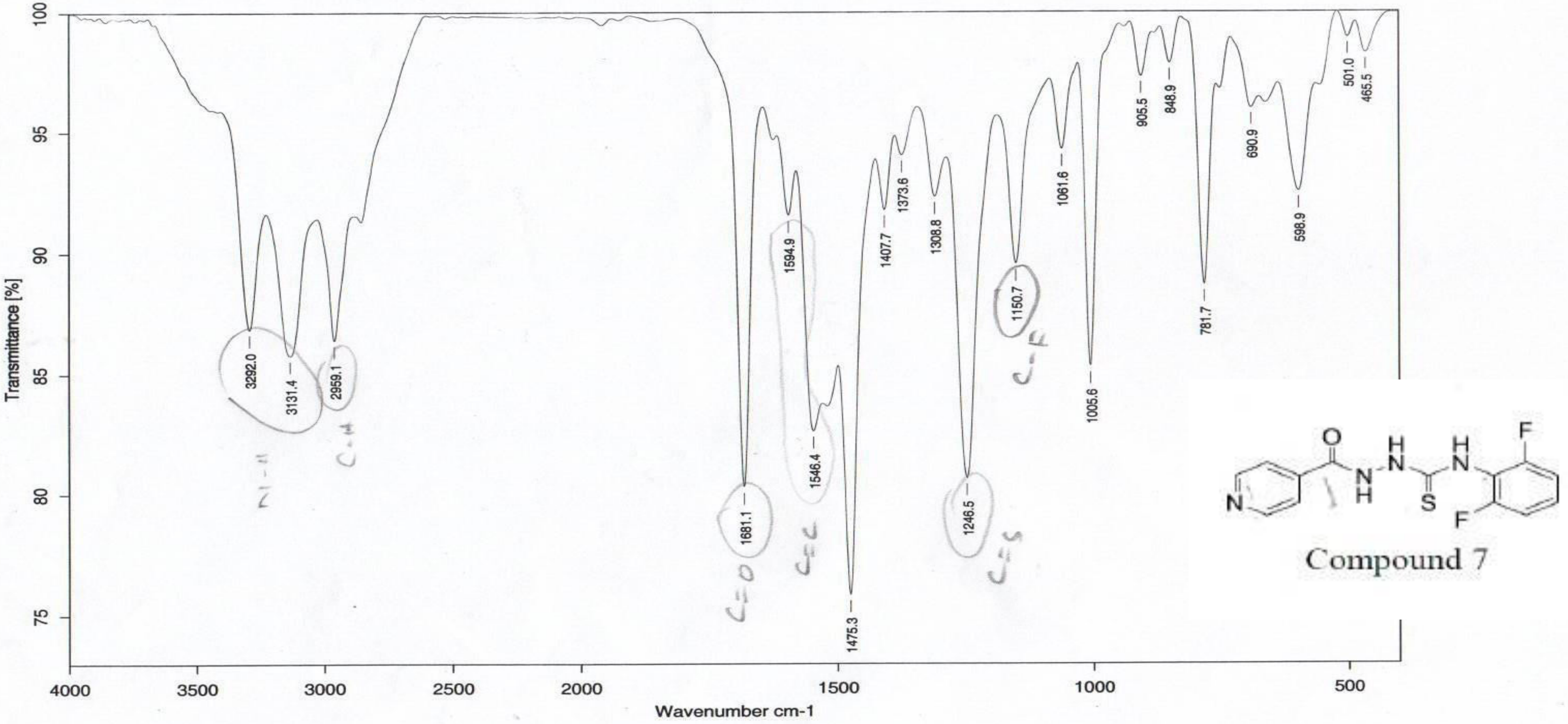

Compound 7

Sample : FZI-ISO12/Fazila Rizvi

Measured : 19/04/2017 on VECTOR22

Resolution : 4 cm-1 (10 scans)

Spectrum : FZ-I-ISO12.0 (in D:\IRSTUDENT)

Technic : Solid

Analyst : MA/ZA/JS

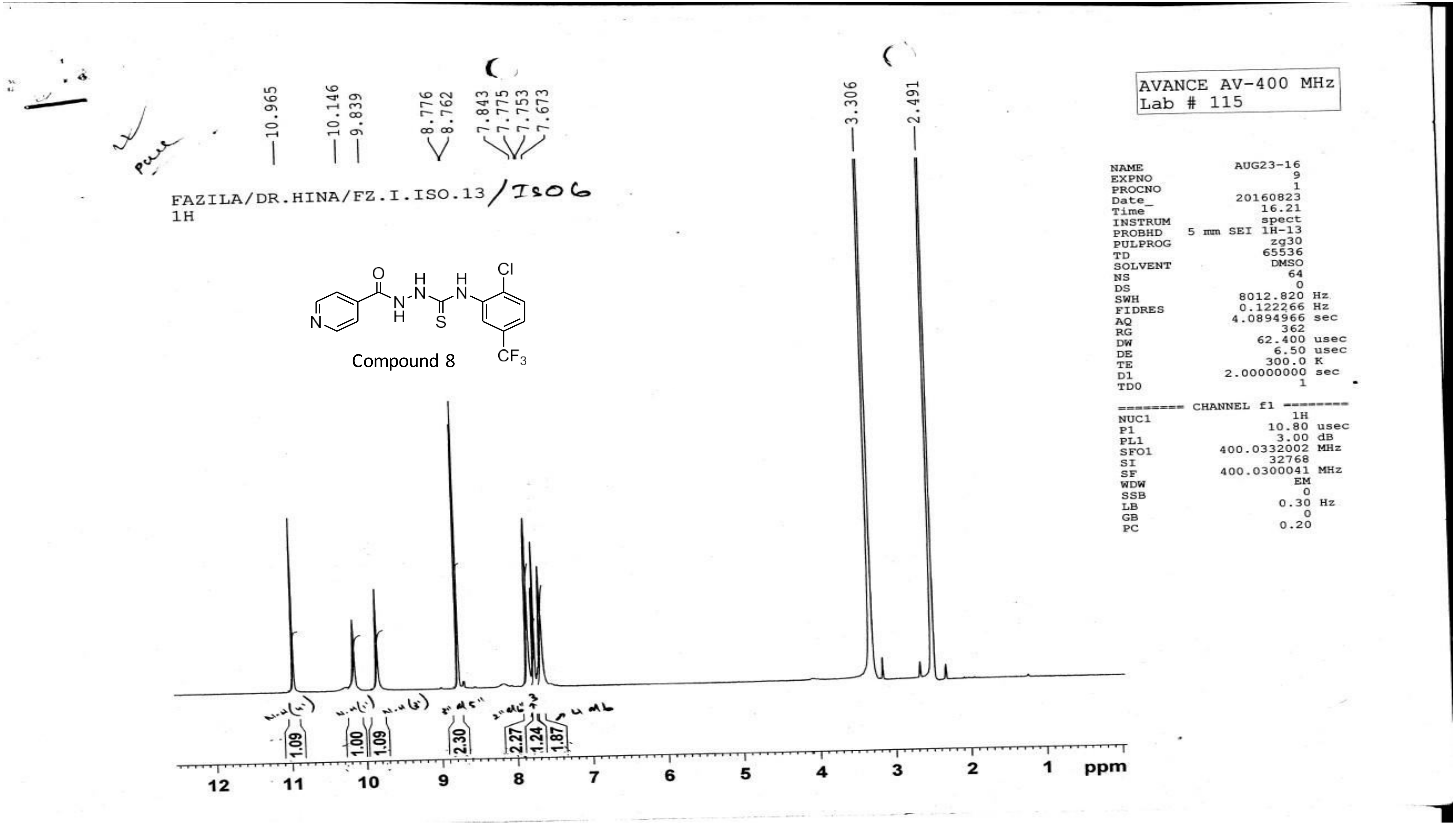

10.965
10.146
9.839
8.776
8.762
7.843
7.775
7.753
7.673
3.306
2.491
FAZILA/DR.HINA/FZ.I.ISO.13 / ISO6
1H
Compound 8
AVANCE AV-400 MHz
Lab # 115
NAME AUG23-16
EXPNO 9
PROCNO 1
Date_ 20160823
Time 16.21
INSTRUM spect
PROBHD 5 mm SEI 1H-13
PULPROG zg30
TD 65536
SOLVENT DMSO
NS 64
DS 0
SWH 8012.820 Hz
FIDRES 0.122266 Hz
AQ 4.0894966 sec
RG 362
DW 62.400 usec
DE 6.50 usec
TE 300.0 K
D1 2.00000000 sec
TD0 1
======== CHANNEL f1 ========
NUC1 1H
P1 10.80 usec
PL1 3.00 dB
SFO1 400.0332002 MHz
SI 32768
SF 400.0300041 MHz
WDW EM
SSB 0
LB 0.30 Hz
GB 0
PC 0.20
1.09
1.00
1.09
2.30
2.27
1.24
1.87
12
11
10
9
8
7
6
5
4
3
2
1
ppm

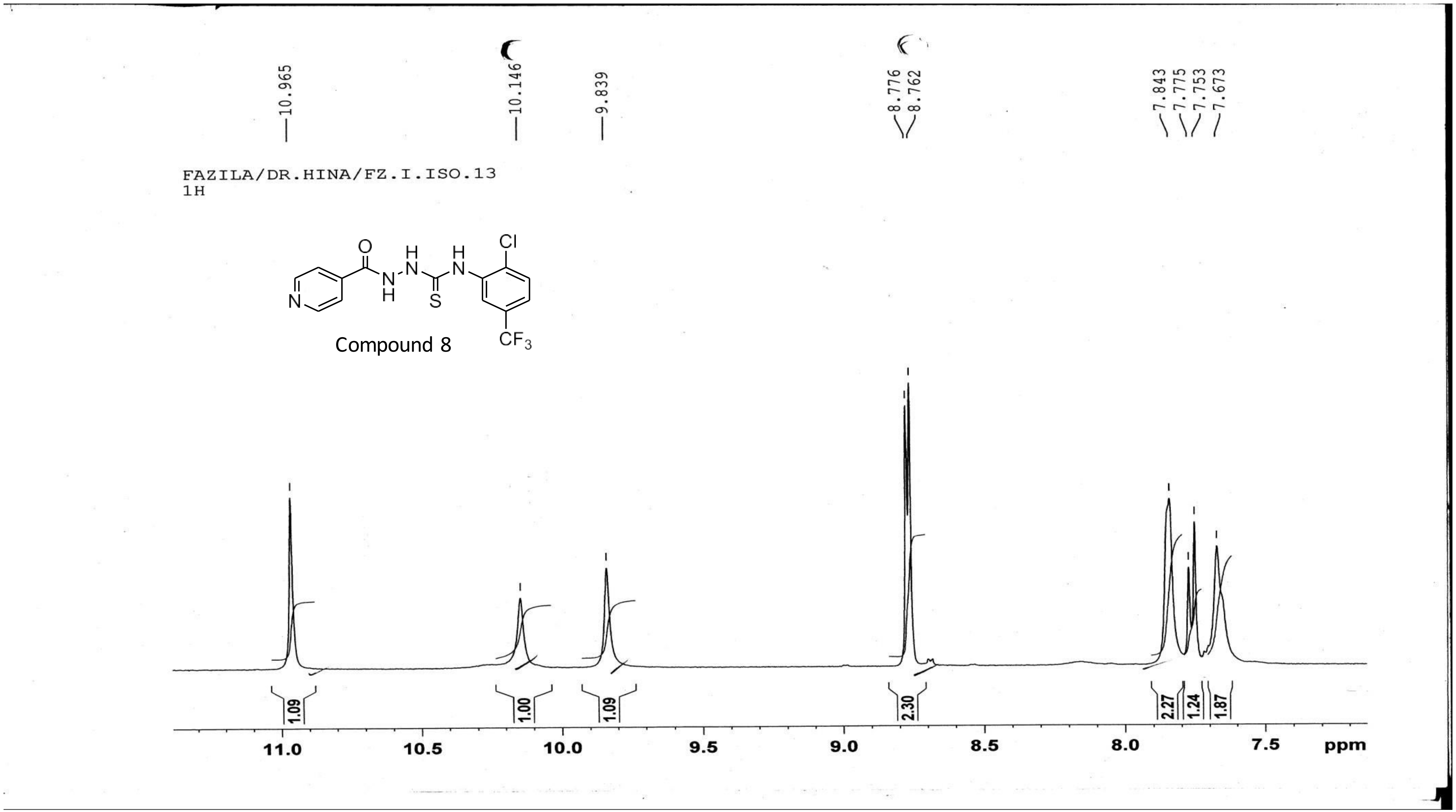
10.965
10.146
9.839
8.776
8.762
7.843
7.775
7.753
7.673
FAZILA/DR.HINA/FZ.I.ISO.13
1H
Compound 8
1.09
1.00
1.09
2.30
2.27
1.24
1.87
11.0
10.5
10.0
9.5
9.0
8.5
8.0
7.5
ppm

File: FZ-I-ISO6(Me)-FABN
Sample: FAZILA /DR. HINA
Instrument: JEOL-600H-2
Inlet: Direct Probe

Date Run: 04-06-2016 (Time Run: 10:12:59)

Ionization mode: FAB-

Scan: 2
Base: m/z 373; 17.1%FS TIC: 1081342

R.T.: .1

#Ions: 353

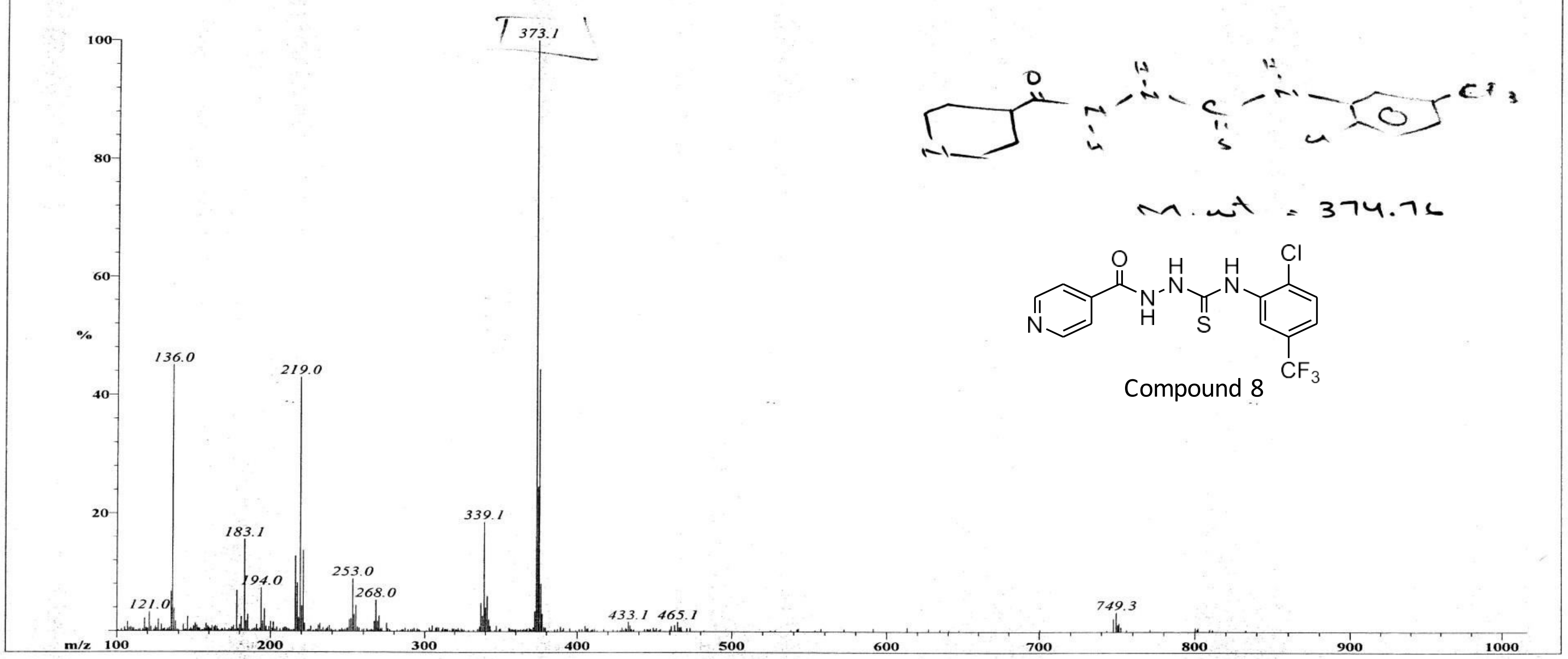

Compound 8

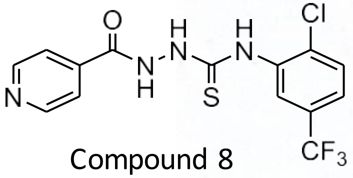

Compound 8

I. C.C.B.S., University of Ka[illegible]hi Analytical Laboratory - Pakistan

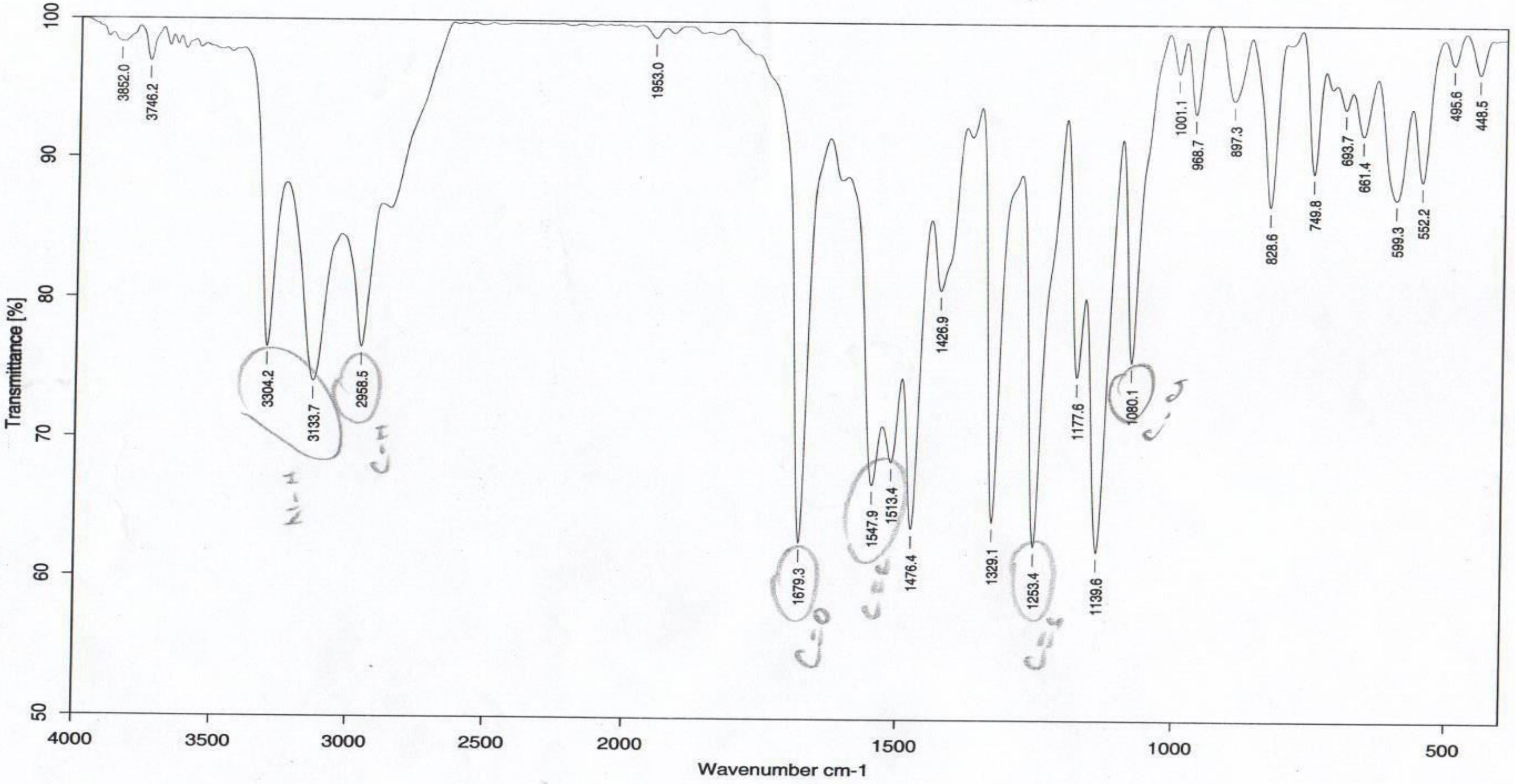

Sample :	FZI-ISO6/Fazila Rizvi	Spectrum	: FZ-I-ISO6.0 (in D:\IRSTUDENT)
Measured :	19/04/2017 on VECTOR22	Technic	: Solid
Resolution :	4 cm-1 (10 scans)	Analyst	: MA/ZA/JS

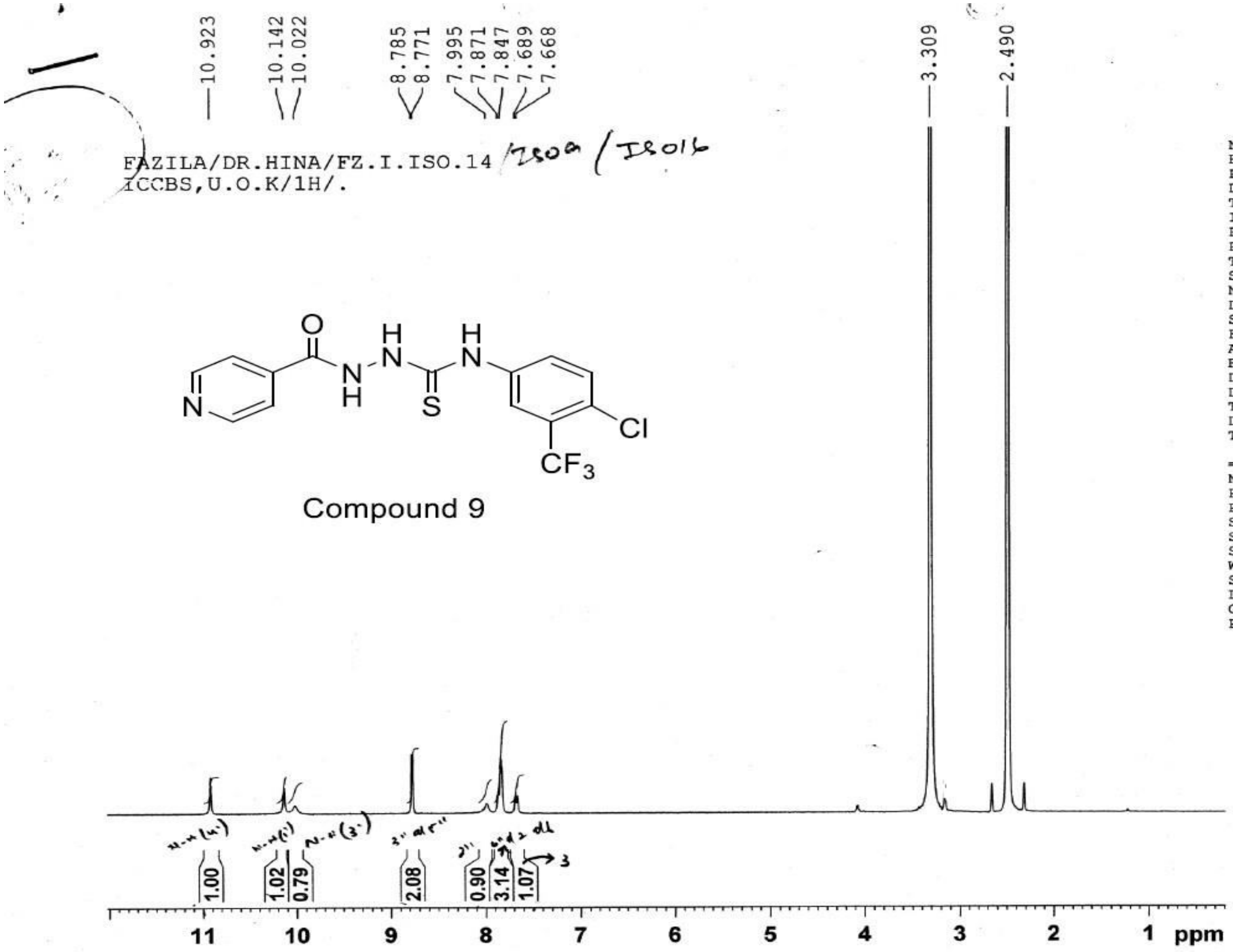

AVANCE AV-400 MHz
Lab # 115

NAME	sep07-16
EXPNO	1
PROCNO	1
Date_	20160907
Time	12.09
INSTRUM	spect
PROBHD	5 mm SEI 1H-13
PULPROG	zg30
TD	65536
SOLVENT	DMSO
NS	128
DS	0
SWH	8012.820 Hz
FIDRES	0.122266 Hz
AQ	4.0894966 sec
RG	322.5
DW	62.400 usec
DE	6.50 usec
TE	300.0 K
D1	1.50000000 sec
TD0	1

======== CHANNEL f1 ========

NUC1	1H
P1	10.80 usec
PL1	3.00 dB
SFO1	400.0332002 MHz
SI	32768
SF	400.0300042 MHz
WDW	EM
SSB	0
LB	0.30 Hz
GB	0
PC	0.20

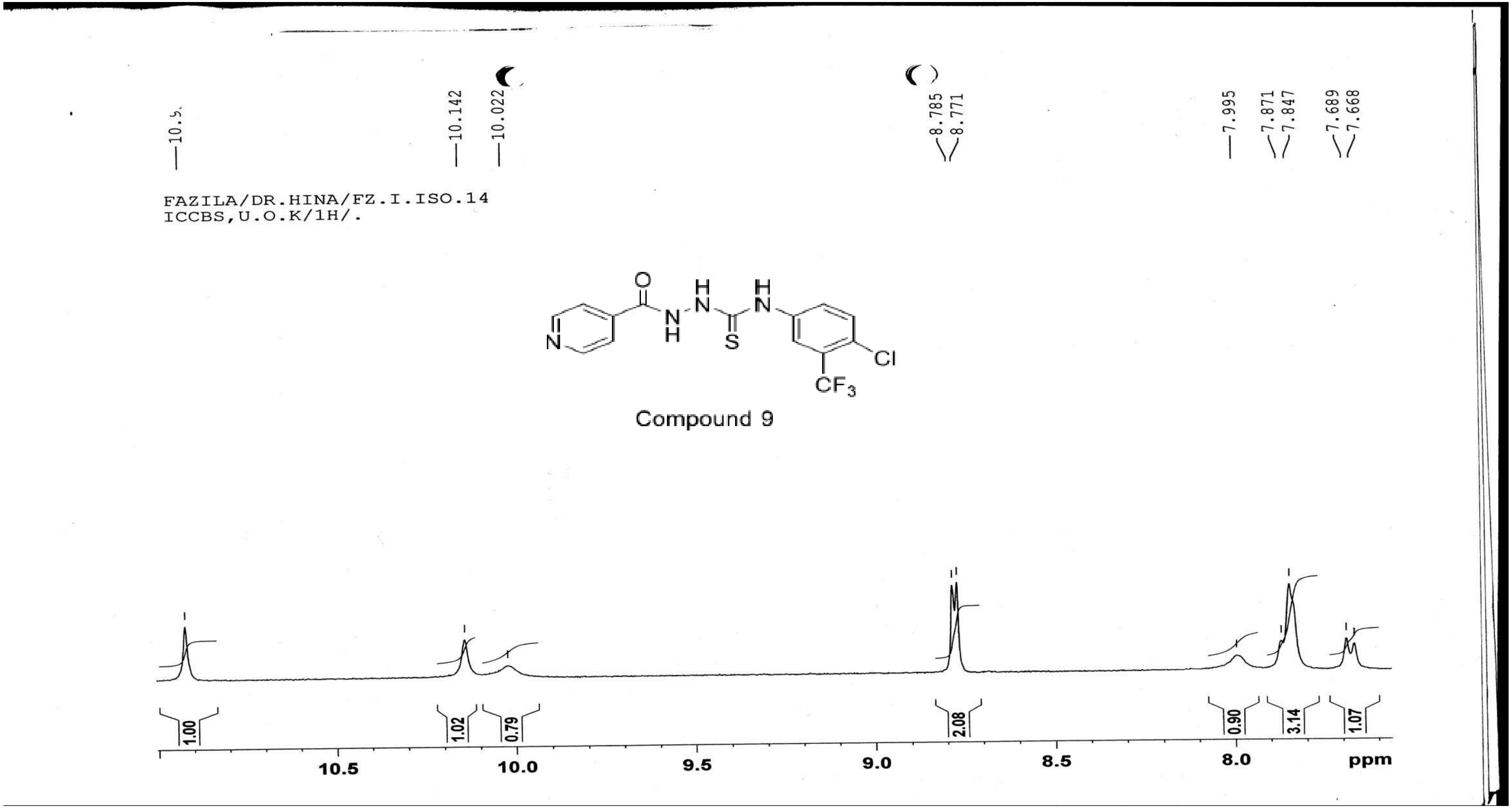

10.142
10.022
8.785
8.771
7.995
7.871
7.847
7.689
7.668
FAZILA/DR.HINA/FZ.I.ISO.14
ICCBS,U.O.K/1H/.
O
H
H
N
N
N
N
H
S
Cl
CF_3
Compound 9
1.00
1.02
0.79
2.08
0.90
3.14
1.07
10.5
10.0
9.5
9.0
8.5
8.0
ppm

HEJ-ICCBS
9/7/2016 3:28:42 PM

File: FZ-I-ISO-14
Sample: FAZILA RIZVI /DR. HINA
Instrument: JEOL-600H-2
Inlet: Direct Probe

Date Run: 09-07-2016 (Time Run: 15:27:11)

Ionization mode: FAB-

Scan: 3
Base: m/z 373; 100%FS TIC: 10685212

R.T.: .18

#Ions: 954

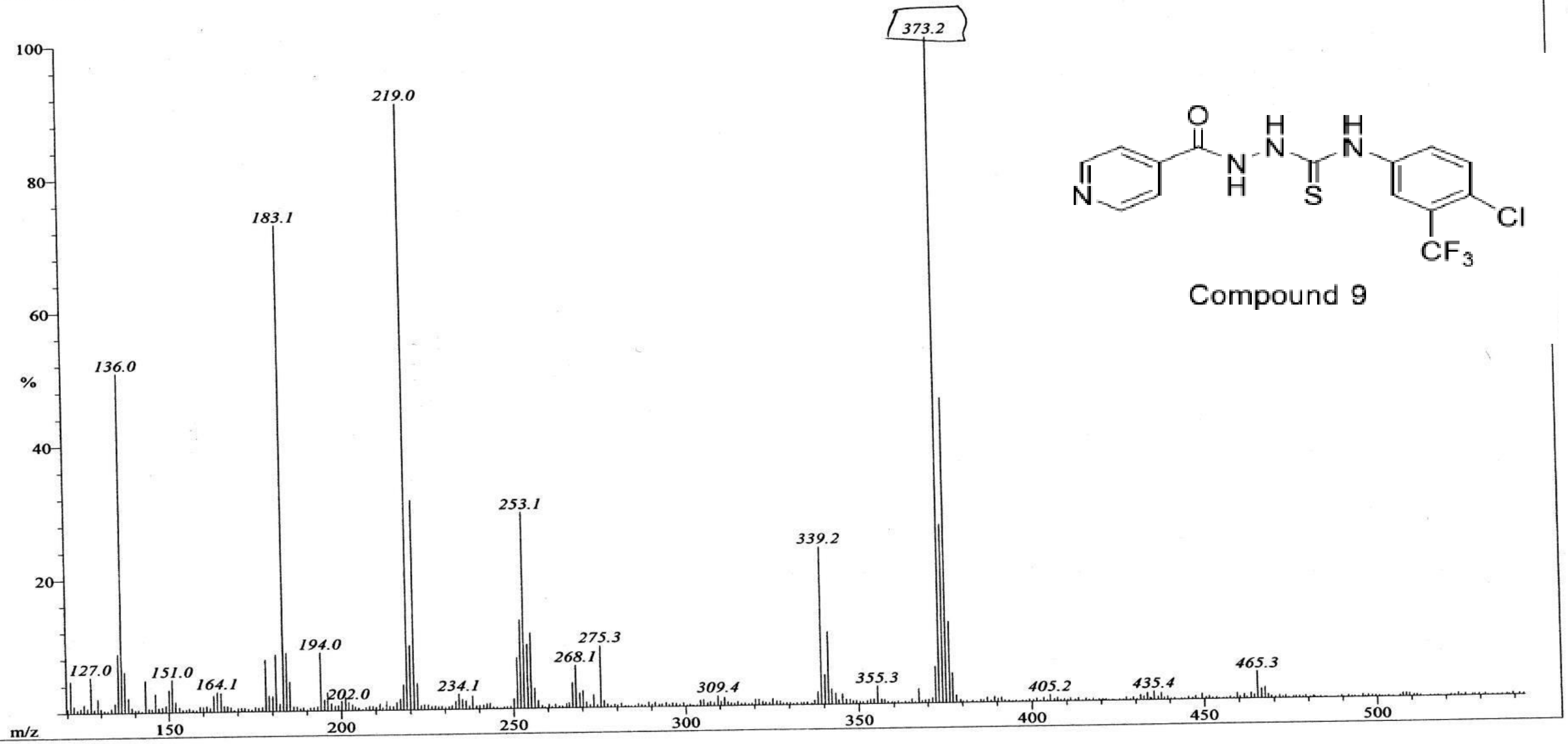

Compound 9

I. C.C.B.S., University of Karacl[illegible]nalytical Laboratory - Pakistan

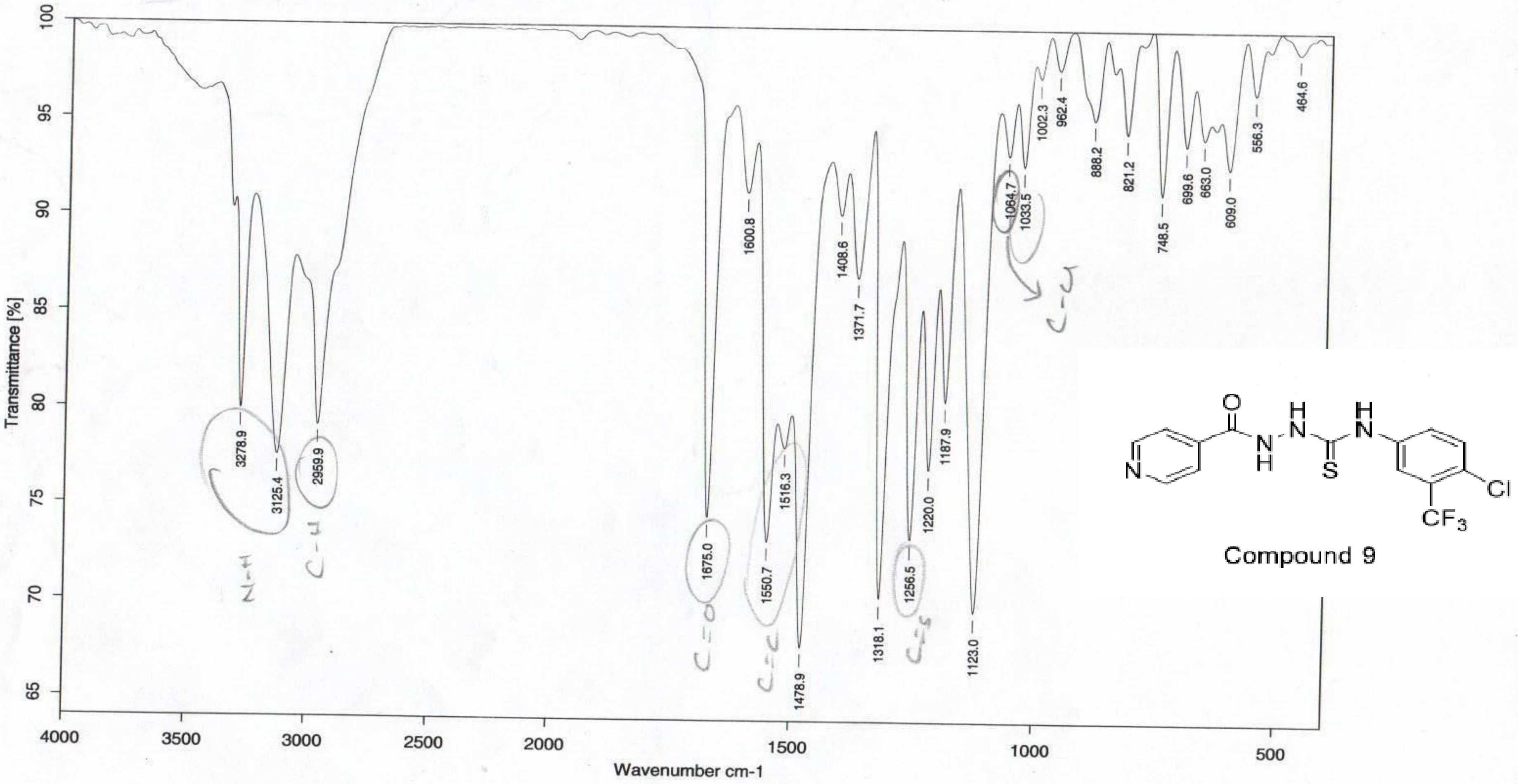

Compound 9

Sample : FZI-ISO14/Fazila Rizvi	Spectrum : FZ-I-ISO14.0 (in D:\IRSTUDENT)
Measured : 19/04/2017 on VECTOR22	Technic : Solid
Resolution : 4 cm-1 (10 scans)	Analyst[illegible] MA/ZA/JS

FAZILA/DR.HINA/FZ-I-1SO14/DMSO
BB

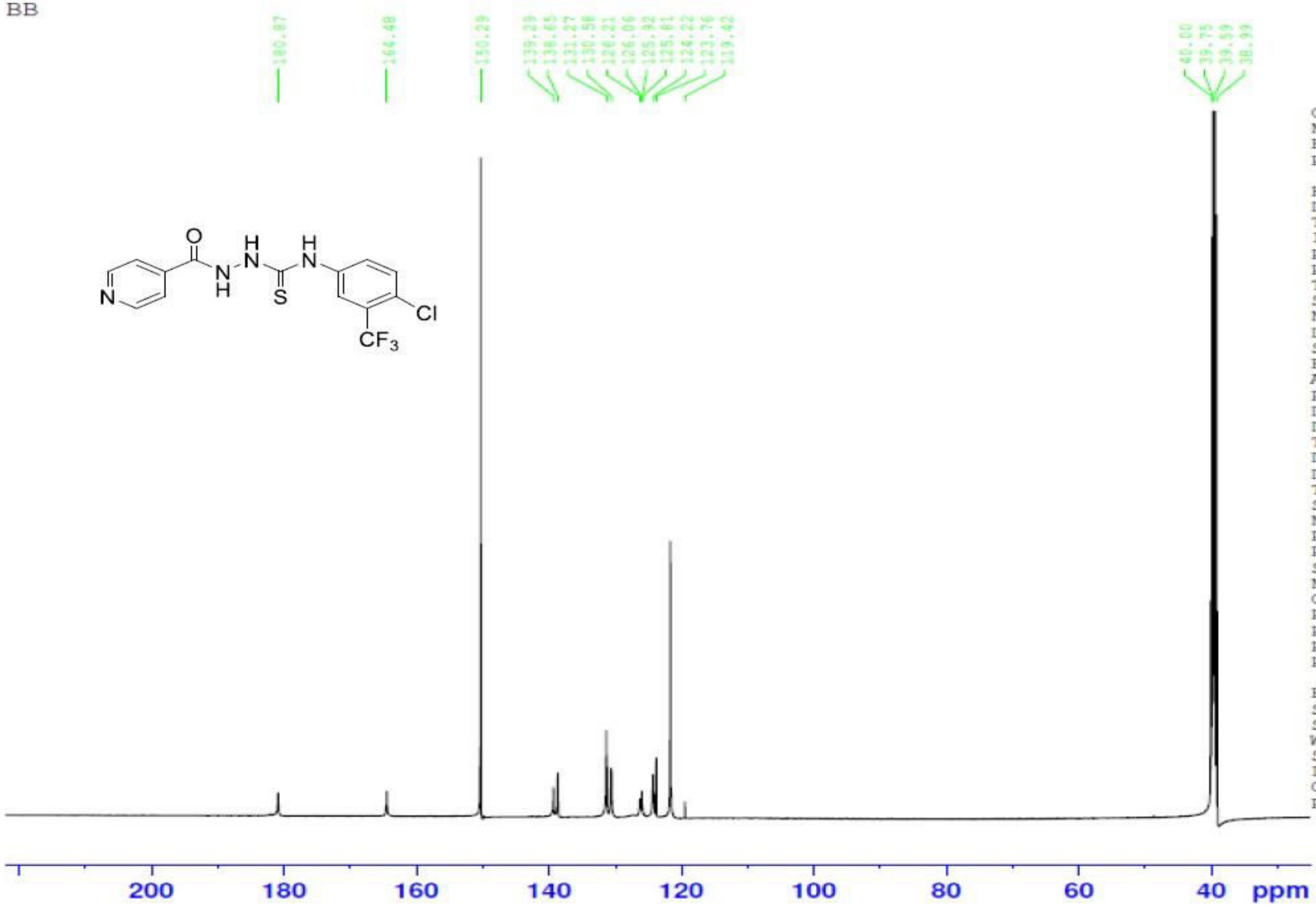

Current Data Parameters
NAME ISO 14 NMR
EXPNO 5
PROCNO 1

F2 - Acquisition Parameters
Date_ 20190205
Time 3.55 h
INSTRUM Avance Neo 500
PROBHD Z44862_0021 (C
PULPROG zgpg
TD 32768
SOLVENT DMSO
NS 16384
DS 8
SWH 30120.482 Hz
FIDRES 1.838408 Hz
AQ 0.5439488 sec
RG 101
DW 16.600 usec
DE 20.00 usec
TE 298.0 K
D1 2.00000000 sec
D11 0.03000000 sec
TD0 16
SFO1 125.8227986 MHz
NUC1 13C
P1 10.00 usec
PLW1 26.46199989 W
SFO2 500.3320013 MHz
NUC2 1H
CPDPRG[2 waltz65
PCPD2 80.00 usec
PLW2 9.74149990 W
PLW12 0.34246999 W
PLW13 0.17199001 W

F2 - Processing parameters
SI 16384
SF 125.8081399 MHz
WDW EM
SSB 0
LB 1.00 Hz
GB 0
PC 1.40

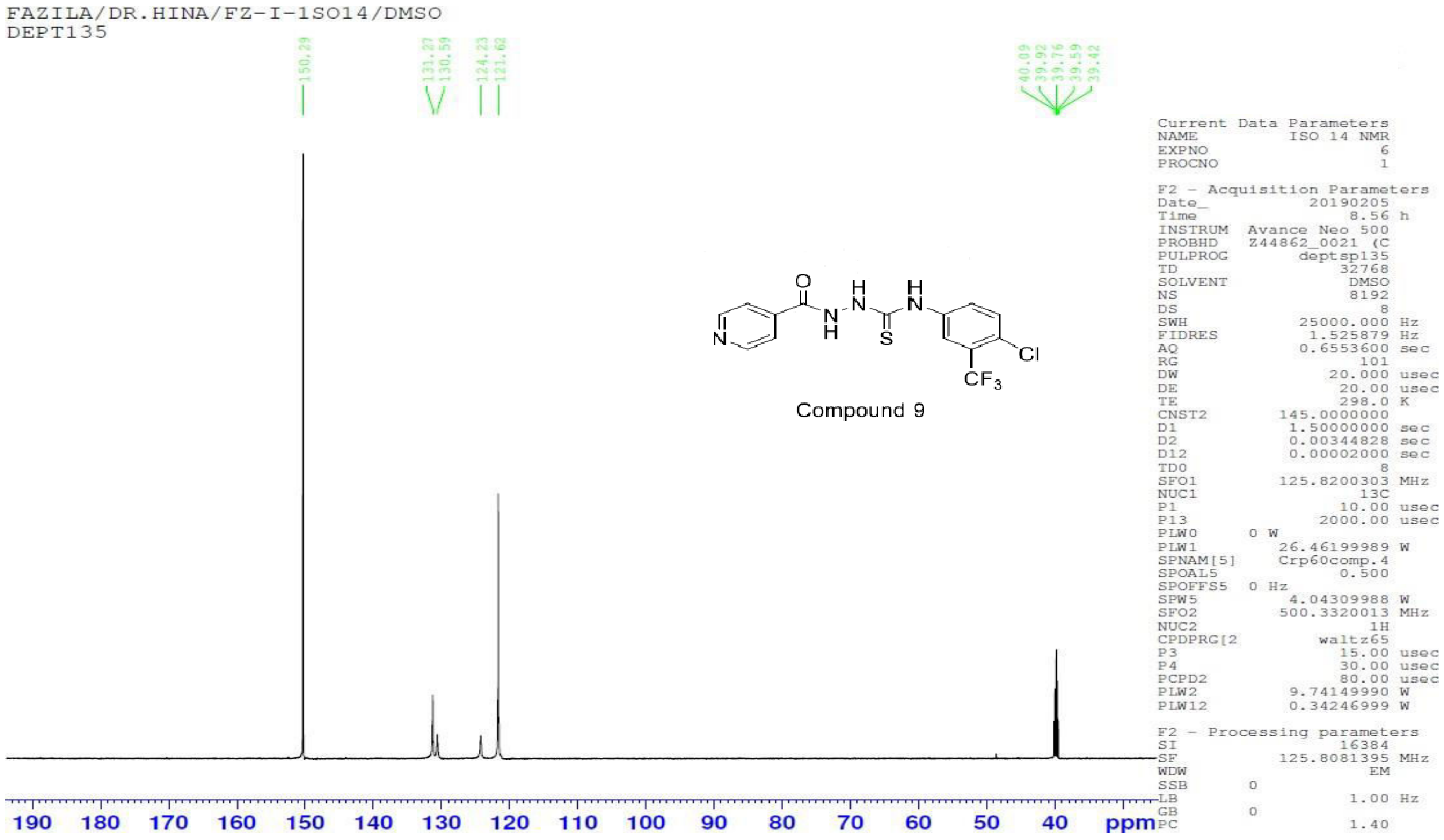
FAZILA/DR.HINA/FZ-I-1SO14/DMSO
DEPT135
150.29
131.27
130.59
124.23
121.62
40.09
39.92
39.76
39.59
39.42
O
H
H
N
N
H
N
H
N
S
Cl
CF3
Compound 9
Current Data Parameters
NAME ISO 14 NMR
EXPNO 6
PROCNO 1
F2 - Acquisition Parameters
Date_ 20190205
Time 8.56 h
INSTRUM Avance Neo 500
PROBHD Z44862_0021 (C
PULPROG deptsp135
TD 32768
SOLVENT DMSO
NS 8192
DS 8
SWH 25000.000 Hz
FIDRES 1.525879 Hz
AQ 0.6553600 sec
RG 101
DW 20.000 usec
DE 20.00 usec
TE 298.0 K
CNST2 145.0000000
D1 1.50000000 sec
D2 0.00344828 sec
D12 0.00002000 sec
TD0 8
SFO1 125.8200303 MHz
NUC1 13C
P1 10.00 usec
P13 2000.00 usec
PLW0 0 W
PLW1 26.46199989 W
SPNAM[5] Crp60comp.4
SPOAL5 0.500
SPOFFS5 0 Hz
SPW5 4.04309988 W
SFO2 500.3320013 MHz
NUC2 1H
CPDPRG[2 waltz65
P3 15.00 usec
P4 30.00 usec
PCPD2 80.00 usec
PLW2 9.74149990 W
PLW12 0.34246999 W
F2 - Processing parameters
SI 16384
SF 125.8081395 MHz
WDW EM
SSB 0
LB 1.00 Hz
GB 0
PC 1.40
190 180 170 160 150 140 130 120 110 100 90 80 70 60 50 40 ppm

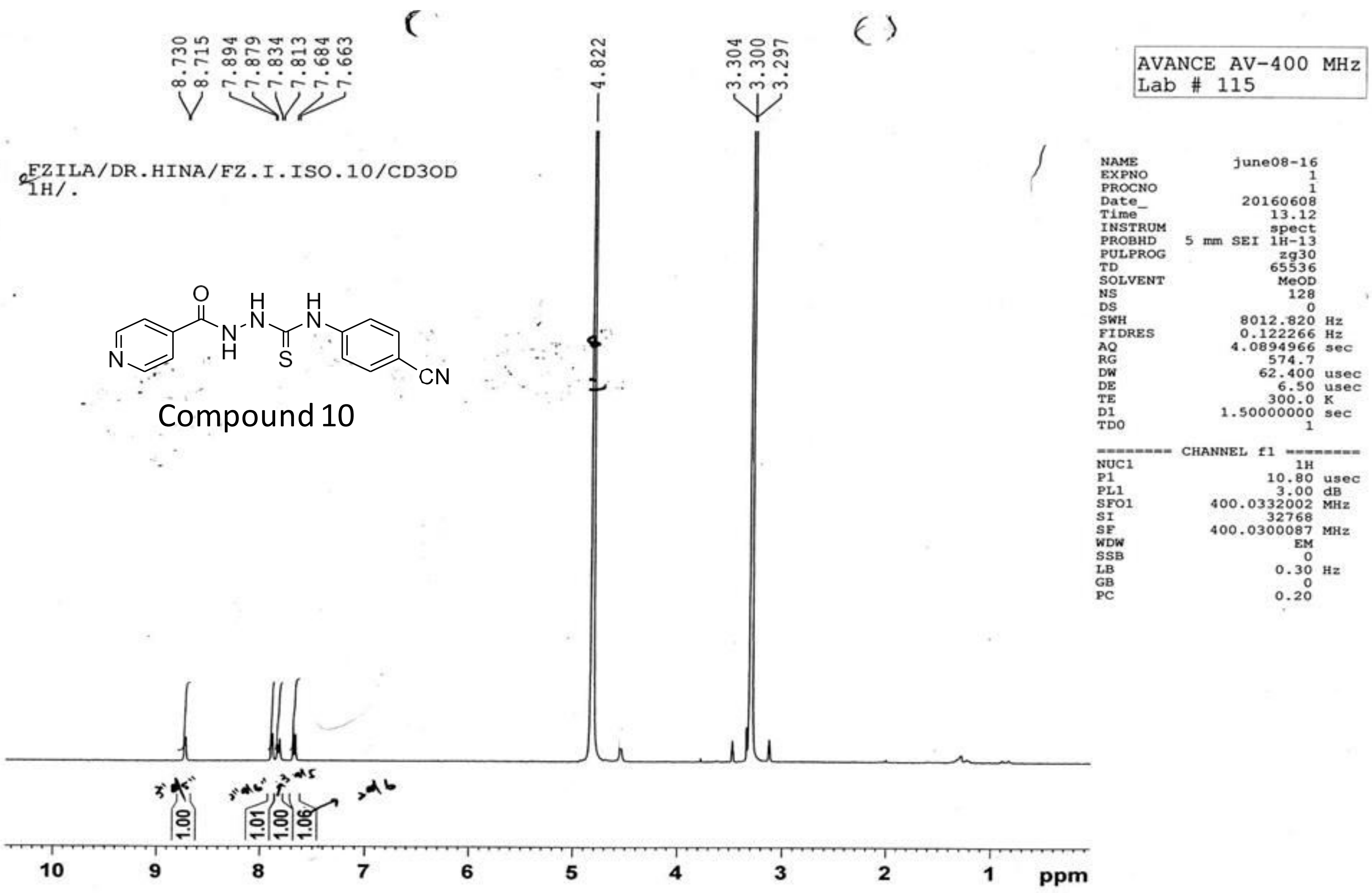
8.730
8.715
7.894
7.879
7.834
7.813
7.684
7.663
4.822
3.304
3.300
3.297
AVANCE AV-400 MHz
Lab # 115
FZILA/DR.HINA/FZ.I.ISO.10/CD3OD
1H/.
O
H
N
H
N
N
H
S
N
CN
Compound 10
NAME june08-16
EXPNO 1
PROCNO 1
Date_ 20160608
Time 13.12
INSTRUM spect
PROBHD 5 mm SEI 1H-13
PULPROG zg30
TD 65536
SOLVENT MeOD
NS 128
DS 0
SWH 8012.820 Hz
FIDRES 0.122266 Hz
AQ 4.0894966 sec
RG 574.7
DW 62.400 usec
DE 6.50 usec
TE 300.0 K
D1 1.50000000 sec
TD0 1
======== CHANNEL f1 ========
NUC1 1H
P1 10.80 usec
PL1 3.00 dB
SFO1 400.0332002 MHz
SI 32768
SF 400.0300087 MHz
WDW EM
SSB 0
LB 0.30 Hz
GB 0
PC 0.20
1.00
1.01
1.00
1.06
10
9
8
7
6
5
4
3
2
1
ppm

FZILA/DR.HINA/FZ.I.ISO.10/CD3OD
1H/.

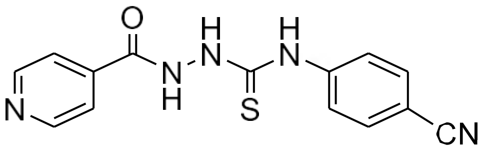

Compound 10

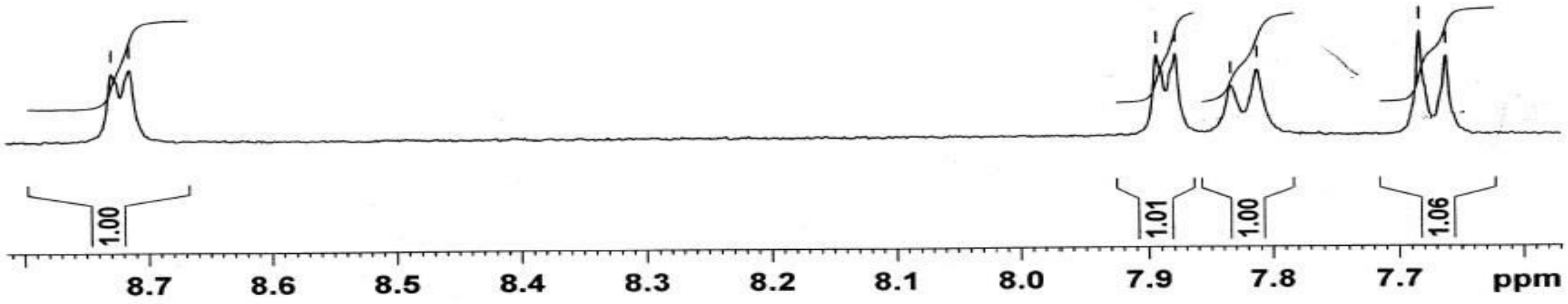

6/9/2016 12:42:48 PM Page 1

File: FZ-I-ISO10-FABP
Sample: FAZILA /DR. HINA
Instrument: JEOL-600H-2
Inlet: Direct Probe

Date Run: 06-09-2016 (Time Run: 12:40:22)

Ionization mode: FAB+

Scan: 11 R.T.: .88
Base: m/z 185; 36.5%FS TIC: 1004100 #Ions: 542

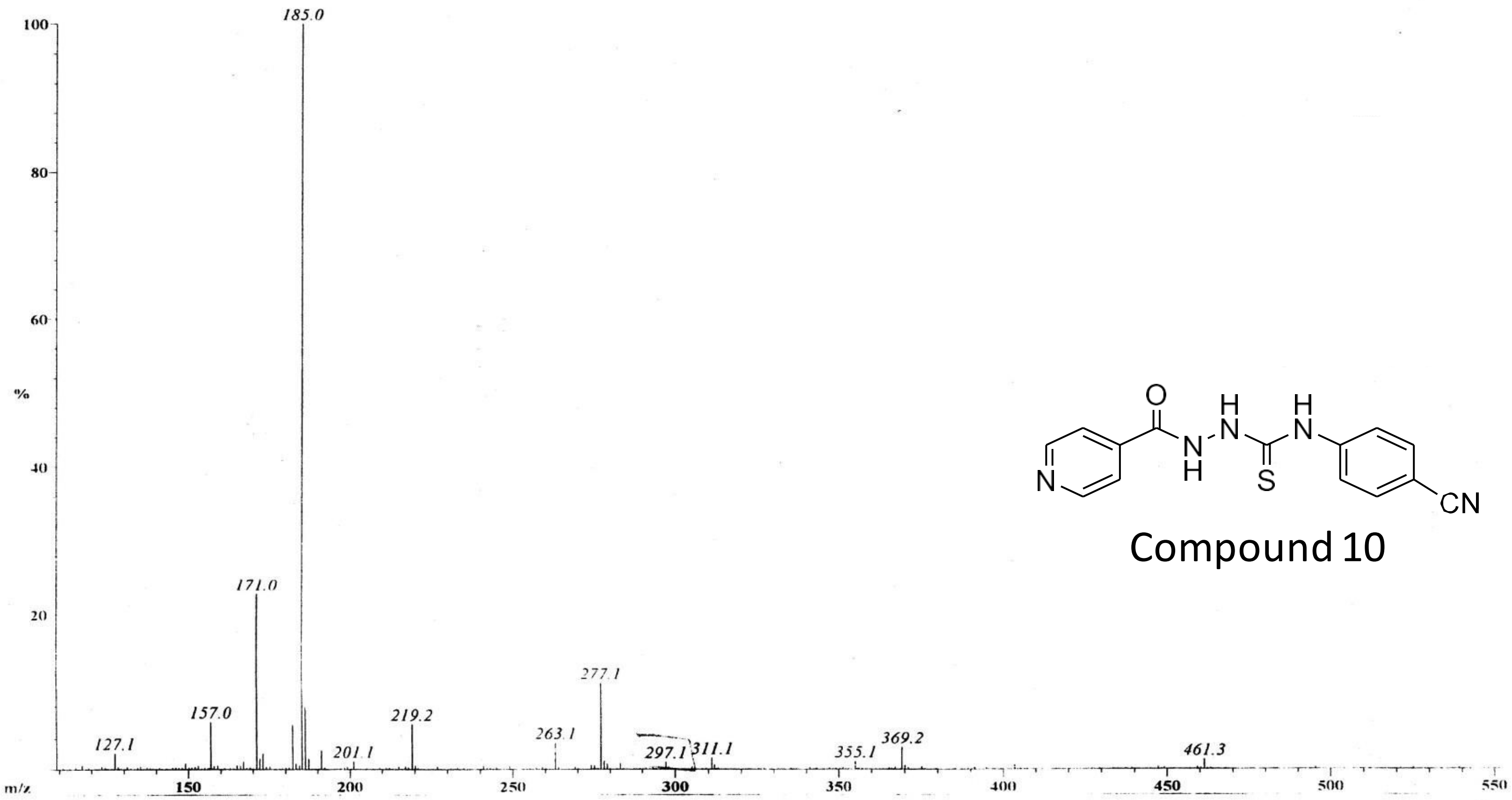

Compound 10

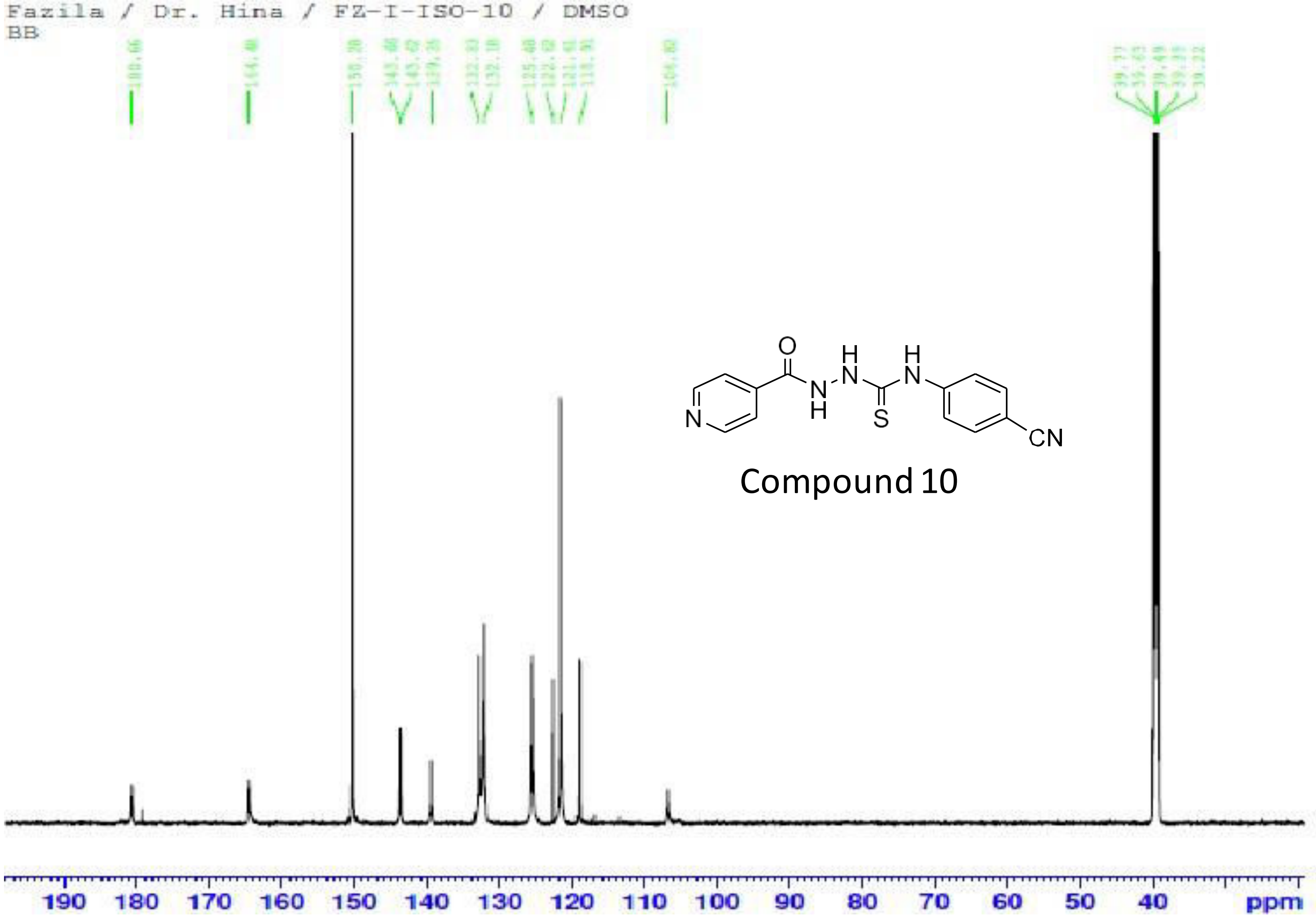

Current Data Parameters		
NAME	ISO 10	
EXPNO	4	
PROCNO	1	
**F2 - Acquisition Parameters**		
Date_	20190207	
Time	14.14	h
INSTRUM	AVNeo_600	
PROBHD	Z117768_0039 (	
PULPROG	zgpg	
TD	32768	
SOLVENT	DMSO	
NS	6606	
DS	4	
SWH	35714.285	Hz
FIDRES	2.179827	Hz
AQ	0.4587520	sec
RG	101	
DW	14.000	usec
DE	18.00	usec
TE	298.0	K
D1	1.50000000	sec
D11	0.03000000	sec
TD0	8	
SFO1	150.9553694	MHz
NUC1	13C	
P1	12.00	usec
PLW1	107.76000214	W
SFO2	600.2724011	MHz
NUC2	1H	
CPDPRG[2	waltz65	
PCPD2	70.00	usec
PLW2	9.53950024	W
PLW12	0.12460000	W
PLW13	0.06267200	W
**F2 - Processing parameters**		
SI	16384	
SF	150.9380853	MHz
WDW	EM	
SSB	0	
LB	1.00	Hz
GB	0	
PC	1.40	

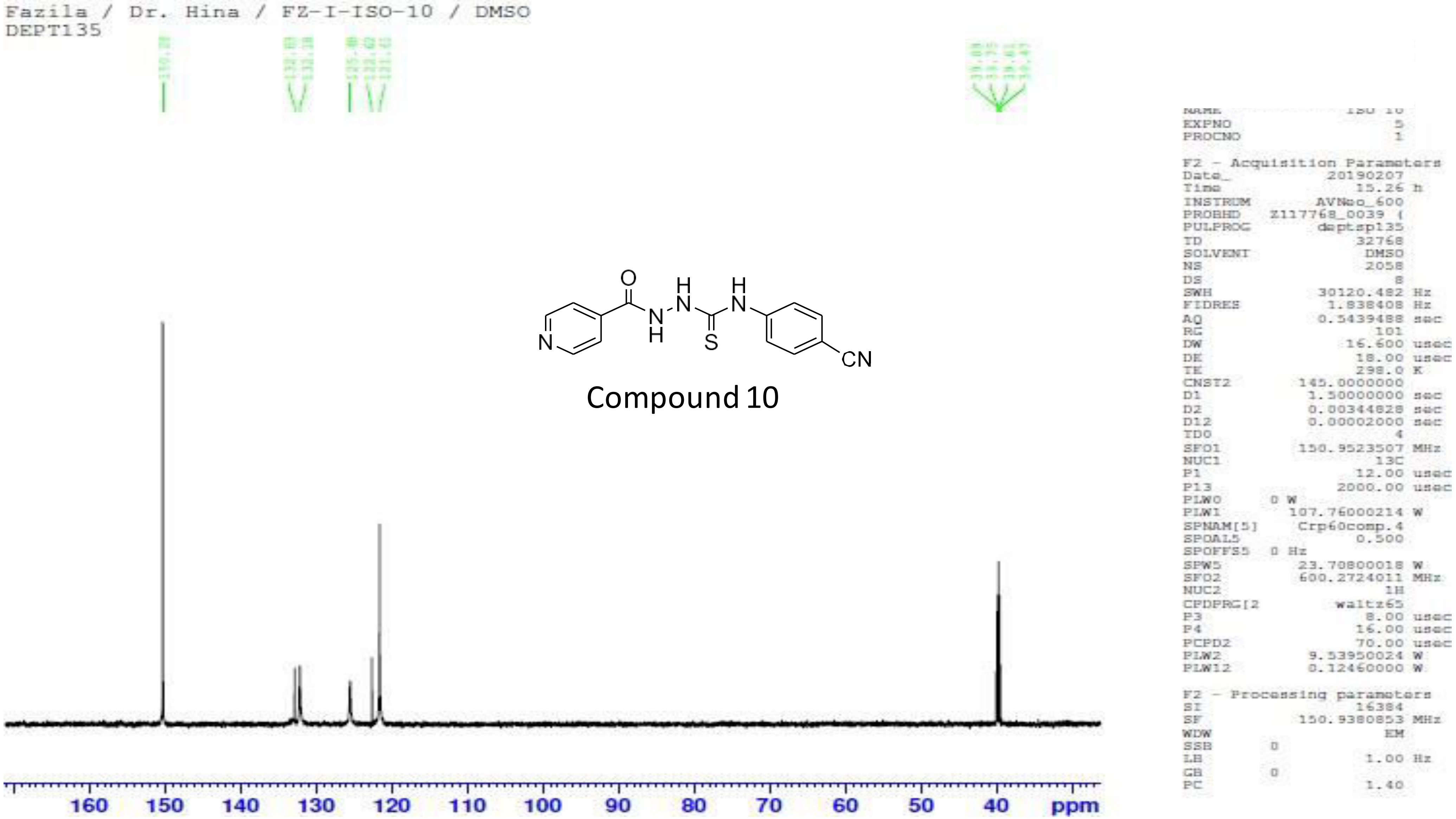
Fazila / Dr. Hina / FZ-I-ISO-10 / DMSO
DEPT135
160 150 140 130 120 110 100 90 80 70 60 50 40 ppm
NAME ISO 10
EXPNO 5
PROCNO 1
F2 - Acquisition Parameters
Date_ 20190207
Time 15.26 h
INSTRUM AVNeo_600
PROBHD Z117768_0039 (
PULPROG deptsp135
TD 32768
SOLVENT DMSO
NS 2058
DS 8
SWH 30120.482 Hz
FIDRES 1.838408 Hz
AQ 0.5439488 sec
RG 101
DW 16.600 usec
DE 18.00 usec
TE 298.0 K
CNST2 145.0000000
D1 1.50000000 sec
D2 0.00344828 sec
D12 0.00002000 sec
TD0 4
SFO1 150.9523507 MHz
NUC1 13C
P1 12.00 usec
P13 2000.00 usec
PLW0 0 W
PLW1 107.76000214 W
SPNAM[5] Crp60comp.4
SPOAL5 0.500
SPOFFS5 0 Hz
SPW5 23.70800018 W
SFO2 600.2724011 MHz
NUC2 1H
CPDPRG[2 waltz65
P3 8.00 usec
P4 16.00 usec
PCPD2 70.00 usec
PLW2 9.53950024 W
PLW12 0.12460000 W
F2 - Processing parameters
SI 16384
SF 150.9380853 MHz
WDW EM
SSB 0
LB 1.00 Hz
GB 0
PC 1.40
O
H
N
N
H
H
N
S
N
CN
Compound 10

I. C.C.B.S., University of Ka[illegible]chi Analytical Laboratory - Pakistan

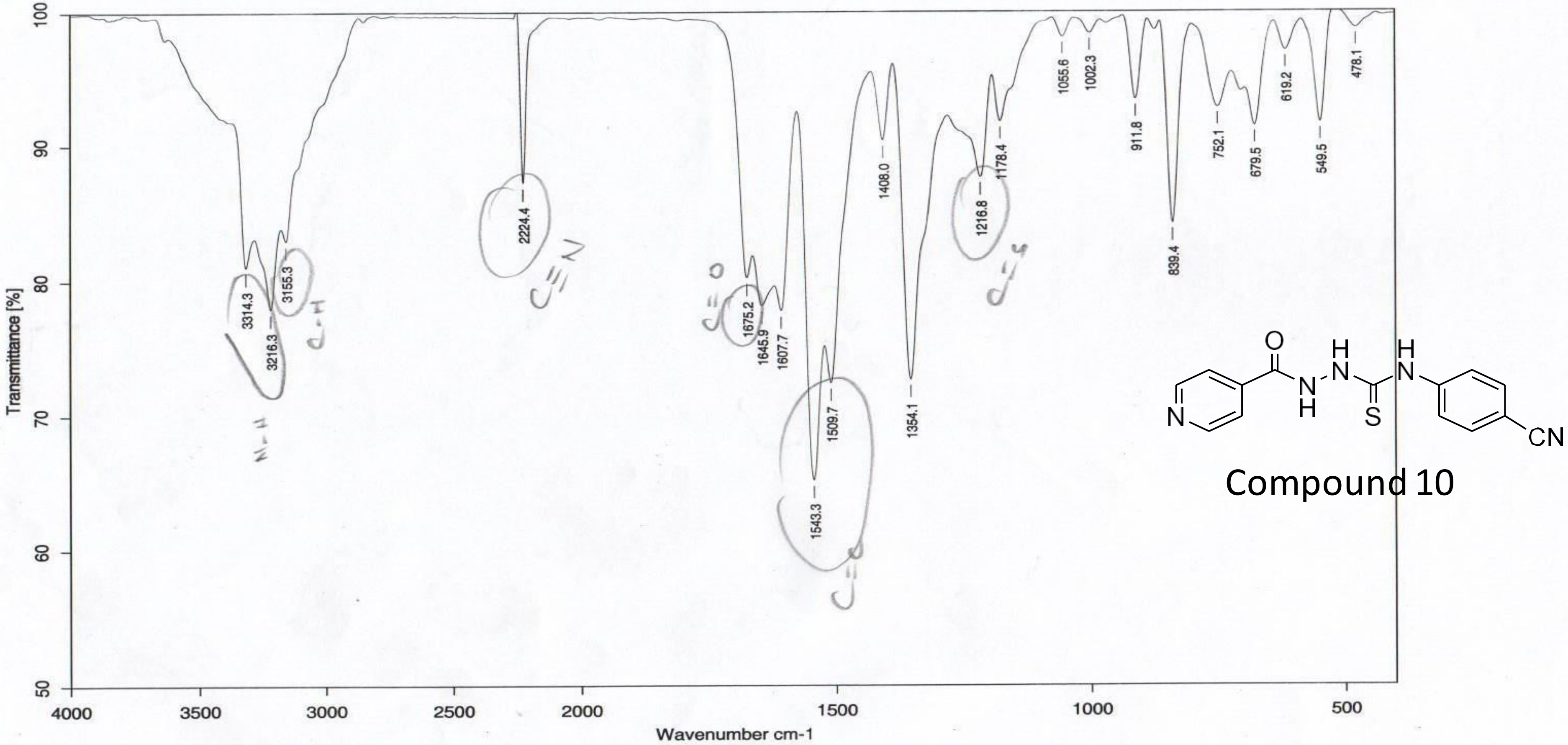

Compound 10

Sample :	FZI-ISO10/Fazila Rizvi	Spectrum	: FZ-I-ISO10.0 (in D:\IRSTUDENT)
Measured :	19/04/2017 on VECTOR22	Technic	: Solid
Resolution :	4 cm-1 (10 scans)	Analyst	: MA/ZA/JS

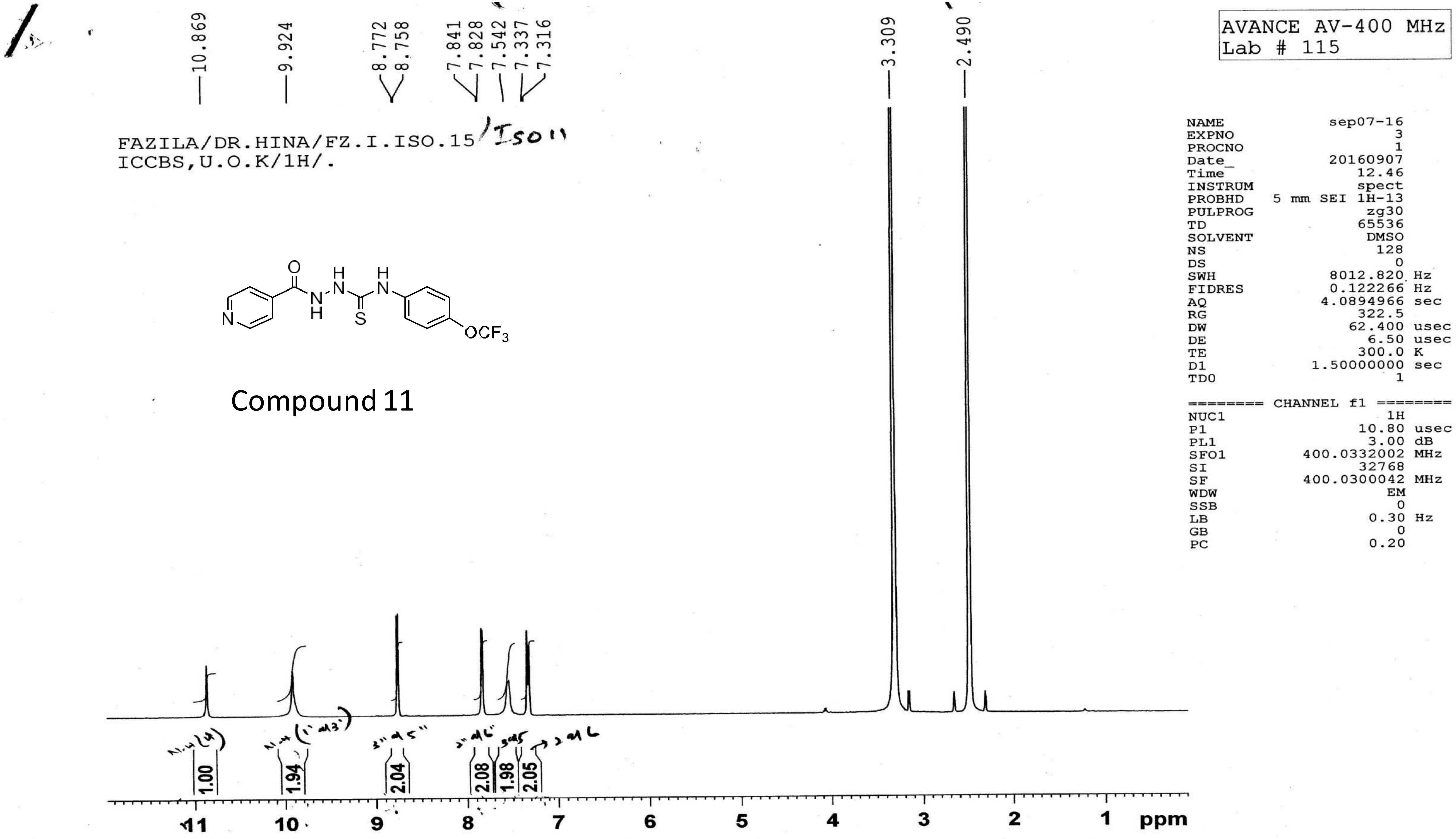

10.869
9.924
8.772
8.758
7.841
7.828
7.542
7.337
7.316
3.309
2.490
AVANCE AV-400 MHz
Lab # 115
FAZILA/DR.HINA/FZ.I.ISO.15/ISO 11
ICCBS,U.O.K/1H/.
O
H
H
N
N
N
H
S
OCF3
Compound 11
NAME sep07-16
EXPNO 3
PROCNO 1
Date_ 20160907
Time 12.46
INSTRUM spect
PROBHD 5 mm SEI 1H-13
PULPROG zg30
TD 65536
SOLVENT DMSO
NS 128
DS 0
SWH 8012.820 Hz
FIDRES 0.122266 Hz
AQ 4.0894966 sec
RG 322.5
DW 62.400 usec
DE 6.50 usec
TE 300.0 K
D1 1.50000000 sec
TD0 1
======== CHANNEL f1 ========
NUC1 1H
P1 10.80 usec
PL1 3.00 dB
SFO1 400.0332002 MHz
SI 32768
SF 400.0300042 MHz
WDW EM
SSB 0
LB 0.30 Hz
GB 0
PC 0.20
1.00
1.94
2.04
2.08
1.98
2.05
11
10
9
8
7
6
5
4
3
2
1
ppm

FAZILA/DR.HINA/FZ.I.ISO.15
ICCBS,U.O.K/1H/.

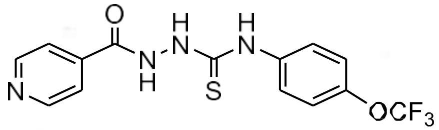

Compound 11

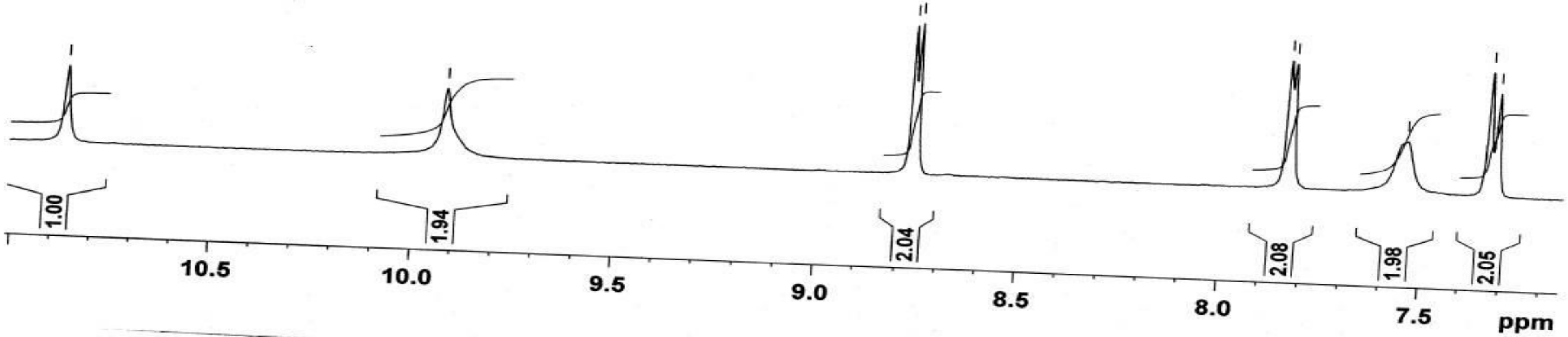

HEJ-ICCBS
9/7/2016 2:55:13 PM

File: FZ-I-ISO15/Iso 11
Sample: FAZILA RIZVI /DR. HINA
Instrument: JEOL-600H-2
Inlet: Direct Probe

Date Run: 09-07-2016 (Time Run: 14:52:37)

Ionization mode: FAB+

Scan: 4
Base: m/z 185; 54.4%FS TIC: 3045914

R.T.: .27

#Ions: 243

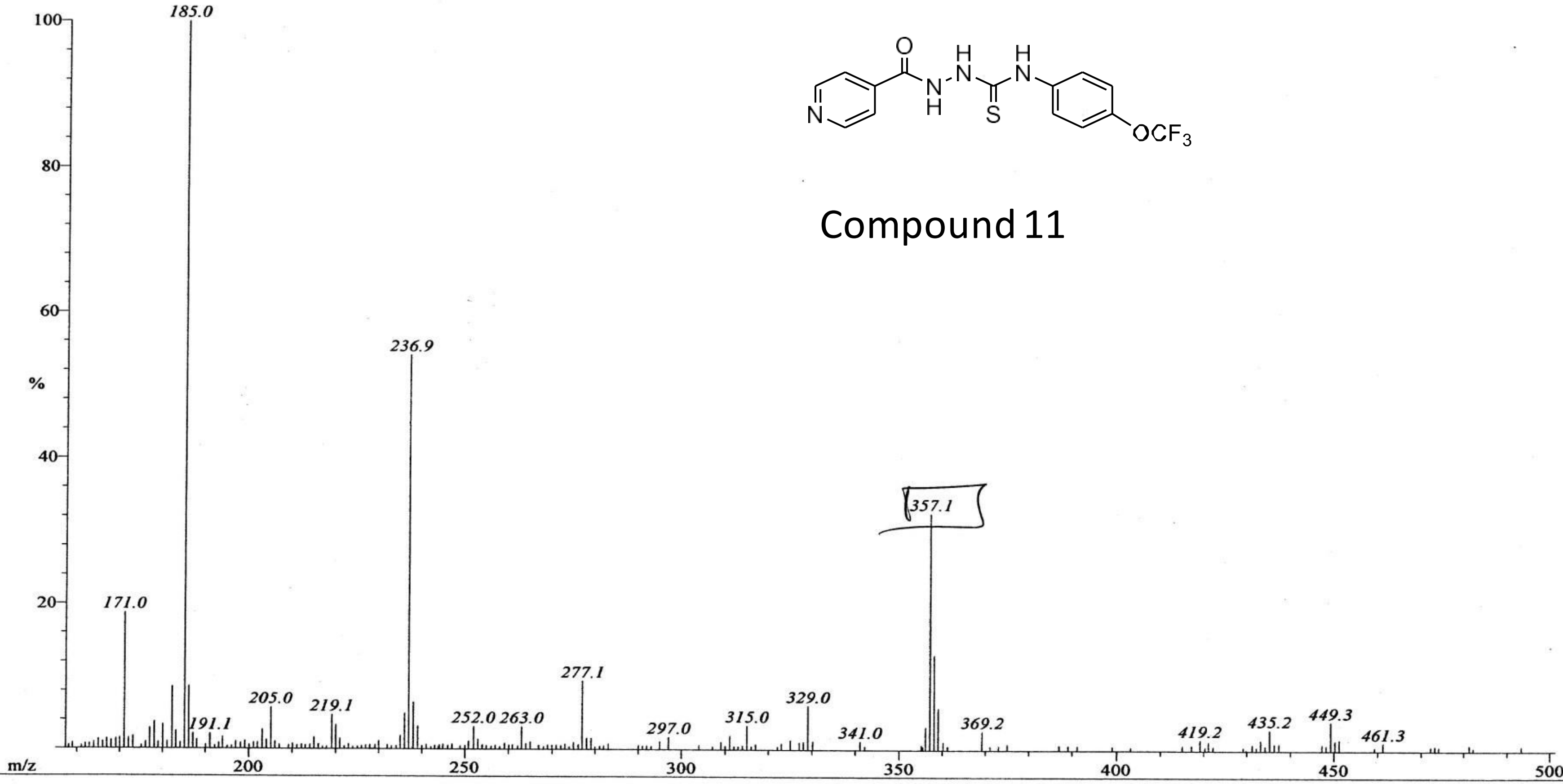

Compound 11

I. C.C.B.S., University of Kar[illegible]hi Analytical Laboratory - Pakistan

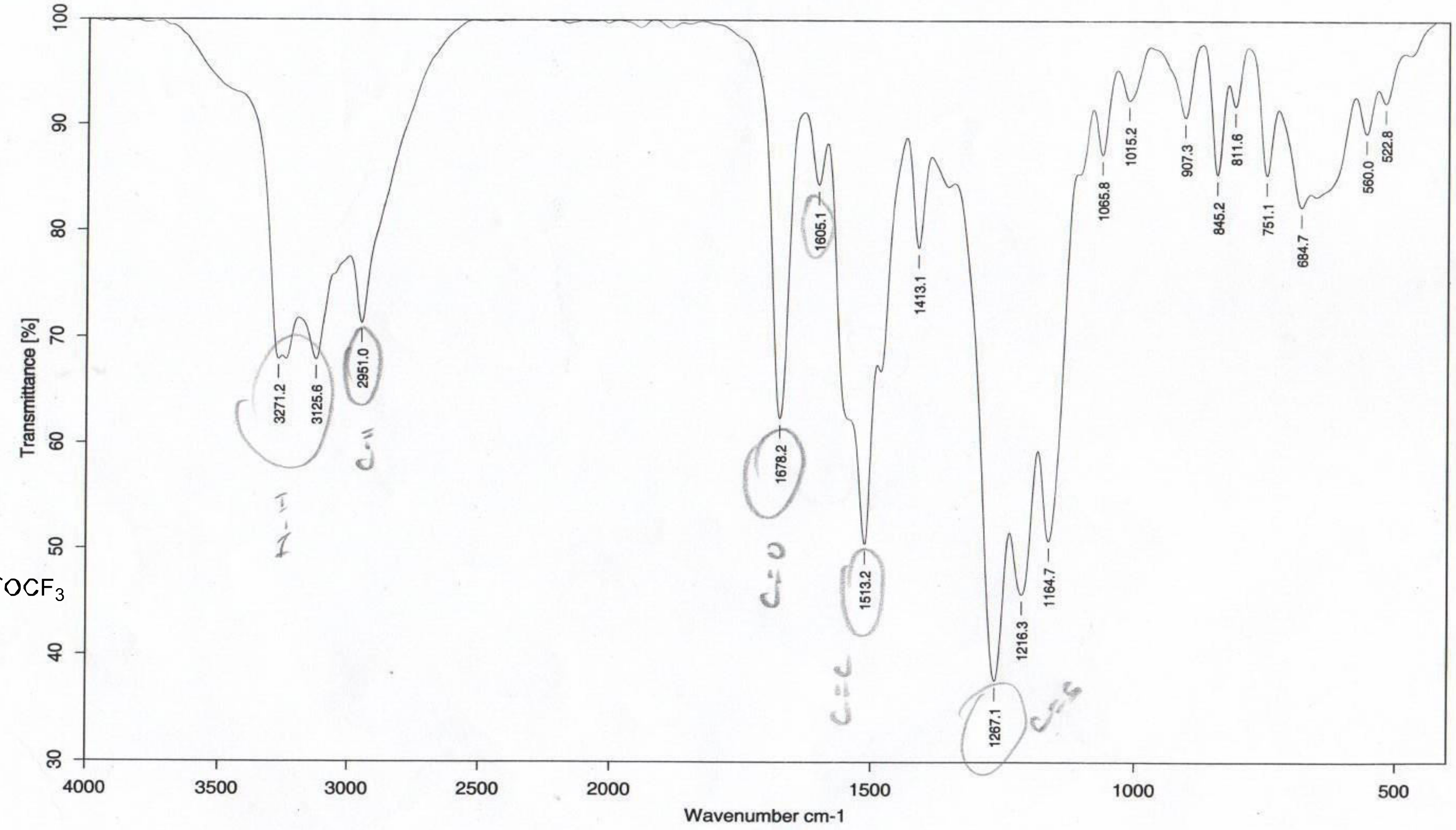

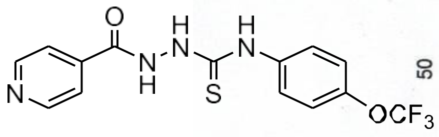

Compound 11

Sample : FZI-ISO11/Fazila Rizvi	Spectrum : FZ-I-ISO11.0 (in D:\IRSTUDENT)
Measured : 19/04/2017 on VECTOR22	Technic : Solid
Resolution : 4 cm-1 (10 scans)	Analyst : MA/ZA/JS

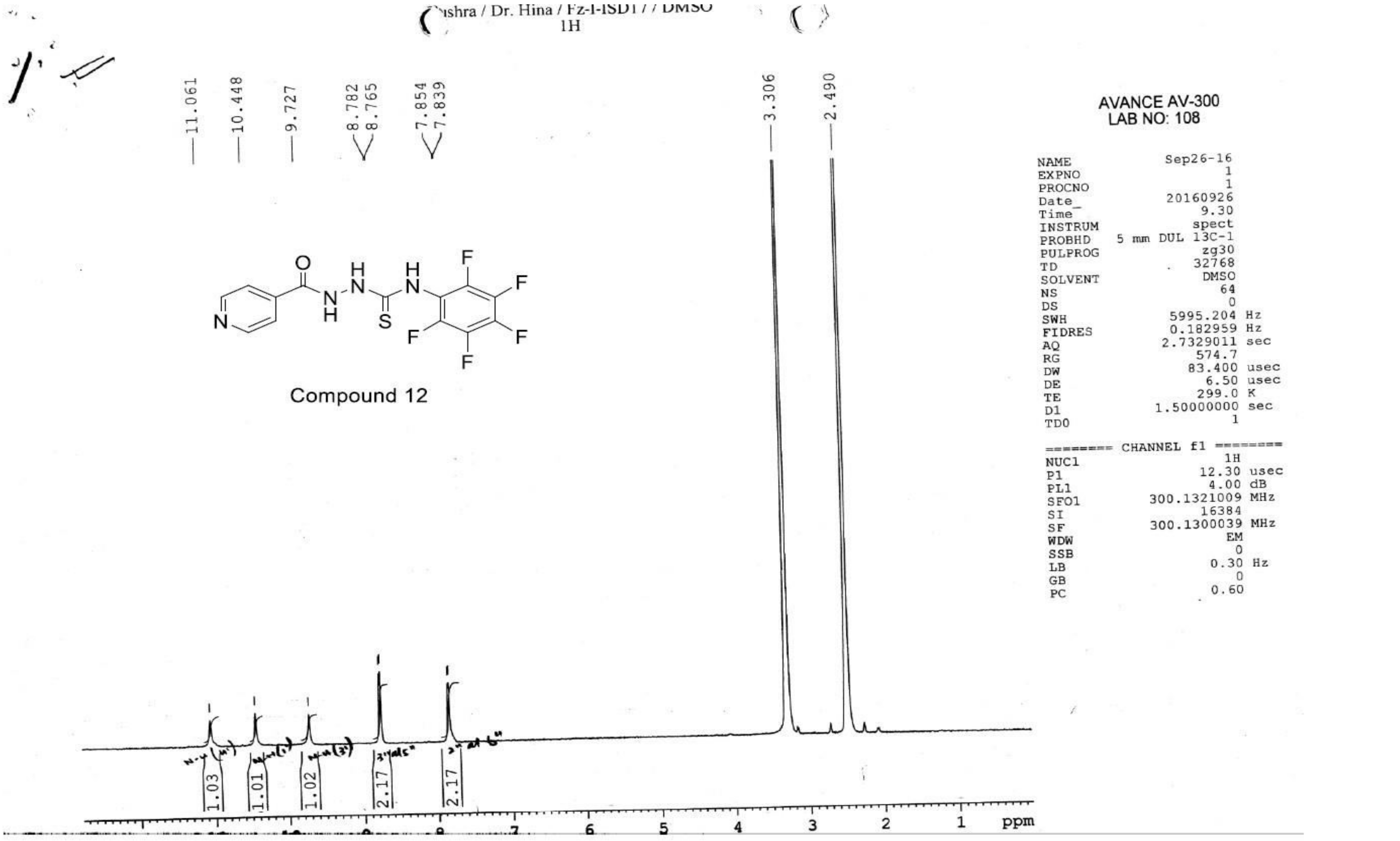

ishra / Dr. Hina / Fz-I-ISD1 / / DMSO
1H
11.061
10.448
9.727
8.782
8.765
7.854
7.839
3.306
2.490
Compound 12
1.03
1.01
1.02
2.17
2.17
ppm
AVANCE AV-300
LAB NO: 108
NAME Sep26-16
EXPNO 1
PROCNO 1
Date_ 20160926
Time 9.30
INSTRUM spect
PROBHD 5 mm DUL 13C-1
PULPROG zg30
TD 32768
SOLVENT DMSO
NS 64
DS 0
SWH 5995.204 Hz
FIDRES 0.182959 Hz
AQ 2.7329011 sec
RG 574.7
DW 83.400 usec
DE 6.50 usec
TE 299.0 K
D1 1.50000000 sec
TD0 1
======== CHANNEL f1 ========
NUC1 1H
P1 12.30 usec
PL1 4.00 dB
SFO1 300.1321009 MHz
SI 16384
SF 300.1300039 MHz
WDW EM
SSB 0
LB 0.30 Hz
GB 0
PC 0.60

shra / Dr. Hina / Fz-I-ISD17 / DMSO
1H

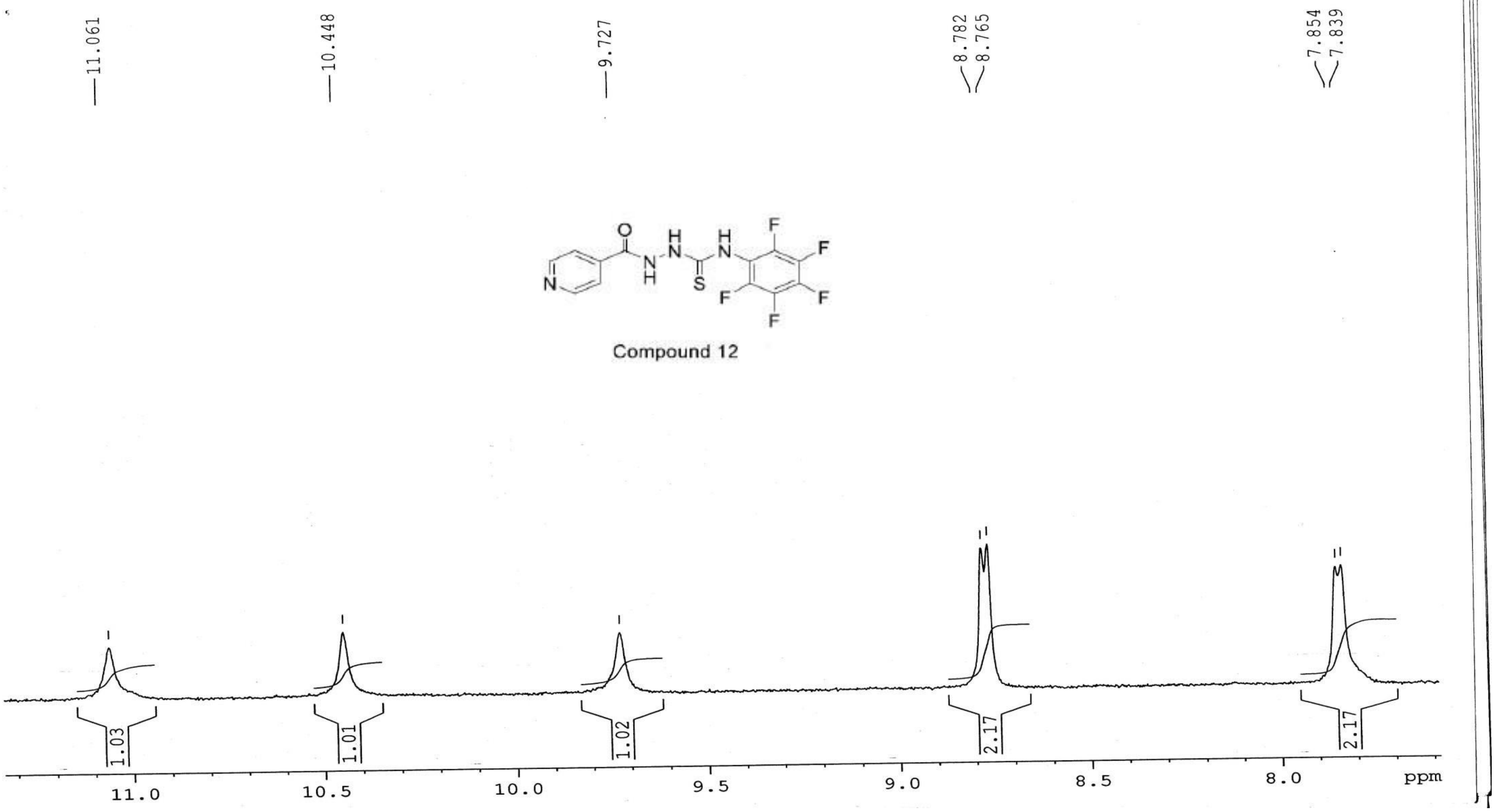

Compound 12

HEJ-ICCBS
10/4/2016 3:39:29 PM

File: FZ-I-ISO-17
Sample: FAZILA RIZVI /DR. HINA
Instrument: JEOL-600H-2
Inlet: Direct Probe

Date Run: 10-04-2016 (Time Run: 15:38:00)

Ionization mode: FAB-

Scan: 1
Base: m/z 183; 43.3%FS TIC: 3670516

R.T.: 0

#Ions: 774

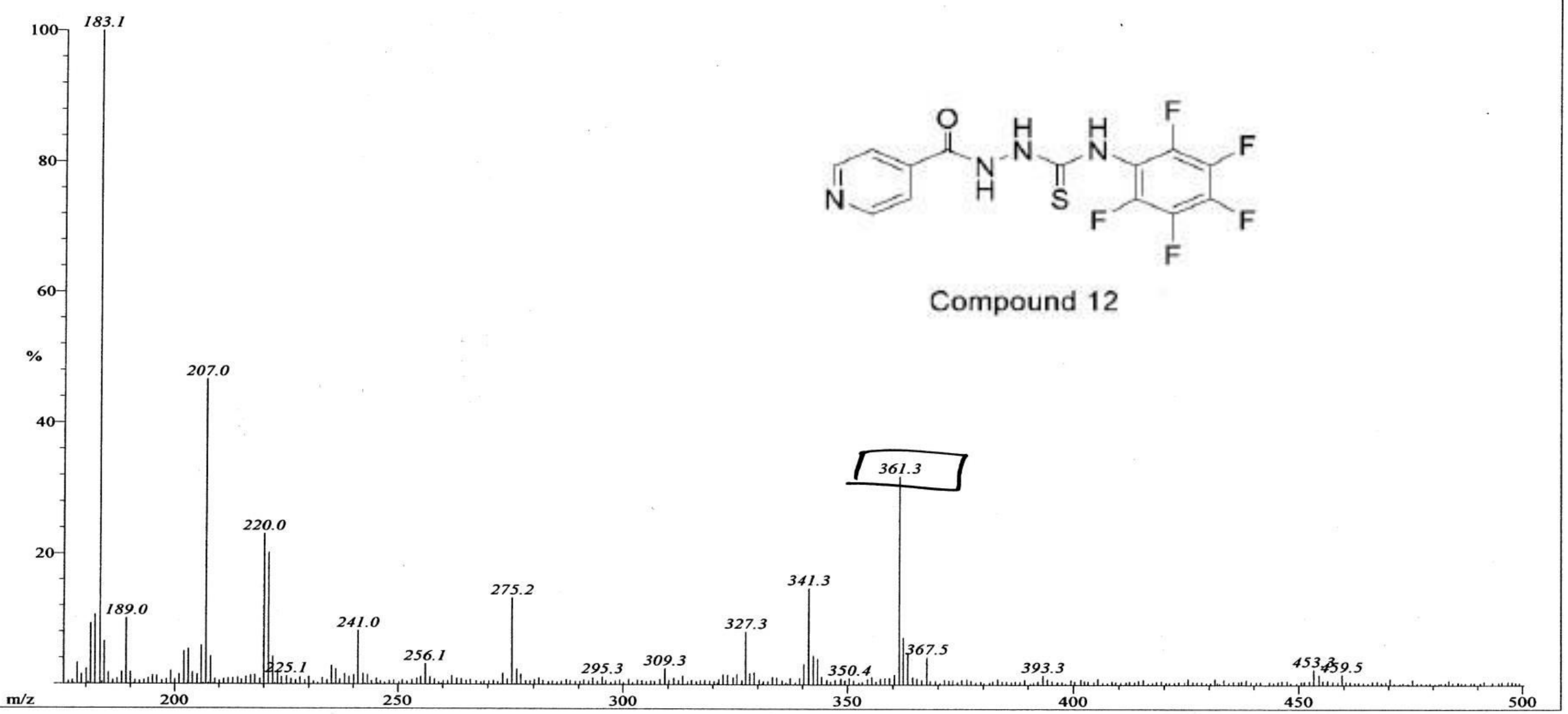

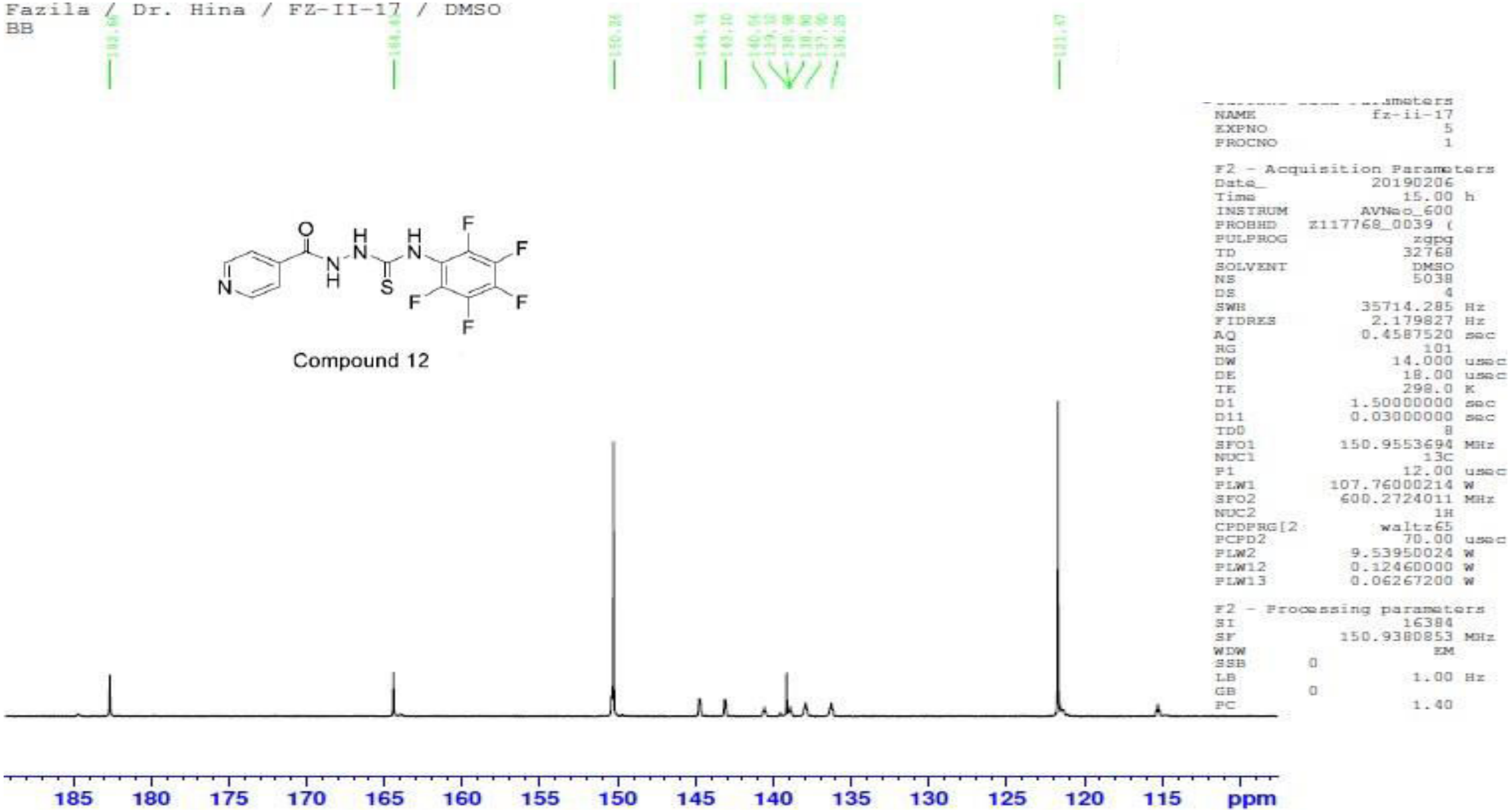

Fazila / Dr. Hina / FZ-II-17 / DMSO
BB
NAME fz-ii-17
EXPNO 5
PROCNO 1
F2 - Acquisition Parameters
Date_ 20190206
Time 15.00 h
INSTRUM AVNeo_600
PROBHD Z117768_0039 (
PULPROG zgpg
TD 32768
SOLVENT DMSO
NS 5038
DS 4
SWH 35714.285 Hz
FIDRES 2.179827 Hz
AQ 0.4587520 sec
RG 101
DW 14.000 usec
DE 18.00 usec
TE 298.0 K
D1 1.50000000 sec
D11 0.03000000 sec
TD0 8
SFO1 150.9553694 MHz
NUC1 13C
P1 12.00 usec
PLW1 107.76000214 W
SFO2 600.2724011 MHz
NUC2 1H
CPDPRG[2 waltz65
PCPD2 70.00 usec
PLW2 9.53950024 W
PLW12 0.12460000 W
PLW13 0.06267200 W
F2 - Processing parameters
SI 16384
SF 150.9380853 MHz
WDW EM
SSB 0
LB 1.00 Hz
GB 0
PC 1.40
185 180 175 170 165 160 155 150 145 140 135 130 125 120 115 ppm

Compound 12

Fazila / Dr. Hina / FZ-II-17 / DMSO
DEPT135

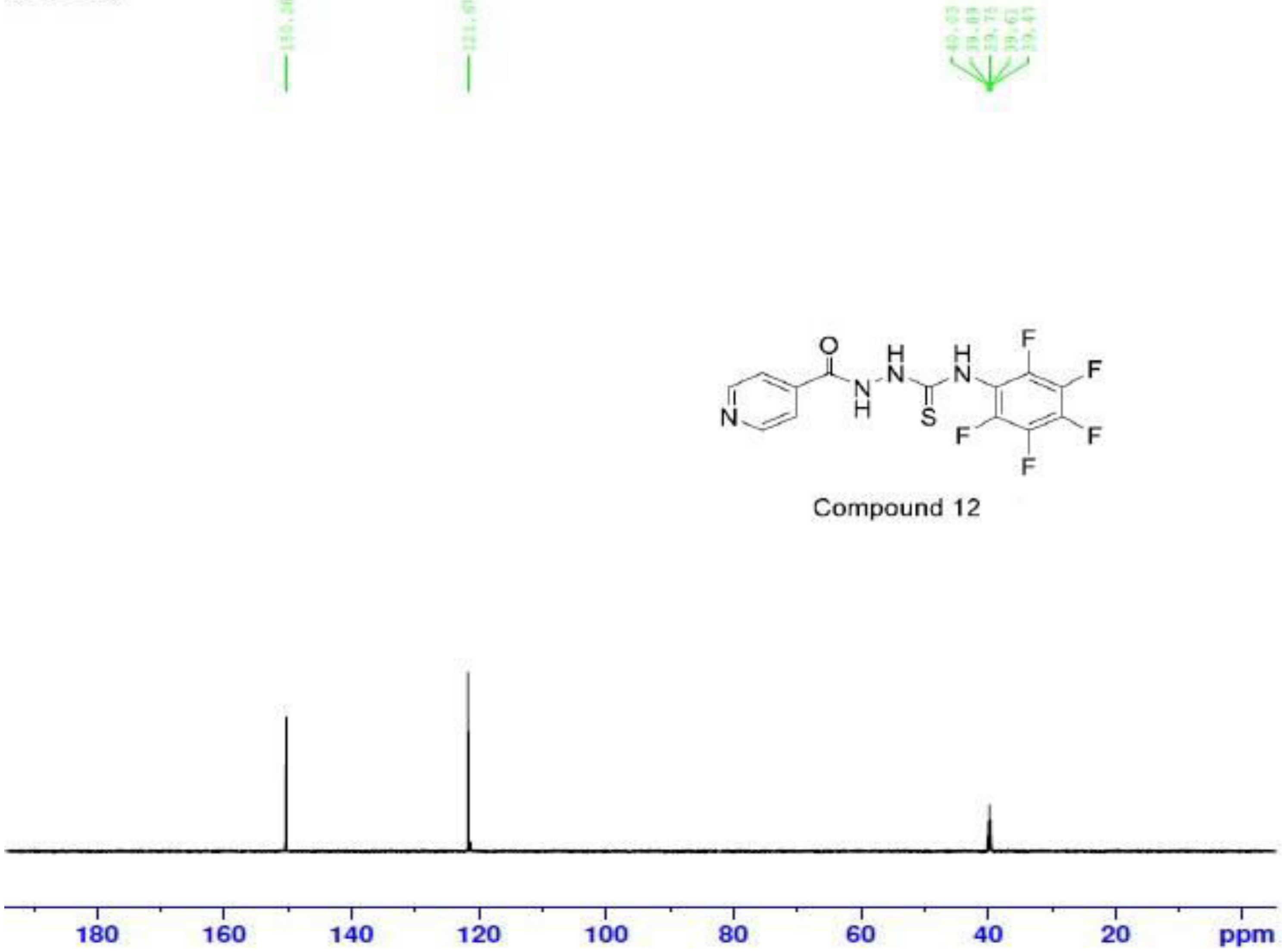

Compound 12

```
EXPNO                   6
PROCNO                  1

F2 - Acquisition Parameters
Date_            20190206
Time                15.33 h
INSTRUM        AVNeo_600
PROBHD   Z117768_0039 (
PULPROG        deptsp135
TD                  32768
SOLVENT              DMSO
NS                    925
DS                      8
SWH            30120.482 Hz
FIDRES          1.838408 Hz
AQ             0.5439488 sec
RG                    101
DW                 16.600 usec
DE                  18.00 usec
TE                  298.0 K
CNST2         145.0000000
D1             1.50000000 sec
D2             0.00344828 sec
D12            0.00002000 sec
TD0                     4
SFO1          150.9523507 MHz
NUC1                  13C
P1                  12.00 usec
P13               2000.00 usec
PLW0      0 W
PLW1         107.76000214 W
SPNAM[5]      Crp60comp.4
SPOAL5              0.500
SPOFFS5   0 Hz
SPW5          23.70800018 W
SFO2          600.2724011 MHz
NUC2                   1H
CPDPRG[2          waltz65
P3                   8.00 usec
P4                  16.00 usec
PCPD2               70.00 usec
PLW2           9.53950024 W
PLW12          0.12460000 W

F2 - Processing parameters
SI                  16384
SF            150.9380853 MHz
WDW                    EM
SSB       0
LB                   1.00 Hz
GB        0
PC                   1.40
```

I. C.C.B.S., University of Ka[illegible]chi Analytical Laboratory - Pakistan

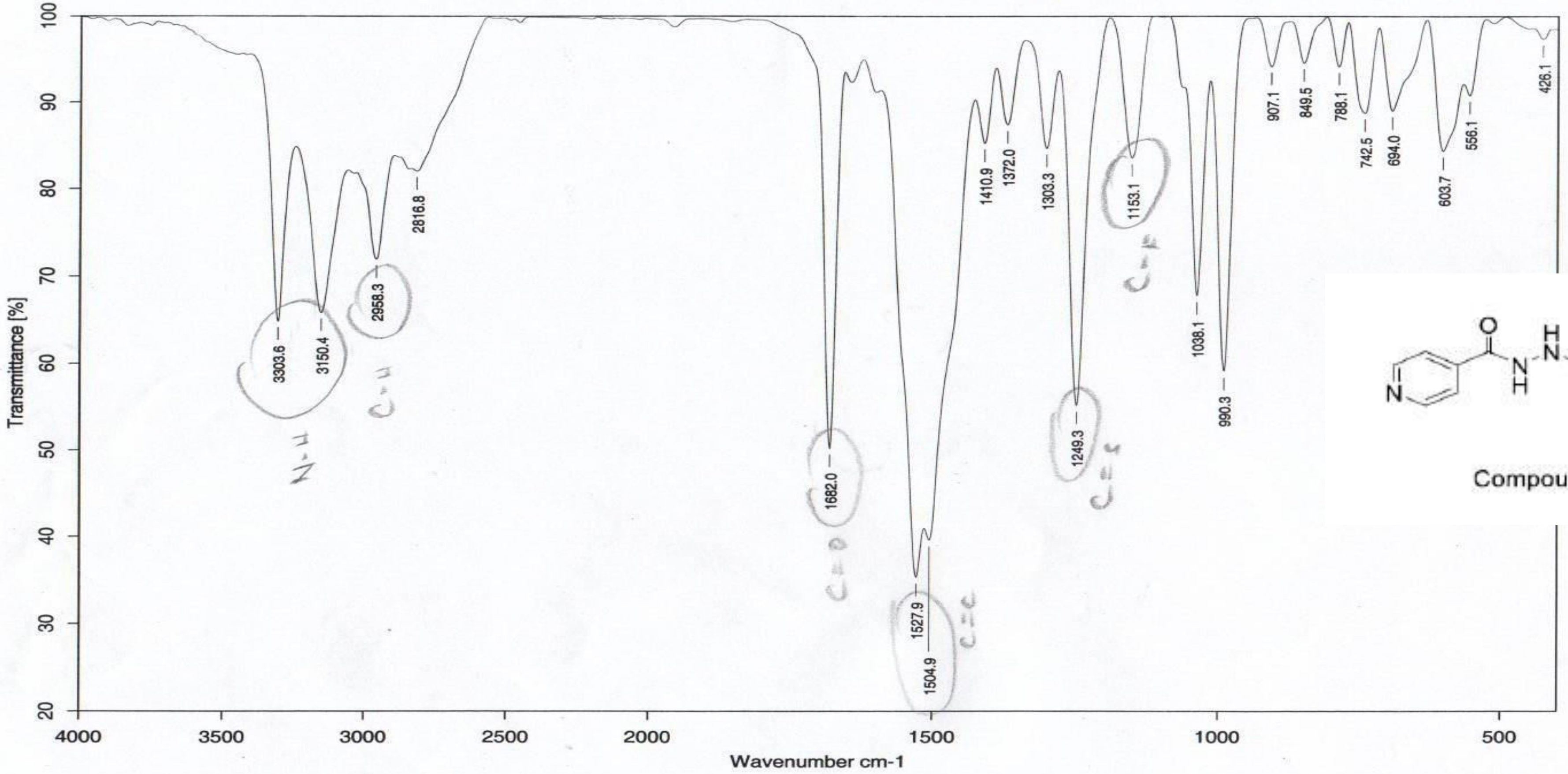

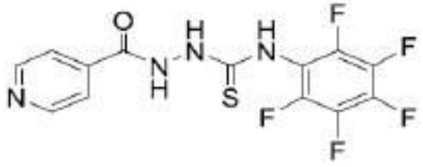

Compound 12

Sample : FZI-ISO17/Fazila Rizvi	Spectrum : FZ-I-ISO17.0 (in D:\IRSTUDENT)
Measured : 19/04/2017 on VECTOR22	Technic : Solid
Resolution : 4 cm-1 (10 scans)	Analyst : MA/ZA/JS

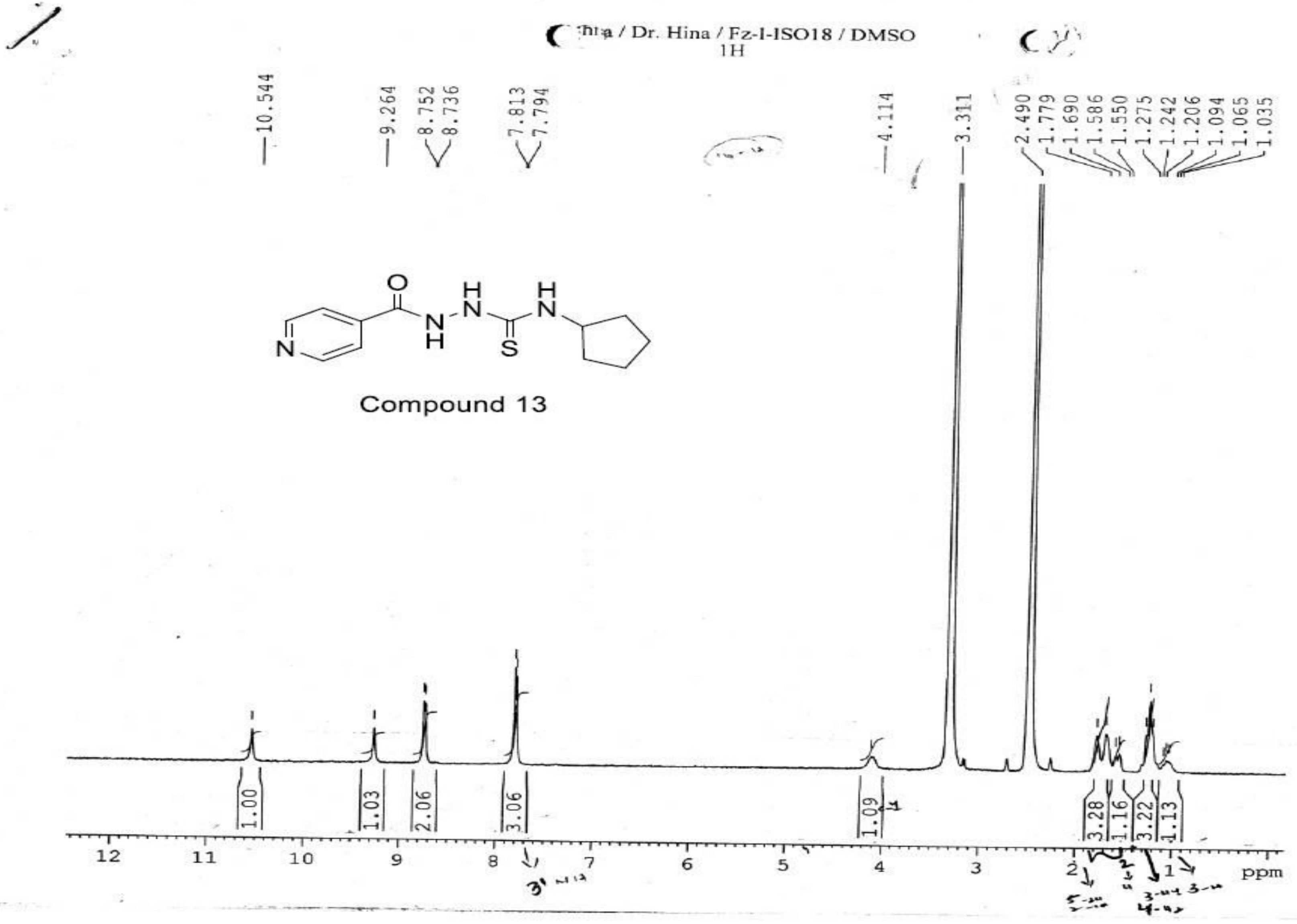

AVANCE AV-300
LAB NO: 108

NAME	Sep26-16
EXPNO	7
PROCNO	1
Date_	20160926
Time	10.45
INSTRUM	spect
PROBHD	5 mm DUL 13C-1
PULPROG	zg30
TD	32768
SOLVENT	DMSO
NS	64
DS	0
SWH	5995.204 Hz
FIDRES	0.182959 Hz
AQ	2.7329011 sec
RG	574.7
DW	83.400 usec
DE	6.50 usec
TE	297.8 K
D1	1.50000000 sec
TD0	1

======== CHANNEL f1 ========

NUC1	1H
P1	12.30 usec
PL1	4.00 dB
SFO1	300.1321009 MHz
SI	16384
SF	300.1300039 MHz
WDW	EM
SSB	0
LB	0.30 Hz
GB	0
PC	0.60

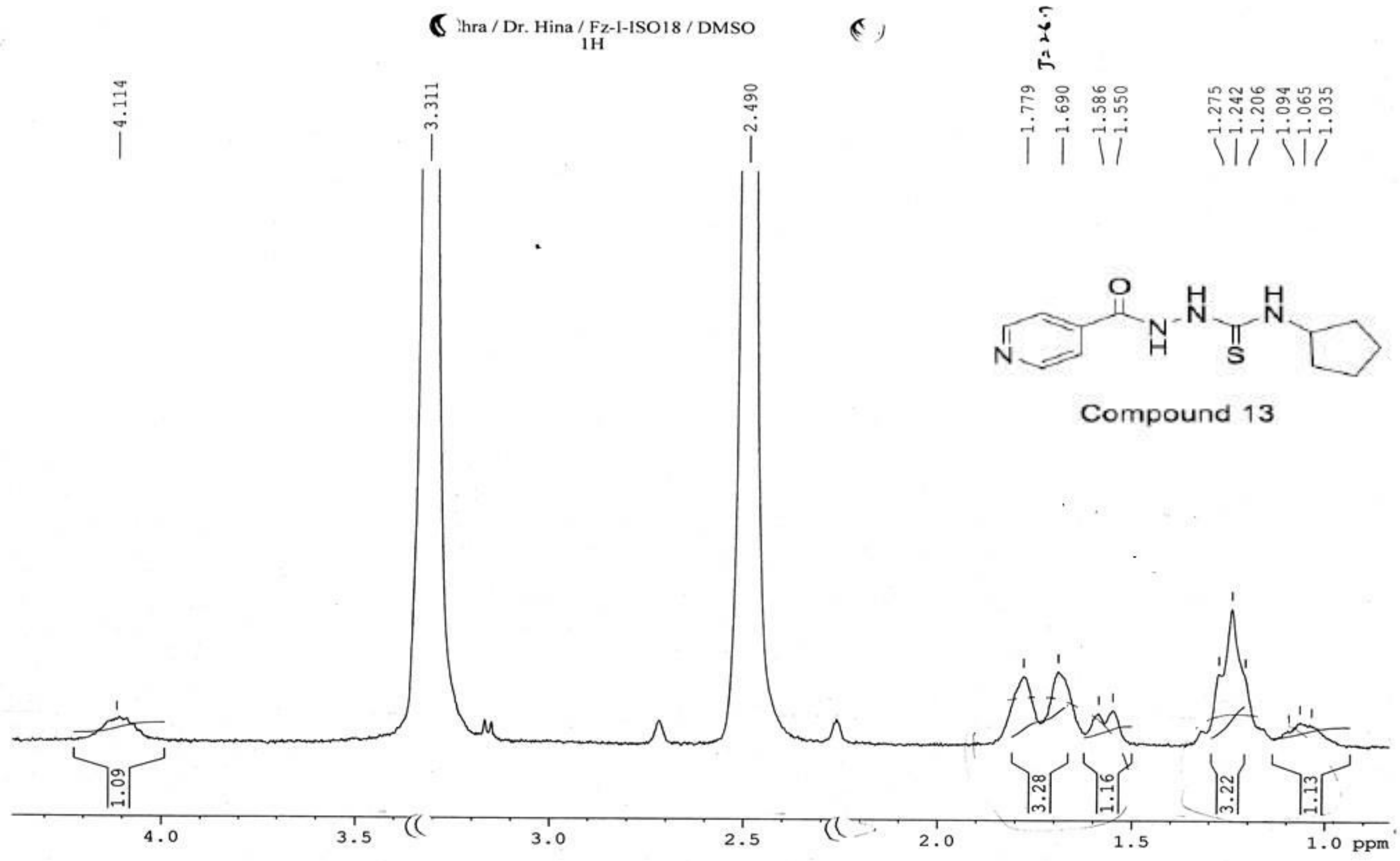
hra / Dr. Hina / Fz-I-ISO18 / DMSO
1H
4.114
3.311
2.490
1.779
1.690
1.586
1.550
1.275
1.242
1.206
1.094
1.065
1.035
Compound 13
1.09
3.28
1.16
3.22
1.13
4.0
3.5
3.0
2.5
2.0
1.5
1.0 ppm

HEJ-ICCBS
10/4/2016 3:44:37 PM

File: FZ-I-ISO-18
Sample: FAZILA RIZVI /DR. HINA
Instrument: JEOL-600H-2
Inlet: Direct Probe

Date Run: 10-04-2016 (Time Run: 15:42:59)

Ionization mode: FAB-

Scan: 2
Base: m/z 183; 100%FS TIC: 8366892

R.T.: .1

#Ions: 954

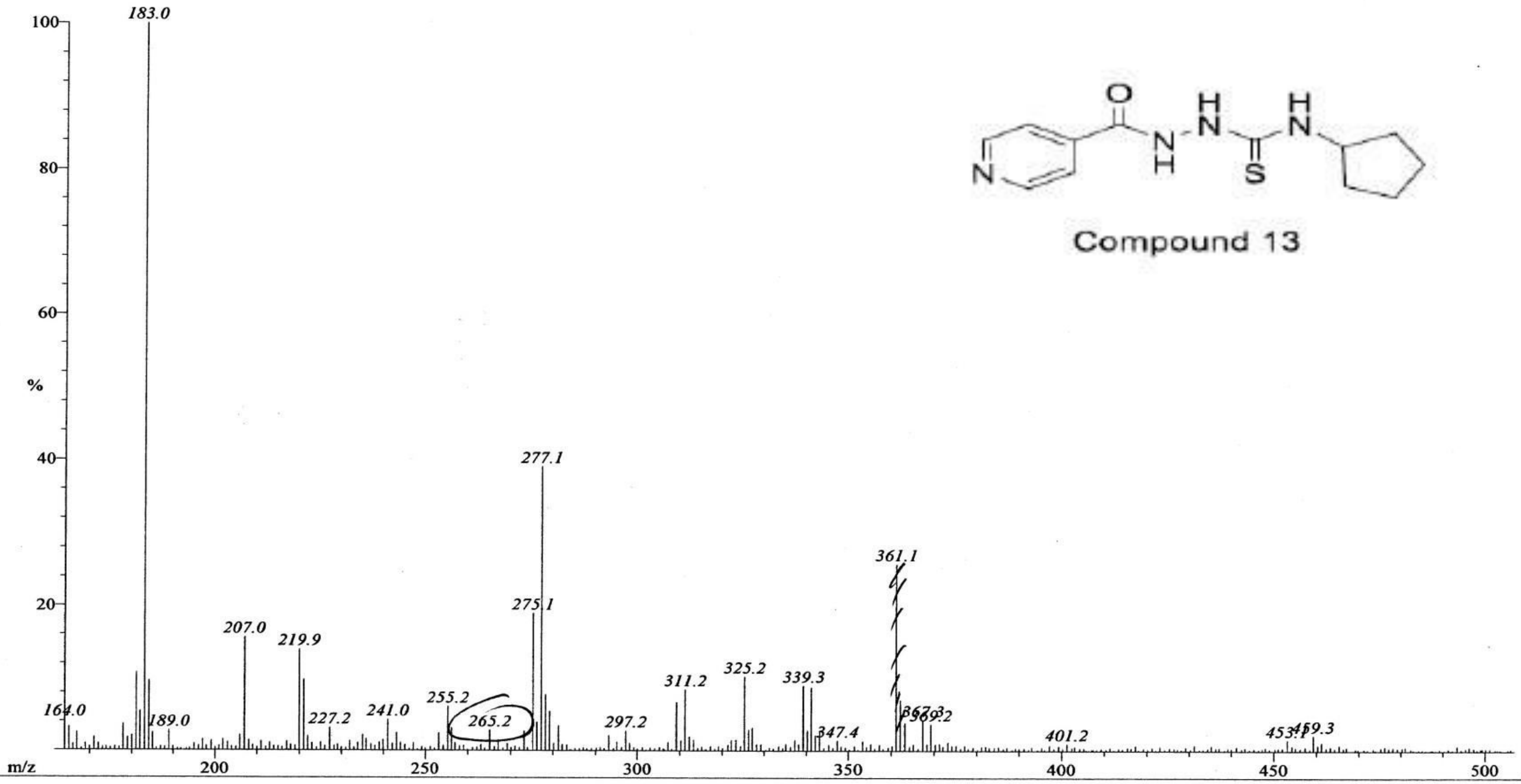

Compound 13

I. C.C.B.S., University of Ka[illegible]chi Analytical Laboratory - Pakistan

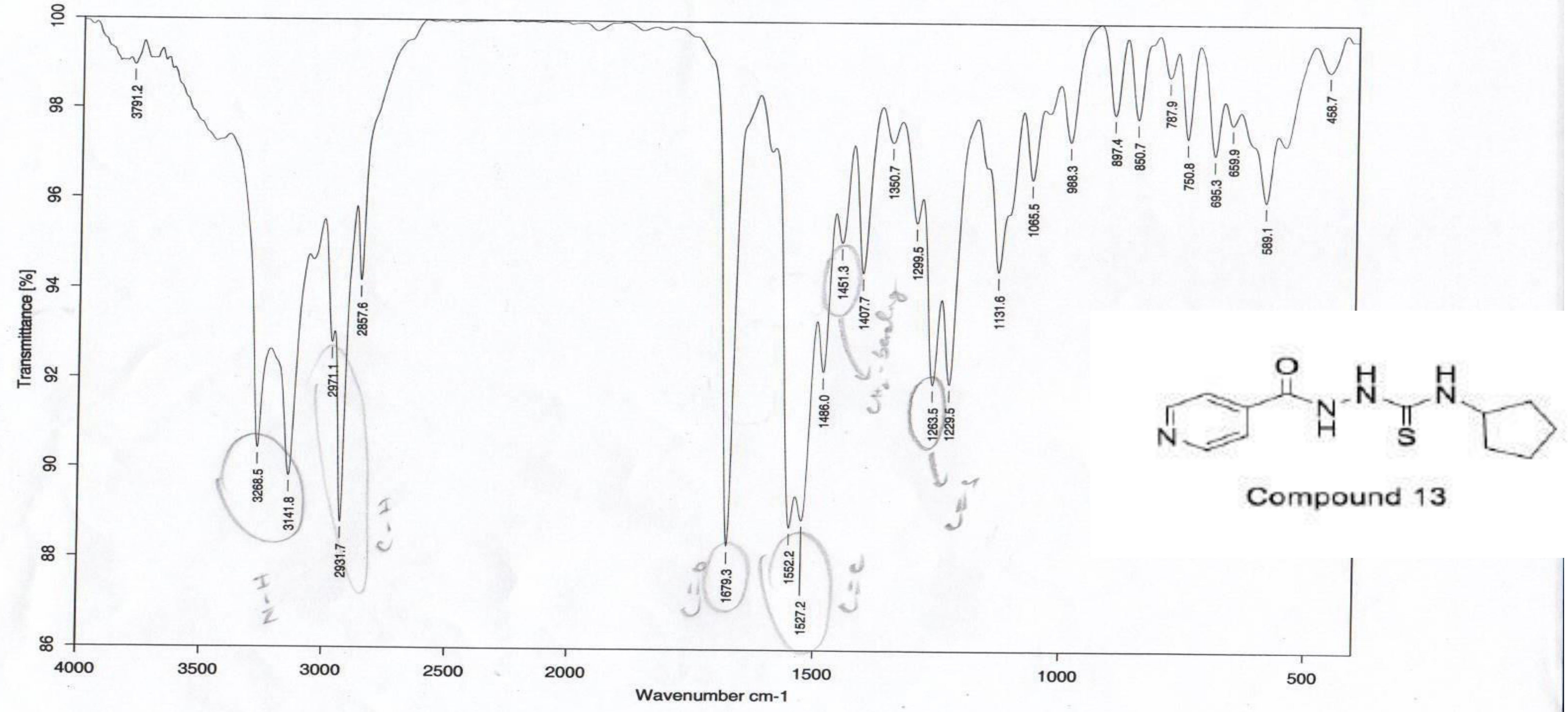

Compound 13

Sample : FZI-ISO18/Fazila Rizvi

Measured : 19/04/2017 on VECTOR22

Resolution : 4 cm-1 (10 scans)

Spectrum : FZ-I-ISO18.0 (in D:\IRSTUDENT)

Technic : Solid

Analyst : MA/ZA/JS

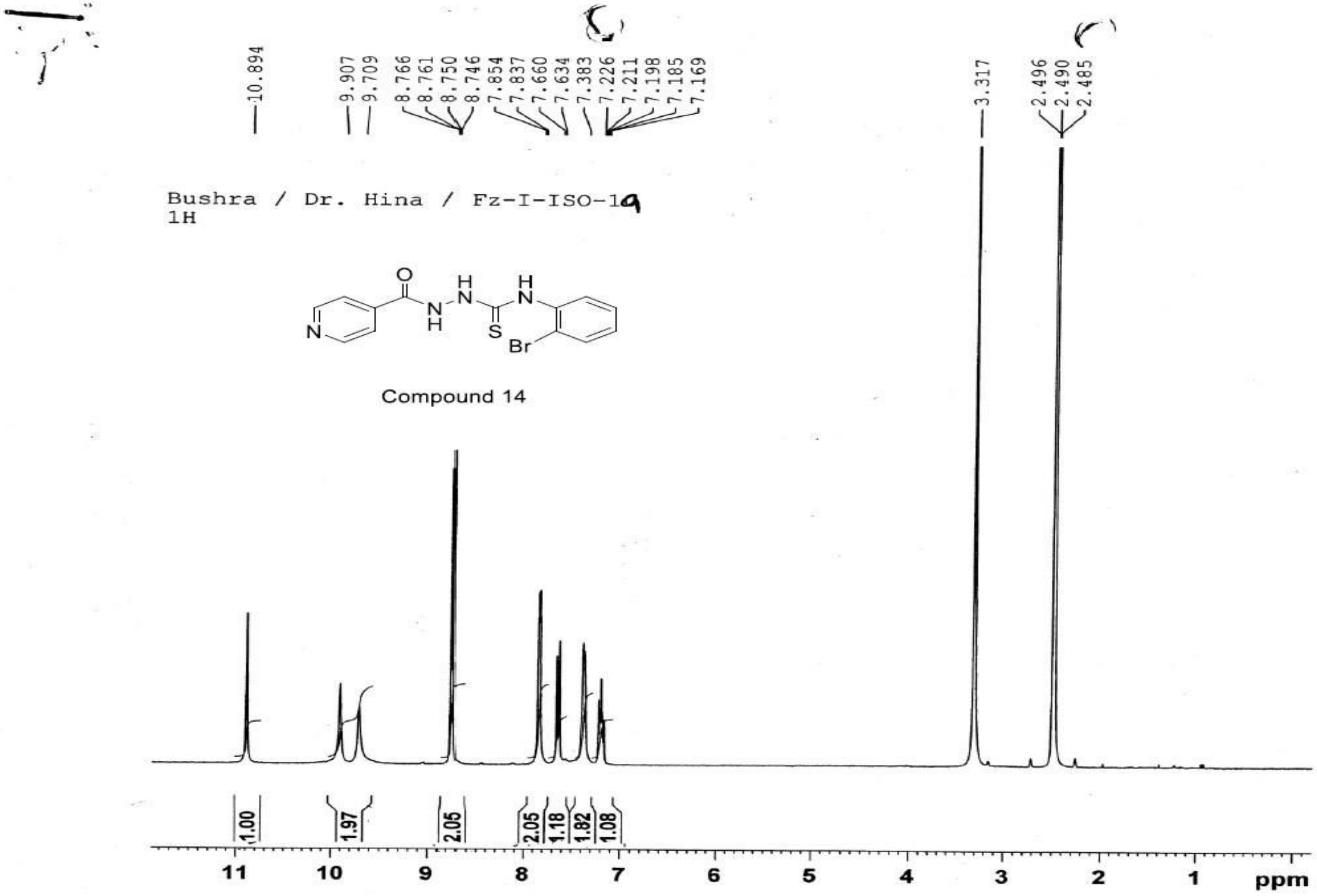

AVANCE AV - III
300 MHz, LAB # 116

NAME	Oct04-16	
EXPNO	4	
PROCNO	1	
Date_	20161004	
Time	10.47	
INSTRUM	Spect	
PROBHD	5 mm BBO BB-1H	
PULPROG	zg30	
TD	32768	
SOLVENT	DMSO	
NS	64	
DS	0	
SWH	6188.119	Hz
FIDRES	0.188846	Hz
AQ	2.6477044	sec
RG	203	
DW	80.800	usec
DE	6.50	usec
TE	300.0	K
D1	2.00000000	sec
TD0	1	

======== CHANNEL f1 ========

NUC1	1H	
P1	12.50	usec
PL1	0.00	dB
PL1W	13.16228485	W
SFO1	300.1318534	MHz
SI	32768	
SF	300.1300040	MHz
WDW	EM	
SSB	0	
LB	0.30	Hz
GB	0	
PC	1.00	

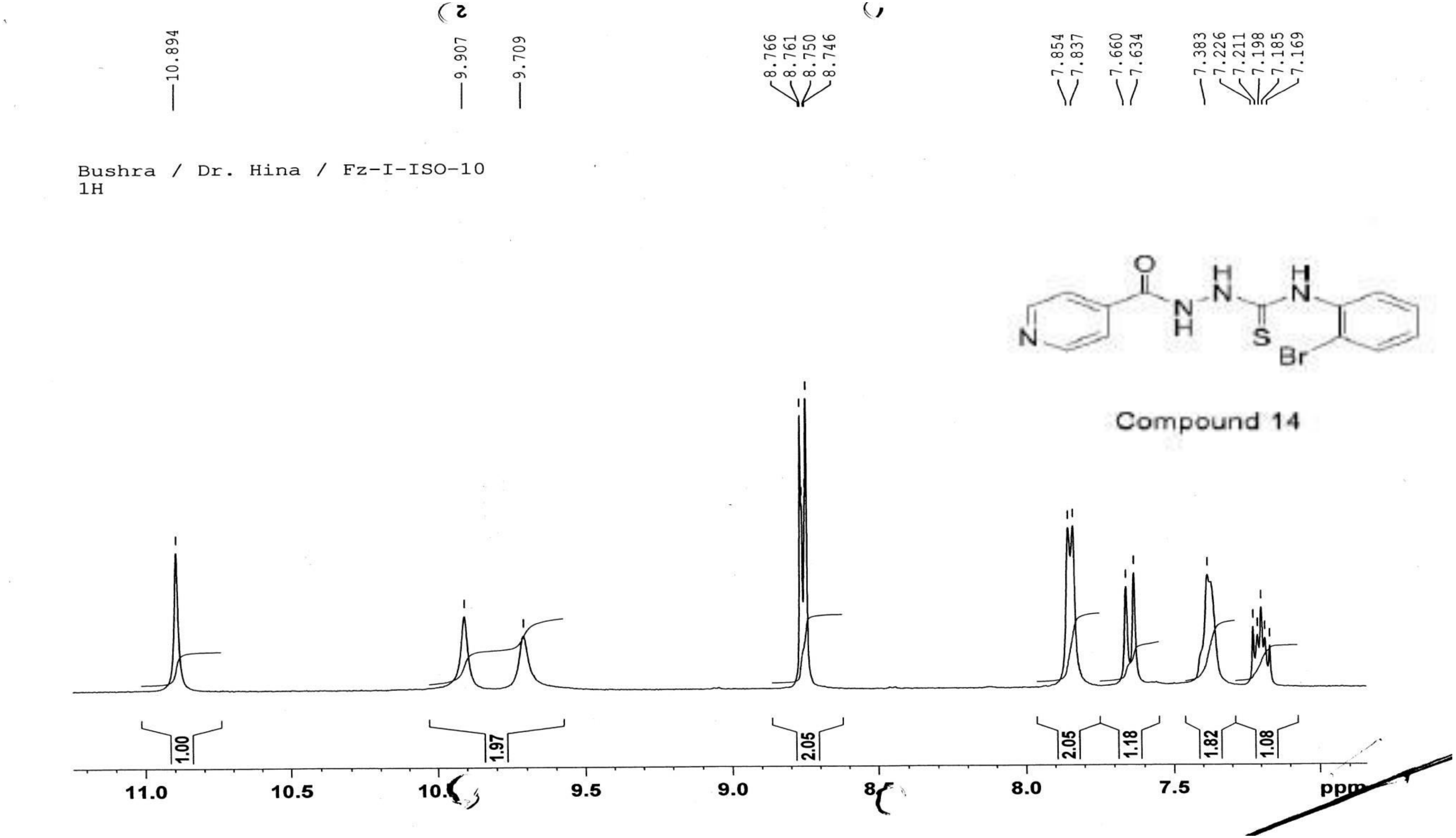
10.894
9.907
9.709
8.766
8.761
8.750
8.746
7.854
7.837
7.660
7.634
7.383
7.226
7.211
7.198
7.185
7.169
Bushra / Dr. Hina / Fz-I-ISO-10
1H
O
H
N
N
H
S
Br
Compound 14
1.00
1.97
2.05
2.05
1.18
1.82
1.08
11.0
10.5
9.5
9.0
8.0
7.5
ppm

File: FZ-I-ISO19
Sample: BUSHRA QAMAR /DR. HINA
Instrument: JEOL-600H-2
Inlet: Direct Probe

Date Run: 10-05-2016 (Time Run: 10:05:14)

Ionization mode: FAB+

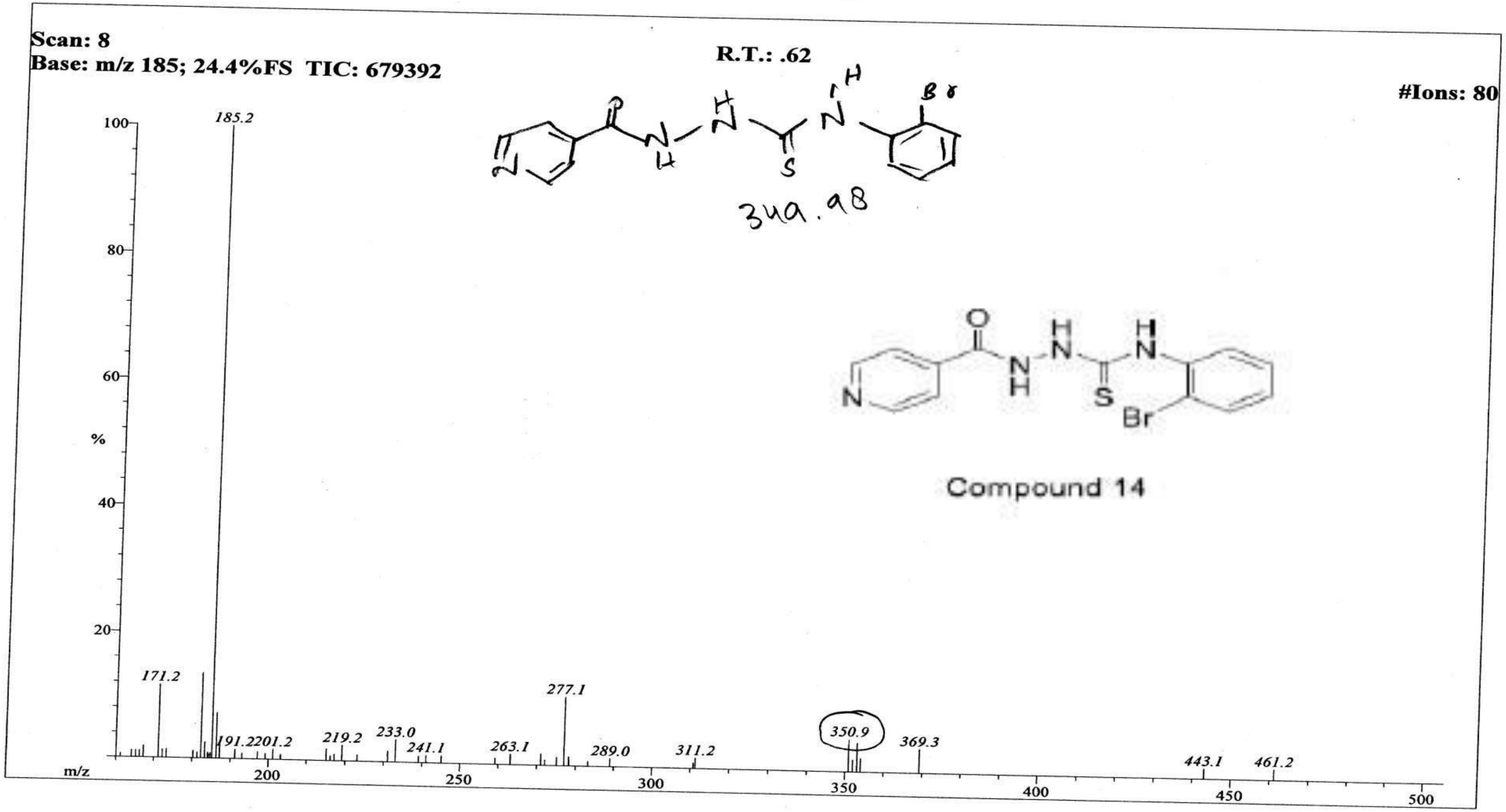

I. C.C.B.S., University of Ka[illegible]chi Analytical Laboratory - Pakistan

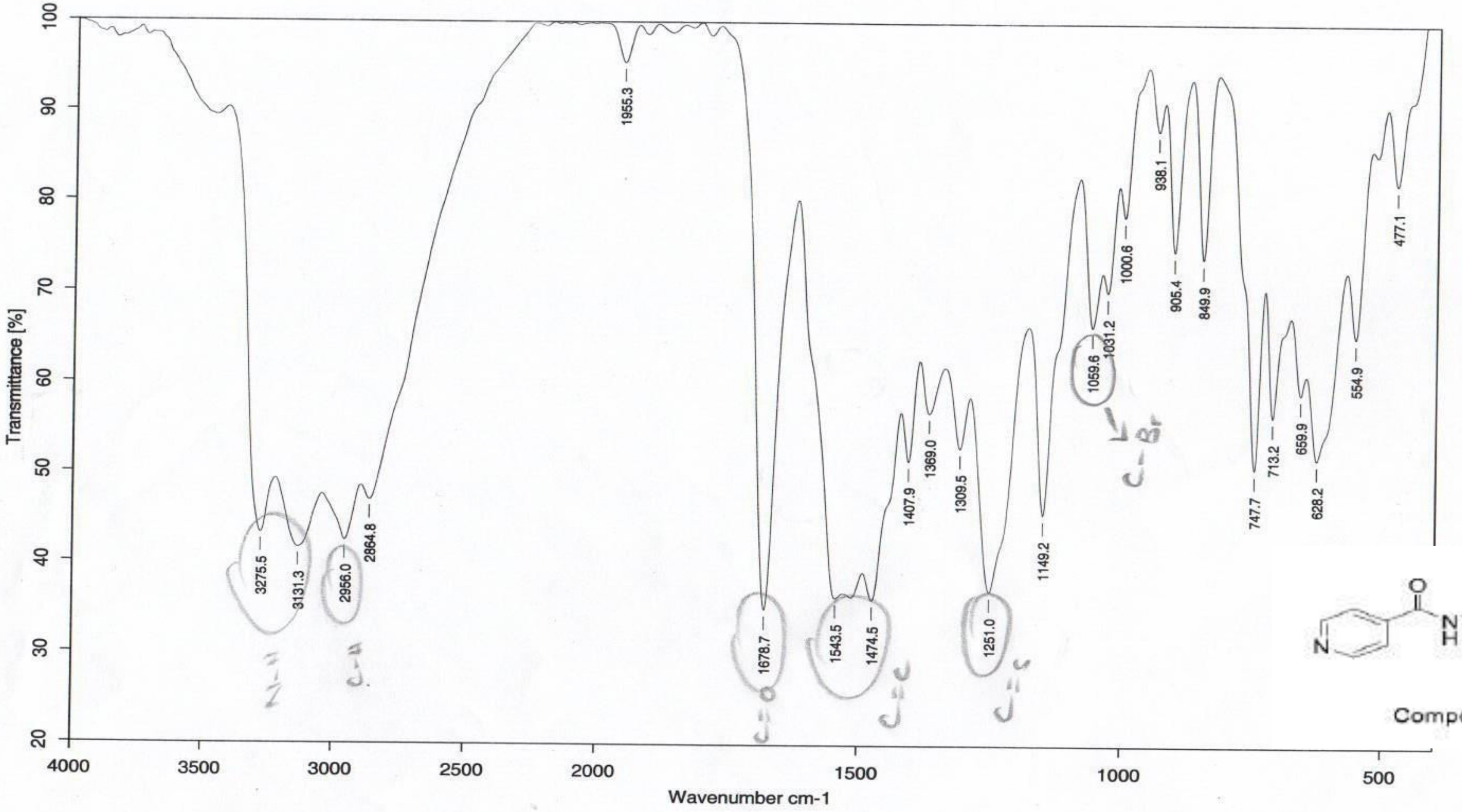

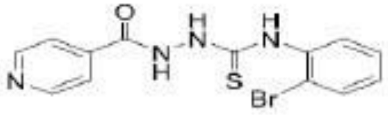

Compound 14

Sample : FZI-ISO19/Fazila Rizvi	
Measured : 19/04/2017 on VECTOR22	Spectrum : FZ-I-ISO19.0 (in D:\IRSTUDENT)
	Technic : Solid
Resolution : 4 cm-1 (10 scans)	Analyst : MA/ZA/JS

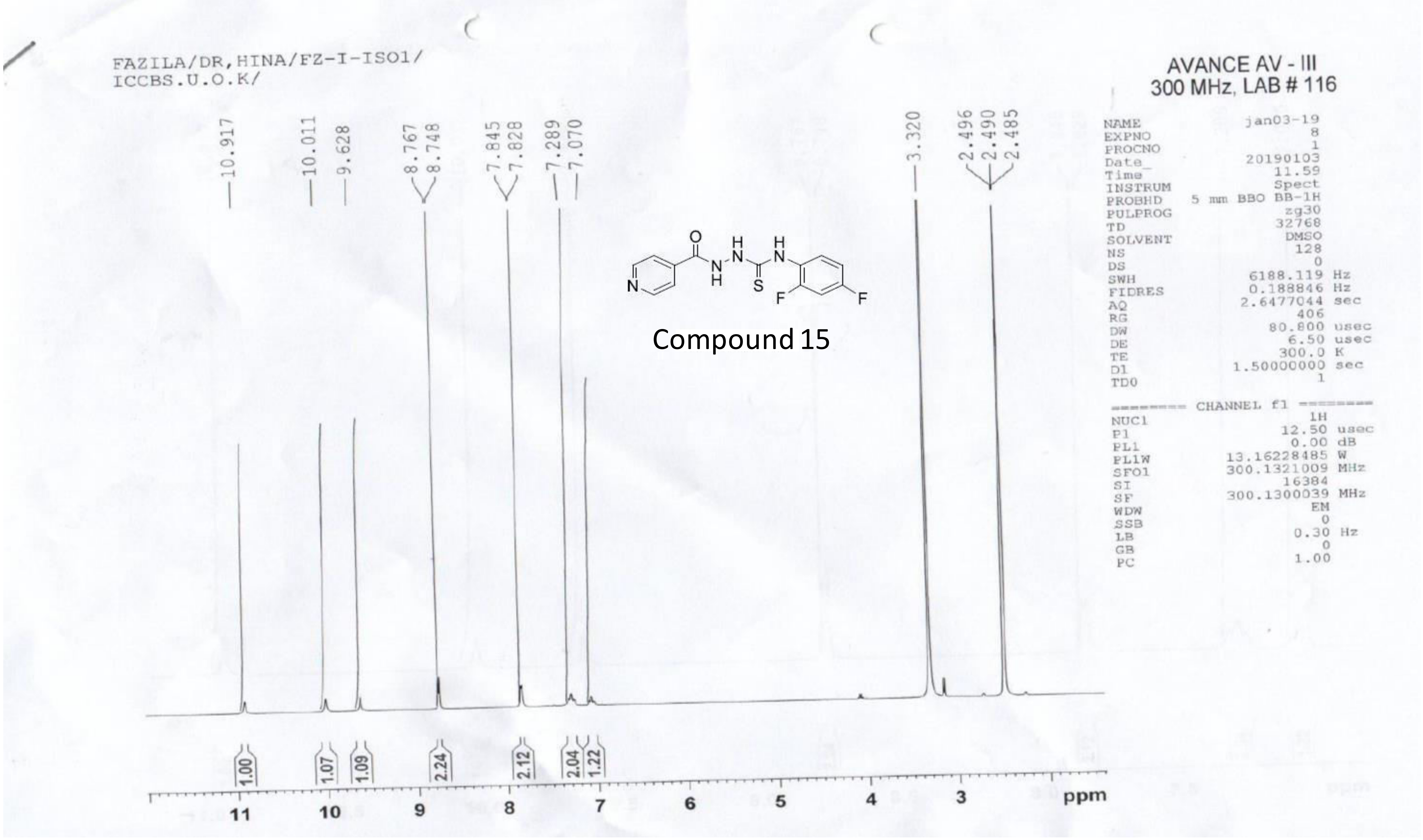

FAZILA/DR,HINA/FZ-I-ISO1/
ICCBS.U.O.K/
AVANCE AV - III
300 MHz, LAB # 116
10.917
10.011
9.628
8.767
8.748
7.845
7.828
7.289
7.070
3.320
2.496
2.490
2.485
Compound 15
NAME jan03-19
EXPNO 8
PROCNO 1
Date_ 20190103
Time 11.59
INSTRUM Spect
PROBHD 5 mm BBO BB-1H
PULPROG zg30
TD 32768
SOLVENT DMSO
NS 128
DS 0
SWH 6188.119 Hz
FIDRES 0.188846 Hz
AQ 2.6477044 sec
RG 406
DW 80.800 usec
DE 6.50 usec
TE 300.0 K
D1 1.50000000 sec
TD0 1
======== CHANNEL f1 ========
NUC1 1H
P1 12.50 usec
PL1 0.00 dB
PL1W 13.16228485 W
SFO1 300.1321009 MHz
SI 16384
SF 300.1300039 MHz
WDW EM
SSB 0
LB 0.30 Hz
GB 0
PC 1.00
1.00
1.07
1.09
2.24
2.12
2.04
1.22
11
10
9
8
7
6
5
4
3
ppm

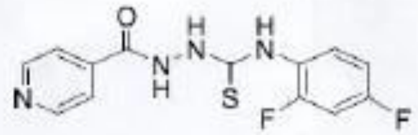

Compound 15

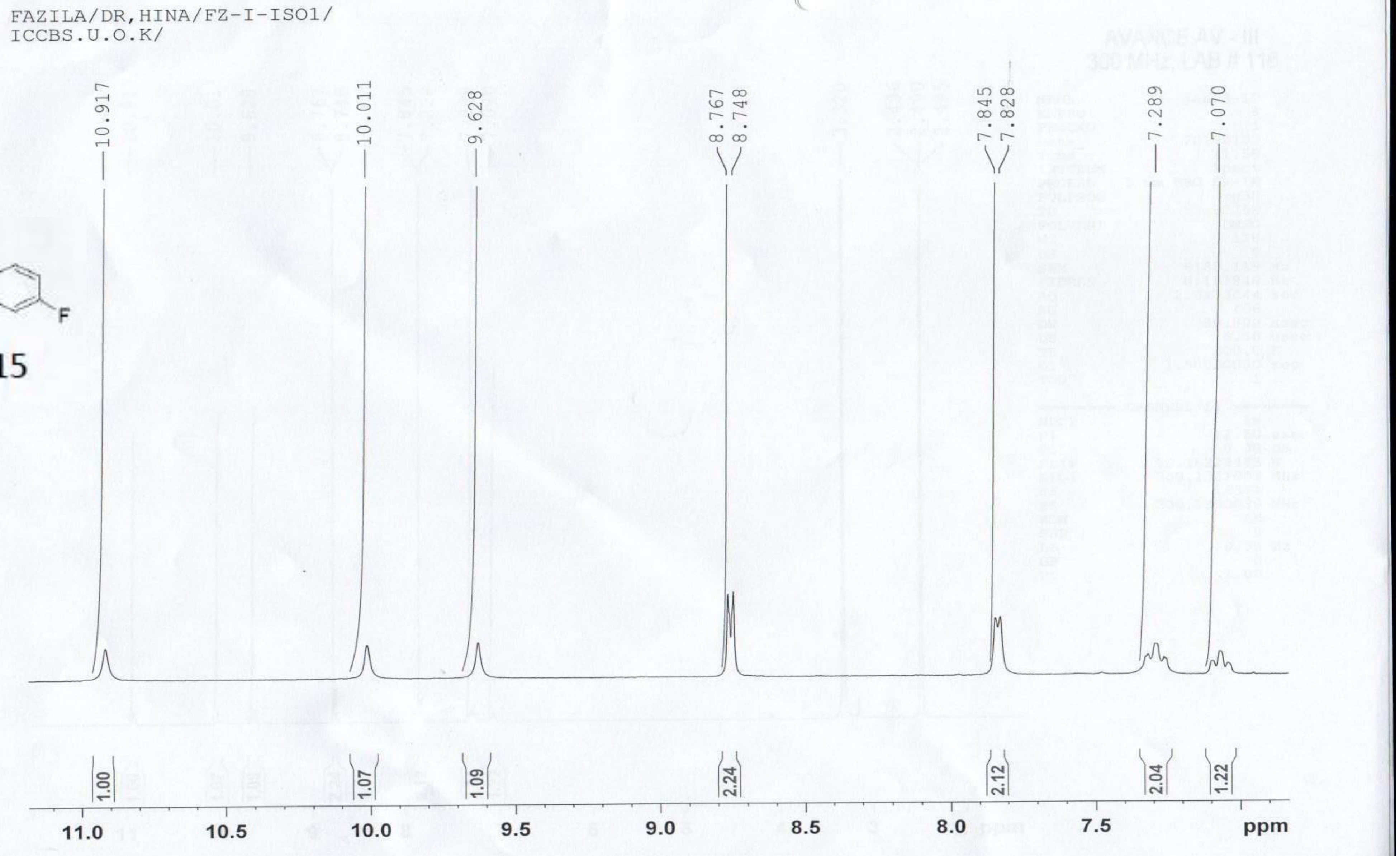

FAZILA/DR,HINA/FZ-I-ISO1/
ICCBS.U.O.K/
10.917
10.011
9.628
8.767
8.748
7.845
7.828
7.289
7.070
1.00
1.07
1.09
2.24
2.12
2.04
1.22
11.0
10.5
10.0
9.5
9.0
8.5
8.0
7.5
ppm

HEJ MASS SECTION
1/22/2016 11:23:32 AM

File: FZ-I-ISO1
Sample: FAZILA RIZVI / DR. HINA
Instrument: JEOL MS 600H-1

Date Run: 01-22-2016 (Time Run: 11:17:36)

Ionization mode: EI+

Scan: 22
Base: m/z 171; 22.7%FS TIC: 1213396

R.T.: 1.87

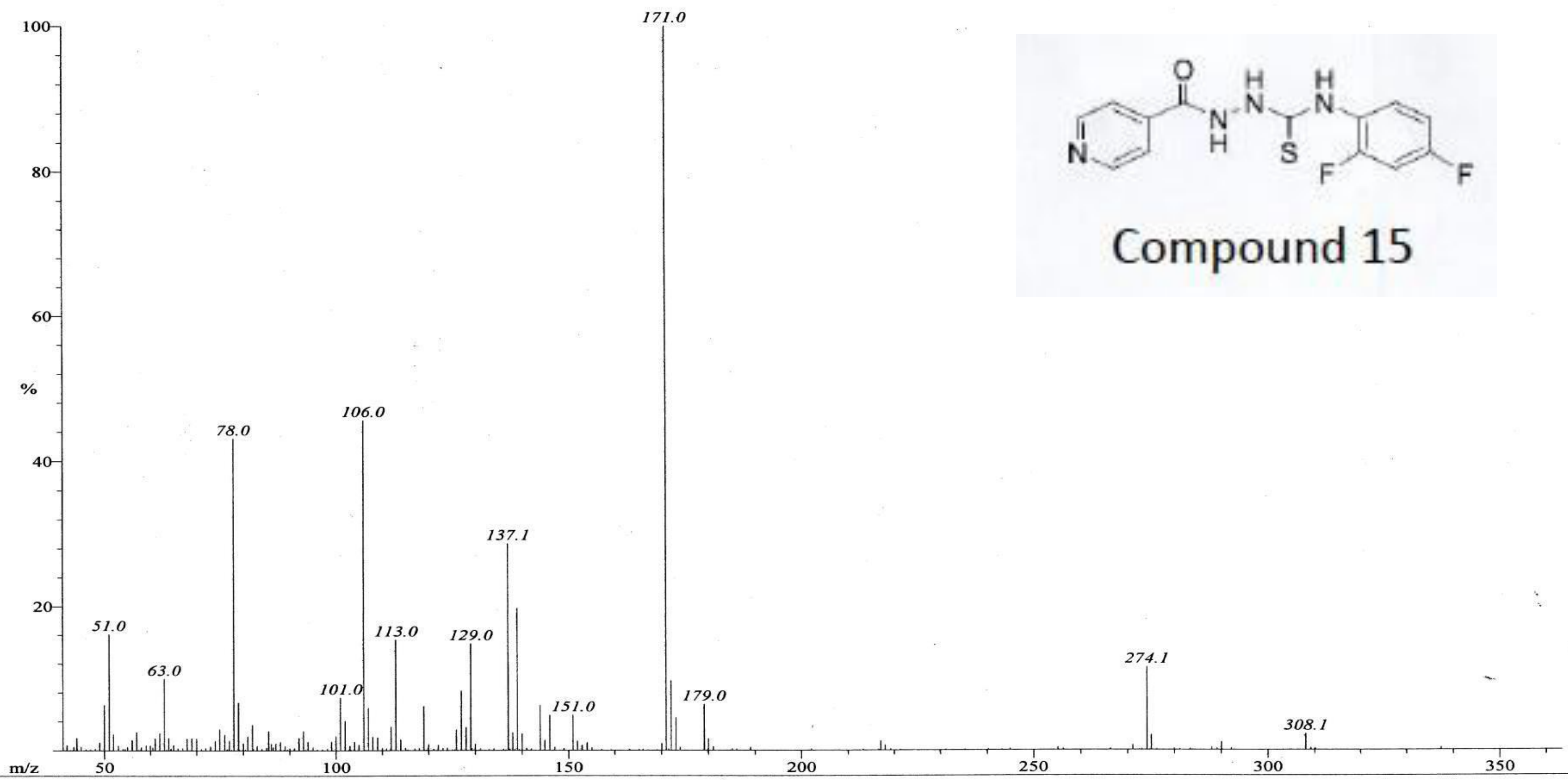

Compound 15

HEJ MASS SECTION
1/22/2016 11:23:57 AM

File: FZ-I-ISO1
Sample: FAZILA RIZVI / DR. HINA
Instrument: JEOL MS 600H-1

Date Run: 01-22-2016 (Time Run: 11:17:36)

Ionization mode: EI+

Scan: 22
Base: m/z 171; 22.7%FS TIC: 1213396

R.T.: 1.87

Threshold: .2% of Base

Displayed TIC: 1213396

Mass	%Base	Mass	%Base	Mass	%Base	Mass	%Base	Mass	%Base	Mass	%Base	Mass	%Base	Mass	%Base
41.9	.8	59.0	.8	76.5	.2	93.0	2.7	114.0	1.5	136.1	.2	170.0	1.0	271.1	.7
43.3	.5	60.0	.7	77.0	1.4	94.0	1.2	115.1	.4	137.1	28.7	171.0	100.0	273.0	.2
44.0	1.8	60.5	.5	78.0	43.1	95.0	.5	117.0	.3	137.4	.2	171.5	.2	274.1	11.5
44.1	.3	61.0	1.7	79.0	6.7	97.1	.2	118.0	.3	138.1	2.5	172.0	9.6	275.1	2.1
44.9	.6	62.0	2.4	80.0	1.0	98.1	.3	119.0	6.1	139.0	19.8	173.0	4.5	279.9	.2
45.9	.2	63.0	9.9	81.0	2.0	99.0	1.2	119.9	.4	140.0	2.3	174.0	.5	288.0	.4
46.9	.2	64.0	1.8	82.0	3.6	100.0	2.0	120.0	.9	141.0	.3	179.0	6.4	289.1	.2
48.0	.3	64.5	.3	82.2	.2	101.0	7.3	121.0	.2	142.0	.3	180.0	1.6	290.1	1.1
49.0	1.1	65.0	.8	83.0	.7	102.0	4.1	121.1	.2	144.0	6.3	181.1	.6	292.1	.3
50.0	6.3	66.0	.3	83.9	.2	103.0	.7	122.0	.8	144.9	1.4	185.1	.3	308.1	2.2
51.0	16.2	67.0	.4	84.9	.4	104.0	1.2	123.1	.3	146.0	4.8	186.0	.2	309.1	.3
52.0	2.2	68.0	1.7	85.5	2.7	105.0	.8	124.0	.4	147.0	.5	189.0	.5	310.0	.2
53.0	.7	69.0	1.7	86.0	.9	106.0	45.6	126.0	2.9	149.0	.3	202.1	.2	310.2	.2
54.0	.2	70.0	1.7	86.5	.5	107.0	5.9	127.0	8.2	151.0	4.9	217.0	1.3	337.4	.4
54.5	.3	71.0	.2	87.0	1.0	108.0	1.9	128.0	3.2	152.0	1.3	218.0	.8		
55.0	.5	72.0	.4	88.0	1.2	109.0	1.8	129.0	14.8	153.0	.7	219.2	.2		
56.0	1.5	73.0	.6	89.0	.7	110.0	.4	129.3	.3	154.0	1.1	245.0	.2		
57.0	2.6	74.0	1.3	90.0	.3	111.0	.3	130.0	.9	155.0	.4	255.2	.4		
57.9	.2	75.0	2.9	91.0	.4	112.0	3.3	131.0	.3	163.1	.2	256.3	.2		
58.0	.5	76.0	2.2	92.0	1.8	113.0	15.4	134.0	.3	166.0	.2	266.3	.2		

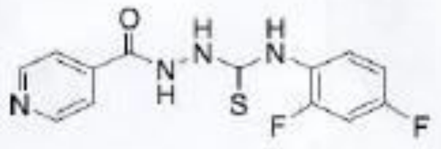

Compound 15

I. C.C.B.S., University of Ka[illegible]hi Analytical Laboratory - Pakistan

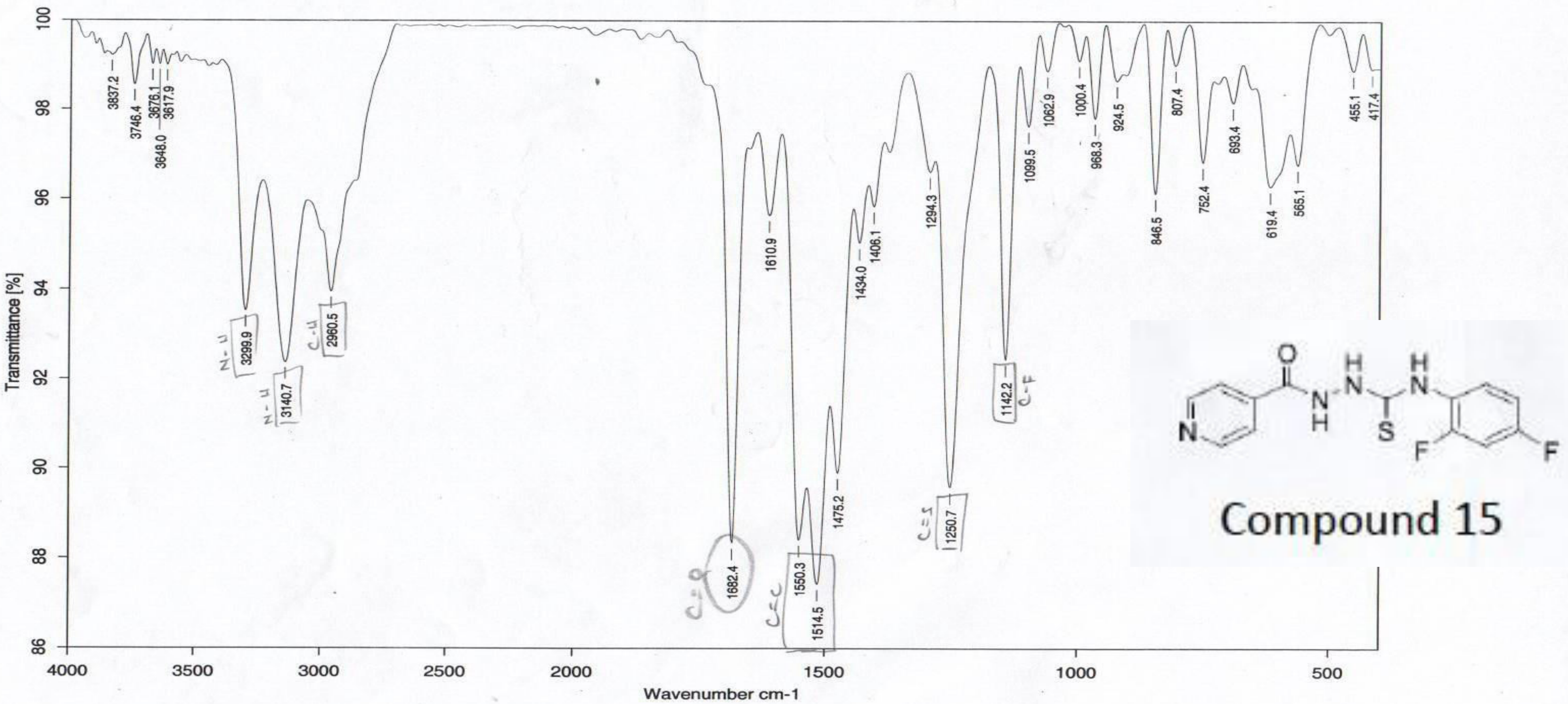

Compound 15

Sample : FZI-ISO1/Fazila Rizvi	Spectrum : FZ-I-ISO1.0 (in D:\IRSTUDENT)
Measured : 19/04/2017 on VECTOR22	Technic : Solid
Resolution : 4 cm-1 (10 scans)	Analyst : MA/ZA/JS

BUSHRA/DR.HINA/FZ.I.ISO.21
1H

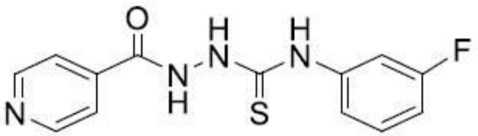

Compound 16

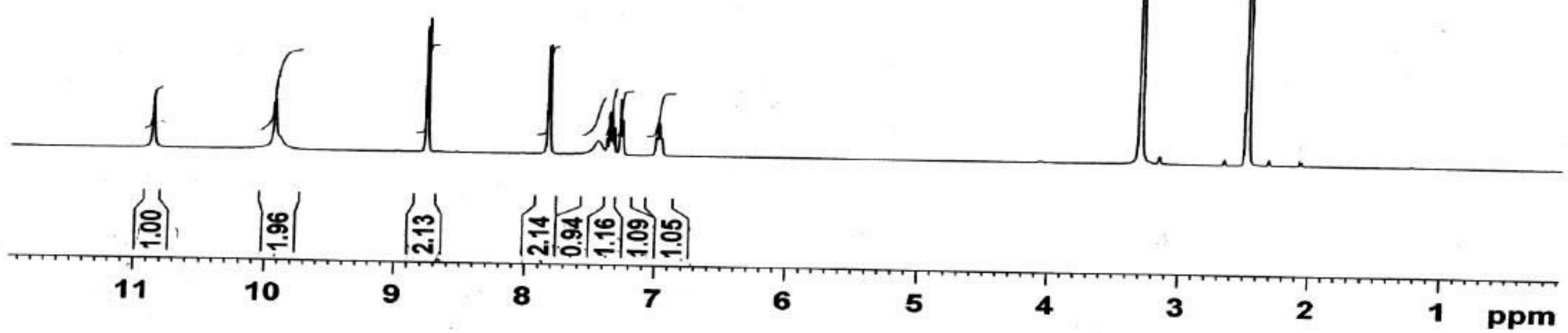

AVANCE AV-400 MHz
Lab # 115

NAME	oct06-16
EXPNO	7
PROCNO	1
Date_	20161006
Time	15.32
INSTRUM	spect
PROBHD	5 mm SEI 1H-13
PULPROG	zg30
TD	65536
SOLVENT	DMSO
NS	128
DS	0
SWH	8012.820 Hz
FIDRES	0.122266 Hz
AQ	4.0894966 sec
RG	362
DW	62.400 usec
DE	6.50 usec
TE	300.0 K
D1	1.50000000 sec
TD0	1

======== CHANNEL f1 ========

NUC1	1H
P1	10.80 usec
PL1	3.00 dB
SFO1	400.0332002 MHz
SI	32768
SF	400.0300041 MHz
WDW	EM
SSB	0
LB	0.30 Hz
GB	0
PC	0.20

10/19/2016 3:47:25 PM

File: FZ-I-ISO21-FABN
Sample: BUSHRA QAMAR /DR. HINA
Instrument: JEOL-600H-2
Inlet: Direct Probe

Date Run: 10-19-2016 (Time Run: 15:44:52)

Ionization mode: FAB-

Scan: 10
Base: m/z 183; 59.2%FS TIC: 2167114

R.T.: .8

#Ions: 427

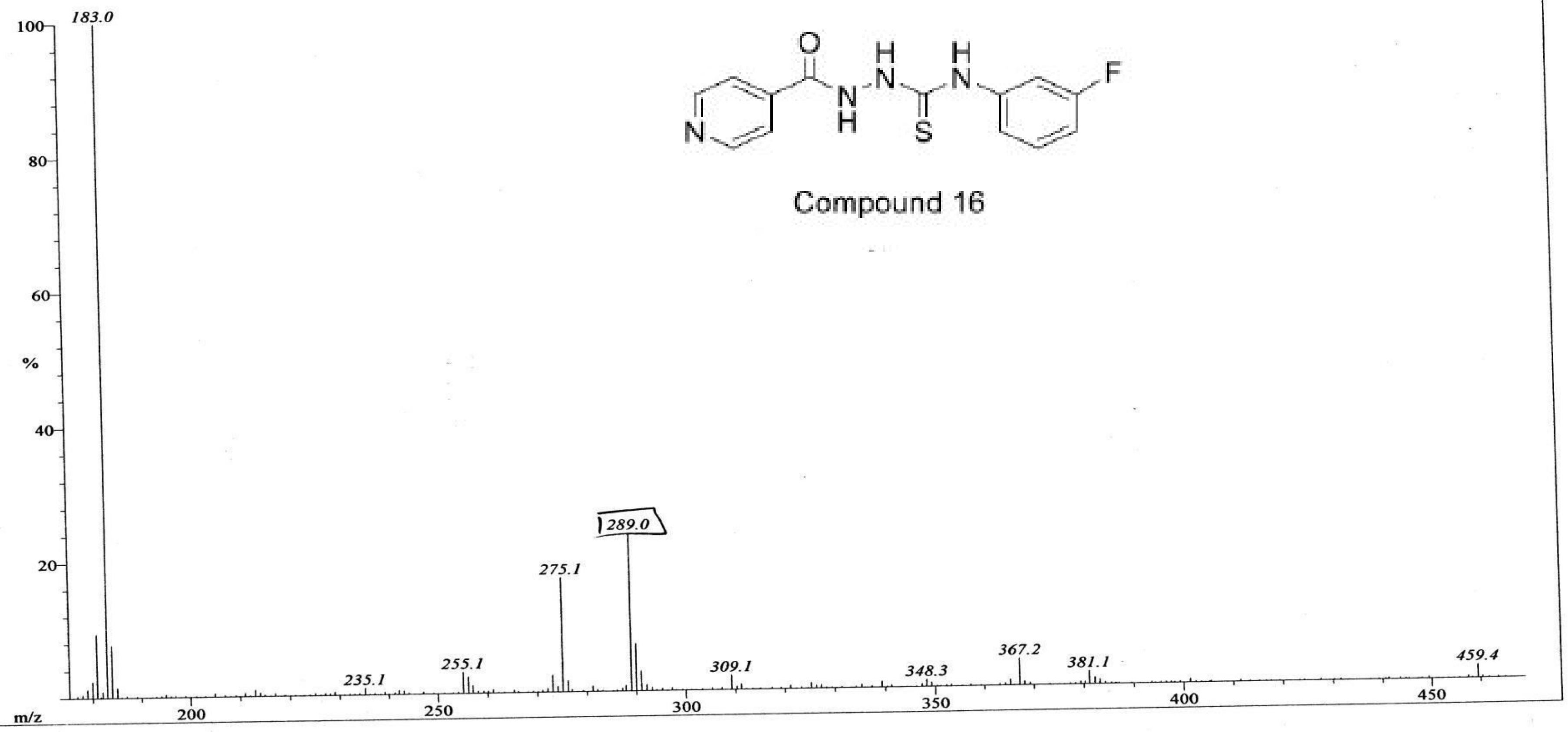

I. C.C.B.S., University of Ka[illegible]chi Analytical Laboratory - Pakistan

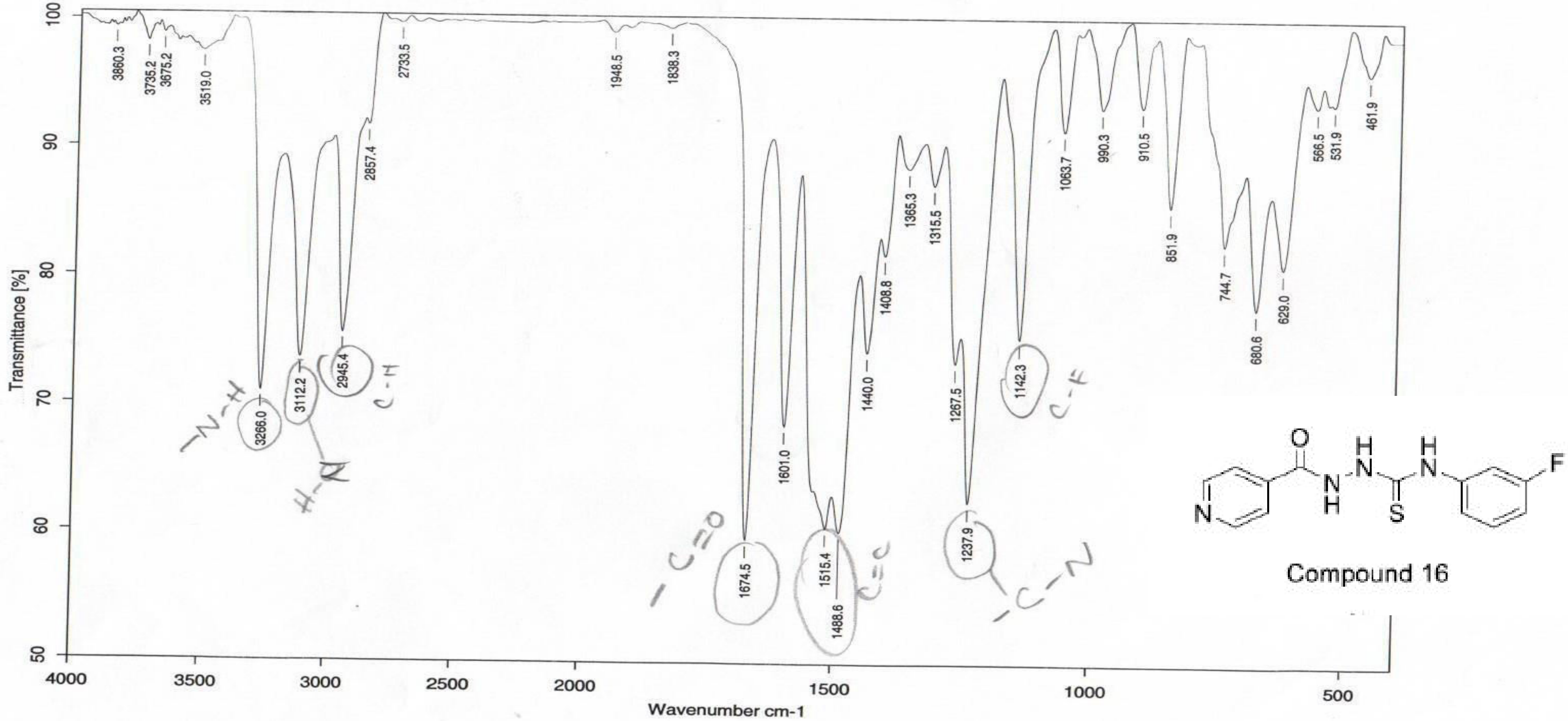

Compound 16

Sample : Fz-I-Iso21/Fazila Rizvi/Dr. Hina

Measured : 29/12/2016 on VECTOR22

Resolution : 4 cm-1 (10 scans)

Spectrum : Fz-I-Iso21.0 (in D:\IRSTUDENT)

Technic : Solid

Analyst : M. Asif

BUSHRA/DR.HINA/FZ.I.ISO.22
1H

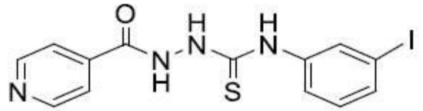

Compound 17

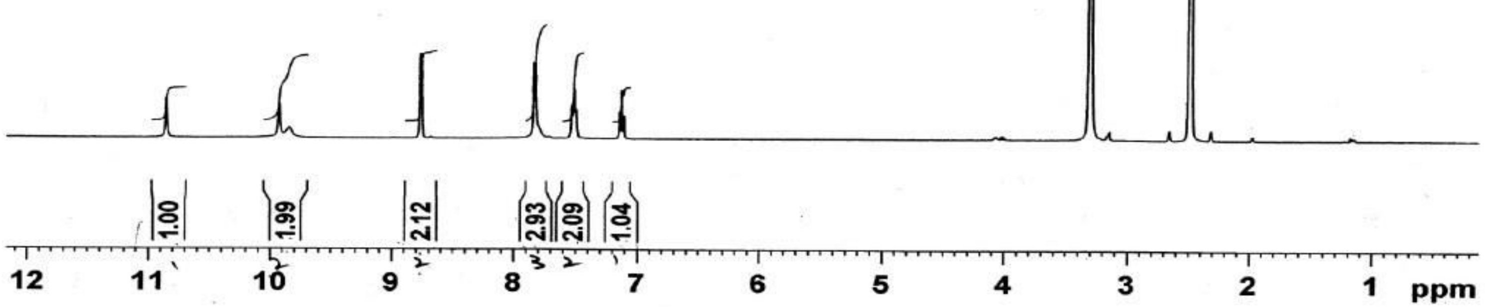

AVANCE AV-400 MHz
Lab # 115

NAME	oct06-16
EXPNO	6
PROCNO	1
Date_	20161006
Time	15.15
INSTRUM	spect
PROBHD	5 mm SEI 1H-13
PULPROG	zg30
TD	65536
SOLVENT	DMSO
NS	128
DS	0
SWH	8012.820 Hz
FIDRES	0.122266 Hz
AQ	4.0894966 sec
RG	574.7
DW	62.400 usec
DE	6.50 usec
TE	300.0 K
D1	1.50000000 sec
TD0	1

======== CHANNEL f1 ========

NUC1	1H
P1	10.80 usec
PL1	3.00 dB
SFO1	400.0332002 MHz
SI	32768
SF	400.0300041 MHz
WDW	EM
SSB	0
LB	0.30 Hz
GB	0
PC	0.20

File: FZ-I-ISO22-FABN
Sample: BUSHRA QAMAR /DR. HINA
Instrument: JEOL-600H-2
Inlet: Direct Probe

Date Run: 10-19-2016 (Time Run: 15:28:48)

Ionization mode: FAB-

Scan: 8
Base: m/z 183; 73.8%FS TIC: 2944182

R.T.: .62

#Ions: 565

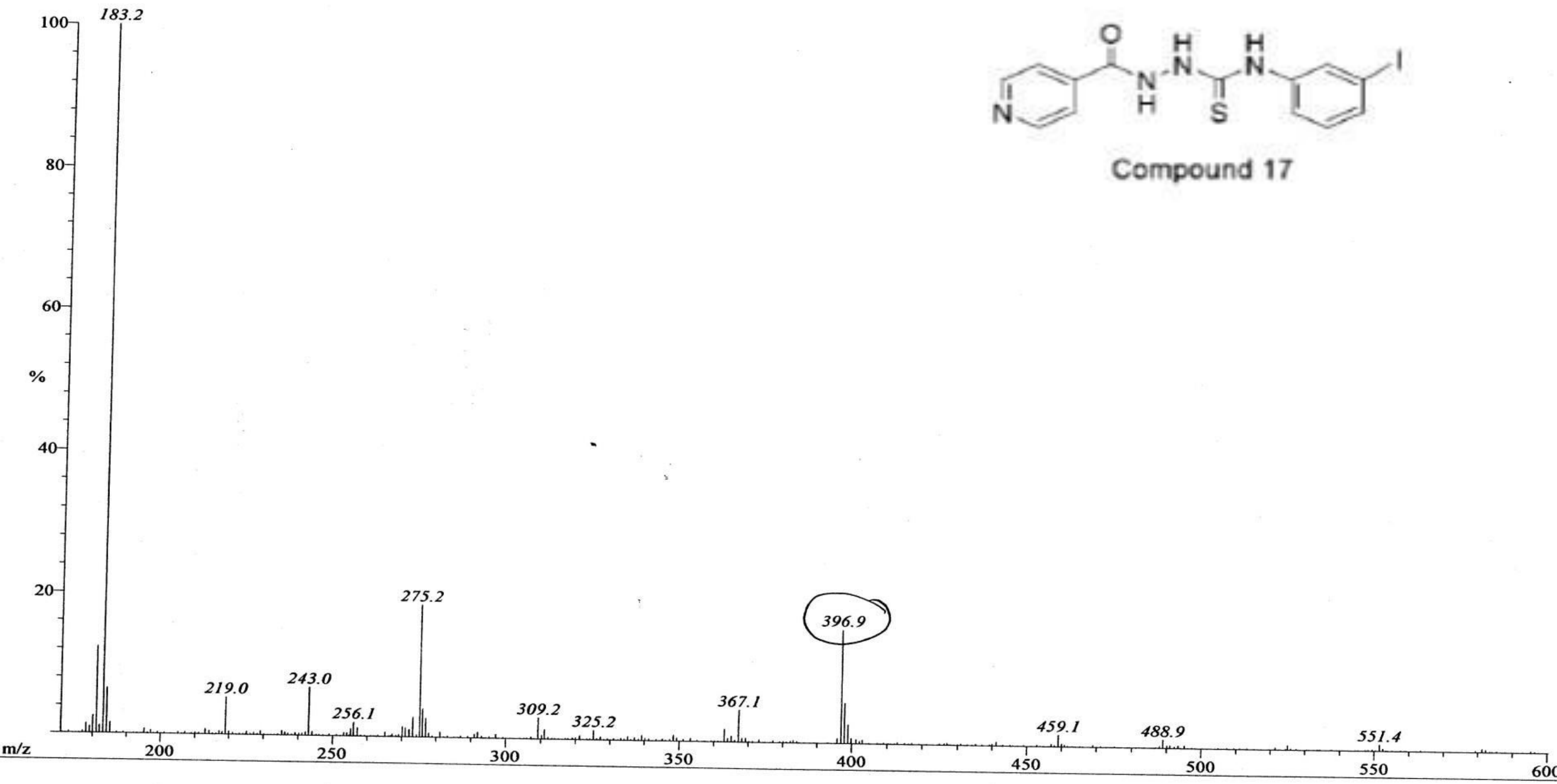

Compound 17

I. C.C.B.S., University of Ka[illegible]chi Analytical Laboratory - Pakistan

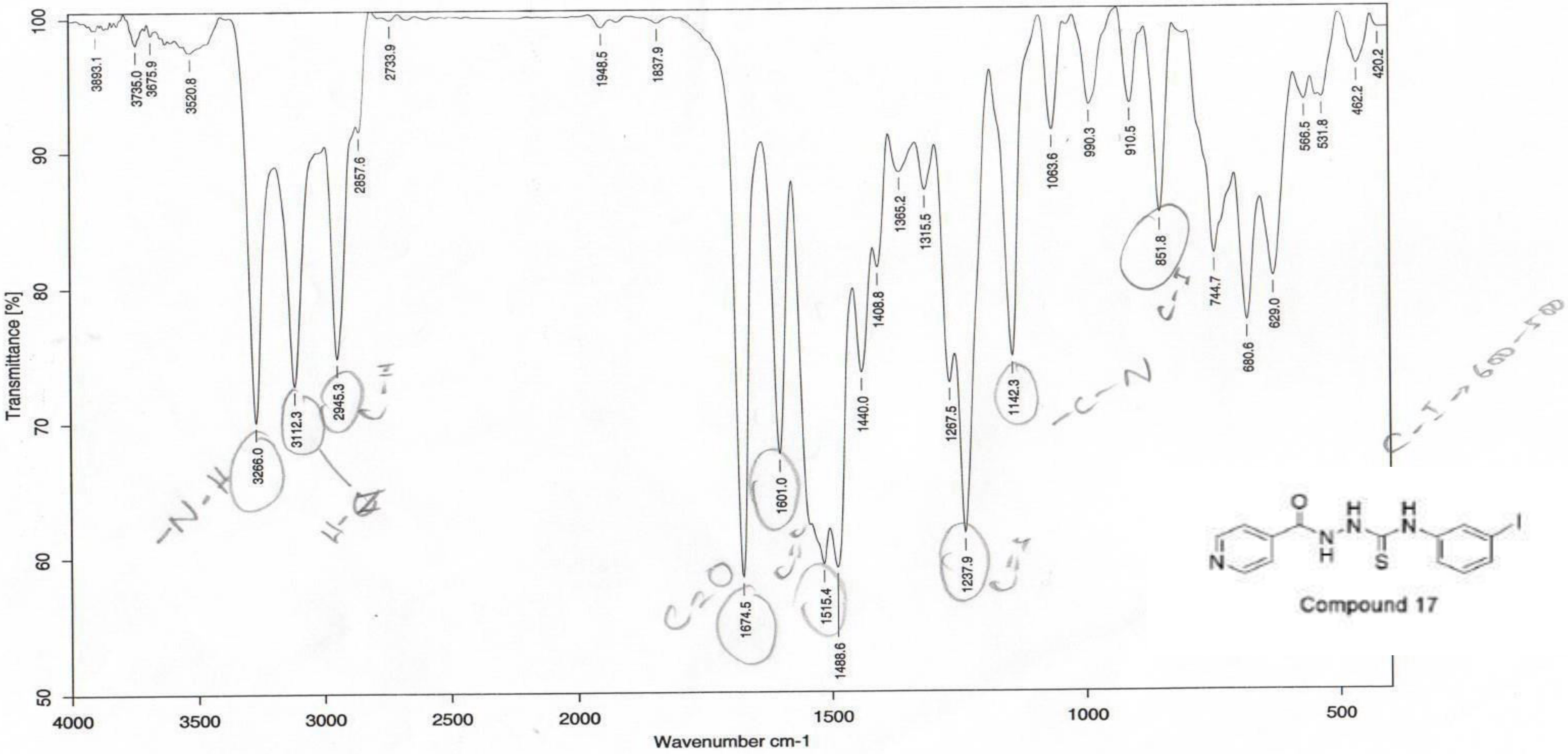

Compound 17

Sample : Fz-I-Iso22/Fazila Rizvi/Dr. Hina	Spectrum : Fz-I-Iso22.0 (in D:\IRSTUDENT)
Measured : 29/12/2016 on VECTOR22	Technic : Solid
Resolution : 4 cm-1 (10 scans)	Analyst : M. Asif

fazila rizvi/Dr.Hina/Fz-I-iso23
1H

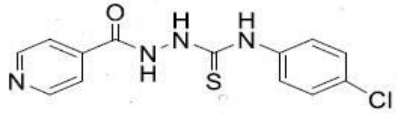

Compound 18

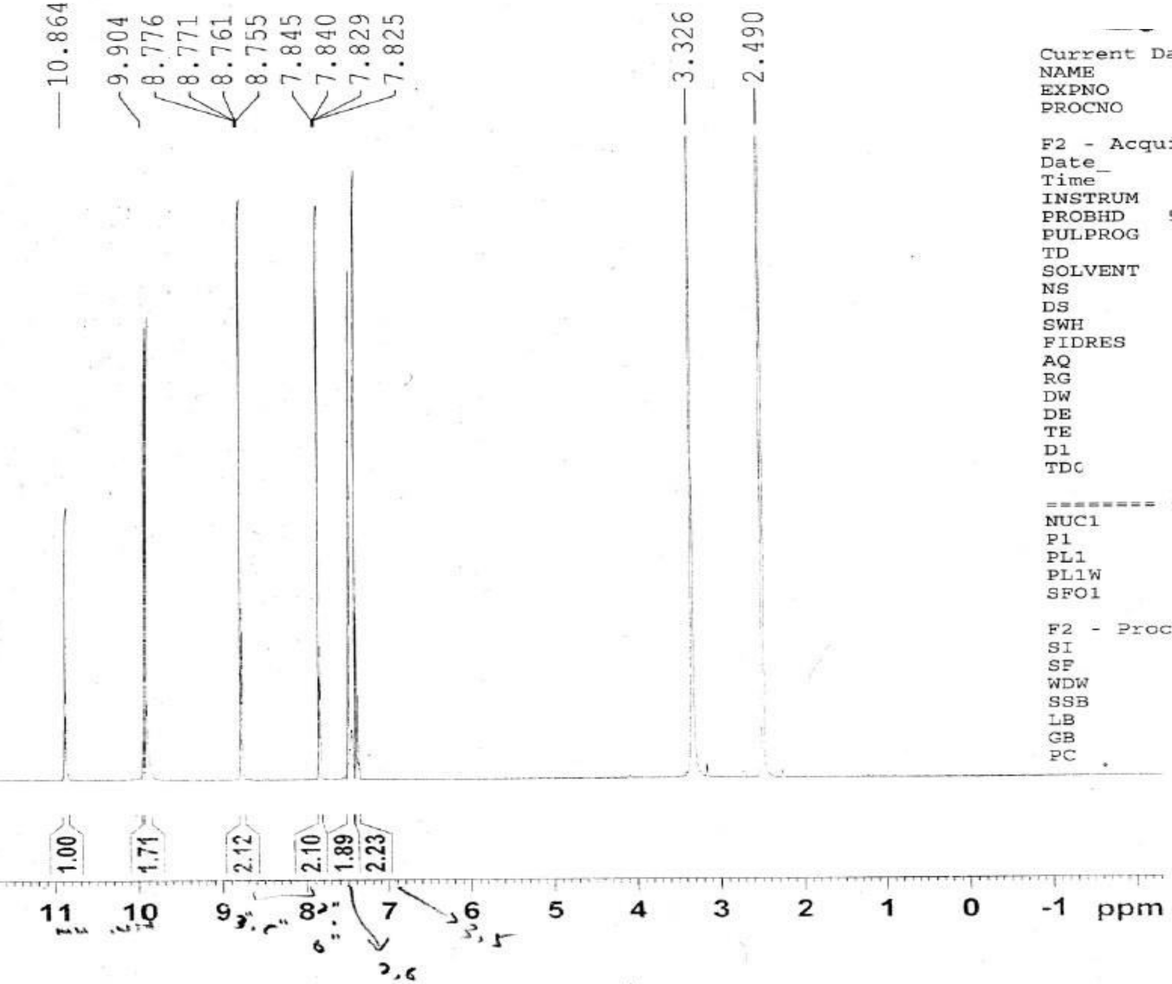

Current Data Parameters
NAME iso23
EXPNO 4
PROCNO 1

F2 - Acquisition Parameters
Date_ 20161117
Time 11.36
INSTRUM Spect
PROBHD 5 mm BBO BB-1H
PULPROG zg30
TD 32768
SOLVENT DMSO
NS 64
DS 0
SWH 6188.119 Hz
FIDRES 0.188846 Hz
AQ 2.6476543 sec
RG 203
DW 80.800 usec
DE 6.50 usec
TE 300.0 K
D1 2.00000000 sec
TD0 1

======== CHANNEL f1 ========
NUC1 1H
P1 12.50 usec
PL1 0 dB
PL1W 13.16228485 W
SFO1 300.1324010 MHz

F2 - Processing parameters
SI 32768
SF 300.1300040 MHz
WDW EM
SSB 0
LB 0.30 Hz
GB 0
PC 1.00

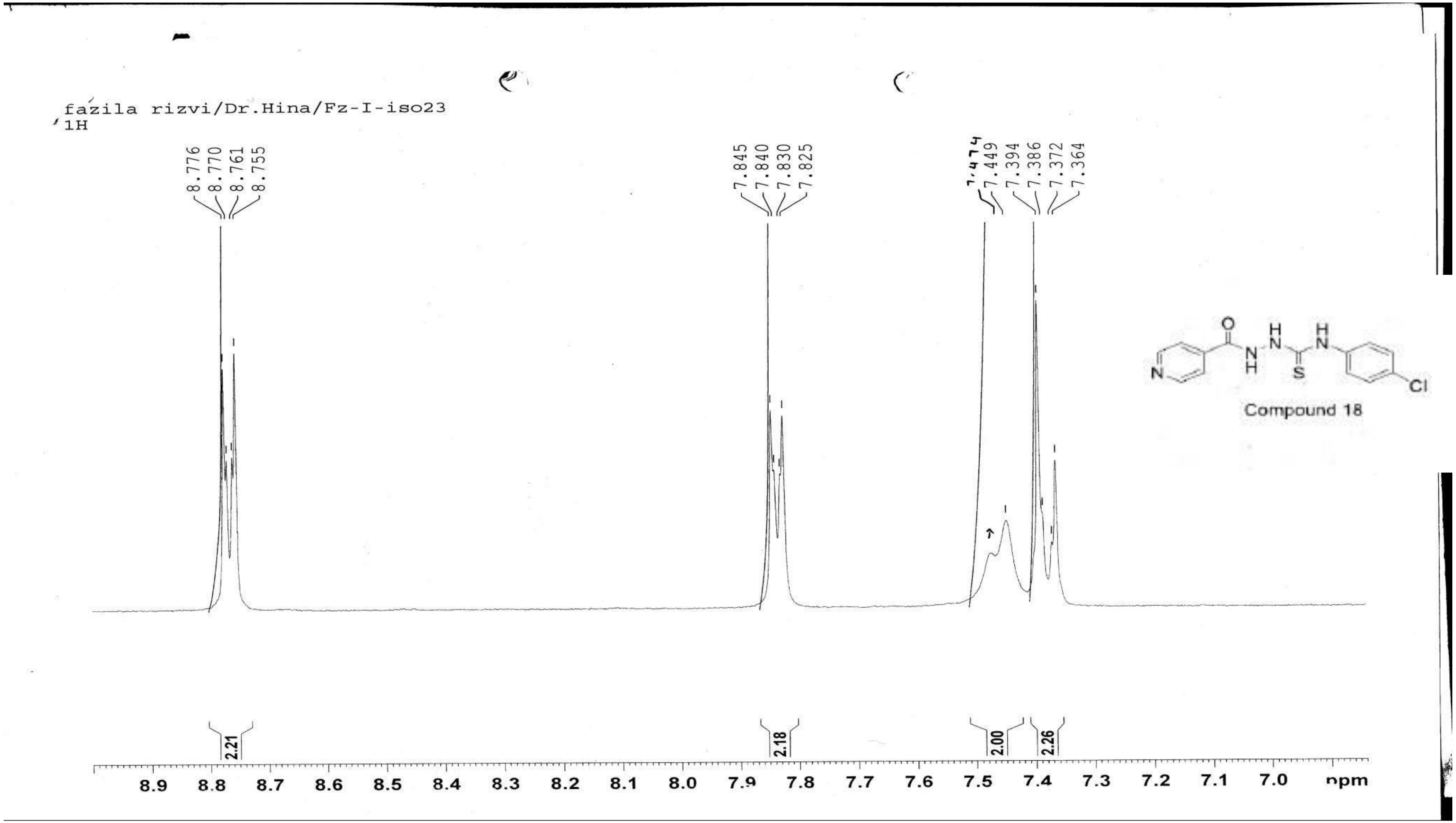

fazila rizvi/Dr.Hina/Fz-I-iso23
1H
8.776
8.770
8.761
8.755
7.845
7.840
7.830
7.825
7.474
7.449
7.394
7.386
7.372
7.364
2.21
2.18
2.00
2.26
8.9 8.8 8.7 8.6 8.5 8.4 8.3 8.2 8.1 8.0 7.9 7.8 7.7 7.6 7.5 7.4 7.3 7.2 7.1 7.0 ppm

Compound 18

File: FZ-I-ISO23-FABN
Sample: BUSHRA QAMAR /DR. HINA
Instrument: JEOL-600H-2
Inlet: Direct Probe

Date Run: 10-19-2016 (Time Run: 15:52:27)

Ionization mode: FAB-

Scan: 8
Base: m/z 183; 51.4%FS TIC: 1986322

R.T.: .62

#Ions: 591

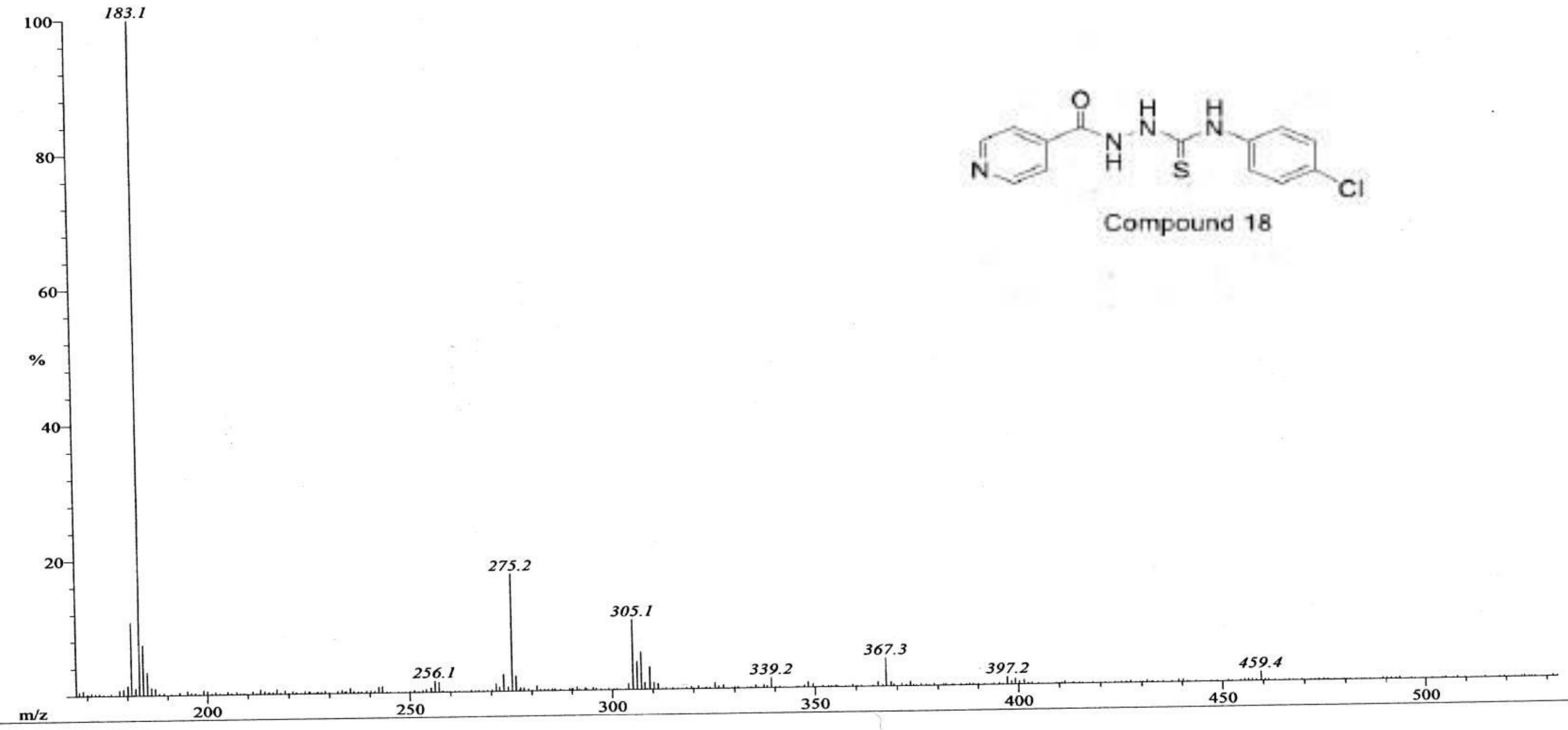

Compound 18

I. C.C.B.S., University of Ka[illegible]hi Analytical Laboratory - Pakistan

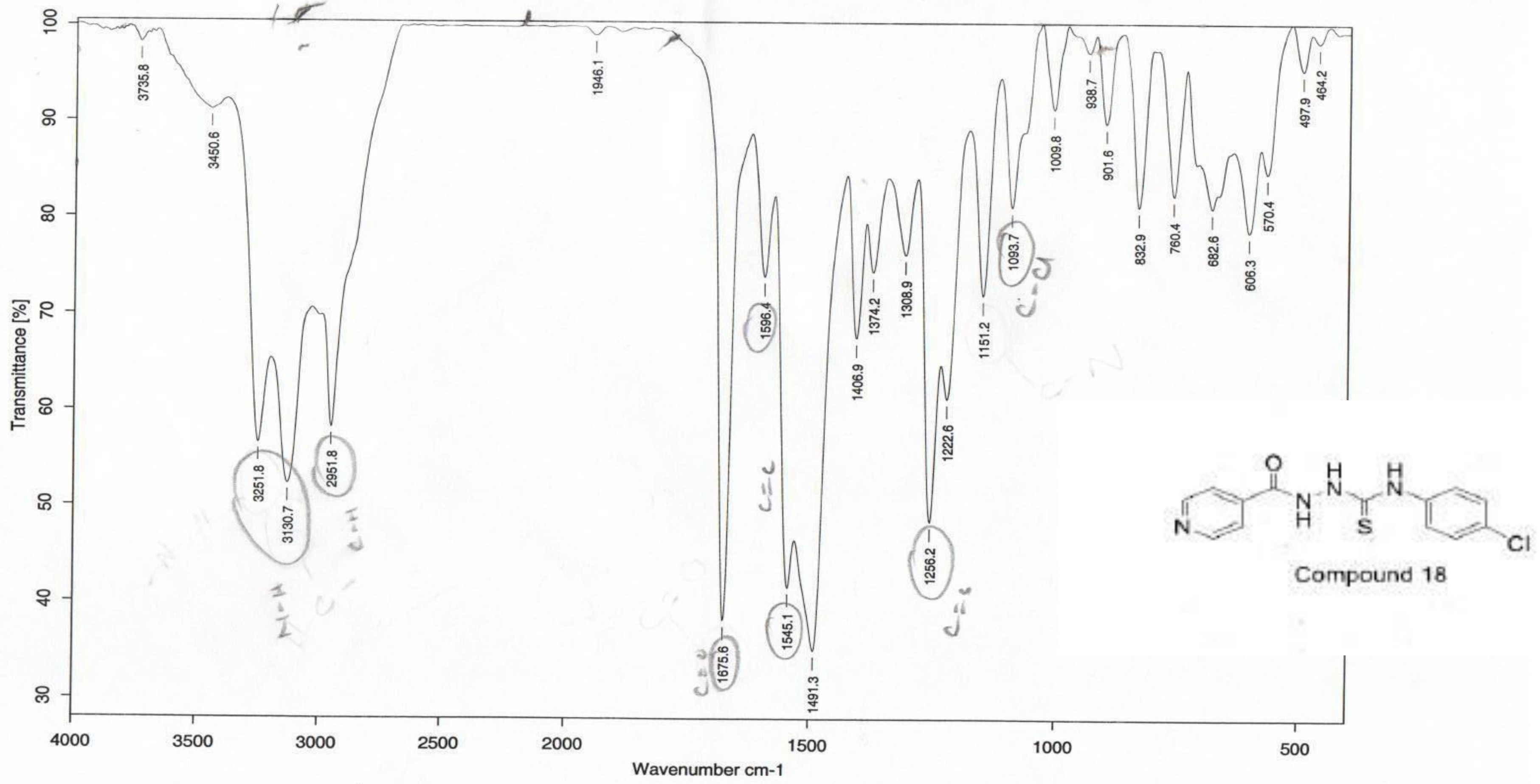

Compound 18

Sample : Fz-I-Iso23/Fazila Rizvi/Dr. Hina

Measured : 29/12/2016 on VECTOR22

Resolution : 4 cm-1 (10 scans)

Spectrum : Fz-I-Iso23.0 (in D:\IRSTUDENT)

Technic : Solid

Analyst : M. Asif

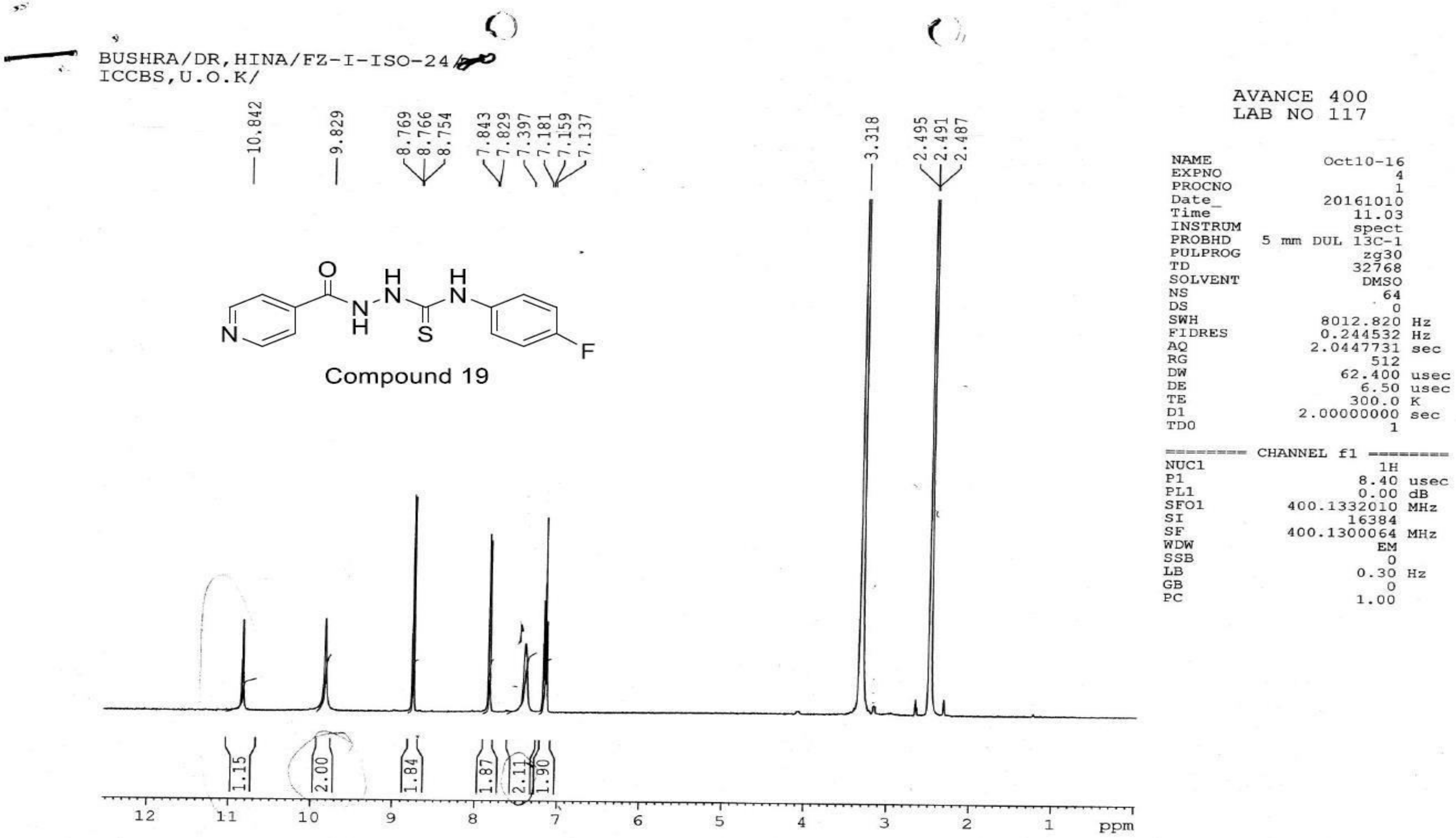
BUSHRA/DR,HINA/FZ-I-ISO-24
ICCBS,U.O.K/
10.842
9.829
8.769
8.766
8.754
7.843
7.829
7.397
7.181
7.159
7.137
3.318
2.495
2.491
2.487
Compound 19
AVANCE 400
LAB NO 117
NAME Oct10-16
EXPNO 4
PROCNO 1
Date_ 20161010
Time 11.03
INSTRUM spect
PROBHD 5 mm DUL 13C-1
PULPROG zg30
TD 32768
SOLVENT DMSO
NS 64
DS 0
SWH 8012.820 Hz
FIDRES 0.244532 Hz
AQ 2.0447731 sec
RG 512
DW 62.400 usec
DE 6.50 usec
TE 300.0 K
D1 2.00000000 sec
TD0 1
======== CHANNEL f1 ========
NUC1 1H
P1 8.40 usec
PL1 0.00 dB
SFO1 400.1332010 MHz
SI 16384
SF 400.1300064 MHz
WDW EM
SSB 0
LB 0.30 Hz
GB 0
PC 1.00
1.15
2.00
1.84
1.87
2.11
1.90
12
11
10
9
8
7
6
5
4
3
2
1
ppm

File: FZ-I-ISO24-FABN
Sample: BUSHRA QAMAR /DR. HINA
Instrument: JEOL-600H-2
Inlet: Direct Probe

Date Run: 10-19-2016 (Time Run: 15:36:52)

Ionization mode: FAB-

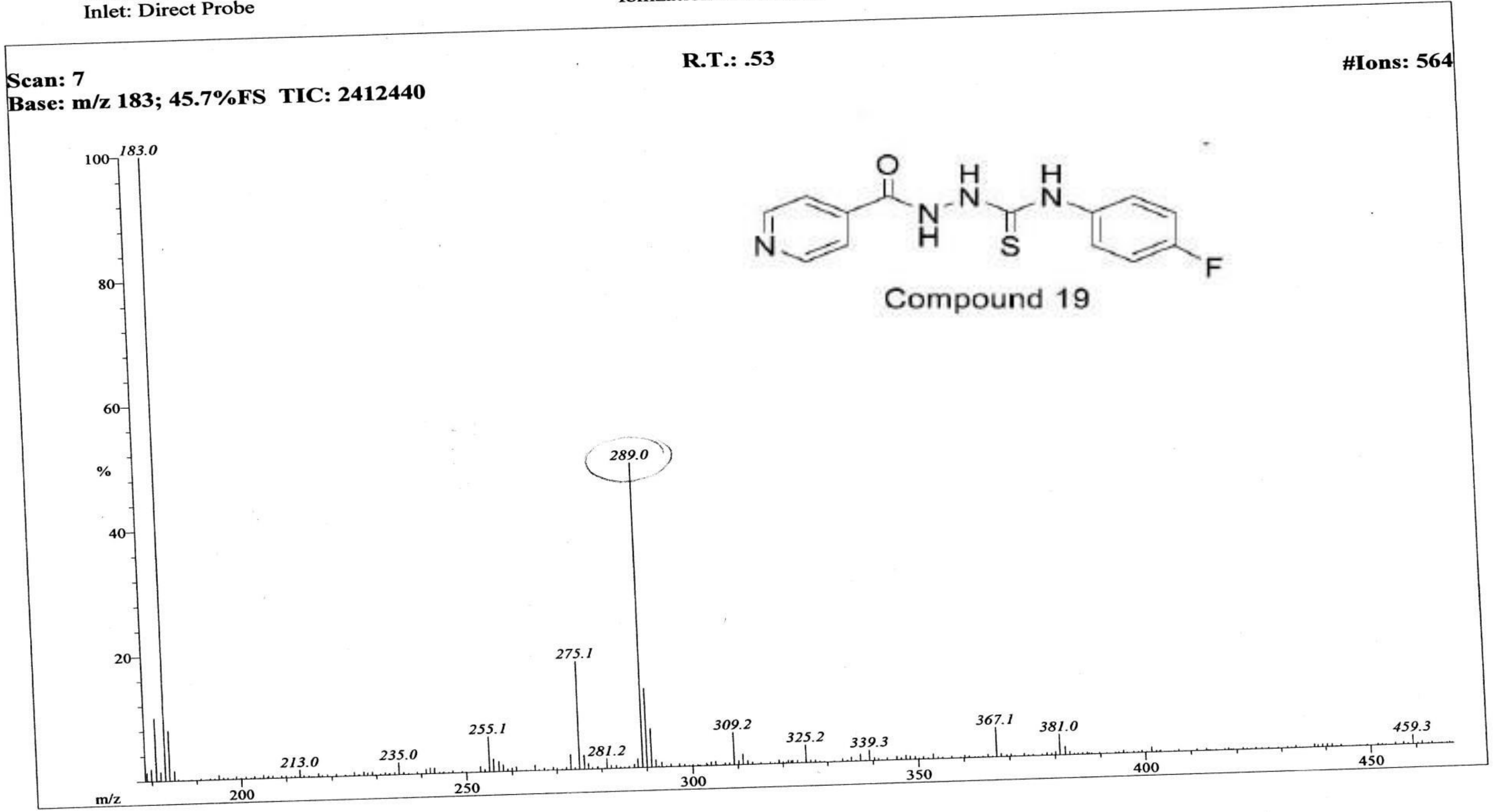

I. C.C.B.S., University of Ka[illegible]hi Analytical Laboratory - Pakistan

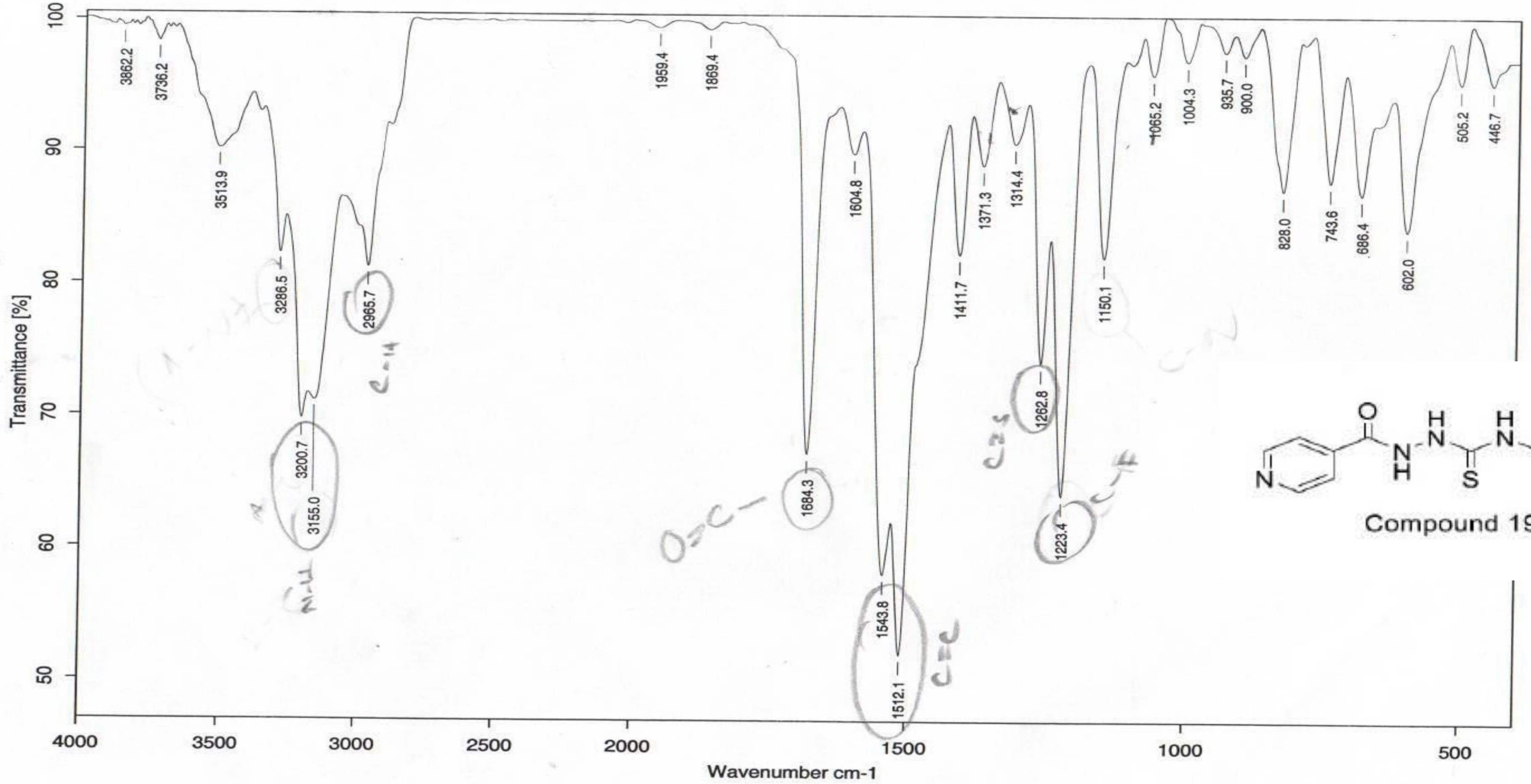

Compound 19

Sample : Fz-I-Iso24/Fazila Rizvi/Dr. Hina

Measured : 29/12/2016 on VECTOR22

Resolution : 4 cm-1 (10 scans)

Spectrum : Fz-I-Iso24.0 (in D:\IRSTUDENT)

Technic : Solid

Analyst : M. Asif

BUSHRA/DR,HINA/FZ-I-ISO-25/
ICCBS,U.O.K/

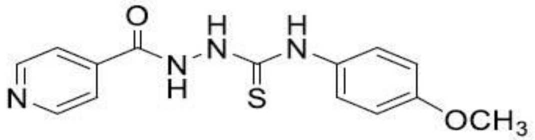

Compound 20

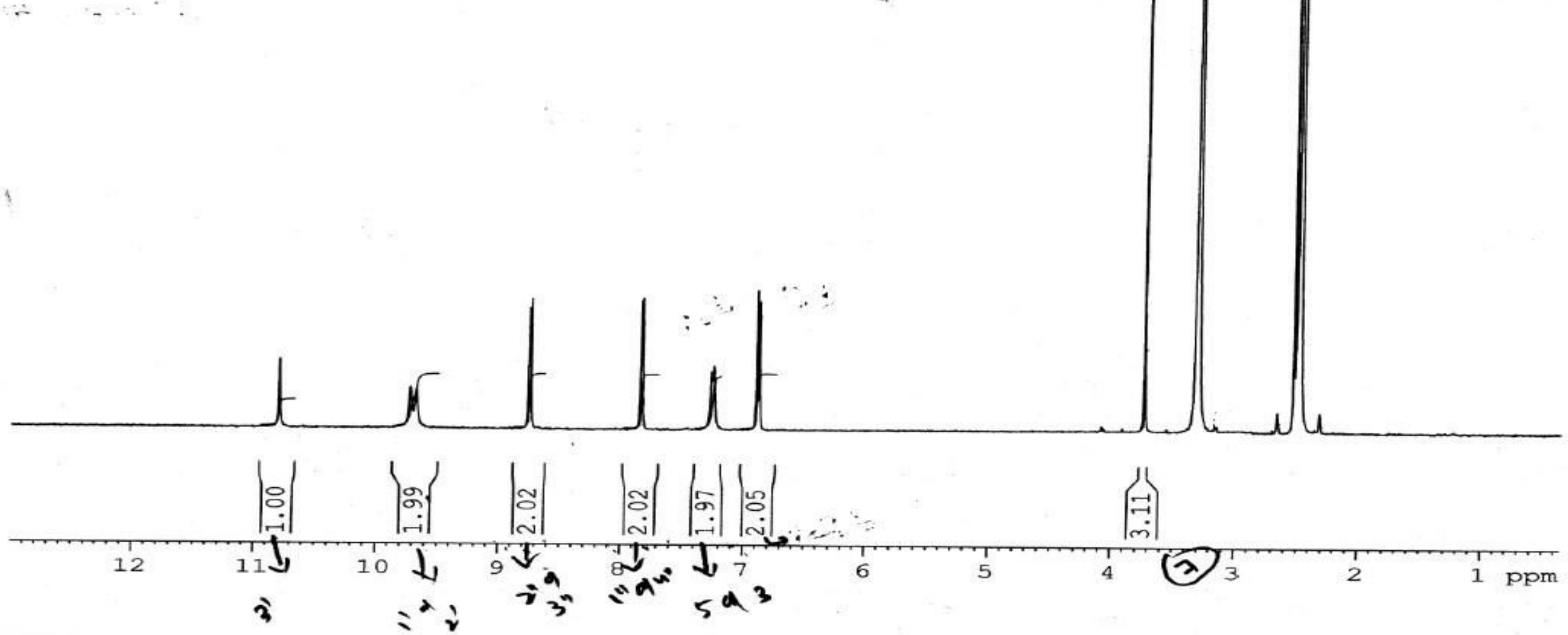

AVANCE 400
LAB NO 117

NAME	nov01-16	
EXPNO	2	
PROCNO	1	
Date_	20161101	
Time	11.07	
INSTRUM	spect	
PROBHD	5 mm DUL 13C-1	
PULPROG	zg30	
TD	32768	
SOLVENT	DMSO	
NS	64	
DS	0	
SWH	8012.820	Hz
FIDRES	0.244532	Hz
AQ	2.0447731	sec
RG	512	
DW	62.400	usec
DE	6.50	usec
TE	300.0	K
D1	2.00000000	sec
TD0	1	

======== CHANNEL f1 ========

NUC1	1H	
P1	8.40	usec
PL1	0.00	dB
SFO1	400.1332010	MHz
SI	16384	
SF	400.1300064	MHz
WDW	EM	
SSB	0	
LB	0.30	Hz
GB	0	
PC	1.00	

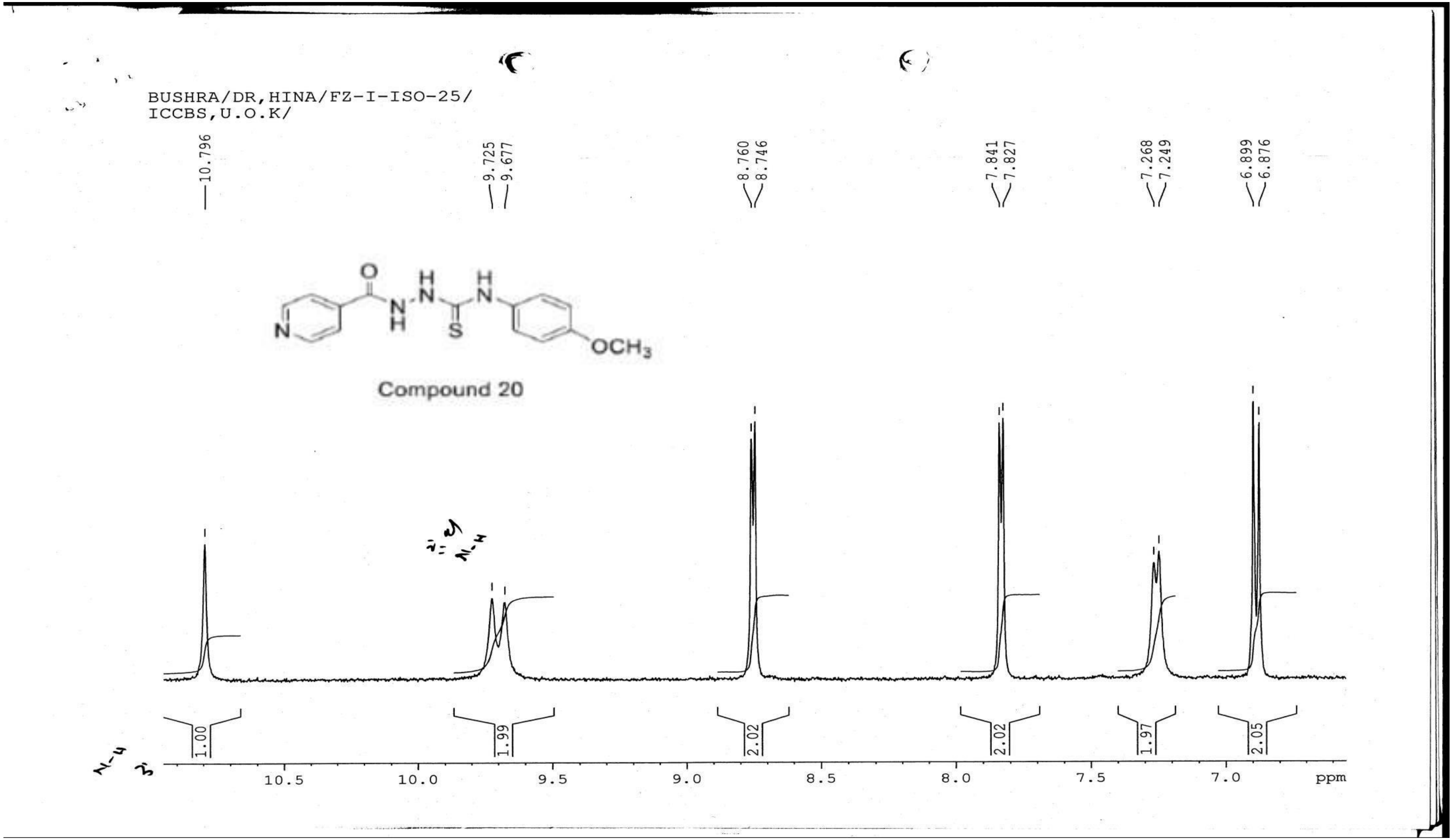
BUSHRA/DR,HINA/FZ-I-ISO-25/
ICCBS,U.O.K/
10.796
9.725
9.677
8.760
8.746
7.841
7.827
7.268
7.249
6.899
6.876
Compound 20
1.00
1.99
2.02
2.02
1.97
2.05
10.5
10.0
9.5
9.0
8.5
8.0
7.5
7.0
ppm

HEJ-ICCBS
10/18/2016 3:18:34 PM

File: FZ-I-ISO25-FABP
Sample: BUSHRA QAMAR /DR. HINA
Instrument: JEOL-600H-2
Inlet: Direct Probe

Date Run: 10-18-2016 (Time Run: 15:16:35)

Ionization mode: FAB+

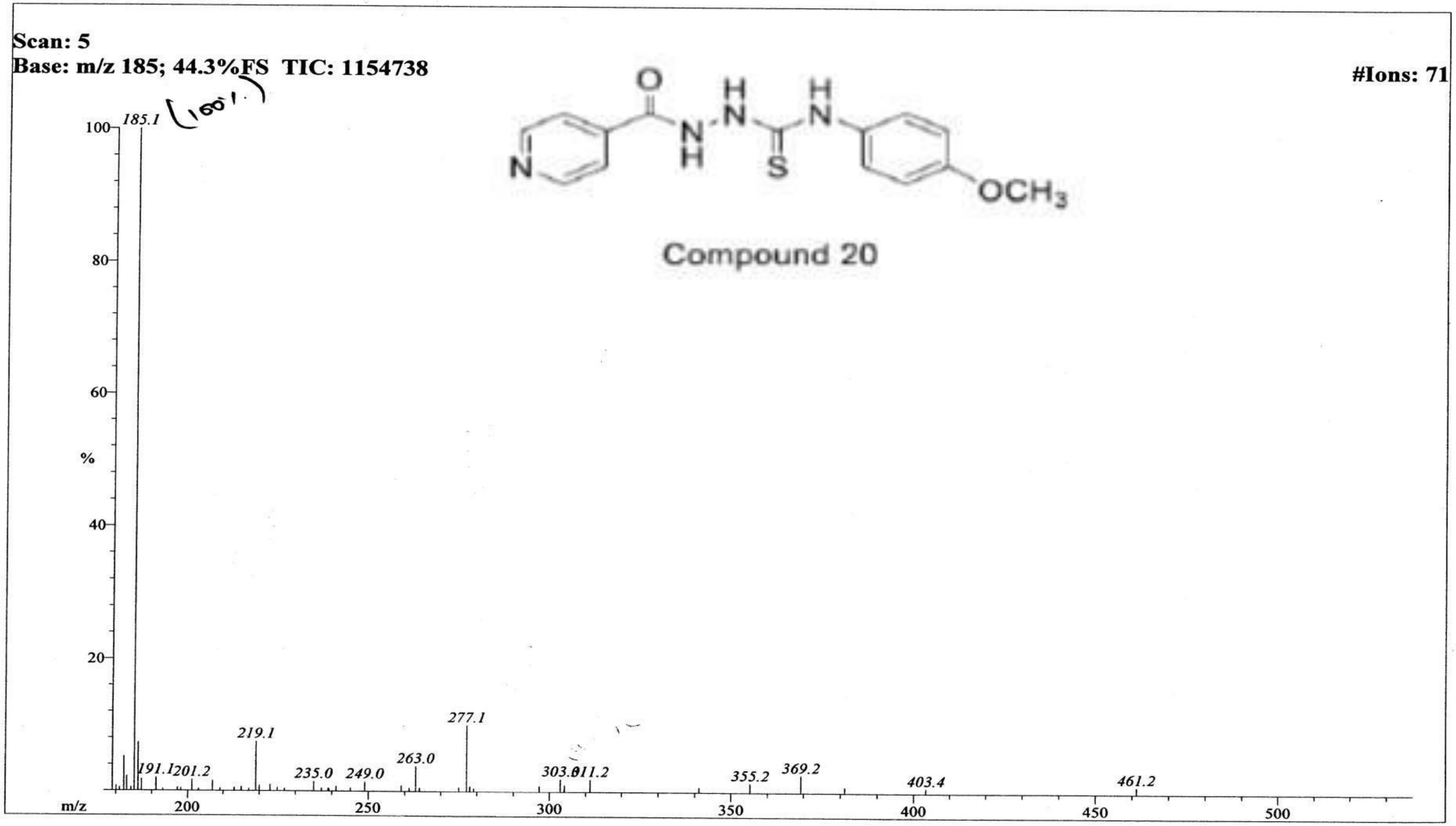

I. C.C.B.S., University of Ka[illegible]hi Analytical Laboratory - Pakistan

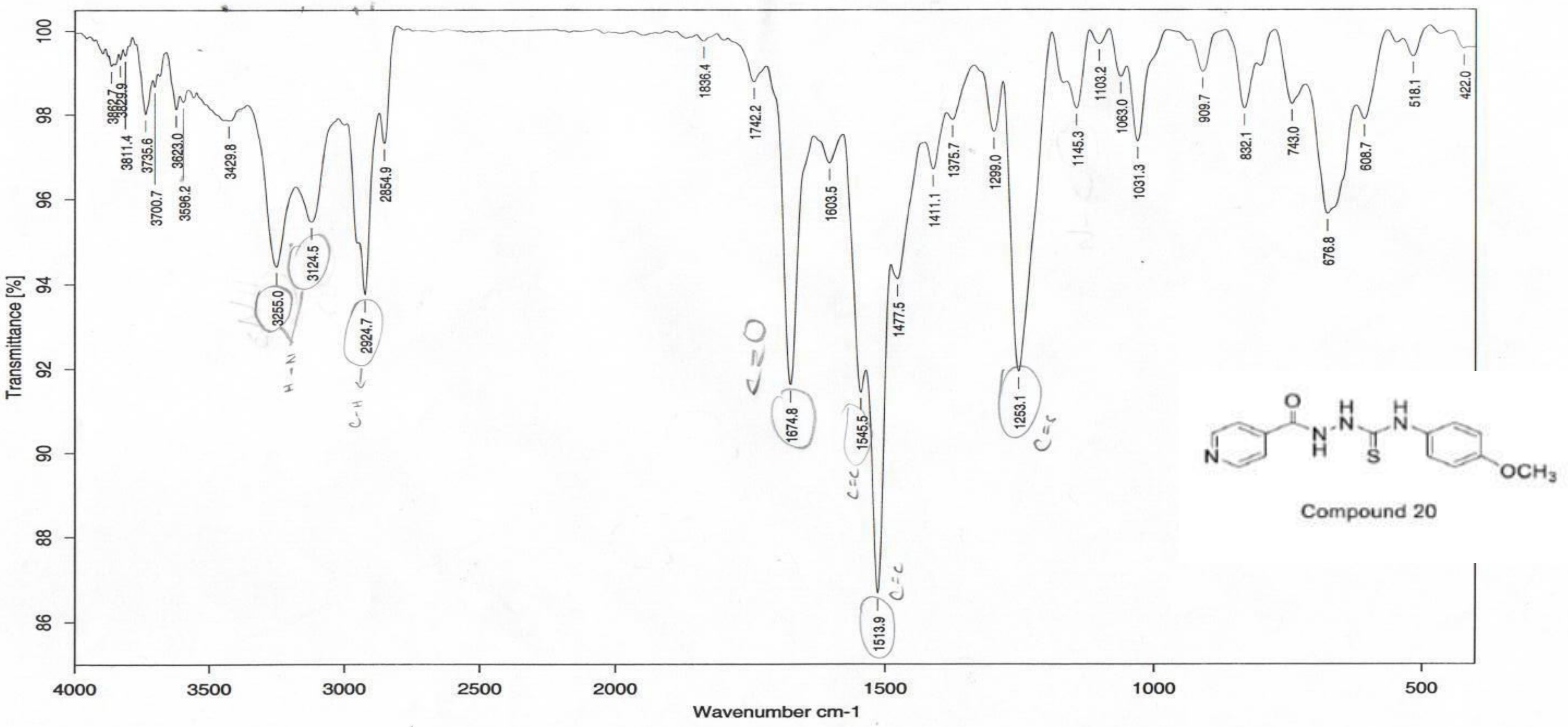

Sample : Fz-I-Iso25/Fazila Rizvi/Dr. Hina	Spectrum : Fz-I-Iso25.0 (in D:\IRSTUDENT)
Measured : 29/12/2016 on VECTOR22	Technic : Solid
Resolution : 4 cm-1 (10 scans)	Analyst : M. Asif

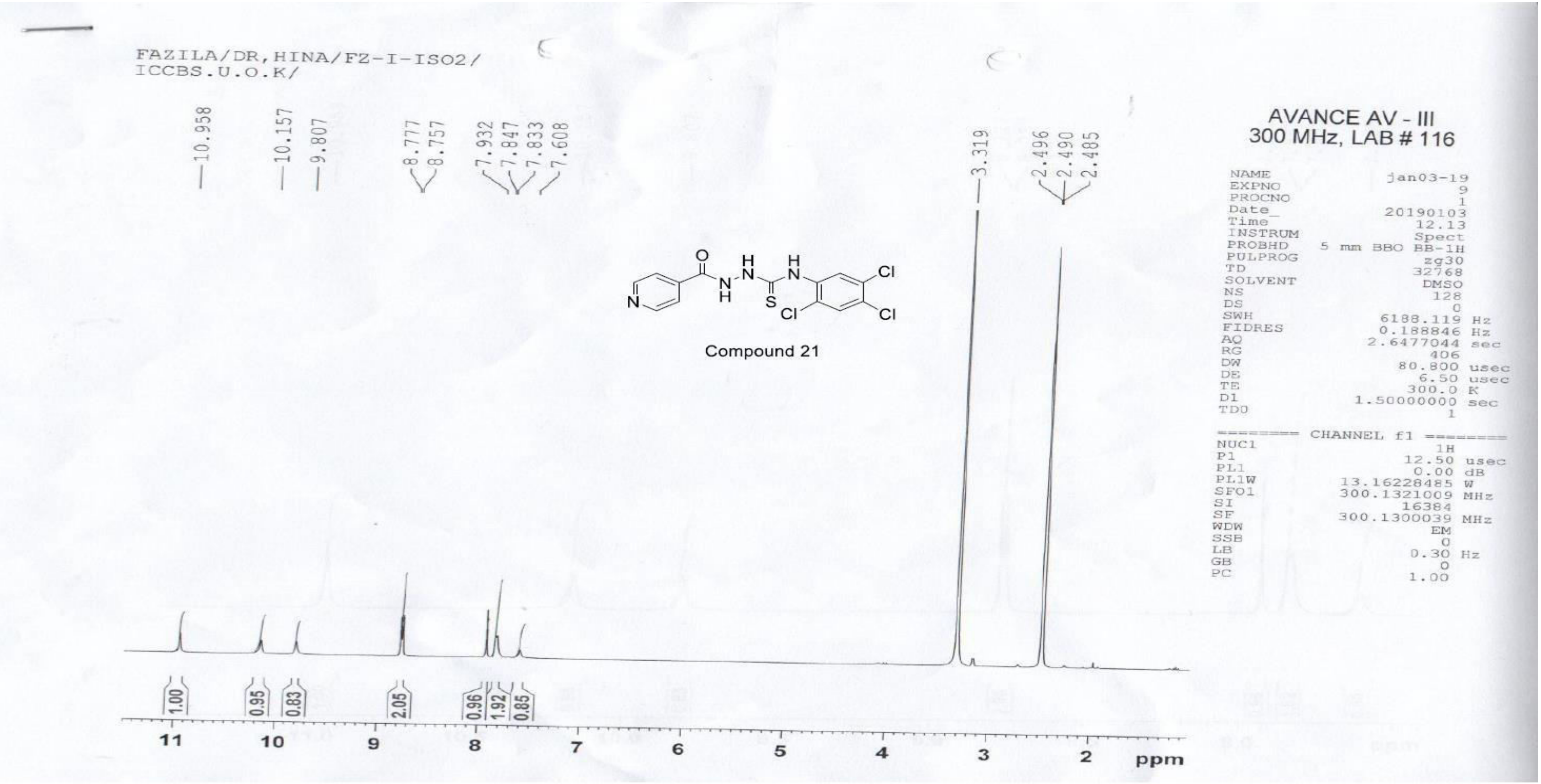
FAZILA/DR,HINA/FZ-I-ISO2/
ICCBS.U.O.K/
10.958
10.157
9.807
8.777
8.757
7.932
7.847
7.833
7.608
3.319
2.496
2.490
2.485
Compound 21
AVANCE AV - III
300 MHz, LAB # 116
NAME jan03-19
EXPNO 9
PROCNO 1
Date_ 20190103
Time 12.13
INSTRUM Spect
PROBHD 5 mm BBO BB-1H
PULPROG zg30
TD 32768
SOLVENT DMSO
NS 128
DS 0
SWH 6188.119 Hz
FIDRES 0.188846 Hz
AQ 2.6477044 sec
RG 406
DW 80.800 usec
DE 6.50 usec
TE 300.0 K
D1 1.50000000 sec
TD0 1
======== CHANNEL f1 ========
NUC1 1H
P1 12.50 usec
PL1 0.00 dB
PL1W 13.16228485 W
SFO1 300.1321009 MHz
SI 16384
SF 300.1300039 MHz
WDW EM
SSB 0
LB 0.30 Hz
GB 0
PC 1.00
1.00
0.95
0.83
2.05
0.96
1.92
0.85
11
10
9
8
7
6
5
4
3
2
ppm

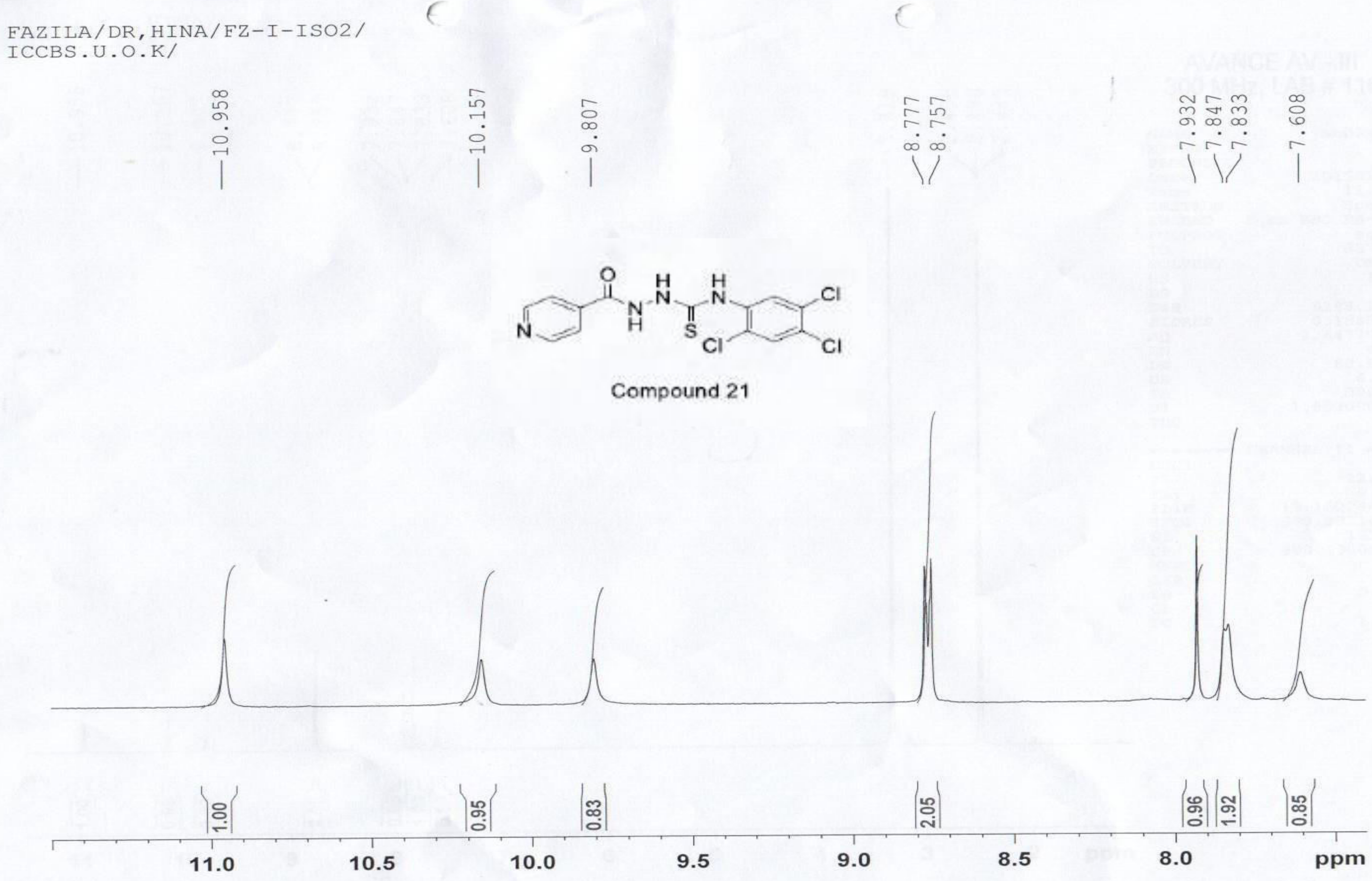
FAZILA/DR,HINA/FZ-I-ISO2/
ICCBS.U.O.K/
10.958
10.157
9.807
8.777
8.757
7.932
7.847
7.833
7.608
O
H
H
N
N
N
N
H
S
Cl
Cl
Cl
Compound 21
1.00
0.95
0.83
2.05
0.96
1.92
0.85
11.0
10.5
10.0
9.5
9.0
8.5
8.0
ppm

File: FZ-I-ISO2(F)-FABN
Sample: FAZILA /DR. HINA
Instrument: JEOL-600H-2
Inlet: Direct Probe

Date Run: 04-09-2016 (Time Run: 13:50:04)

Ionization mode: FAB-

Scan: 9
Base: m/z 183; 100%FS TIC: 5396524

R.T.: .72

#Ions: 834

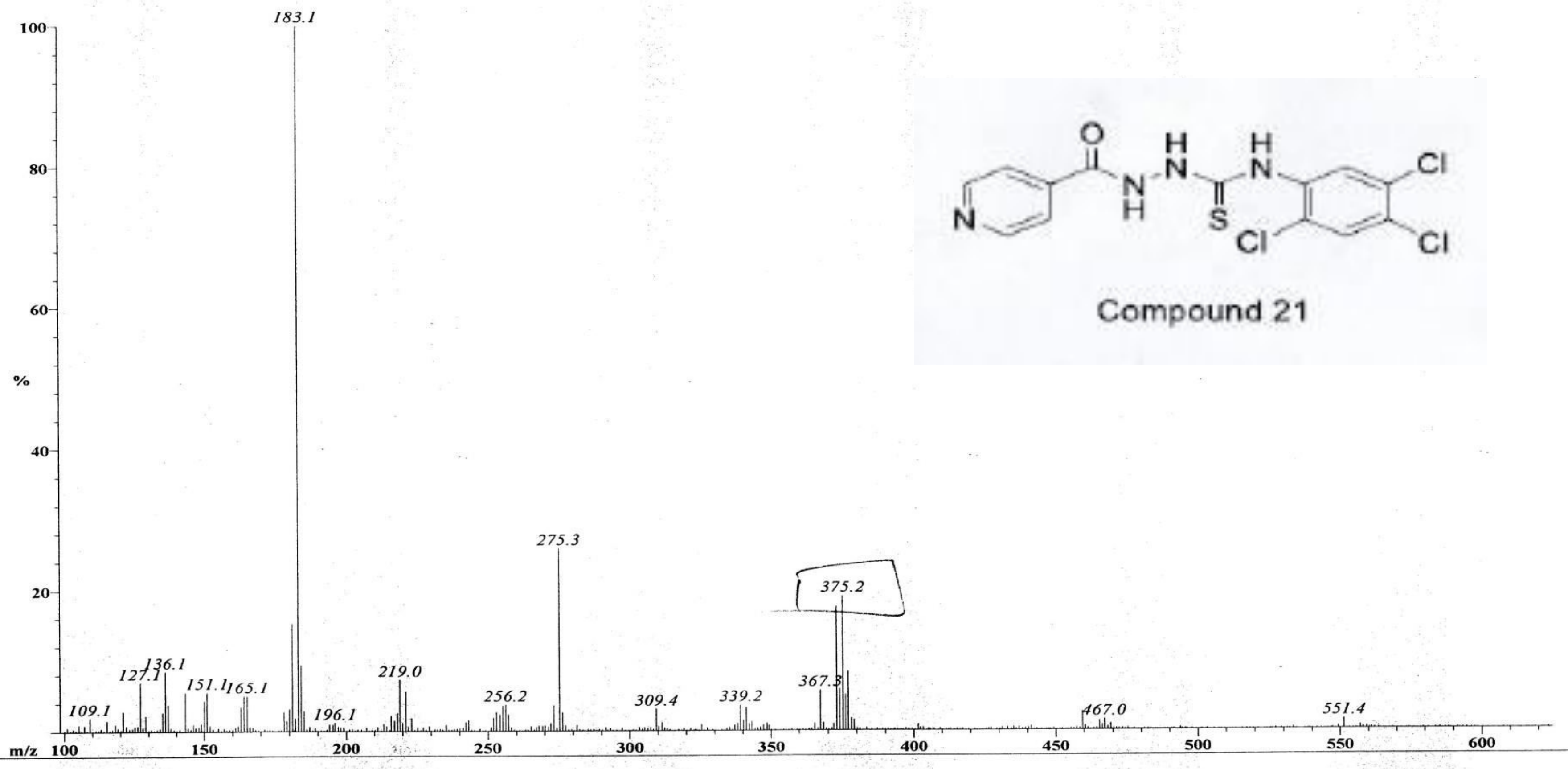

Compound 21

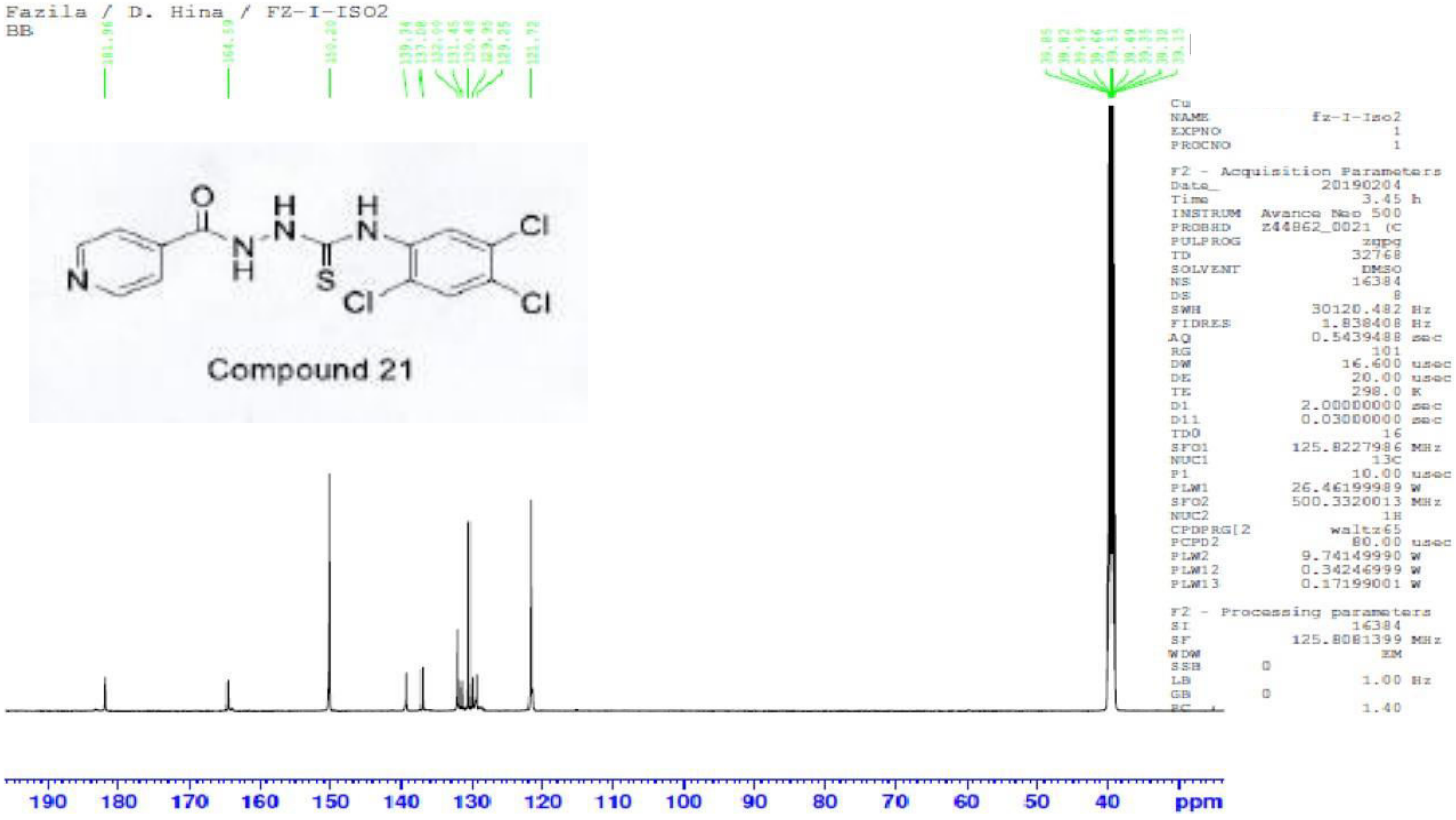
Fazila / D. Hina / FZ-I-ISO2
BB
O
H
H
N
N
N
Cl
N
H
S
Cl
Cl
Compound 21
Cu
NAME fz-I-Iso2
EXPNO 1
PROCNO 1
F2 - Acquisition Parameters
Date_ 20190204
Time 3.45 h
INSTRUM Avance Neo 500
PROBHD Z44862_0021 (C
PULPROG zgpg
TD 32768
SOLVENT DMSO
NS 16384
DS 8
SWH 30120.482 Hz
FIDRES 1.838408 Hz
AQ 0.5439488 sec
RG 101
DW 16.600 usec
DE 20.00 usec
TE 298.0 K
D1 2.00000000 sec
D11 0.03000000 sec
TD0 16
SFO1 125.8227986 MHz
NUC1 13C
P1 10.00 usec
PLW1 26.46199989 W
SFO2 500.3320013 MHz
NUC2 1H
CPDPRG[2 waltz65
PCPD2 80.00 usec
PLW2 9.74149990 W
PLW12 0.34246999 W
PLW13 0.17199001 W
F2 - Processing parameters
SI 16384
SF 125.8081399 MHz
WDW EM
SSB 0
LB 1.00 Hz
GB 0
PC 1.40
190
180
170
160
150
140
130
120
110
100
90
80
70
60
50
40
ppm

Fazila / D. Hina / FZ-I-ISO2
DEPT135

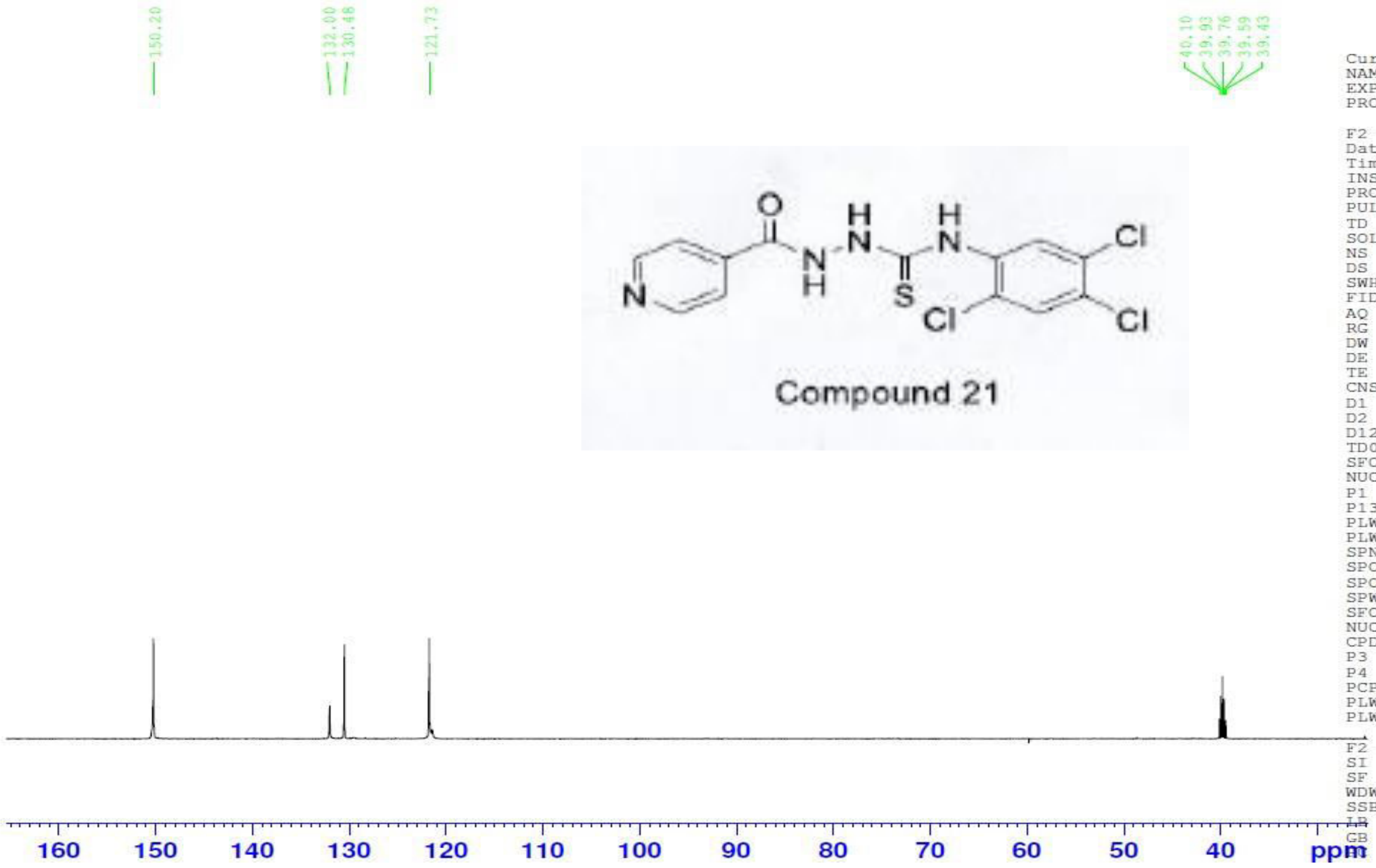

Current Data Parameters
NAME fz-I-Iso2
EXPNO 2
PROCNO 1

F2 - Acquisition Parameters
Date_ 20190204
Time 8.47 h
INSTRUM Avance Neo 500
PROBHD Z44862_0021 (C
PULPROG deptsp135
TD 32768
SOLVENT DMSO
NS 8233
DS 8
SWH 25000.000 Hz
FIDRES 1.525879 Hz
AQ 0.6553600 sec
RG 101
DW 20.000 usec
DE 20.00 usec
TE 298.0 K
CNST2 145.0000000
D1 1.50000000 sec
D2 0.00344828 sec
D12 0.00002000 sec
TD0 10
SFO1 125.8200303 MHz
NUC1 13C
P1 10.00 usec
P13 2000.00 usec
PLW0 0 W
PLW1 26.46199989 W
SPNAM[5] Crp60comp.4
SPOAL5 0.500
SPOFFS5 0 Hz
SPW5 4.04309988 W
SFO2 500.3320013 MHz
NUC2 1H
CPDPRG[2 waltz65
P3 15.00 usec
P4 30.00 usec
PCPD2 80.00 usec
PLW2 9.74149990 W
PLW12 0.34246999 W

F2 - Processing parameters
SI 16384
SF 125.8081395 MHz
WDW EM
SSB 0
LB 1.00 Hz
GB 0
PC 1.40

I. C.C.B.S., University of Ka[illegible]hi Analytical Laboratory - Pakistan

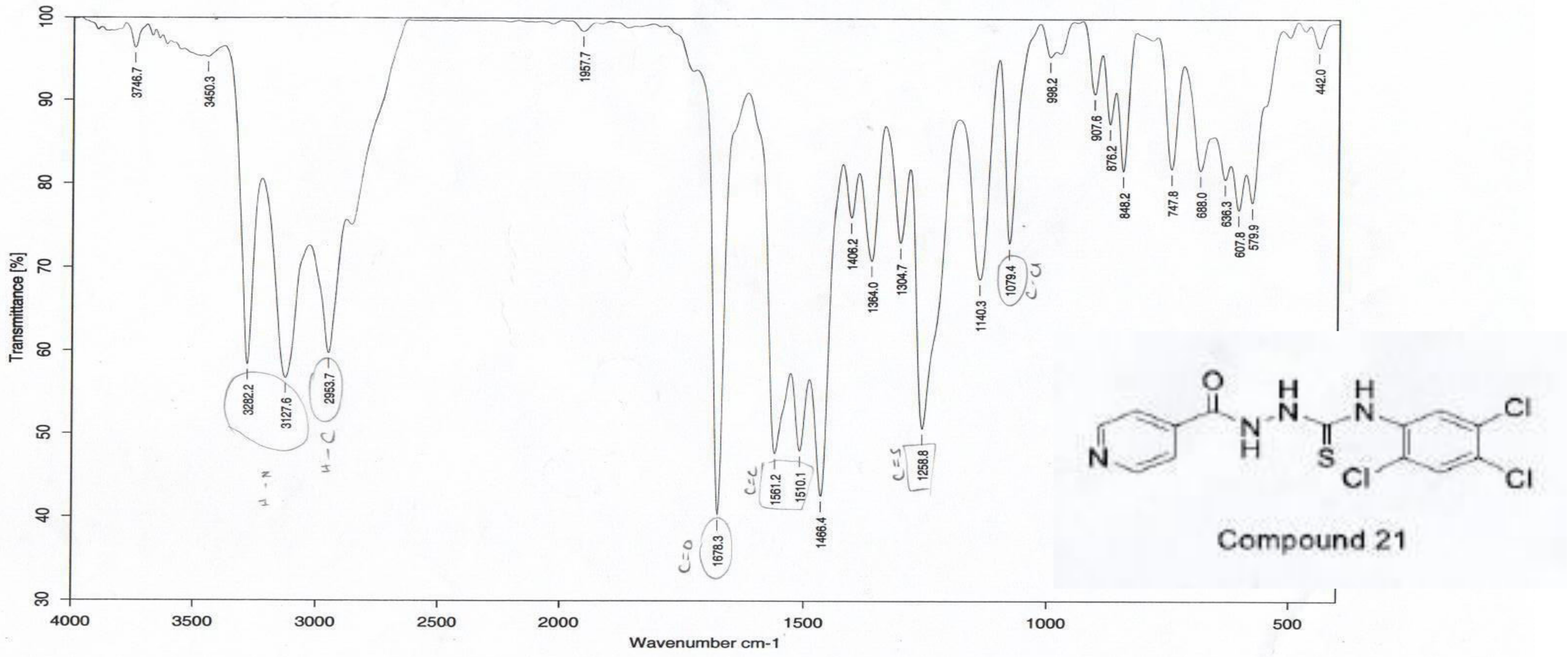

Sample : FZI-ISO2/Fazila Rizvi	
Measured : 19/04/2017 on VECTOR22	Spectrum : FZ-I-ISO2.0 (in D:\IRSTUDENT)
	Technic : Solid
Resolution : 4 cm-1 (10 scans)	Analyst : MA/ZA/JS

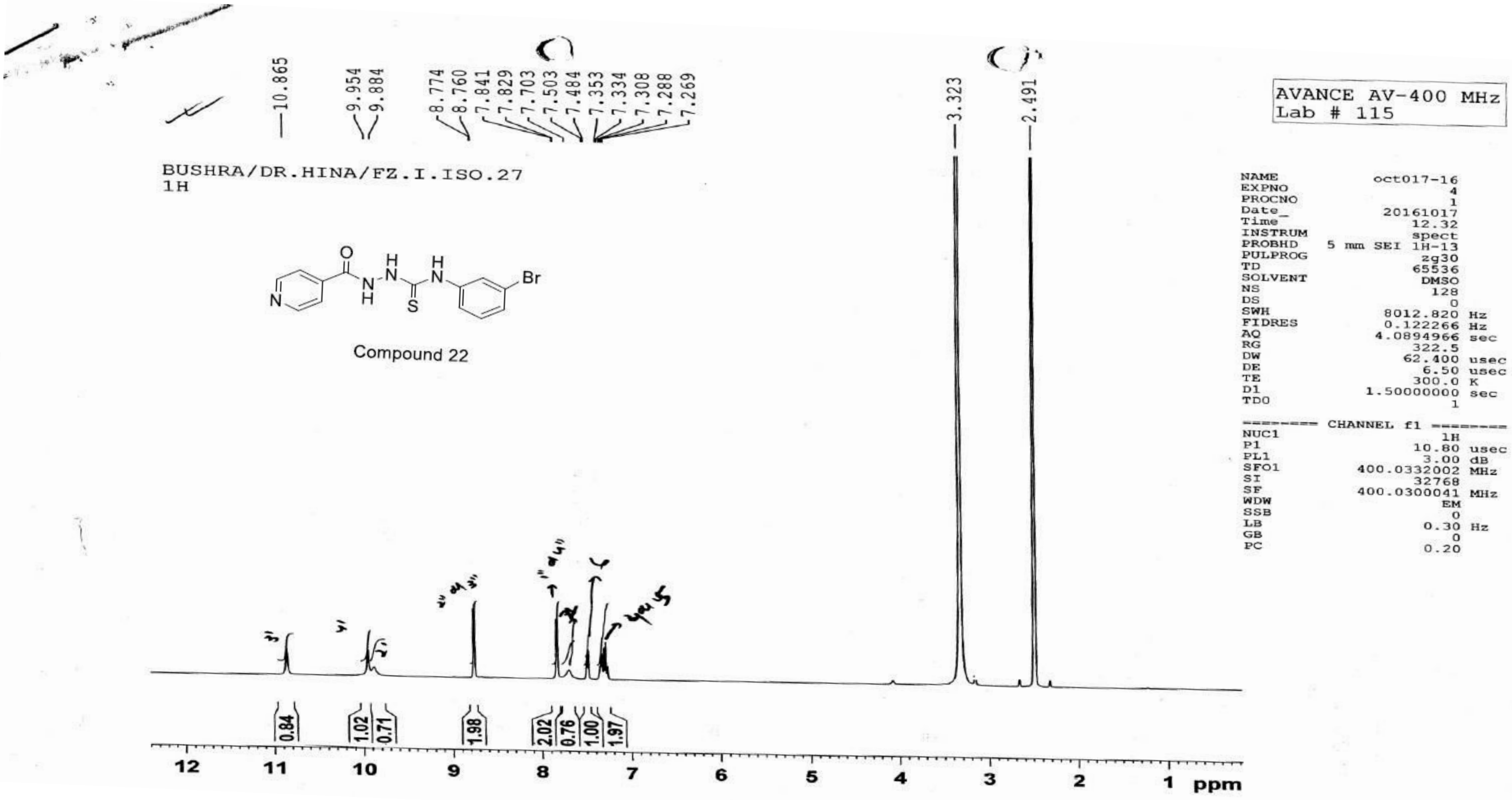

BUSHRA/DR.HINA/FZ.I.ISO.27
1H
Compound 22
AVANCE AV-400 MHz
Lab # 115
10.865
9.954
9.884
8.774
8.760
7.841
7.829
7.703
7.503
7.484
7.353
7.334
7.308
7.288
7.269
3.323
2.491
0.84
1.02
0.71
1.98
2.02
0.76
1.00
1.97
12
11
10
9
8
7
6
5
4
3
2
1
ppm
NAME oct017-16
EXPNO 4
PROCNO 1
Date_ 20161017
Time 12.32
INSTRUM spect
PROBHD 5 mm SEI 1H-13
PULPROG zg30
TD 65536
SOLVENT DMSO
NS 128
DS 0
SWH 8012.820 Hz
FIDRES 0.122266 Hz
AQ 4.0894966 sec
RG 322.5
DW 62.400 usec
DE 6.50 usec
TE 300.0 K
D1 1.50000000 sec
TD0 1
======= CHANNEL f1 =======
NUC1 1H
P1 10.80 usec
PL1 3.00 dB
SFO1 400.0332002 MHz
SI 32768
SF 400.0300041 MHz
WDW EM
SSB 0
LB 0.30 Hz
GB 0
PC 0.20

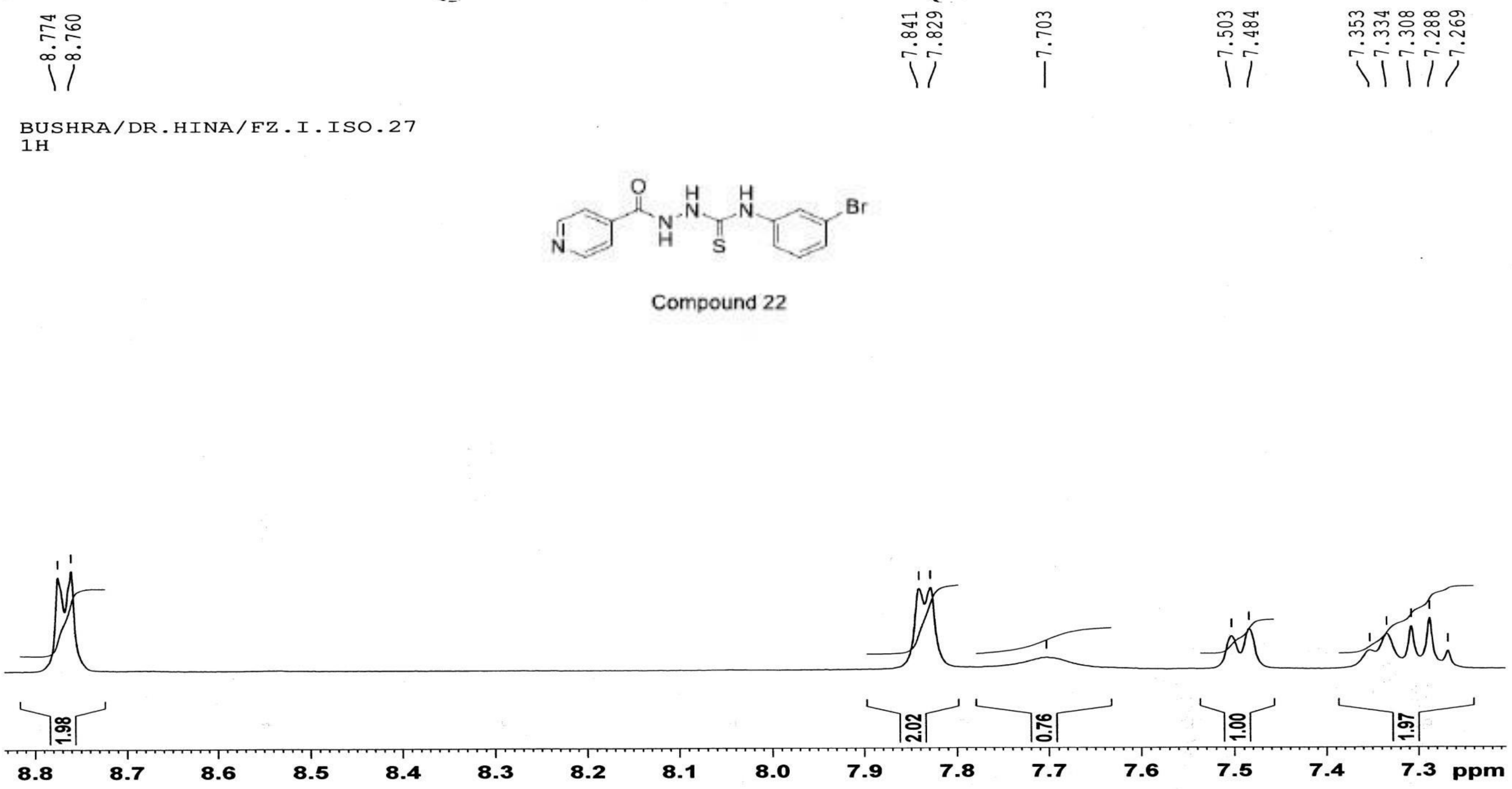
8.774
8.760
7.841
7.829
7.703
7.503
7.484
7.353
7.334
7.308
7.288
7.269
BUSHRA/DR.HINA/FZ.I.ISO.27
1H
O
H
N
H
N
N
H
S
Br
N
1.98
2.02
0.76
1.00
1.97
8.8 8.7 8.6 8.5 8.4 8.3 8.2 8.1 8.0 7.9 7.8 7.7 7.6 7.5 7.4 7.3 ppm

Compound 22

HEJ-ICCBS
10/19/2016 4:15:47 PM

File: FZ-I-ISO27-FABN
Sample: BUSHRA QAMAR /DR. HINA
Instrument: JEOL-600H-2
Inlet: Direct Probe

Date Run: 10-19-2016 (Time Run: 16:13:01)

Ionization mode: FAB-

Scan: 8
Base: m/z 183; 56.2%FS TIC: 2067620

R.T.: .62

#Ions: 462

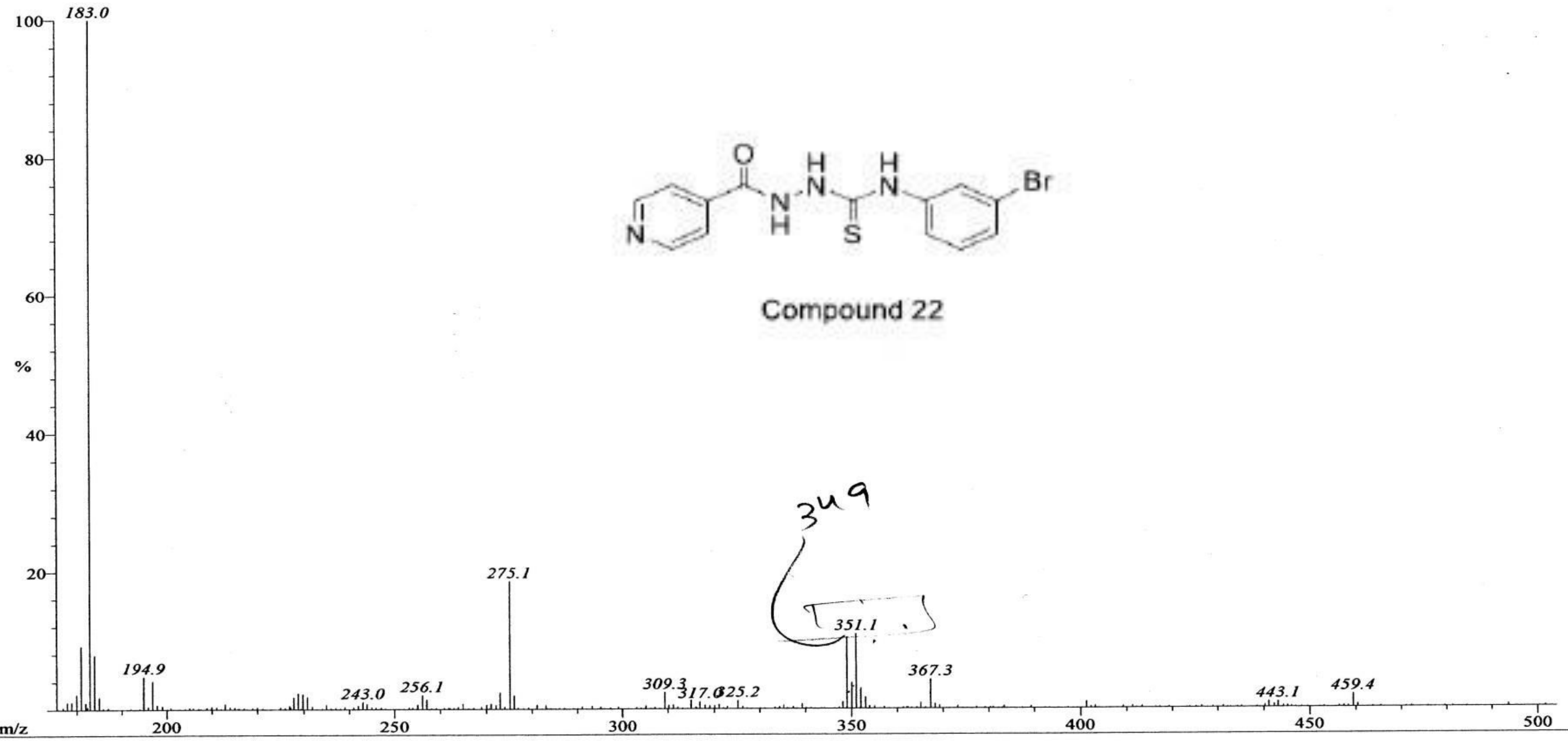

Compound 22

I. C.C.B.S., University of Ka[illegible]hi Analytical Laboratory - Pakistan

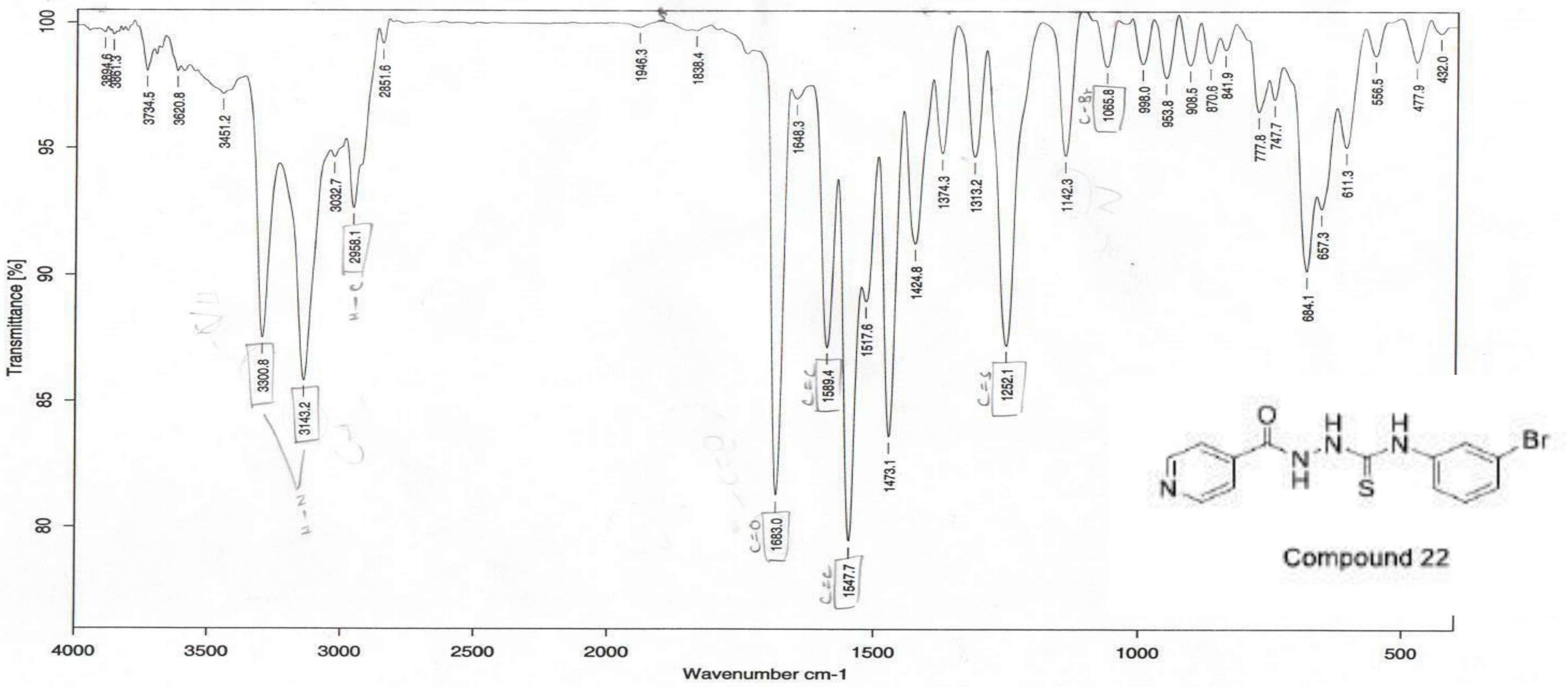

Compound 22

Sample :	Fz-I-Iso27/Fazila Rizvi/Dr. Hina	Spectrum	: Fz-I-Iso27.0 (in D:\IRSTUDENT)
Measured :	29/12/2016 on VECTOR22	Technic	: Solid
Resolution :	4 cm-1 (10 scans)	Analyst	: M. Asif

fazila rizvi/Dr.Hina/Fz-I-iso28
1H

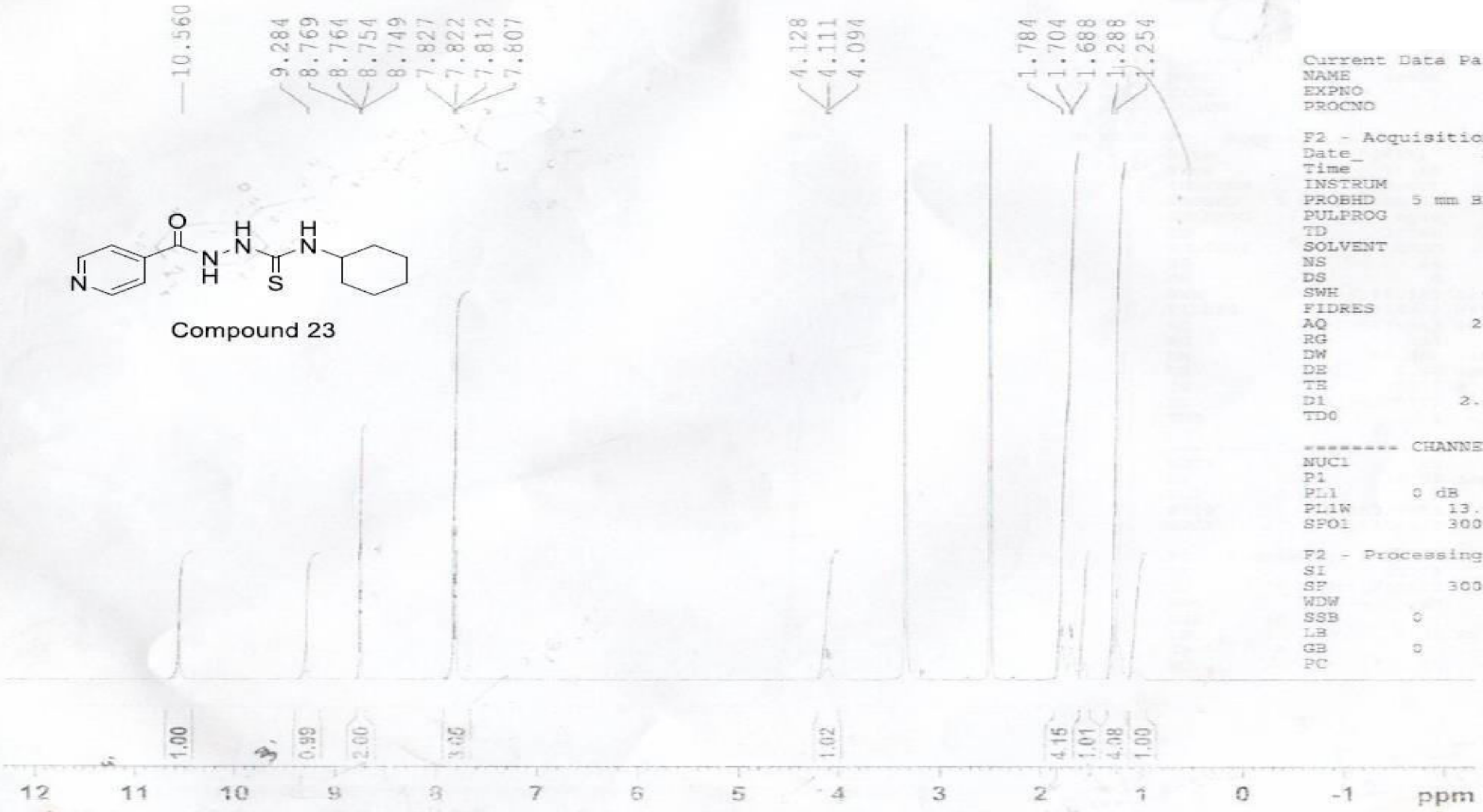

Compound 23

Current Data Parameters
NAME iso28
EXPNO 5
PROCNO 1

F2 - Acquisition Parameters
Date_ 20161117
Time 11.48
INSTRUM Spect
PROBHD 5 mm BBO BB-1H
PULPROG zg30
TD 32768
SOLVENT DMSO
NS 64
DS 0
SWH 6188.119 Hz
FIDRES 0.188846 Hz
AQ 2.6476543 sec
RG 203
DW 80.800 usec
DE 6.50 usec
TE 300.0 K
D1 2.00000000 sec
TD0 1

======== CHANNEL f1 ========
NUC1 1H
P1 12.50 usec
PL1 0 dB
PL1W 13.16228485 W
SFO1 300.1324010 MHz

F2 - Processing parameters
SI 32768
SF 300.1300000 MHz
WDW EM
SSB 0
LB 0.30 Hz
GB 0
PC 1.00

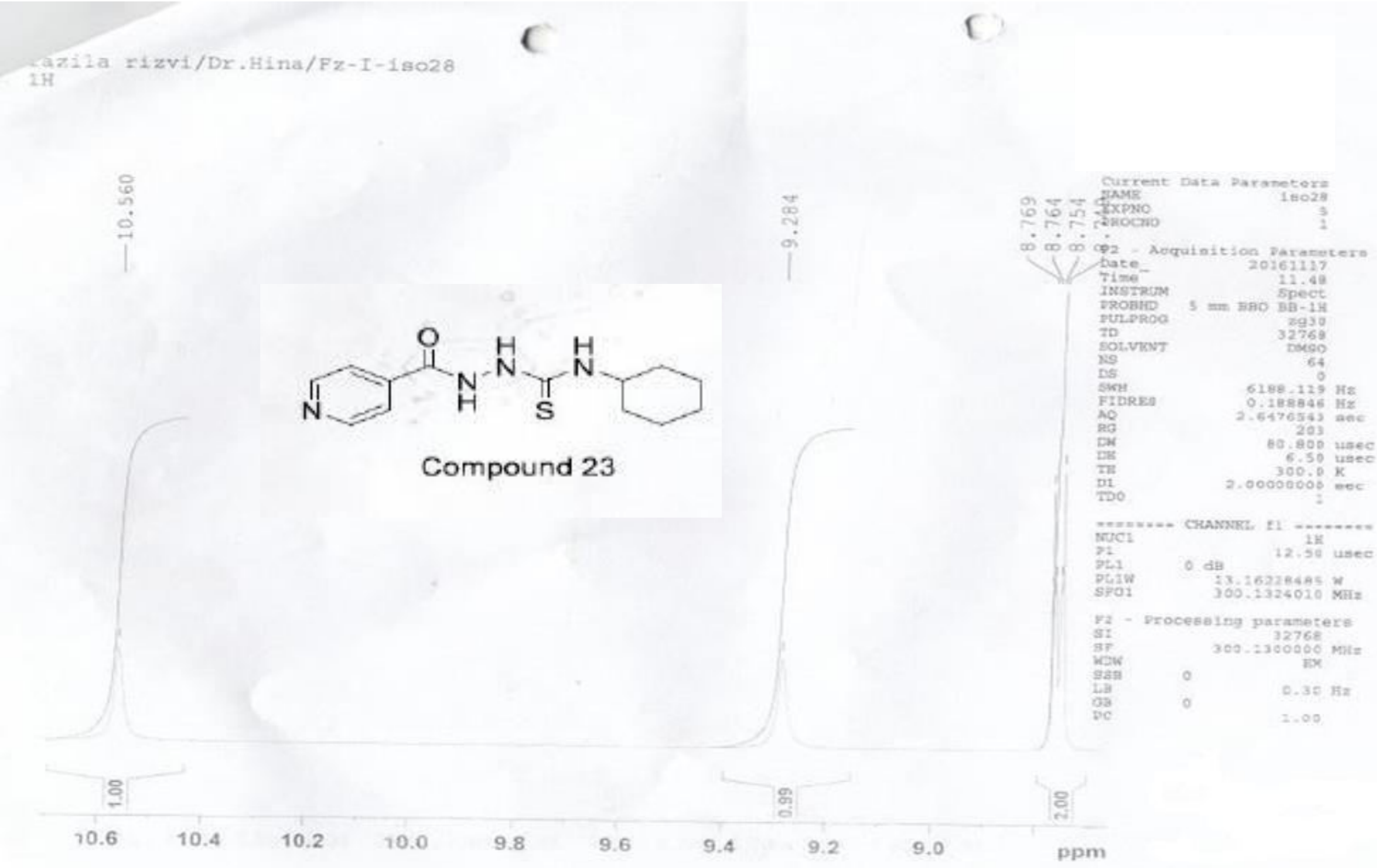
azila rizvi/Dr.Hina/Fz-I-iso28
1H
10.560
9.284
8.769
8.764
8.754
Compound 23
Current Data Parameters
NAME iso28
EXPNO 5
PROCNO 1
F2 - Acquisition Parameters
Date_ 20161117
Time 11.48
INSTRUM Spect
PROBHD 5 mm BBO BB-1H
PULPROG zg30
TD 32768
SOLVENT DMSO
NS 64
DS 0
SWH 6188.119 Hz
FIDRES 0.188846 Hz
AQ 2.6476543 sec
RG 203
DW 80.800 usec
DE 6.50 usec
TE 300.0 K
D1 2.00000000 sec
TD0 1
======== CHANNEL f1 ========
NUC1 1H
P1 12.50 usec
PL1 0 dB
PL1W 13.16228485 W
SFO1 300.1324010 MHz
F2 - Processing parameters
SI 32768
SF 300.1300000 MHz
WDW EM
SSB 0
LB 0.30 Hz
GB 0
PC 1.00
1.00
0.99
2.00
10.6
10.4
10.2
10.0
9.8
9.6
9.4
9.2
9.0
ppm

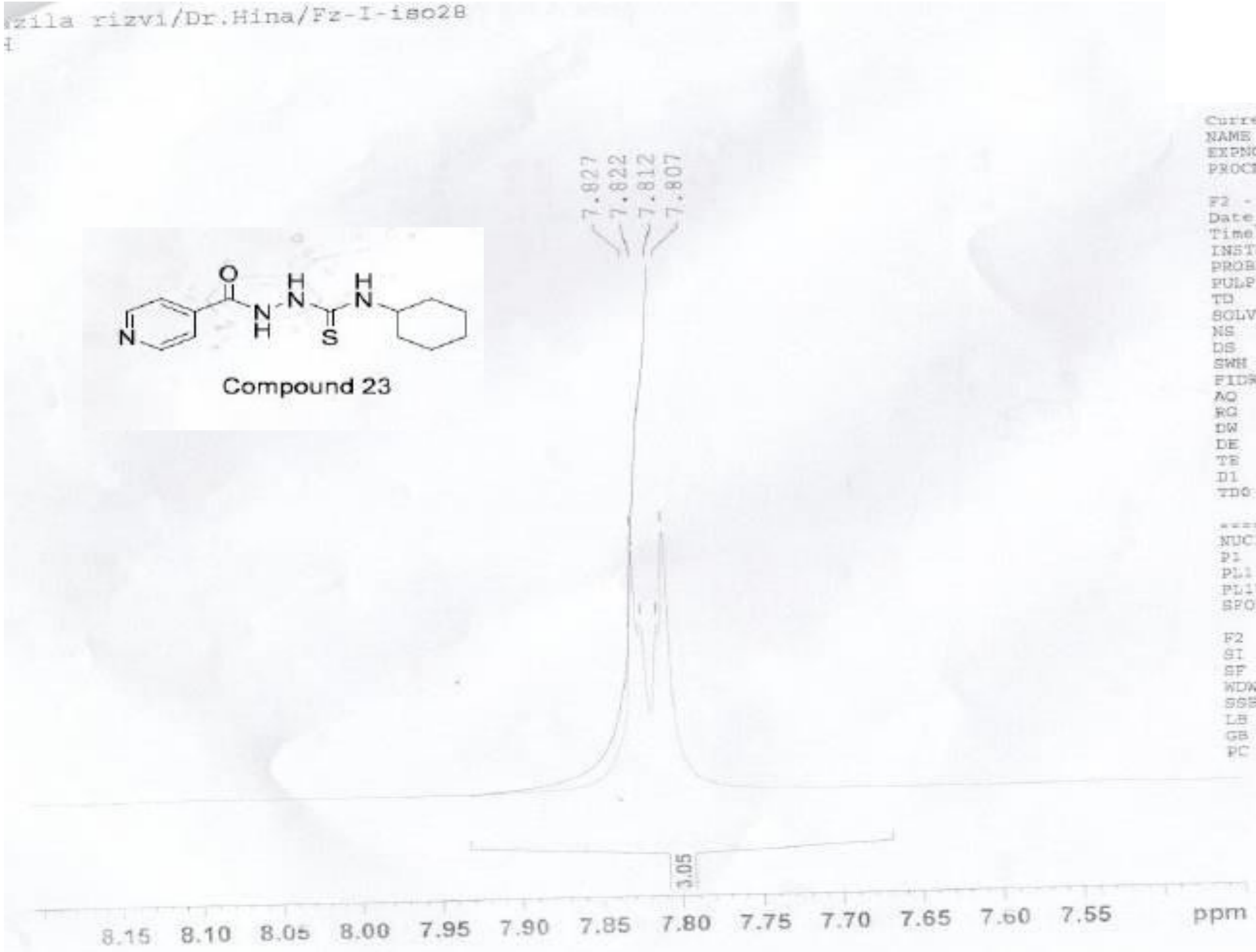

Compound 23

Current Data Parameters
NAME iso28
EXPNO 5
PROCNO 1

F2 - Acquisition Parameters
Date_ 20161117
Time 11.48
INSTRUM Spect
PROBHD 5 mm BBO BB-1H
PULPROG zg30
TD 32768
SOLVENT DMSO
NS 64
DS 0
SWH 6188.119 Hz
FIDRES 0.188846 Hz
AQ 2.6476543 sec
RG 203
DW 80.800 usec
DE 6.50 usec
TE 300.0 K
D1 2.00000000 sec
TD0 1

======== CHANNEL f1 ========
NUC1 1H
P1 12.50 usec
PL1 0 dB
PL1W 13.16228485 W
SFO1 300.1324010 MHz

F2 - Processing parameters
SI 32768
SF 300.1300000 MHz
WDW EM
SSB 0
LB 0.30 Hz
GB 0
PC 1.00

File: FZ-I-ISO28-FABN
Sample: BUSHRA QAMAR /DR. HINA
Instrument: JEOL-600H-2
Inlet: Direct Probe

Date Run: 10-19-2016 (Time Run: 16:20:39)

Ionization mode: FAB-

Scan: 10
Base: m/z 277; 47.4%FS TIC: 2426778

R.T.: .8

#Ions: 507

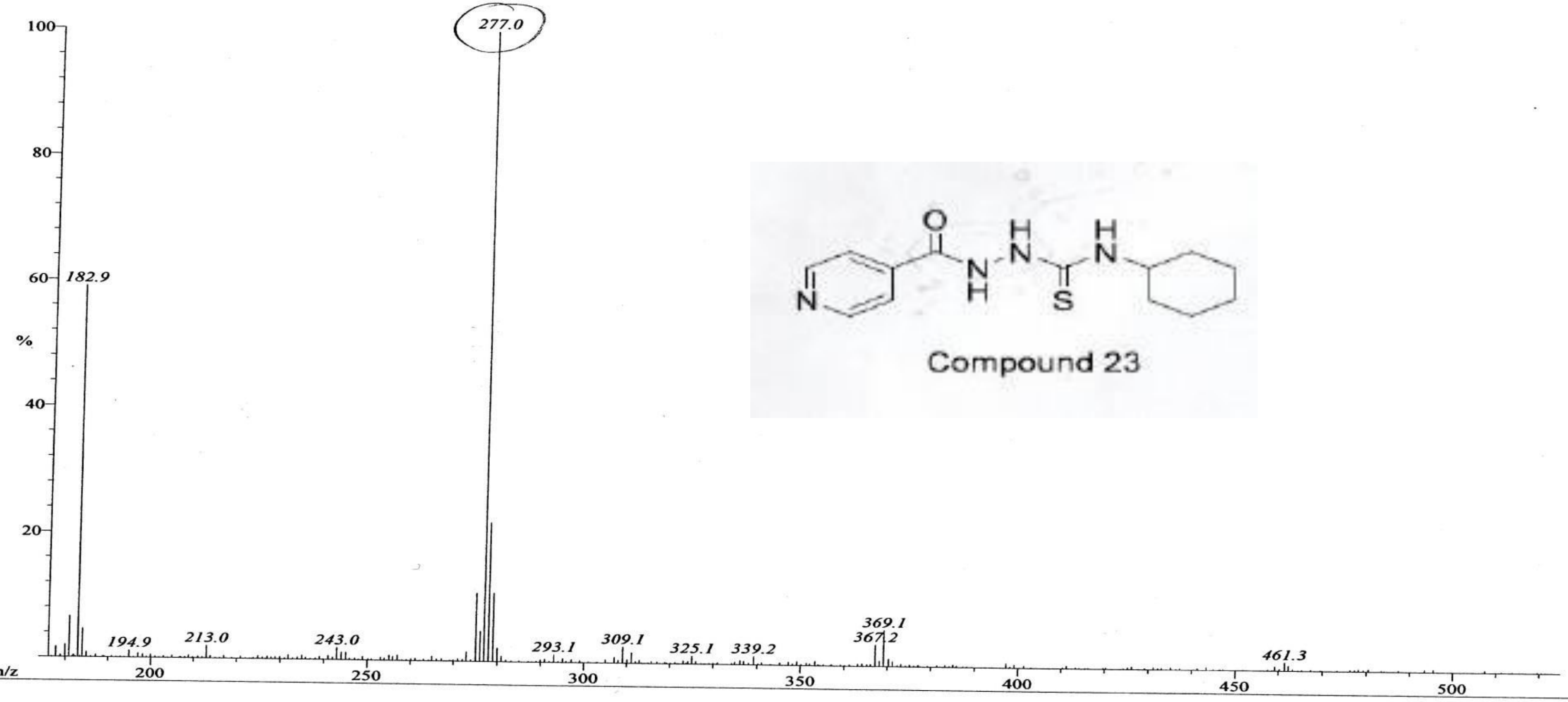

Compound 23

I. C.C.B.S., University of Ka[illegible]hi Analytical Laboratory - Pakistan

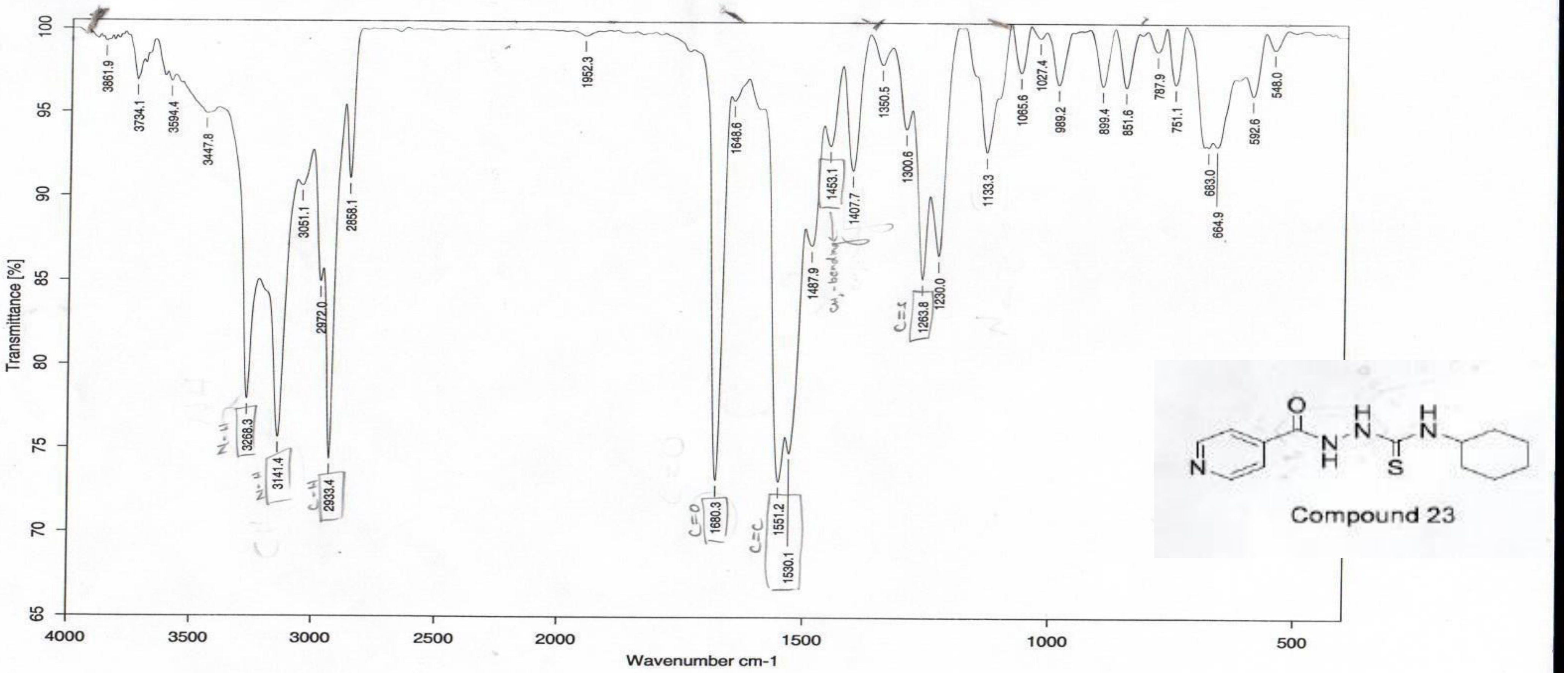

Sample : Fz-I-Iso28/Fazila Rizvi/Dr. Hina

Measured : 29/12/2016 on VECTOR22

Resolution : 4 cm-1 (10 scans)

Spectrum : Fz-I-Iso28.0 (in D:\IRSTUDENT)

Technic : Solid

Analyst : M. Asif

fazila rizvi/Dr.Hina/Fz-I-iso29
1H

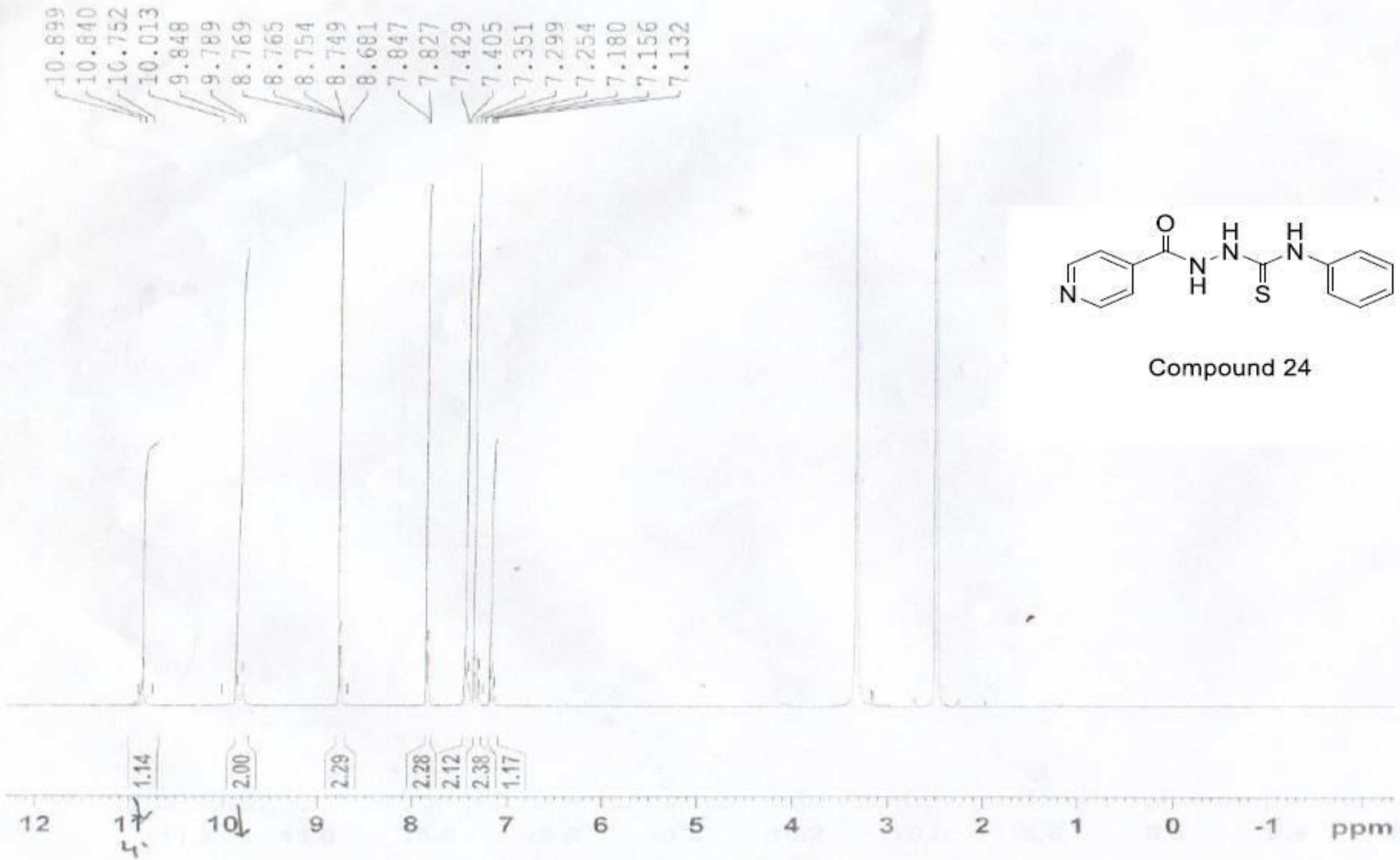

Compound 24

EXPNO 6
PROCNO 1

F2 - Acquisition Parameters
Date_ 20161117
Time 11.58
INSTRUM Spect
PROBHD 5 mm BBO BB-1H
PULPROG zg30
TD 32768
SOLVENT DMSO
NS 64
DS 0
SWH 6188.119 Hz
FIDRES 0.188846 Hz
AQ 2.6476543 sec
RG 203
DW 80.800 usec
DE 6.50 usec
TE 300.0 K
D1 2.00000000 sec
TD0 1

======== CHANNEL f1 ========
NUC1 1H
P1 12.50 usec
PL1 0 dB
PL1W 13.16228485 W
SFO1 300.1324010 MHz

F2 - Processing parameters
SI 32768
SF 300.1300040 MHz
WDW EM
SSB 0
LB 0.30 Hz
GB 0
PC 1.00

fazila rizvi/Dr.Hina/Fz-I-iso29
1H

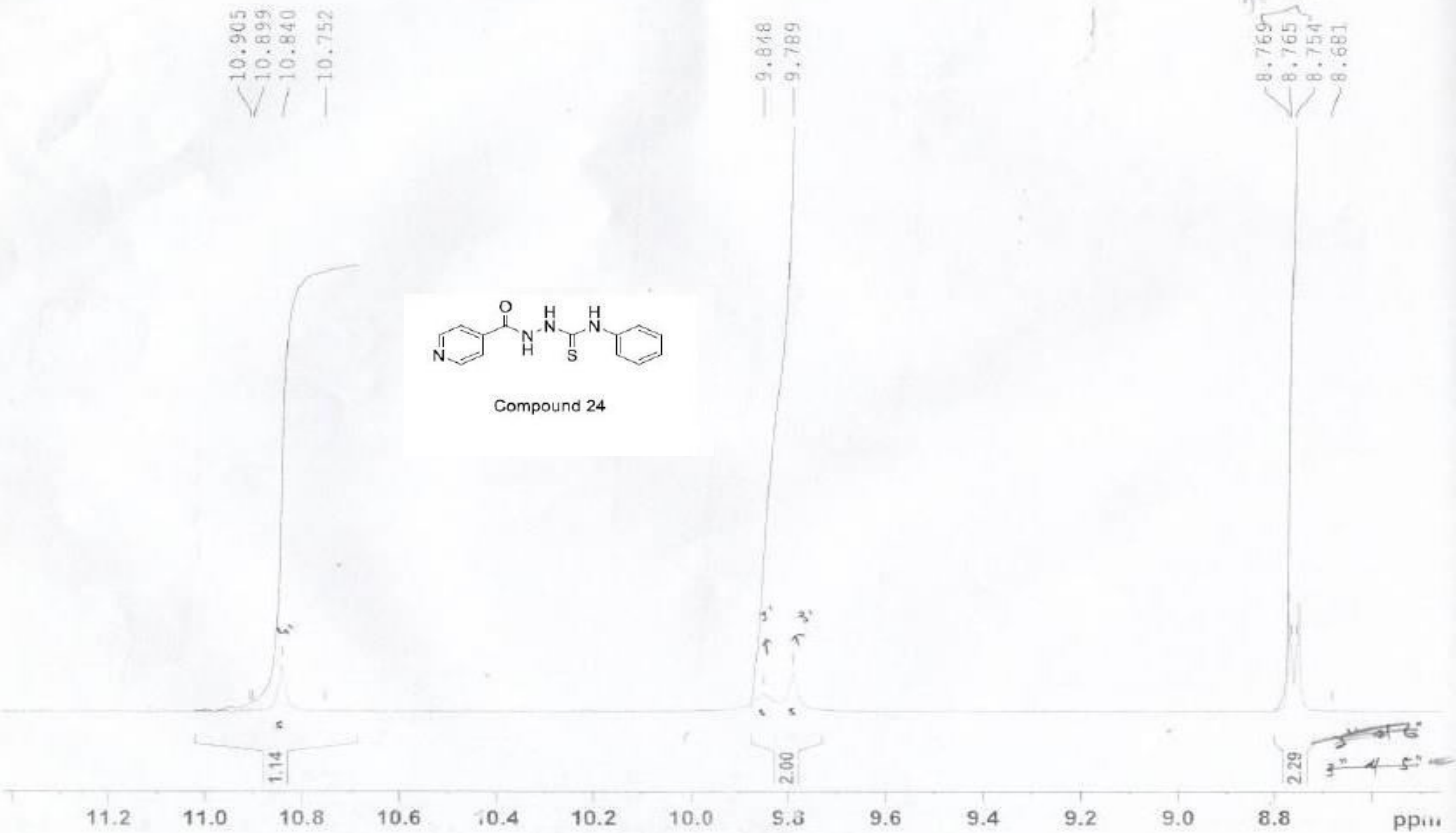

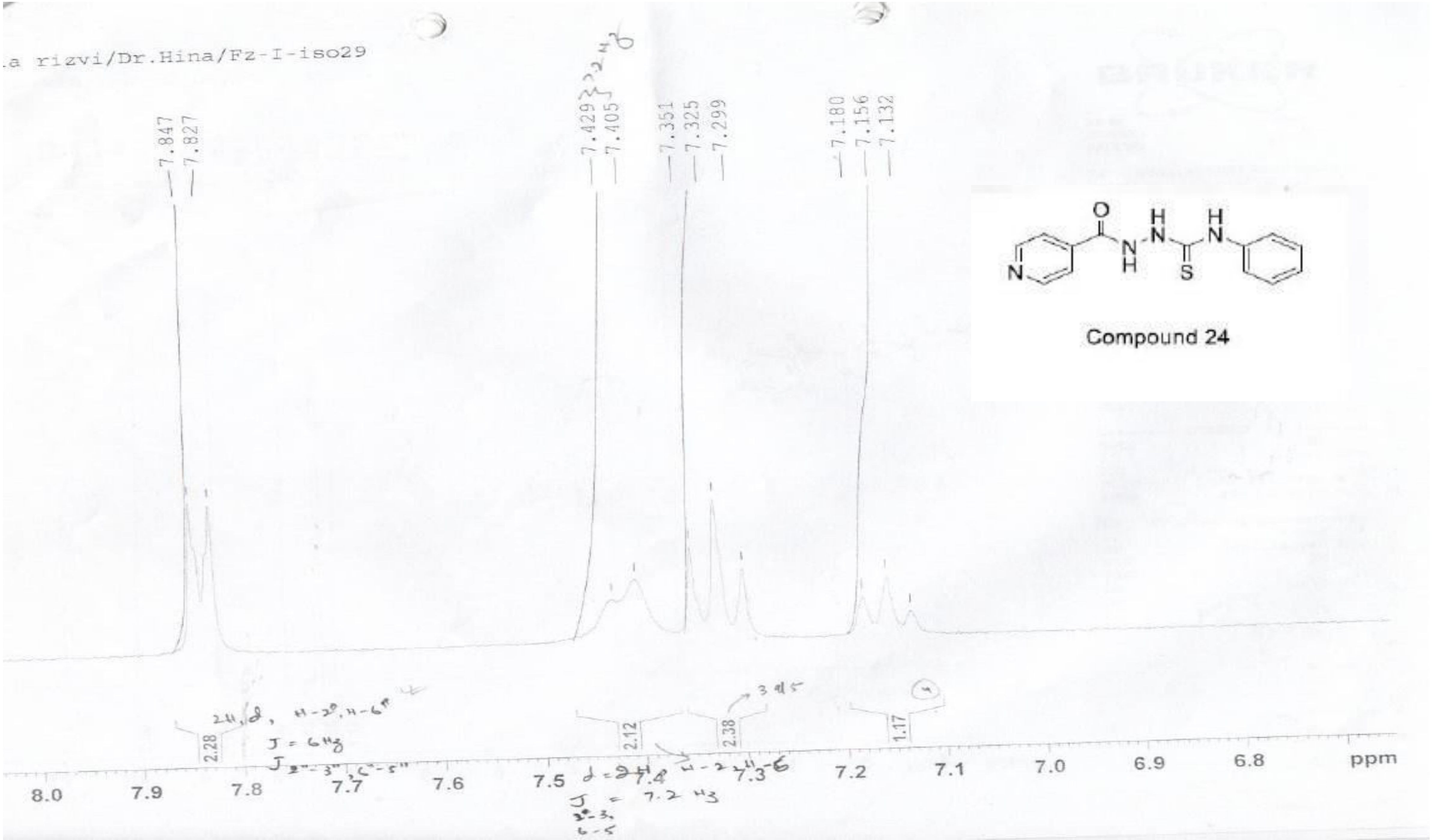
.a rizvi/Dr.Hina/Fz-I-iso29
7.847
7.827
7.429
7.405
7.351
7.325
7.299
7.180
7.156
7.132
2.28
2.12
2.38
1.17
8.0
7.9
7.8
7.7
7.6
7.5
7.4
7.3
7.2
7.1
7.0
6.9
6.8
ppm

Compound 24

HEJ-ICCBS
10/21/2016 10:37:06 AM

File: FZ-I-ISO29-FABP
Sample: bushra qamar /dr. hina
Instrument: JEOL-600H-2
Inlet: Direct Probe

Date Run: 10-21-2016 (Time Run: 10:35:30)

Ionization mode: FAB+

Scan: 7
Base: m/z 185; 37.3%FS TIC: 912828

R.T.: .53

#Ions: 66

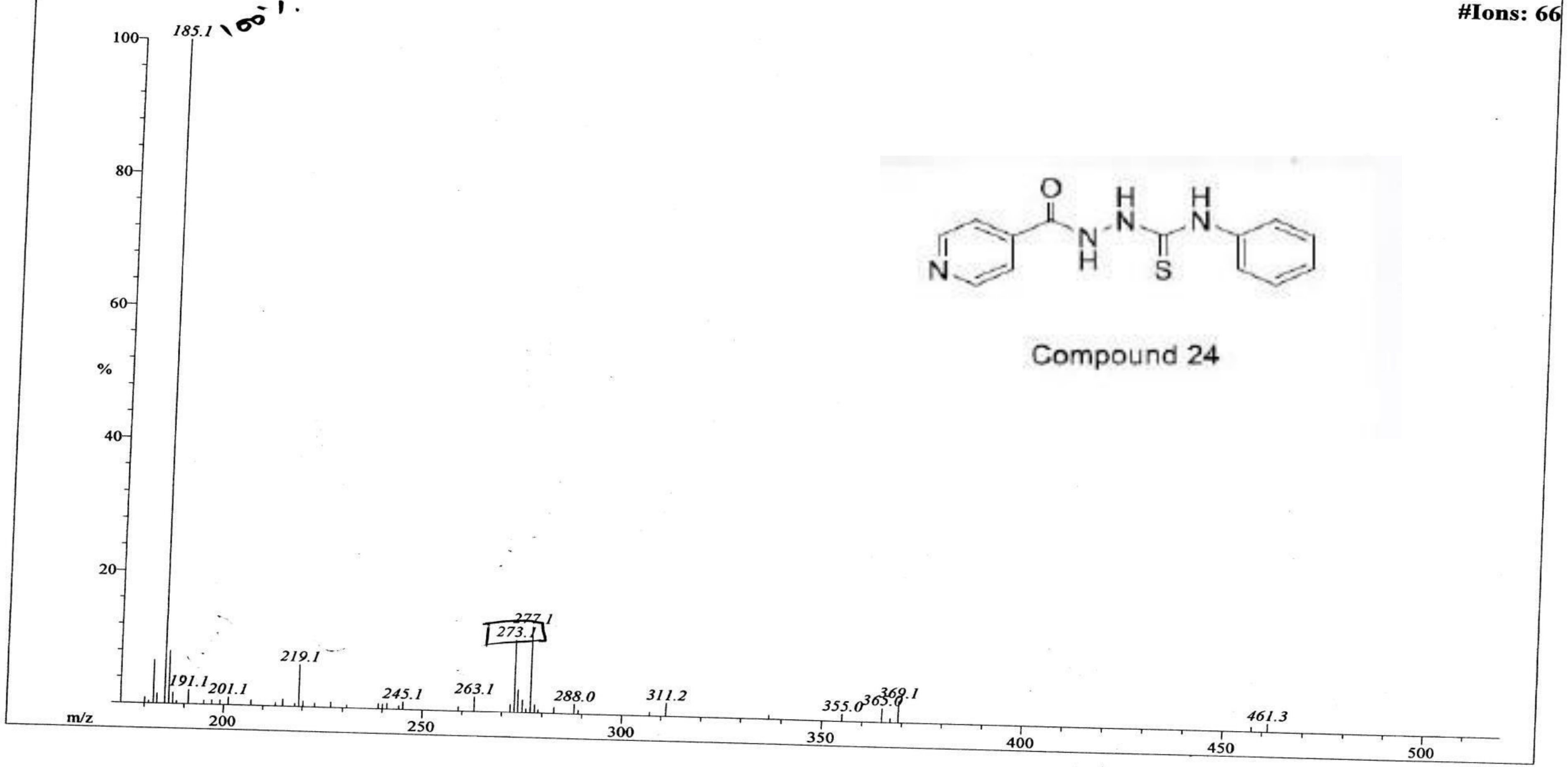

Compound 24

I. C.C.B.S., University of Ka[illegible]hi Analytical Laboratory - Pakistan

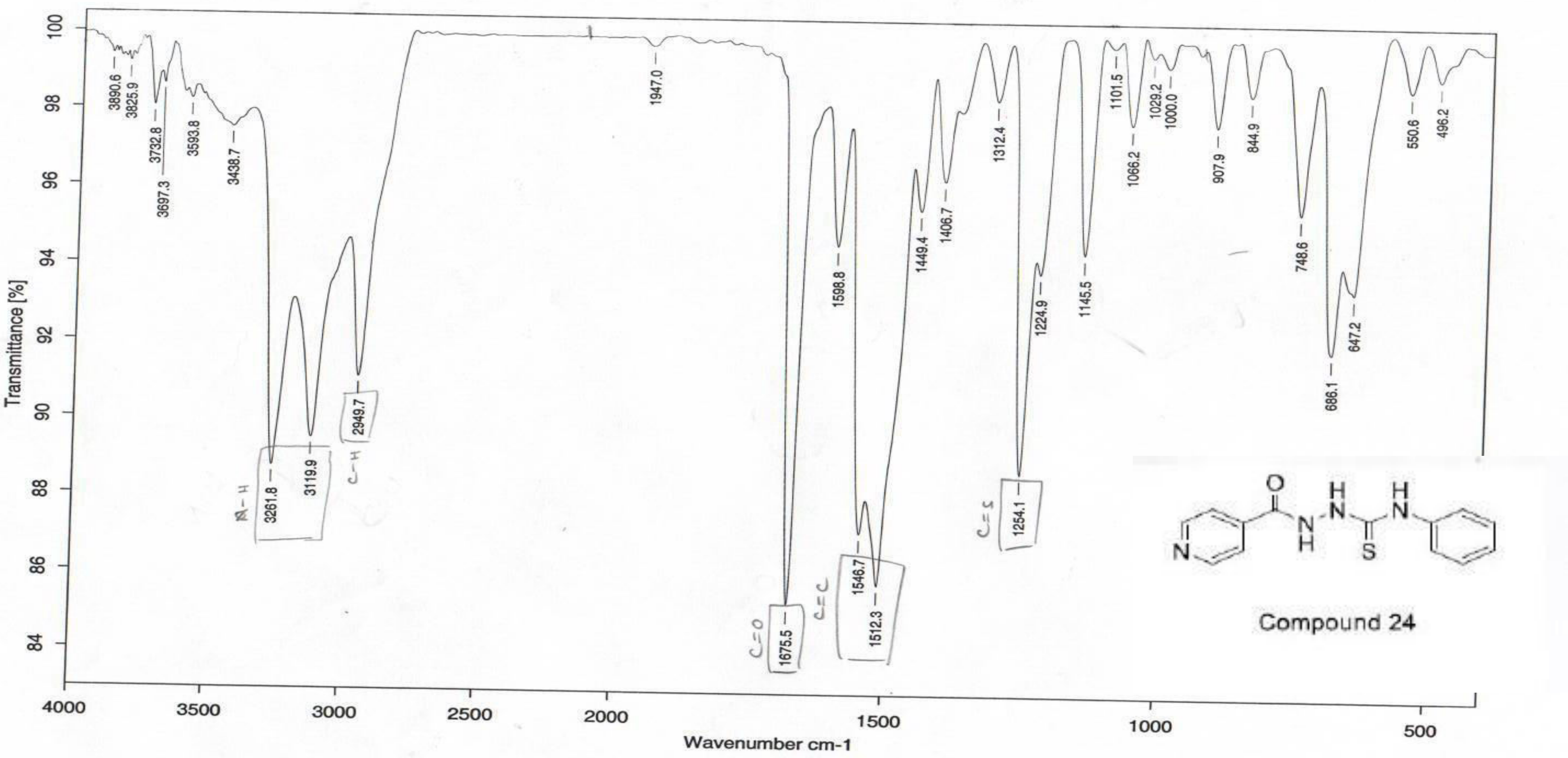

Sample : Fz-I-Iso29/Fazila Rizvi/Dr. Hina	Spectrum : Fz-I-Iso29.0 (in D:\IRSTUDENT)
Measured : 29/12/2016 on VECTOR22	Technic : Solid
Resolution : 4 cm-1 (10 scans)	Analyst : M. Asif

Fazila/DR.Hina/Fz-I-ISO30
ICCBS,U.O.K/1H

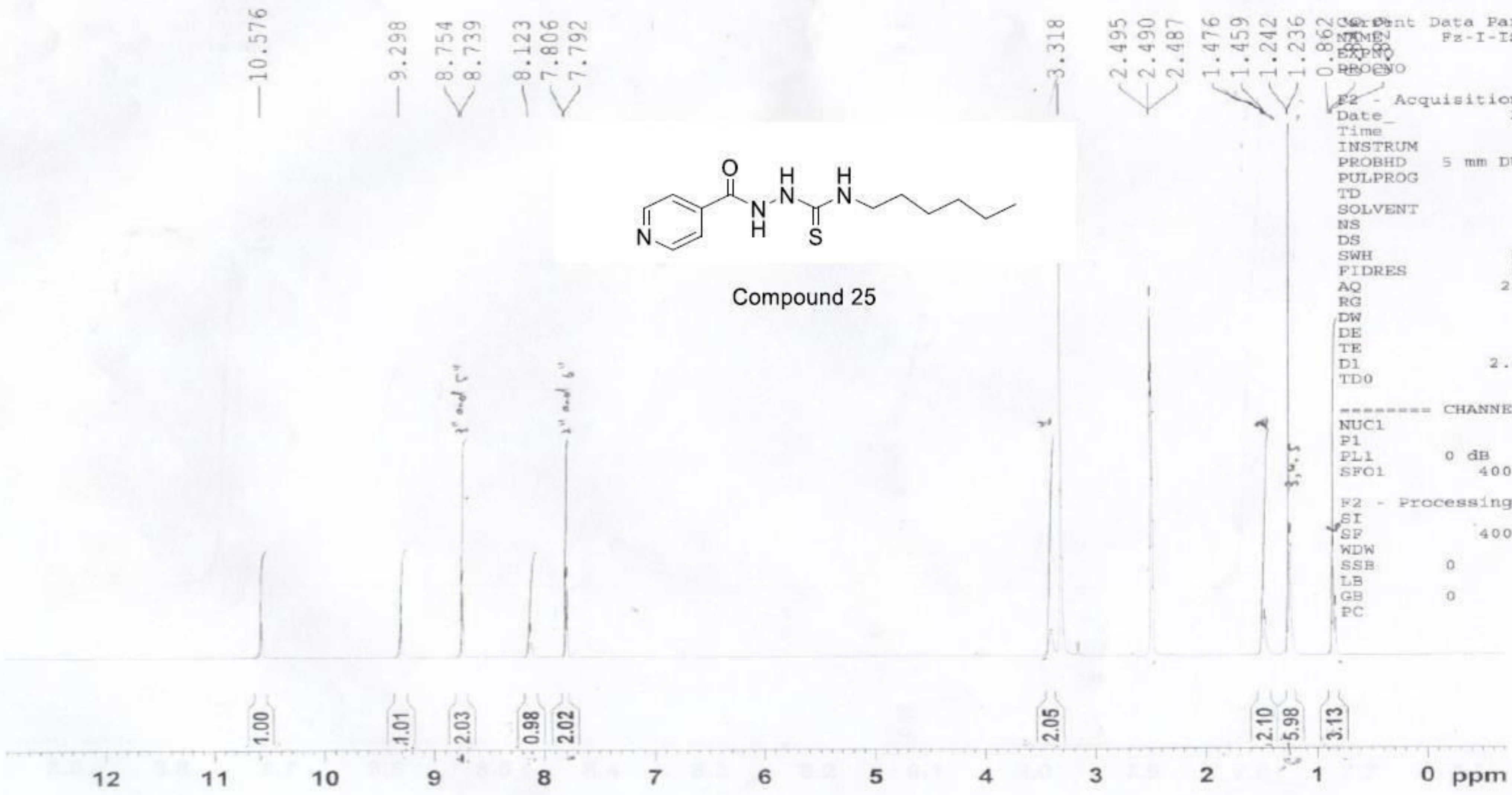

Compound 25

Current Data Parameters
NAME Fz-I-ISO30 (18 apr)
EXPNO 8
PROCNO 1

F2 - Acquisition Parameters
Date_ 20170418
Time 15.32
INSTRUM spect
PROBHD 5 mm DUL 13C-1
PULPROG zg30
TD 32768
SOLVENT DMSO
NS 64
DS 0
SWH 8012.820 Hz
FIDRES 0.244532 Hz
AQ 2.0447233 sec
RG 322.5
DW 62.400 usec
DE 6.50 usec
TE 300.0 K
D1 2.00000000 sec
TD0 1

======== CHANNEL f1 ========
NUC1 1H
P1 8.40 usec
PL1 0 dB
SFO1 400.1332010 MHz

F2 - Processing parameters
SI 16384
SF 400.1300065 MHz
WDW EM
SSB 0
LB 0.30 Hz
GB 0
PC 1.00

Fazila/DR.Hina/Fz-I-ISO30
ICCBS,U.O.K/1H

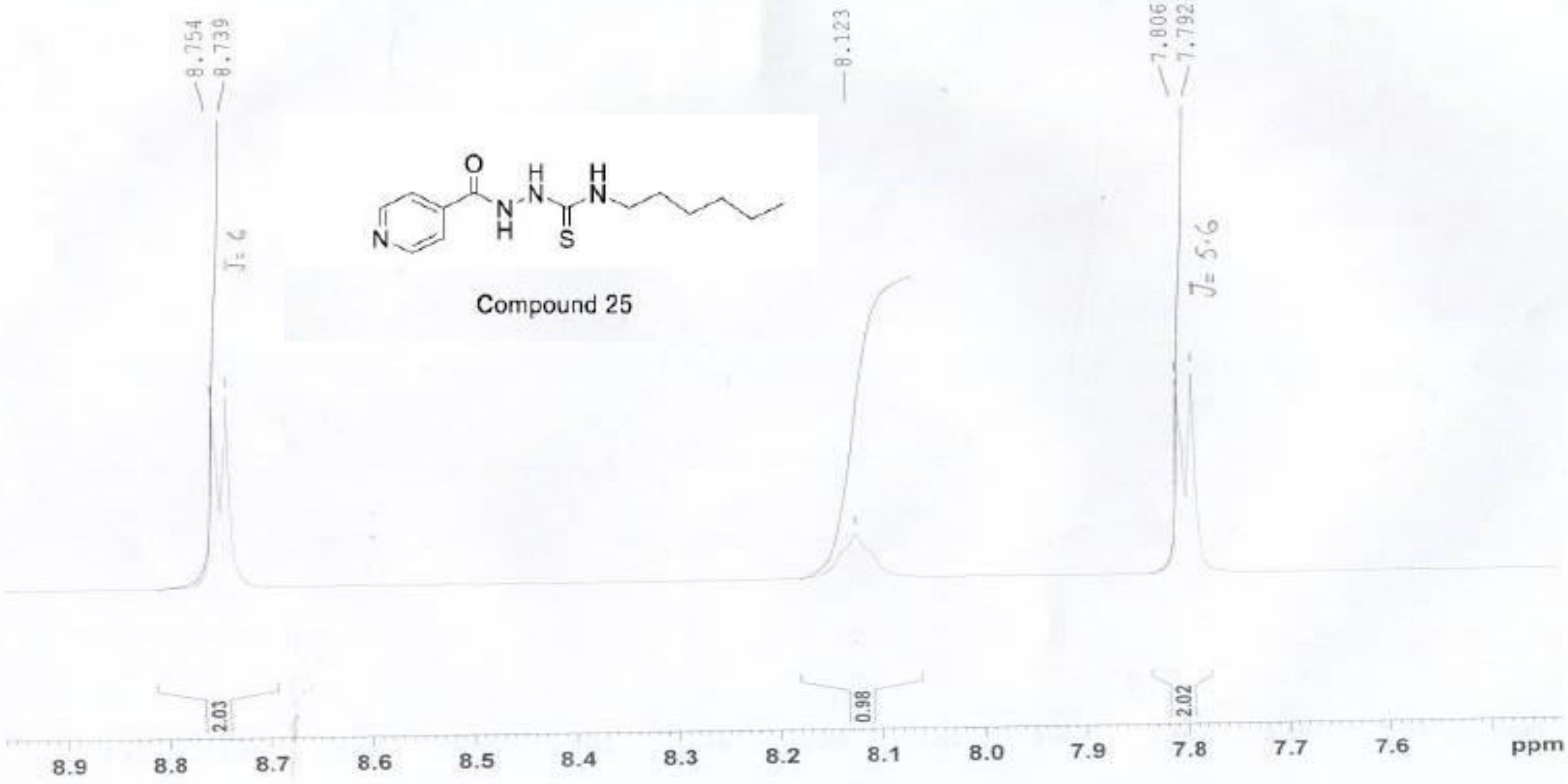

Compound 25

File: FZ-I-ISO30-FABN
Sample: BUSHRA QAMAR /DR. HINA
Instrument: JEOL-600H-2
Inlet: Direct Probe

Date Run: 10-20-2016 (Time Run: 12:51:42)

Ionization mode: FAB-

Scan: 2 **R.T.: .1**
Base: m/z 183; 99.4%FS TIC: 5607252 **#Ions: 1318**

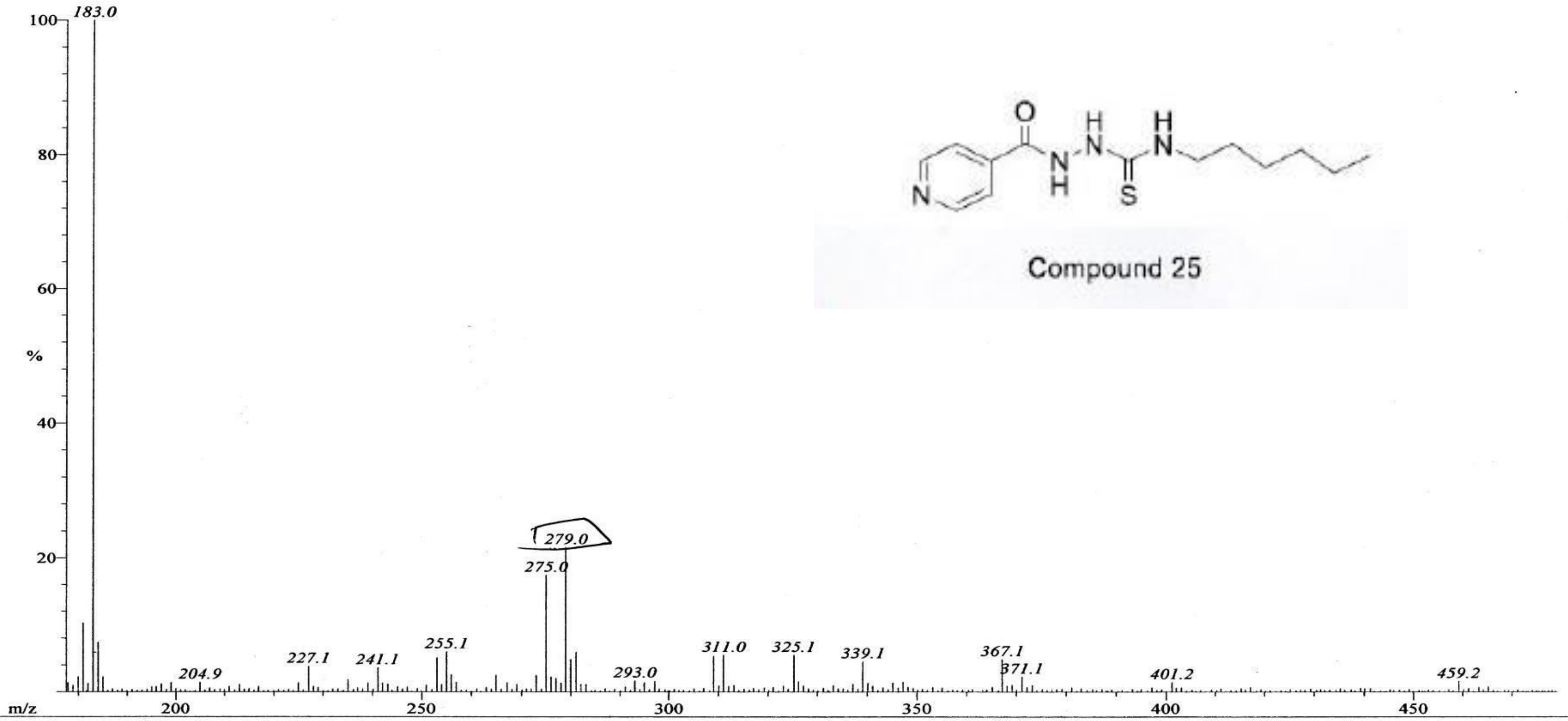

Compound 25

I. C.C.B.S., University of Ka[illegible]chi Analytical Laboratory - Pakistan

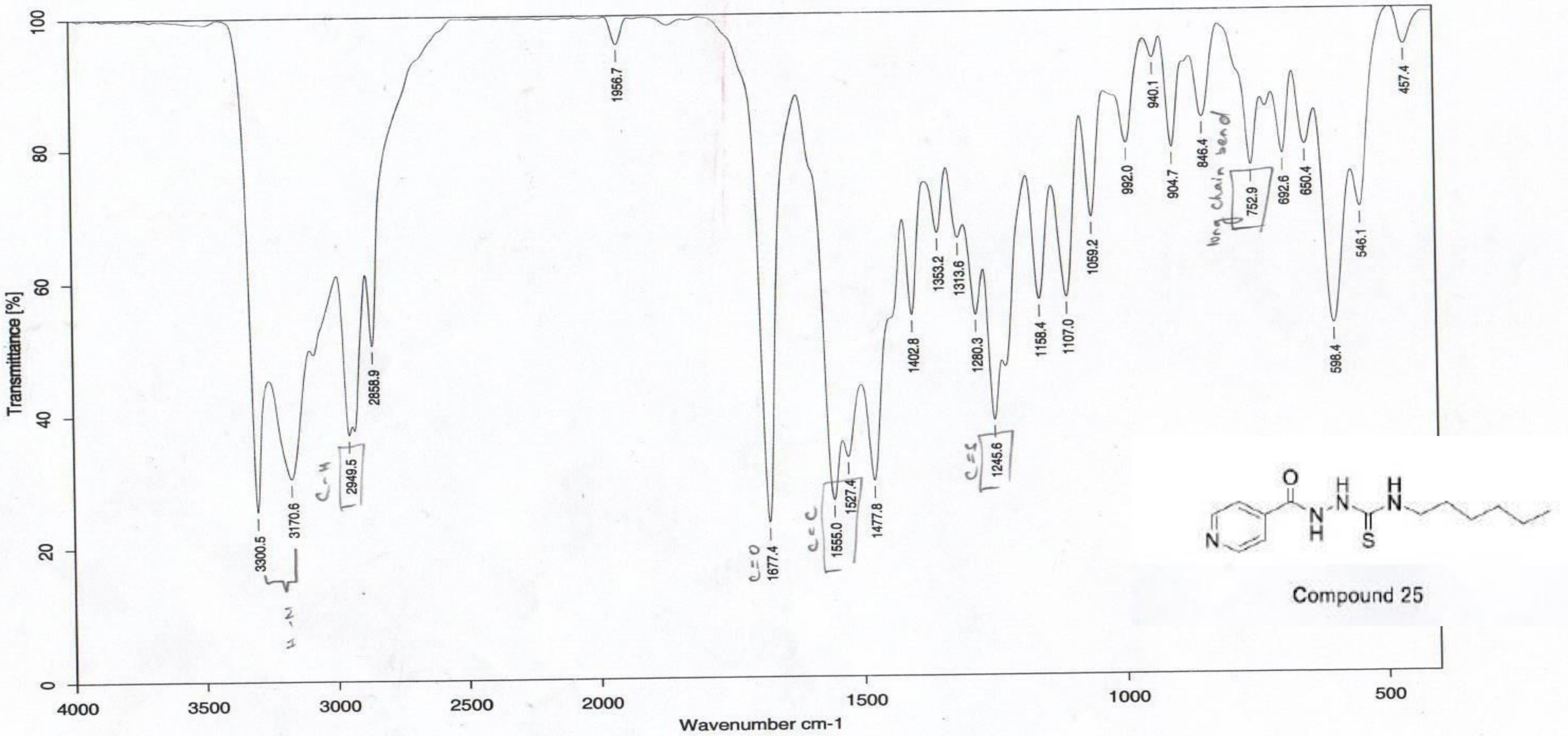

Compound 25

Sample : FZI-ISO30/Fazila Rizvi

Measured : 19/04/2017 on VECTOR22

Resolution : 4 cm-1 (10 scans)

Spectrum : FZ-I-ISO30.0 (in D:\IRSTUDENT)

Technic : Solid

Analyst : MA/ZA/JS

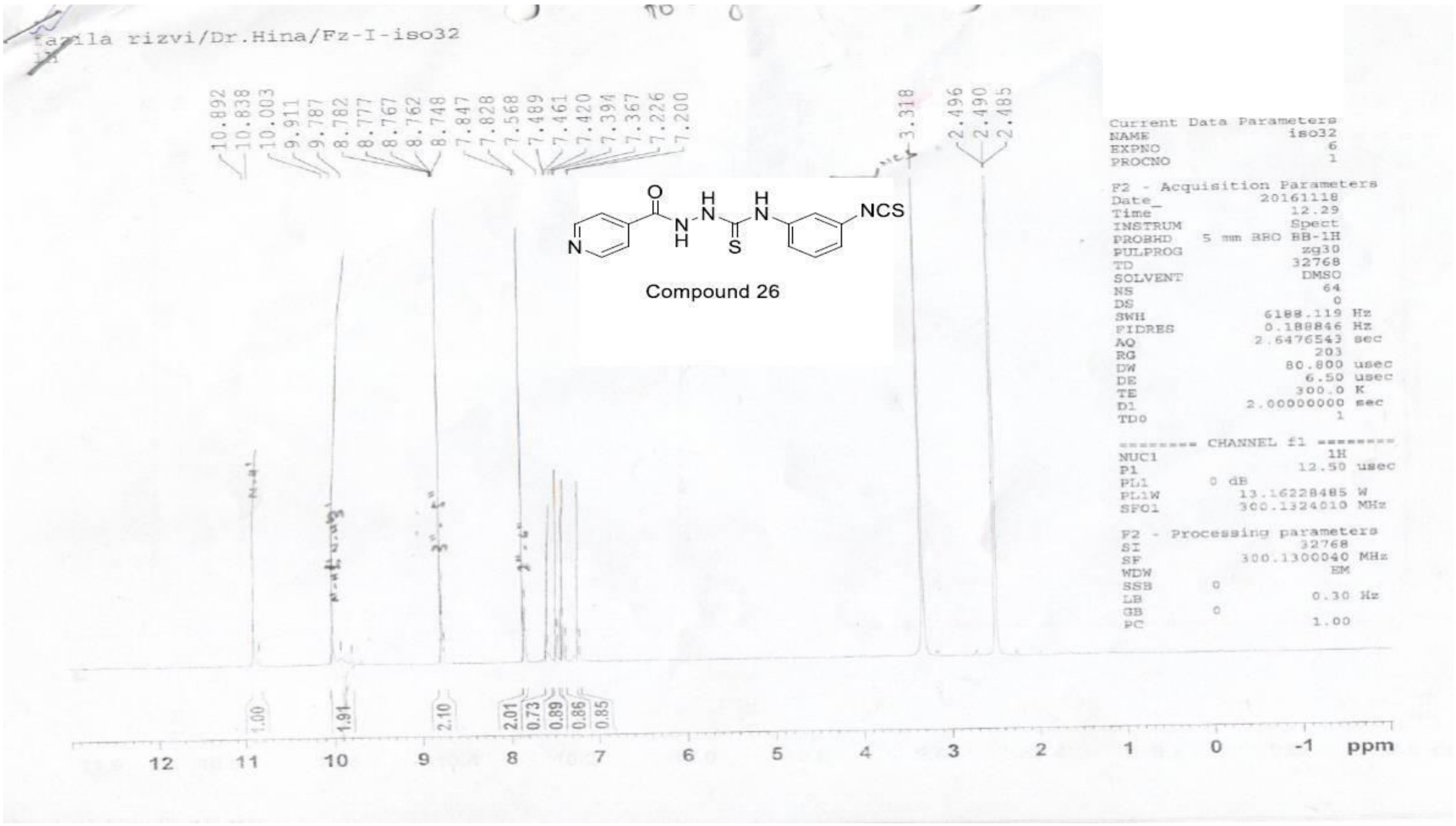
fazila rizvi/Dr.Hina/Fz-I-iso32
1H
10.892
10.838
10.003
9.911
9.787
8.782
8.777
8.767
8.762
8.748
7.847
7.828
7.568
7.489
7.461
7.420
7.394
7.367
7.226
7.200
3.318
2.496
2.490
2.485
NCS
Compound 26
1.00
1.91
2.10
2.01
0.73
0.89
0.86
0.85
12
11
10
9
8
7
6
5
4
3
2
1
0
-1
ppm
Current Data Parameters
NAME iso32
EXPNO 6
PROCNO 1
F2 - Acquisition Parameters
Date_ 20161118
Time 12.29
INSTRUM Spect
PROBHD 5 mm BBO BB-1H
PULPROG zg30
TD 32768
SOLVENT DMSO
NS 64
DS 0
SWH 6188.119 Hz
FIDRES 0.188846 Hz
AQ 2.6476543 sec
RG 203
DW 80.800 usec
DE 6.50 usec
TE 300.0 K
D1 2.00000000 sec
TD0 1
======== CHANNEL f1 ========
NUC1 1H
P1 12.50 usec
PL1 0 dB
PL1W 13.16228485 W
SFO1 300.1324010 MHz
F2 - Processing parameters
SI 32768
SF 300.1300040 MHz
WDW EM
SSB 0
LB 0.30 Hz
GB 0
PC 1.00

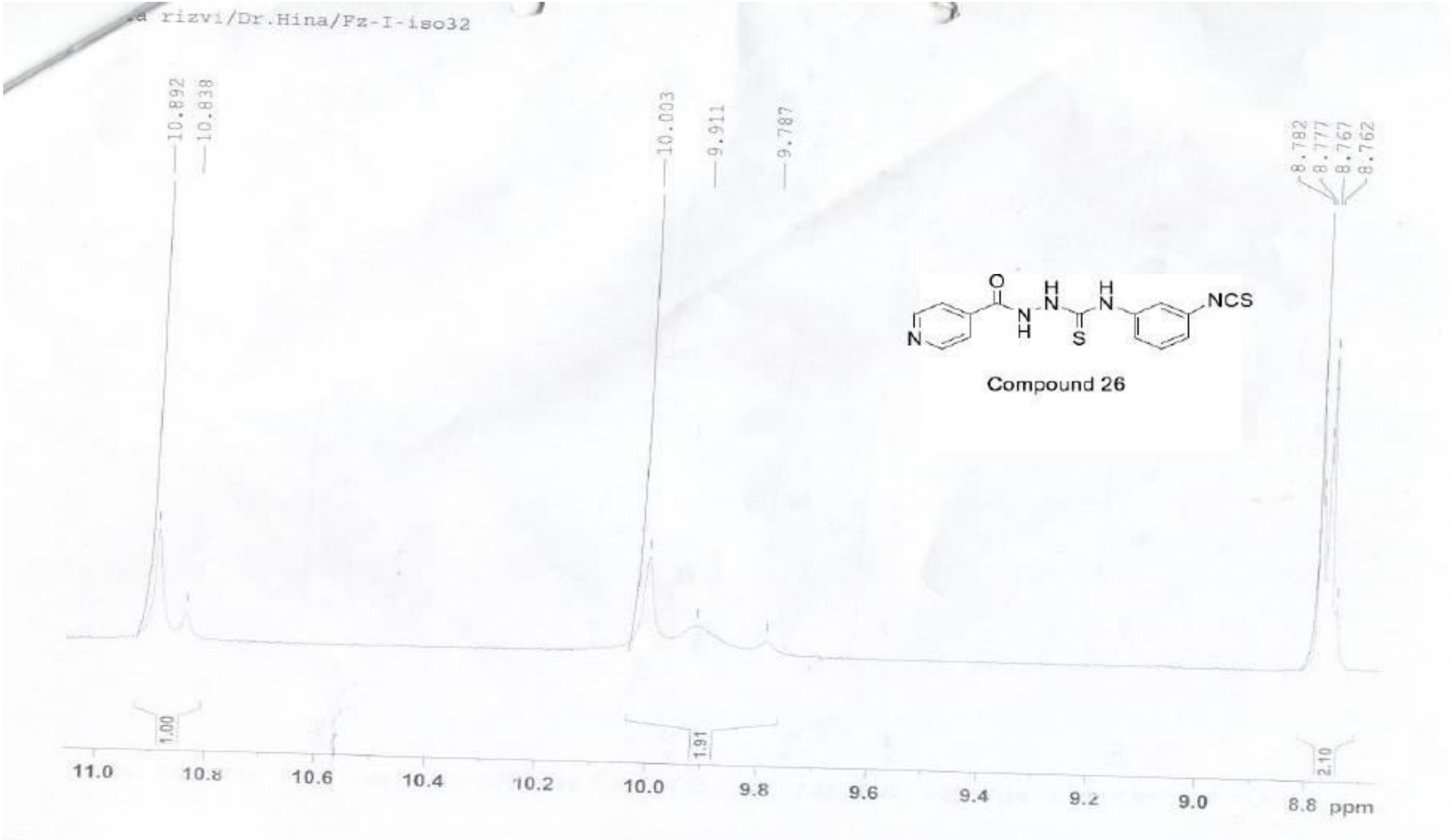
a rizvi/Dr.Hina/Fz-I-iso32
10.892
10.838
10.003
9.911
9.787
8.782
8.777
8.767
8.762
NCS
Compound 26
1.00
1.91
2.10
11.0
10.8
10.6
10.4
10.2
10.0
9.8
9.6
9.4
9.2
9.0
8.8 ppm

HEJ-ICCBS
10/27/2016 12:23:45 PM

Page 1

File: FZ-I-ISO32-FABN
Sample: BUSHRA QAMAR /DR. HINA
Instrument: JEOL-600H-2
Inlet: Direct Probe

Date Run: 10-27-2016 (Time Run: 12:13:20)

Ionization mode: FAB-

Scan: 5
Base: m/z 183; 100%FS TIC: 5774642

R.T.: .37

#Ions: 1271

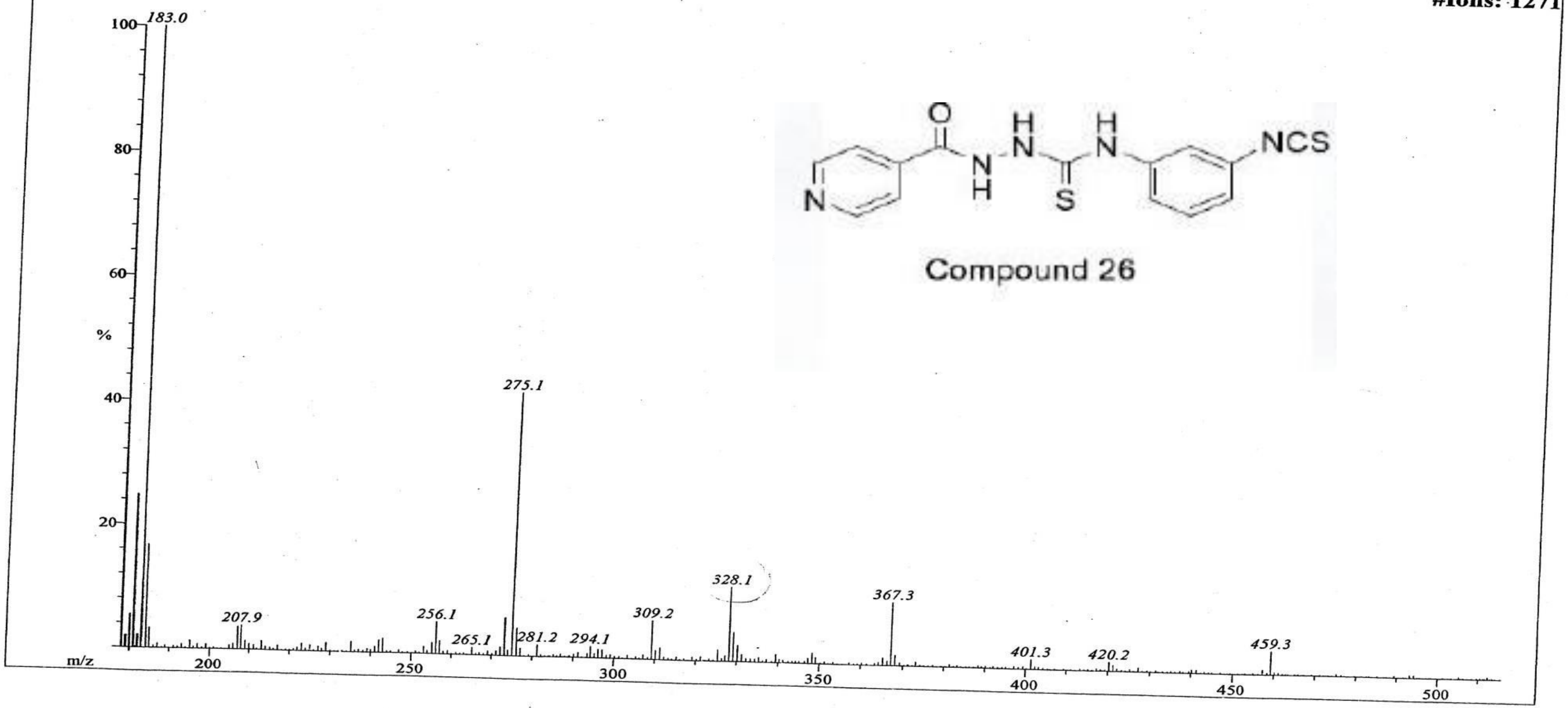

Fazila / D. Hina / FZ-I-ISO32
BB

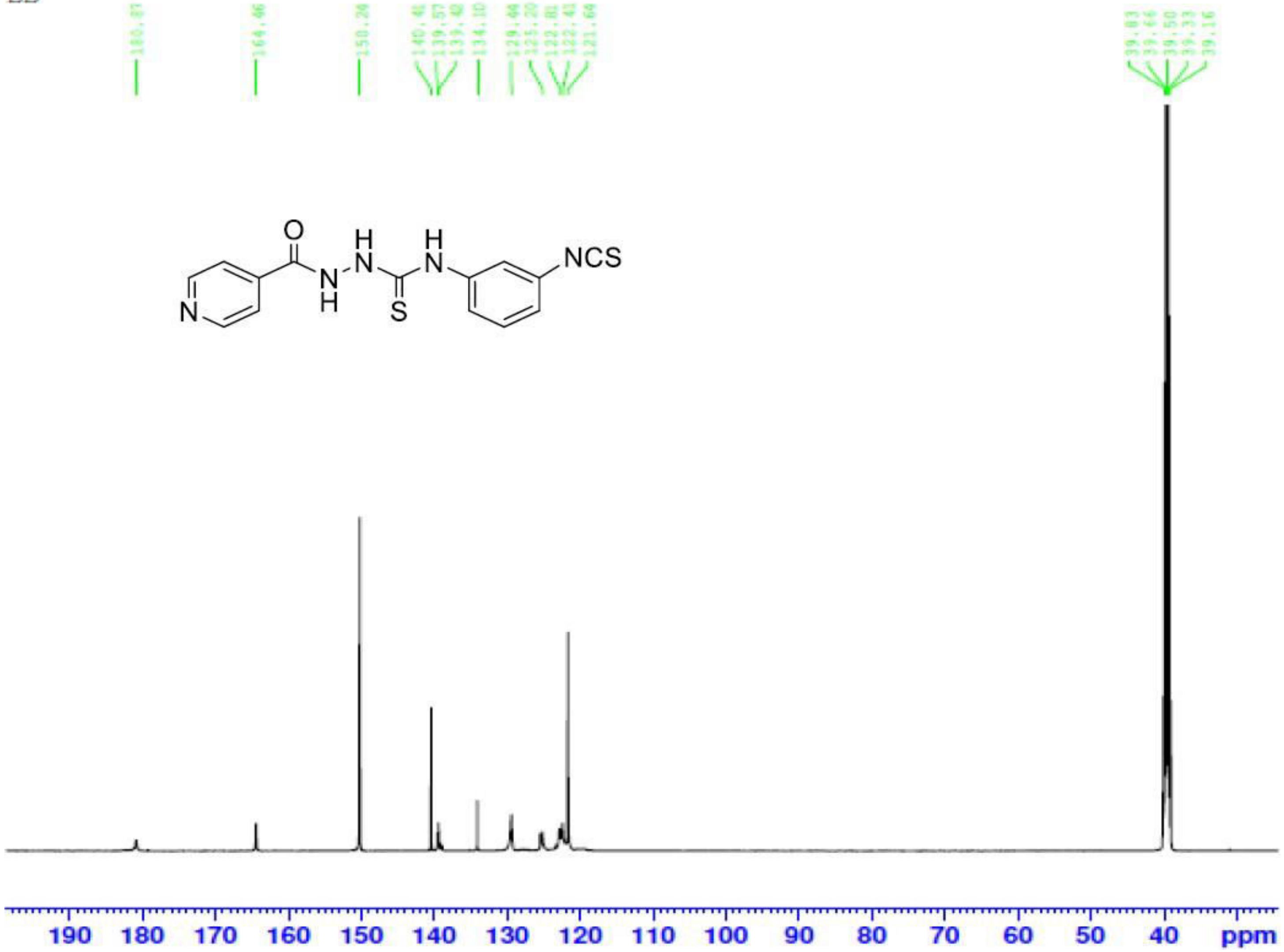

	rameters
NAME	ISO32 NMR
EXPNO	1
PROCNO	1

F2 - Acquisition Parameters

Date_	20190205
Time	23.45 h
INSTRUM	Avance Neo 500
PROBHD	Z44862_0021 (C
PULPROG	zgpg
TD	32768
SOLVENT	DMSO
NS	16384
DS	8
SWH	30120.482 Hz
FIDRES	1.838408 Hz
AQ	0.5439488 sec
RG	101
DW	16.600 usec
DE	20.00 usec
TE	298.0 K
D1	2.00000000 sec
D11	0.03000000 sec
TD0	16
SFO1	125.8227986 MHz
NUC1	13C
P1	10.00 usec
PLW1	26.46199989 W
SFO2	500.3320013 MHz
NUC2	1H
CPDPRG[2	waltz65
PCPD2	80.00 usec
PLW2	9.74149990 W
PLW12	0.34246999 W
PLW13	0.17199001 W

F2 - Processing parameters

SI	16384
SF	125.8081399 MHz
WDW	EM
SSB	0
LB	1.00 Hz
GB	0
PC	1.40

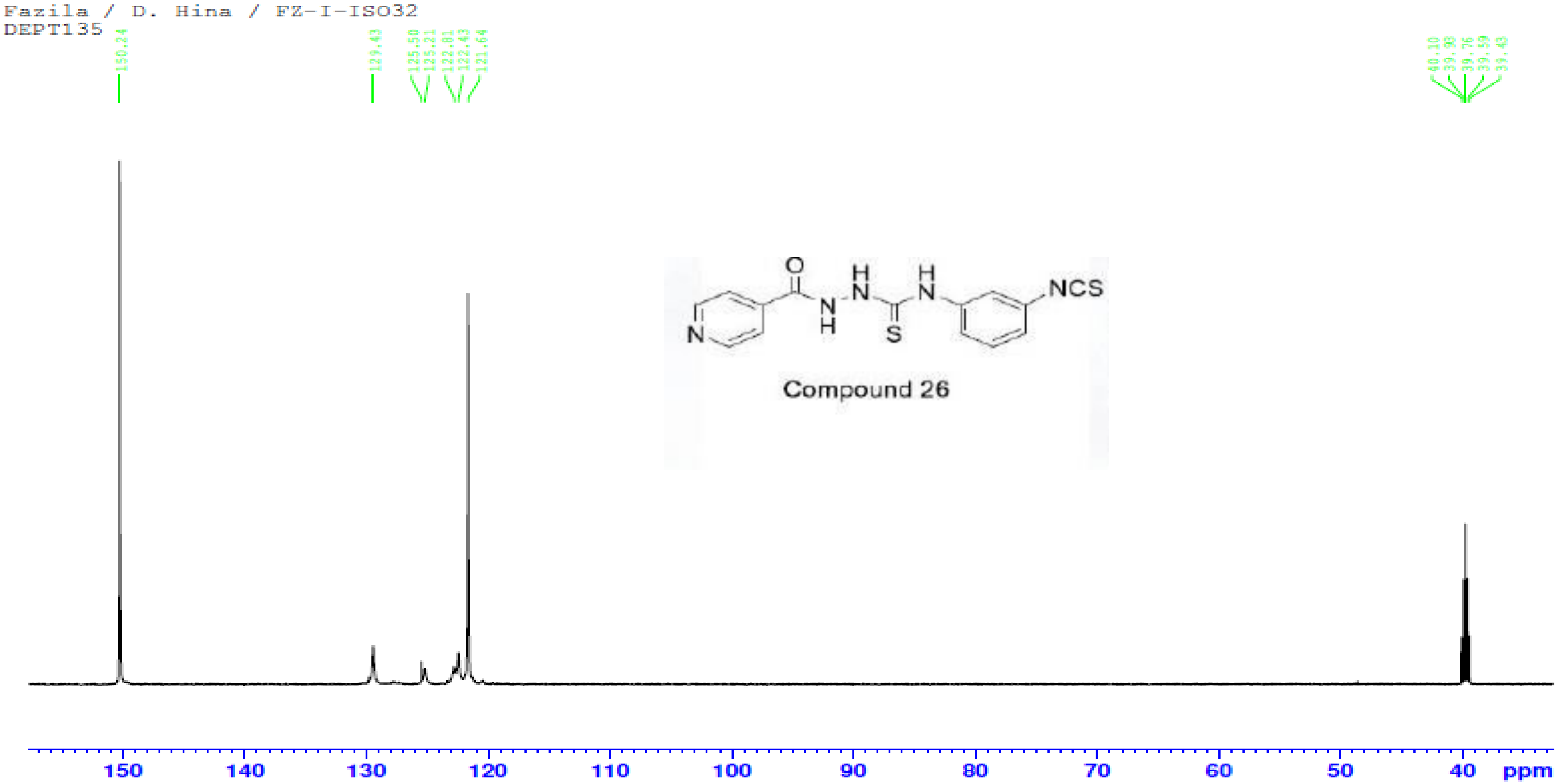
Fazila / D. Hina / FZ-I-ISO32
DEPT135
150.24
129.43
125.50
125.21
122.81
122.43
121.64
40.10
39.93
39.76
39.59
39.43
O
H
N
H
N
N
H
S
NCS
N
Compound 26
150
140
130
120
110
100
90
80
70
60
50
40
ppm

I. C.C.B.S., University of Ka[illegible]chi Analytical Laboratory - Pakistan

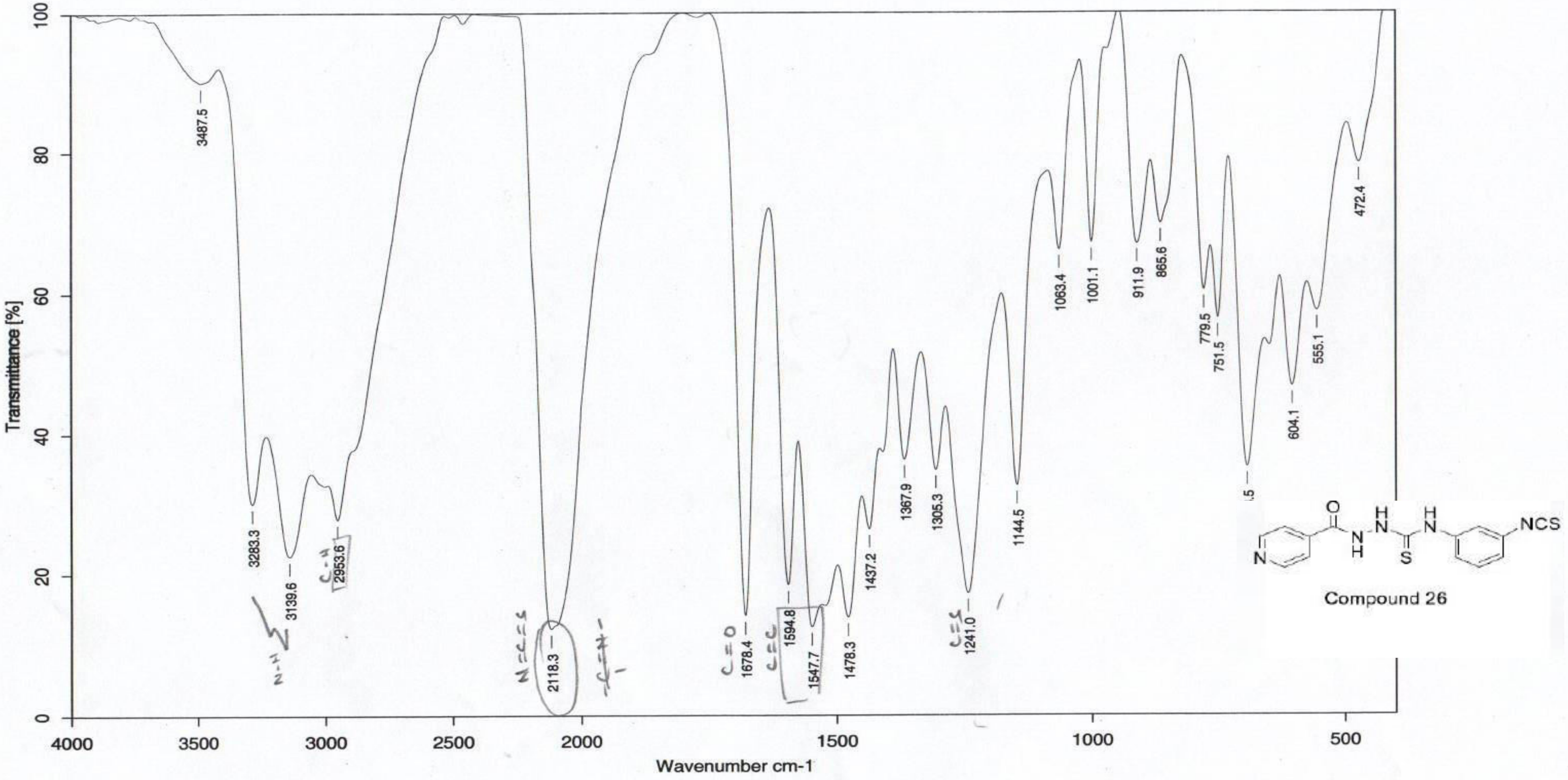

Sample : FZI-ISO32/Fazila Rizvi	Spectrum : FZ-I-ISO32.0 (in D:\IRSTUDENT)
Measured : 19/04/2017 on VECTOR22	Technic : Solid
Resolution : 4 cm-1 (10 scans)	Analyst : MA/ZA/JS

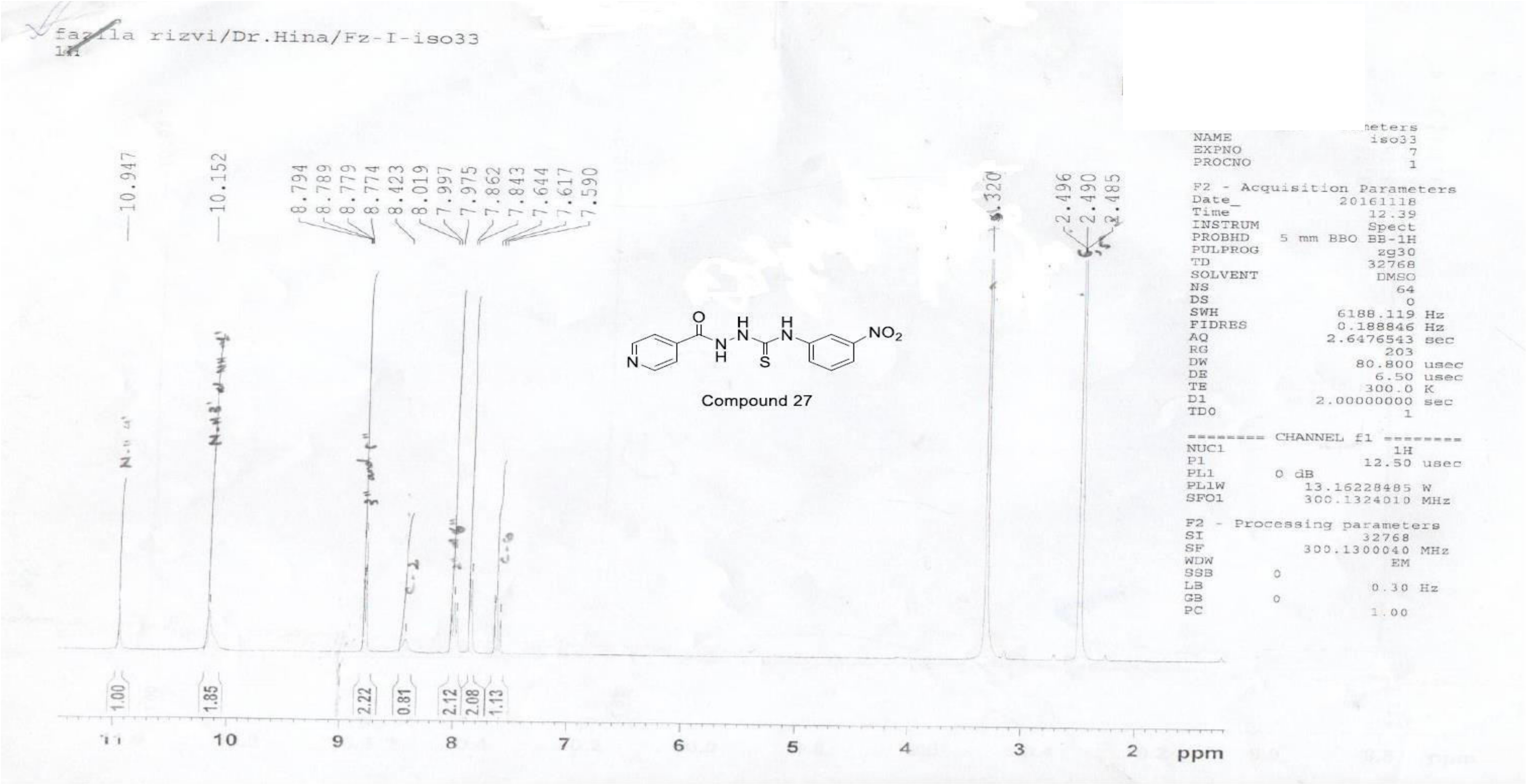
fazila rizvi/Dr.Hina/Fz-I-iso33
1H
10.947
10.152
8.794
8.789
8.779
8.774
8.423
8.019
7.997
7.975
7.862
7.843
7.644
7.617
7.590
3.320
2.496
2.490
2.485
Compound 27
NAME iso33
EXPNO 7
PROCNO 1
F2 - Acquisition Parameters
Date_ 20161118
Time 12.39
INSTRUM Spect
PROBHD 5 mm BBO BB-1H
PULPROG zg30
TD 32768
SOLVENT DMSO
NS 64
DS 0
SWH 6188.119 Hz
FIDRES 0.188846 Hz
AQ 2.6476543 sec
RG 203
DW 80.800 usec
DE 6.50 usec
TE 300.0 K
D1 2.00000000 sec
TD0 1
======== CHANNEL f1 ========
NUC1 1H
P1 12.50 usec
PL1 0 dB
PL1W 13.16228485 W
SFO1 300.1324010 MHz
F2 - Processing parameters
SI 32768
SF 300.1300040 MHz
WDW EM
SSB 0
LB 0.30 Hz
GB 0
PC 1.00
1.00
1.85
2.22
0.81
2.12
2.08
1.13
11
10
9
8
7
6
5
4
3
2
ppm

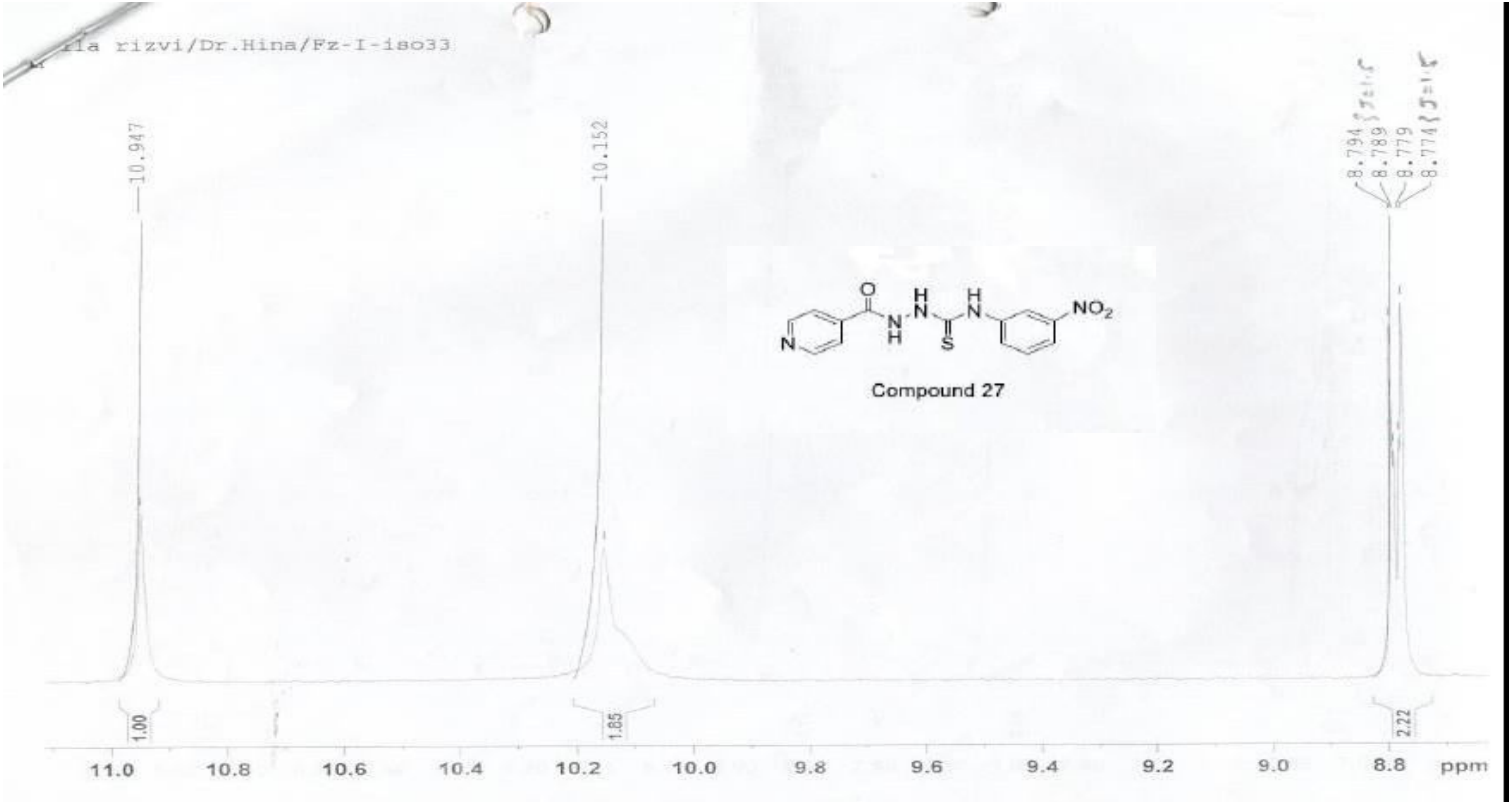
rla rizvi/Dr.Hina/Fz-I-iso33
10.947
10.152
8.794
8.789
8.779
8.774
Compound 27
1.00
1.85
2.22
11.0
10.8
10.6
10.4
10.2
10.0
9.8
9.6
9.4
9.2
9.0
8.8
ppm

File: FZ-I-ISO33-FABN
Sample: FAZILA /DR. HINA
Instrument: JEOL-600H-2
Inlet: Direct Probe

Date Run: 12-01-2016 (Time Run: 13:41:56)

Ionization mode: FAB-

Scan: 5
Base: m/z 183; 78.9%FS TIC: 2423426

R.T.: .37

#Ions: 911

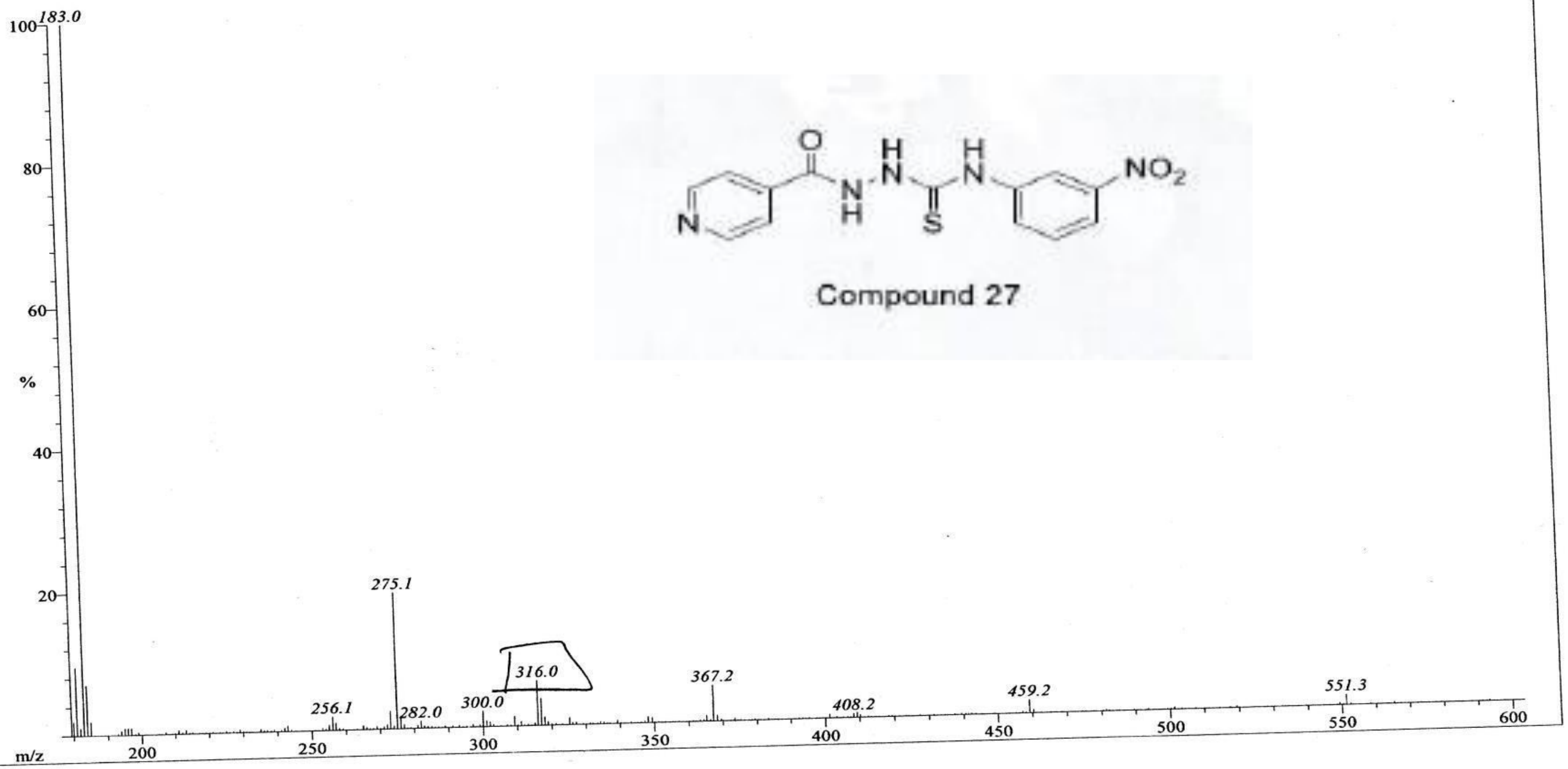

I. C.C.B.S., University of K[illegible]chi Analytical Laboratory - Pakistan

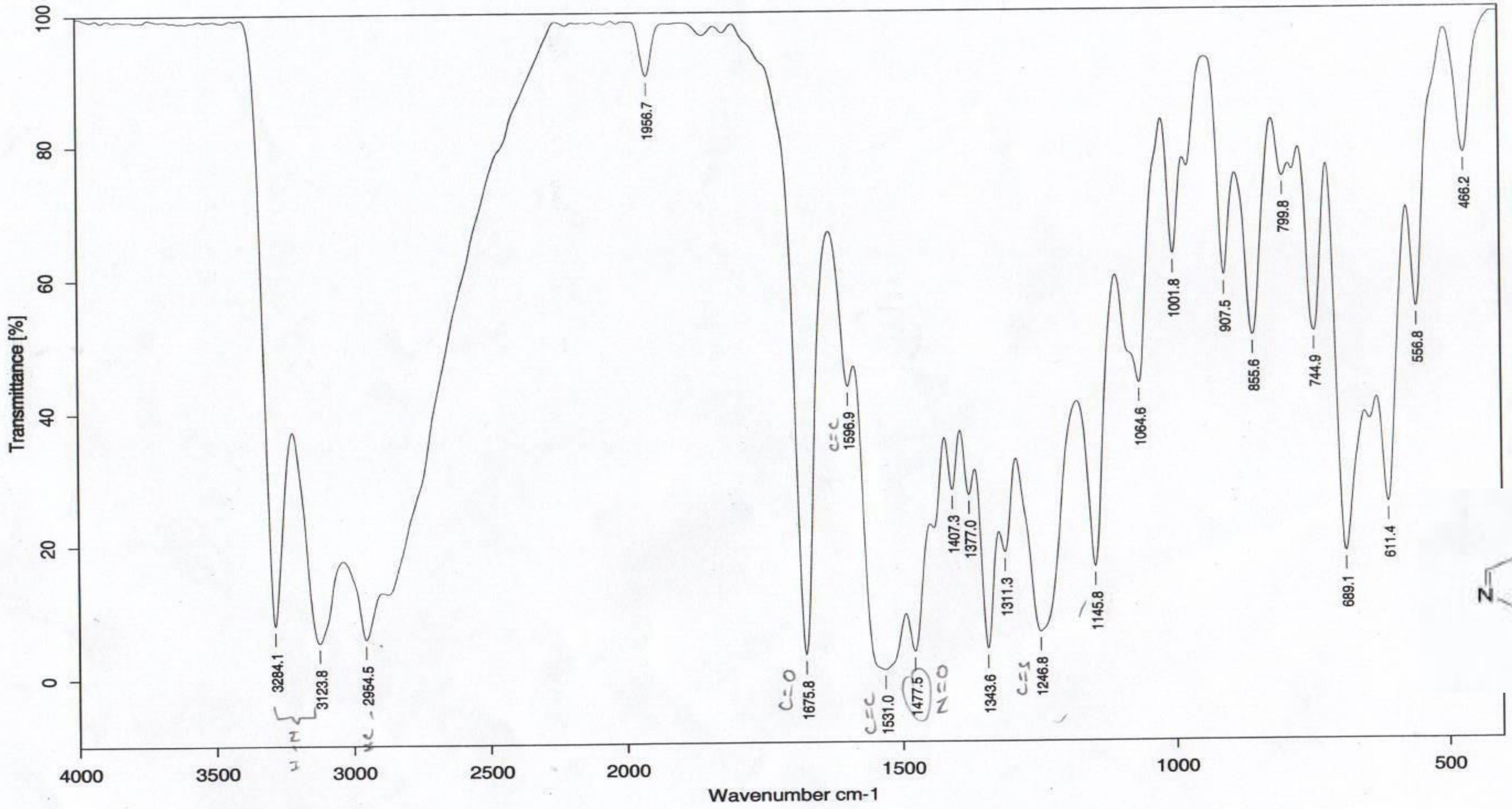

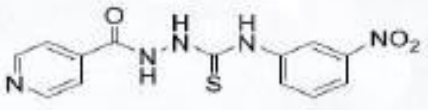

Compound 27

Sample : FZI-ISO33/Fazila Rizvi

Measured : 19/04/2017 on VECTOR22

Resolution : 4 cm-1 (10 scans)

Spectrum : FZ-I-ISO33.0 (in D:\IRSTUDENT)

Technic : Solid

Analyst : MA/ZA/JS
